# Catalysis AI Agent Guides Discovering the Universal Design Principle of Cu‐Based Single‐Atom Alloy Catalysts for CO_2_ Electroreduction

**DOI:** 10.1002/anie.202524612

**Published:** 2026-02-24

**Authors:** Xuning Wang, Zhong Li, Di Zhang, Hao Li, Haoxiang Xu, Daojian Cheng

**Affiliations:** ^1^ Beijing Key Laboratory of Intelligent Design and Manufacturing for Hydrogen Energy Materials State Key Laboratory of Organic‐Inorganic Composites Beijing University of Chemical Technology Beijing 100029 China; ^2^ College of Chemistry and Chemical Engineering Tarim University Alar City 843300 China; ^3^ Advanced Institute for Materials Research (WPI‐AIMR) Tohoku University Sendai 980–8577 Japan; ^4^ Deep Intelligence Experiment Technology (Beijing) Co., Ltd Beijing 100029 China

**Keywords:** AI Agent, CO_2_RR, Database, Rational Design, Single‐atom Alloy

## Abstract

Copper (Cu)‐based single‐atom alloys (SAAs) represent a promising strategy for optimizing the electroreduction of CO_2_ (CO_2_R) to multi‐carbon products (C_2+_). However, the diverse enhancement degrees of C_2+_ selectivity brought about by various dopants have not yet been rationalized, which lead to the absence of guidelines for further designing desired Cu‐based SAAs. Herein, guided by the Catalysis AI Agent developed based on large‐scale data + large language model, as well as the Digital Catalysis Platform (the *DigCat* experimental database), we performed first‐principles calculations to evaluate C_2+_ products selectivity trends through identifying the energy barrier of rate‐determining step (RDS) among diverse C‐C coupling pathways. With first‐principles results fed back, Catalysis AI Agent reveals that the element classification in the periodic table of guest metal dopant is essential for establishing robust structure‐selectivity correlations among Cu‐based SAAs. A structural descriptor (φ) is developed and helps to establish a strong correlation among the electronic‐scale structural features, the adsorption strength of C‐C coupling precursors, and the macroscopic C_2+_ products selectivity. A universal design principle based on φ for Cu‐based SAAs enables the rapid and qualitative evaluation of C_2+_ selectivity, which is fully supported by most of the experimental references and our experimental verification.

## Graphical abstract

Artificial intelligence (AI) enables the systematic analysis and comparative evaluation of experimental and theoretical data, optimizes the catalytic reaction research workflow, and accelerates the discovery of high‐performance electrocatalysts.

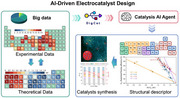

## 1 Introduction

**FIGURE 1:**
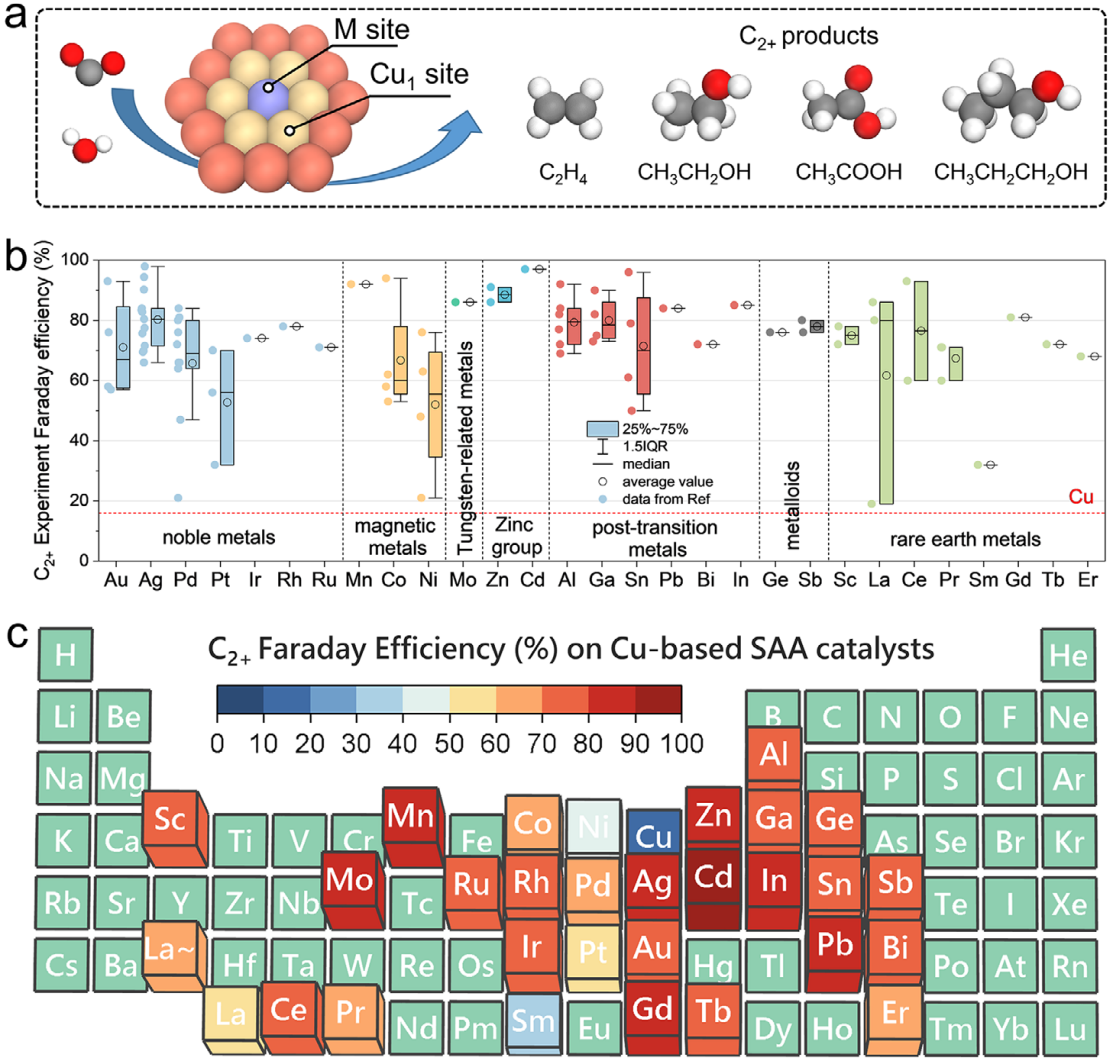
(a) Schematic diagram of reaction site types of SAAs. (b) Summary of experimental Faraday efficiency of multi‐carbon products (C_2+_) from CO_2_RR over Cu‐based SAAs in the Digital Catalysis Platform (*DigCat*) database [4]. (c) Experimental Faraday efficiency of C_2+_ products on Cu‐based SAAs (expressed as the average of different reports) in the form of periodic table with foreign elements. Red: high selectivity zone; blue: low selectivity zone; green: unreported or nonmetallic elements. Detailed references and data are shown in Table S1 of the Supporting Information (SI).

Electrochemical CO_2_ reduction reaction (CO_2_RR) for synthesizing multi‐carbon (C_2+_) compounds powered by renewable electricity has been emerged as a transformative strategy for carbon‐neutral chemical production. Cu stands as the sole metal element demonstrating intrinsic capability for generating C_2+_ products through CO_2_RR [1, 2, 3]. Nevertheless, conventional monometallic Cu electrocatalysts suffer from suboptimal catalytic performance, primarily due to the inherent kinetic limitations in C‐C coupling processes. Cu‐based single‐atom alloys (SAAs) present a promising solution to overcome these limitations via precise incorporation of isolated trace metal elements (Figure 1a). Experimental evidence demonstrates that precisely controlled dopant introduction enables tailored modification of Cu active sites’ electronic structure and optimization of local coordination environments, thereby significantly enhancing C‐C coupling kinetics.

Building upon big data statistics of experimental literatures stored in the Digital Catalysis Platform (DigCat [4, 5], https://www.digcat.org), as comprehensively summarized in Figures 1b and 1c about C_2+_ products Faraday efficiency, we presented a systematic classification of Cu‐based SAAs spanning various guest metals as dopant. The Faraday efficiencies of other products and related analyses are presented in the Supporting Information (Figures S1–S3 and Table S1). The dataset comprises ∼80 data points derived from ∼50 experimental studies and encompasses 29 distinct elements of dopants. The majority of the experimental performance results were obtained using a flow‐cell reactor with KOH electrolyte, along with C_2+_ Faraday efficiency measurements conducted at potentials ranging from ‐0.7 to ‐1.3 V vs. RHE (Figure S3e–g). It demonstrates the enhanced selectivity toward ethylene or other C_2+_ products during CO_2_RR (the average Faraday efficiency of C_2+_ >50%). The experimentally reported dopant elements encompass transition metals (noble metals: Au, Ag, Pd, Pt, Ir, Rh, Ru; magnetic elements: Mn, Co, Ni; Tungsten(W)‐related metals: Mo and Zinc(Zn)‐group metals: Zn, Cd); post‐transition metals (*i.e*., Al, Ga, Sn, Pb, Bi, In); metalloids (*i.e*., Ge, Sb) and Lanthanide rare earth metals (*i.e*., Sc, La, Ce, Pr, Sm, Gd, Tb, Er). This diversity highlights the universal efficacy of Cu‐based SAAs in enhancing C_2+_ products formation. Notably, a minor subset of the experimental data originates from Cu‐based dilute alloy catalysts. Given the ultralow dopant concentrations, these dopants also exist as isolated sites on the Cu surface according to the experimental characterization images [6, 7, 8, 9, 10, 11], justifying their treatment as approximate SAA configurations. Notably, the green region in Figure 1c represents nonmetallic dopants or dopants without reported experimental work, rather than areas of low catalytic activity. Although the present study does not specifically aim to investigate nonmetallic dopants, these species may play a significant role in enhancing the selectivity toward C_2+_ products [12, 13, 14].

Mechanistic studies employing density functional theory (DFT) or operando spectroscopy reveal that isolated dopants in Cu‐based SAAs modulate C‐C coupling through fine‐tuning of intermediate adsorption strengths or introducing alternative coupling precursor species, thereby lowering kinetic barriers. For instance, the Cu‐based SAAs doped with noble metal elements lower the energy barrier of CO‐CHO or CO‐COH coupling process by facilitating the formation of CHO or COH intermediates. Ag_1_/Cu(111) SAAs enhance CHO* stabilization to enable CO‐CHO dimerization [15], while Pd_1_/Cu(111) SAAs facilitate COH*‐mediated CO‐COH coupling [16]. In contrast, rare earth dopants (*i.e*., La [17], Gd [18], Ce [19]) preferentially reduce CO dimerization barriers without generating new precursors. However, controversies persist regarding specific coupling pathways. Wei et al., [20]. attributed C_2+_ production to CO* dimerization on AgCu (with CHO* yielding C_1_ by‐products), contradicting earlier CO‐CHO pathway observations. Similarly, Chhetri et al., [21]. identified CO‐CHO coupling instead of COH*‐mediated coupling as the dominant pathway on Pd_1_/Cu(111). Additionally, researchers have also observed CO‐CHO coupling in Pr‐doped Cu systems [22] despite rare earths' typical CO‐CO preference, suggesting potential dopant‐dependent C‐C coupling mechanism switching. Moreover, post‐transition metal‐doped SAAs exhibit hybrid mechanisms (CO‐CO, CO‐CHO, CO‐COH) influenced by dopant electronegativity. However, most current investigations predominantly emphasize demonstrating the superiority of specific Cu‐based SAAs over pure Cu through examinations of certain individual C‐C coupling mechanisms. Such narrowly focused methodology has led to fragmented insights into the C‐C coupling process, resulting the prevailing inconsistencies among reported enhancement mechanisms across various Cu‐based SAAs. These inconsistencies highlight the necessity to systematically reveal periodic trends in dopant‐mediated mechanisms and establish quantitative structure‐selectivity relationships to advance the rational design of Cu‐based SAAs.

In this work, guided by a well‐trained Catalysis AI Agent based on a large language model (LLM) and scientific data, we systematically investigated CO_2_RR mechanisms across Cu‐based SAA catalysts incorporating transition metals, post‐transition metals, and metalloids as dopants through DFT calculations. Initially, according to the analysis of AI Agent on *DigCat*’s experimental big data statistics, we identified the primary research objectives and developed targeted computational strategies, specifically focusing on the C‐C coupling elementary steps toward multi‐carbon products. Subsequently, we performed a comprehensive analysis of both experimental and computational data by the AI Agent, which revealed that classifying guest dopants is essential for elucidating the structure‐selectivity relationship. Accordingly, we introduced an energy descriptor (($E_{CO*}^{ads}\left(M-Cu_{1}\right)$ or $E_{CHO*}^{ads}\left(M-Cu_{1}\right)$) to categorize Cu‐based SAAs into five distinct groups. The energy descriptor demonstrates a linear correlation with the activation energy barriers of rate‐determining step for C_2+_ formation (Ea‐C_2+_), across different classes of Cu‐based SAAs. Ultimately, we proposed a structural descriptor (φ) that quantitatively links electronic‐scale structural features, adsorption behavior, and the macroscopic selectivity of C_2+_ products. These theoretical predictions based on φ exhibit remarkable consistency with C_2+_ Faraday efficiency trends among most of the available experimental references reported to date. A universal design principle based on φ can be further extended to Cu‐based SAAs doped with rare earth elements and dual single‐atom alloys (DSAAs), rationalizing available experimental observations. It also identifies the newly discovered M_1_/Cu(111) (M = Y, Lu) SAAs and Ni_1_Zn_1_/Cu, Rh_1_Zn_1_/Cu DSAAs as promising candidates with high C_2+_ selectivity.

## 2 Results and discussion

**SCHEME 1:**
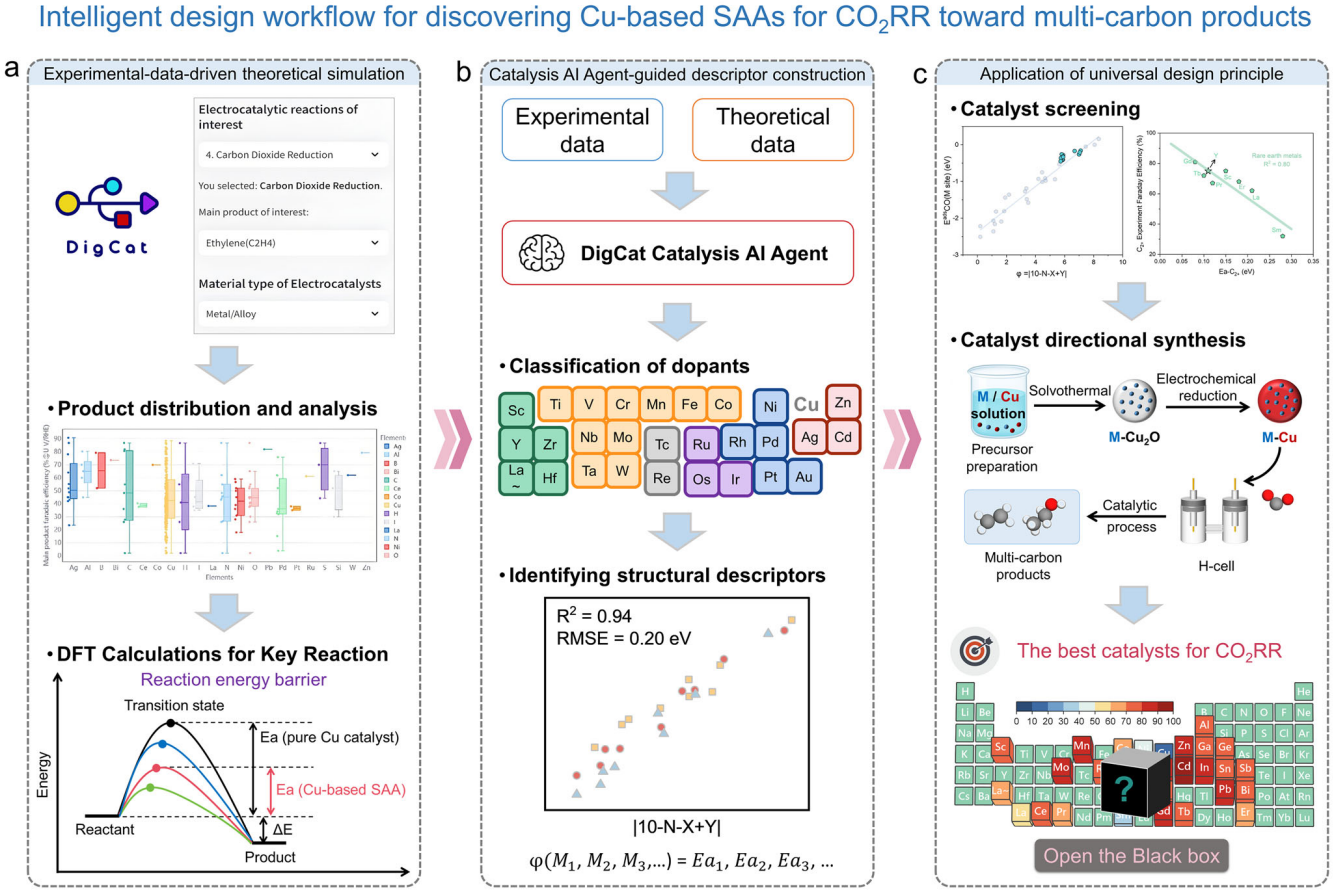
Intelligent design workflow for discovering Cu‐based SAA electrocatalysts for CO_2_RR toward multi‐carbon products. (a) Experimental‐data‐driven theoretical simulations. (b) Catalysis AI Agent‐aided descriptor construction. (c) Application of universal design principle.

The integrated workflow employed in this study (Scheme 1) comprises three stages, namely experimental‐data‐driven theoretical simulation, Catalysis AI Agent‐guided descriptor construction and application of universal design principle [5, 23, 24]. In the first step (Scheme 1a), we conducted experimental data mining using the “CO_2_ Reduction” module of the *DigCat* platform (with >4,000 experimental CO_2_RR electrocatalysts’ data entries, including catalyst formula, element composition, content ratio, structural property, electrolytes, reaction conditions such as temperature, pressure, pH, and cell type, main and secondary products with Faraday efficiencies, current densities, stability and durability metrics, as well as publication year), which enabled rapid identification of products distribution differences between Cu‐based SAAs and pure Cu catalyst, directing us to focus primarily on C‐C coupling elementary steps. For the second step (Scheme 1b), DFT calculations were conducted to determine the stability of the catalyst model, along with the thermodynamic and kinetic parameters of the C‐C coupling reaction. Subsequently, the Catalysis AI Agent developed based on LLM quickly analyzed the structure‐selectivity relationships by correlating experimental and simulation data. Interestingly, the AI Agent directly identified that dopants classification is a prerequisite, helping to identify catalyst structural descriptors and accelerated the establishment of universal catalyst design principles. During the third step (Scheme 1c), we screened and identified novel and highly efficient catalysts based on the established universal design principles. Predicted candidates were synthesized and we evaluated their electrochemical activity and selectivity of CO_2_RR. This AI‐integrated workflow significantly optimizes catalytic research planning, structure‐performance relationship establishment, and efficient catalyst screening, accelerating the discovery of high‐performance electrocatalysts.

### 2.1 Thermodynamic and Electrochemical Stability Analysis of Cu‐based SAAs

**FIGURE 2:**
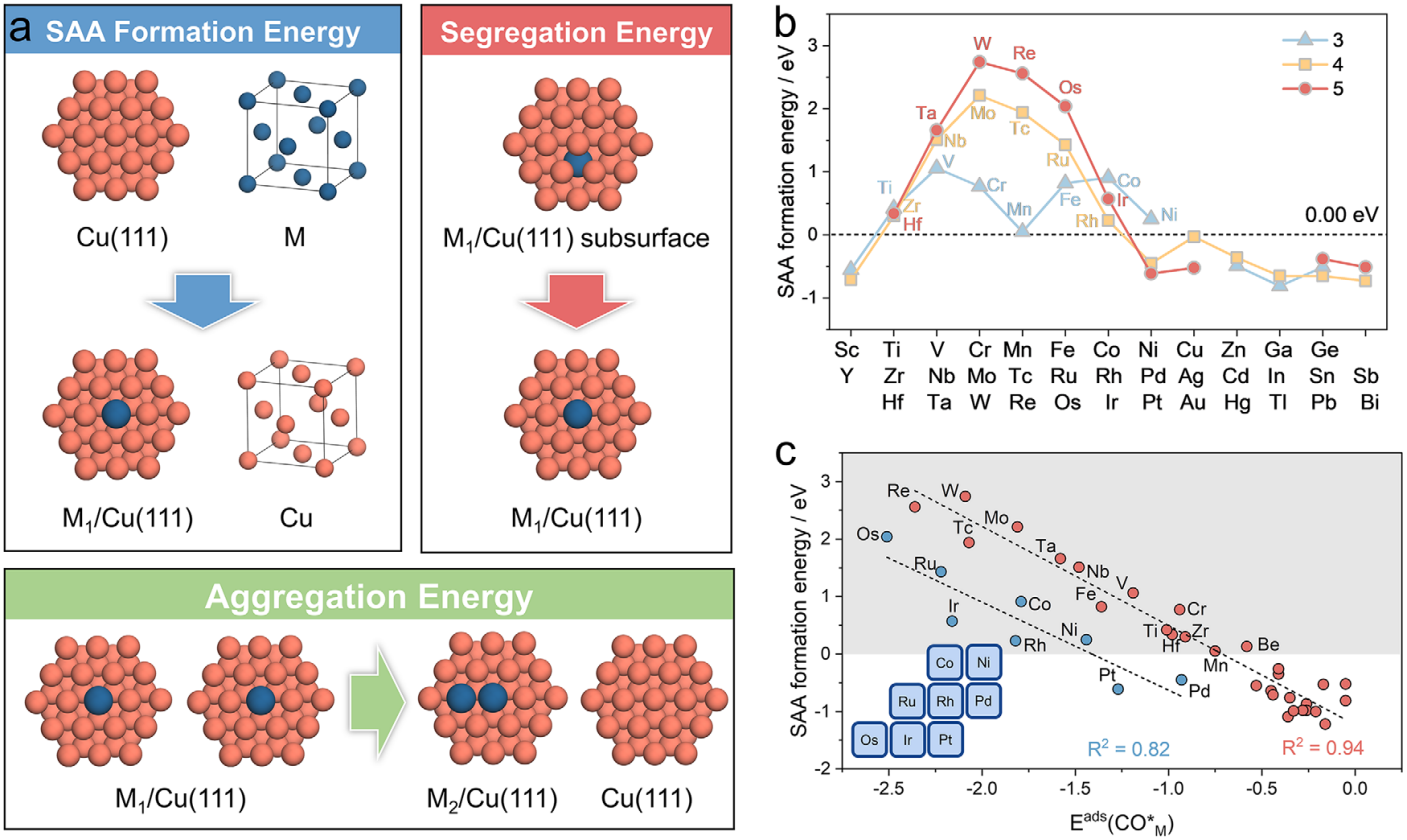
(a) Schematic diagram for the formation of SAAs and the dopants segregation, aggregation processes in Cu‐based SAAs. (b) Calculated formation energies of Cu‐based SAAs. The blue, yellow, and red dots respectively represent the elements of the third, fourth, and fifth periods in the periodic table. (c) Linear correlation between the thermodynamic formation energy of Cu‐based SAAs and the adsorption energies of CO* at dopant sites.

Prior to conducting the catalytic performance analysis, we initially carried out a theoretical evaluation of the stability of the Cu‐based SAAs. We first calculated the formation energies of isolated atom on the Cu(111) and Cu(100) surfaces (Figure S4), demonstrating that the Cu(111) surface is more energetically favorable for SAAs formation. This finding is consistent with the majority of high‐resolution transmission electron microscopy (HR‐TEM) observations [16, 17, 18, 19, 21, 22, 25, 26, 27, 28, 29]. Therefore, we focused our subsequent analysis on SAAs based on the Cu(111) surface. As illustrated in Figure 2a, we systematically evaluated the thermodynamic viability of isolated guest metal atom by quantifying three critical energetic parameters, namely formation energy (ΔE_form_), segregation energy (ΔE_seg_), and aggregation energy (ΔE_agg_) (Figure S5 and Table S2). Early transition metals (*i.e*., Ti, Zr, Hf, V, Nb, Ta, Cr, W, Tc, Re, Fe, Os) exhibit prohibitively high ΔE_form_ (> 0.0 eV) or ΔE_seg_ (> 0.0 eV) or low ΔE_agg_ (< 0.0 eV), indicating a strong thermodynamic driving force for dopant aggregation and inherent instability (Figure 2b and Figure S6). This finding aligns well with the lack of experimental reports for such Cu‐based SAAs (Figures 1b, c). In addition, several SAAs (M = Mn, Ru, Co, Rh, Ir, and Ni) although situated within the thermodynamically unstable region, are relatively closer to the stability boundary compared to the previously mentioned SAAs and have therefore been successfully synthesized. It should be noted that the present thermodynamic stability analysis does not account for the experimental success in synthesizing SAAs doped with Mo. Nevertheless, the current analytical framework remains valid and provides a reasonable explanation for the majority of existing experimental observations.

Furthermore, we investigated the thermal stability of Cu‐based SAAs doped with Zr, Ti, and Zn using molecular dynamics (MD) simulations. The results reveal that most Zr atoms migrate and achieve uniform distribution on the subsurface layers or coordinate with residual oxygen in deep layers (Figure S7). A similar behavior was observed in the MD simulation of Ti‐doped Cu‐based SAAs (Figure S8). These results align with thermodynamic calculations that the Zr_1_/Cu(111) or Ti_1_/Cu(111) SAA exhibits relatively high segregation and aggregation energies (Figure 2b and Figure S6), which are consistent with the experimental evidence of ZrO_x_‐Cu [30] or TiO_x_‐Cu [31] composite interfaces. Conversely, no atomic migration occurrs in Zn‐doped SAAs. All Zn atoms remain stably anchored on the outer surface (Figure S9) which is consistent with thermodynamic calculations (Figure 2b and Figure S6) and the widely documented alloy structures of CuZn catalysts [8, 11, 32]. We further conducted simulations of the surface oxidation process for SAAs and observed the formation of ZrO_2_ and TiO_2_ oxide aggregates (Figure S10a–c), respectively. These results provide further insights into experimentally observed ZrO_2_‐Cu and TiO_2_‐Cu interface structure [30, 31]. In contrast, Zn atoms in Zn_1_/Cu SAA maintain equivalent‐height coordination with Cu at the outer surface, exhibiting neither segregation nor detectable ZnO formation (Figure S10d). When the oxygen atom coverage on the Ti_1_/Cu SAA surface was decreased by 12.5%, Ti atoms predominantly reacted with O atoms to form the Ti_4_O_7_ or Ti_2_O_3_ surface polymers instead of TiO_2_ (as shown in Figure S10e and Figure S11). This finding aligns with the experimental observation of the Ti_4_O_7_/Ti_2_O_3_‐Cu interface formed through oxygen vacancy engineering [31]. These comprehensive MD simulations have validated the reliability of the thermodynamic calculations and provided a rational explanation for the observation that Cu‐based catalysts incorporating dopants such as Zr and Ti predominantly exist in the form of oxide interfaces, whereas Cu‐based alloys containing elements like Zn have been extensively documented.

As illustrated in Figure 2c, there exists a significant correlation between the stability of SAAs and their reactivity. As the adsorption strength of CO* at dopant sites becomes increasingly weak, the corresponding thermodynamic formation energy decreases accordingly. We further observed that elements such as Palladium (Pd) and Platinum (Pt) exhibited a stronger adsorption of CO* (blue data points in Figure 2c). Likewise, a linear relationship remaines between their thermodynamic formation energies and this adsorption behavior. In short, it highlights the necessity of computational screening to prioritize thermodynamically accessible SAAs.

Then the electrochemical stability of SAAs under CO_2_R conditions was systematically evaluated, with attention to the dissolution potential (U_diss_, Figure S12) and migration tendencies (E_for_^adatom^, Figure S13) of dopants. The calculated electrochemical stability trends are in good agreement with experimental observations or thermodynamic stability analysis. SAAs incorporating dopants with U_diss_ < 0 V (M = Ti, Zr, Hf, V, Nb, Ta, Cr, W, Fe, Re) have not been reported, as summarized in Table S1‐1. In contrast, SAAs with significantly positive U_diss_ values (M = Au, Ag, Pd, Pt) have been widely reported. Furthermore, SAAs susceptible to migration under the influence of O*, H*, and OH* species (M = Hf, Nb, Ta, W, Re, Os: E_for_^adatom^ < 0 eV) have not been experimentally reported. Moreover, the presence of CO* does not promote the formation of adatoms. Similar to the findings from thermodynamic stability calculations, the electrochemical dissolution potential does not fully account for the behavior of Mo, Sc, Mn, Co, and Ge. However, it still demonstrates strong explanatory power.

### 2.2 Experimental‐data‐driven Theoretical Simulations

**FIGURE 3:**
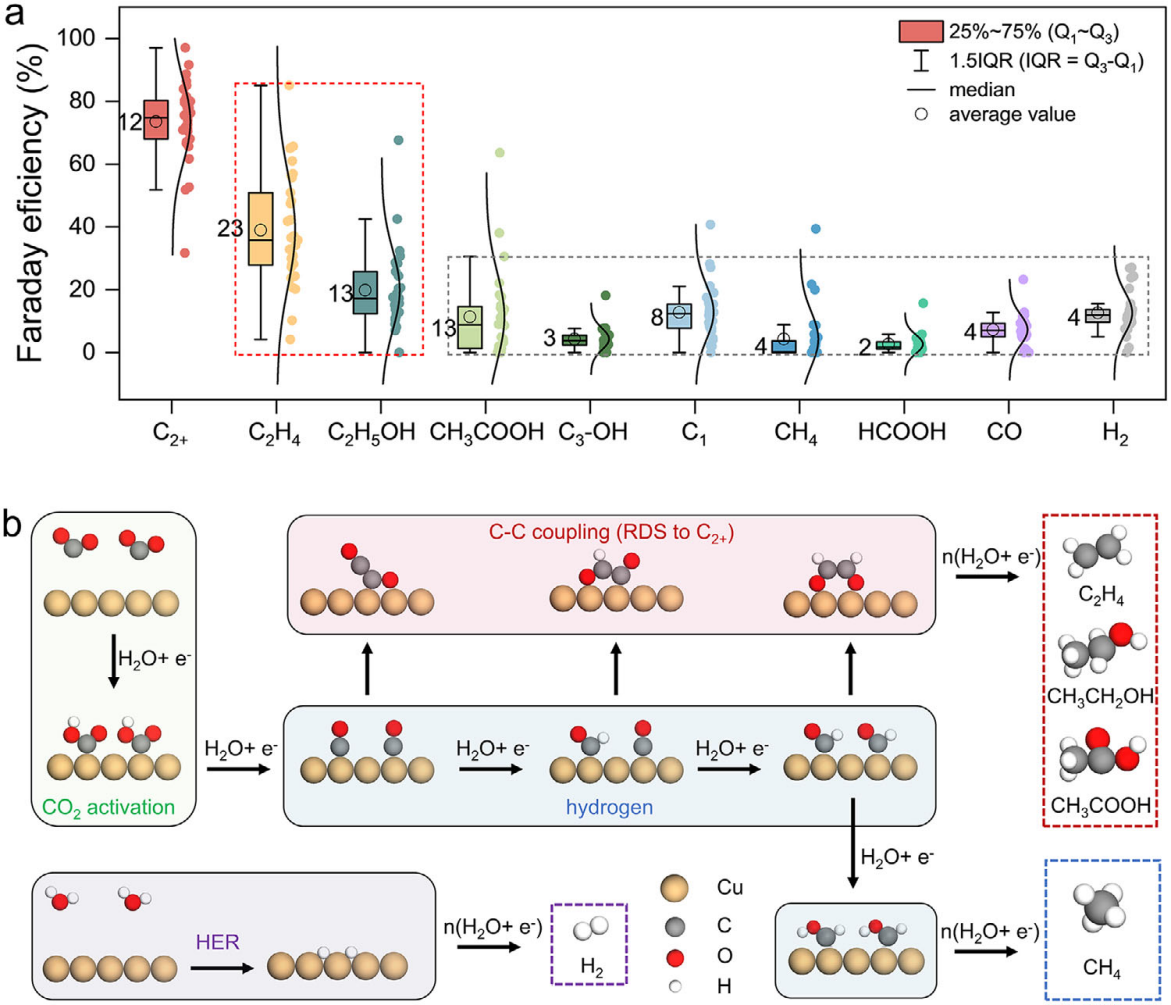
(a) Summary of experimental Faraday efficiency of CO_2_RR products over Cu‐based SAAs. The IQR value is presented adjacent to the box. (b) The mechanism diagram of CO_2_R to C_2+_ and C_1_ products on Cu considered in this work.

We utilized experimental literature data (the Faraday efficiencies of all CO_2_RR products on Cu‐based SAAs) extracted from *DigCat*’s ‘CO_2_ reduction’ module to train the Catalysis AI Agent. The detailed analysis process and corresponding research plan guidance from the AI Agent are presented in Video 1 and SI Part 1 (Supplementary Notes). Interestingly, the Catalysis AI Agent reported two key findings: (1) Cu‐based SAAs significantly enhance C_2+_ selectivity compared with pure Cu, which is statistically attributed to the promoted ethylene/ethanol formation and the suppressed methane formation. (2) The significant fluctuations of C_2+_ Faradaic efficiency across different Cu‐SAAs are mainly contributed from the different Faradaic efficiency of ethylene/ethanol depending on dopants. Building upon the Catalysis AI Agent's first finding, we constructed box plots to visualize the Faraday efficiencies of CO_2_R products (Figure 1b, Figure S1, and Figure S2), and H_2_ (Figure S2d) across Cu‐based SAAs. A further comparison of the CO_2_RR products distribution across all Cu‐based SAAs from experimental references is presented in Figure 3a and Figure S3a–d. The statistical analysis indicates that the primary differences in C_2+_ selectivity among various SAAs are predominantly contributed by ethylene and ethanol production (higher interquartile range value representing higher degree of data dispersion), whereas variations in the FEs of other products remain less distinguishable. Specifically, multiple reaction sites, such as Cu‐based polymetallic alloys [33, 34, 35], are essential for the formation of C_3_ products, whereas Cu‐based SAAs lack sufficient multiple sites to facilitate consecutive C‐C coupling steps. This viewpoint aligns well with current experimental findings, which shows that the Faraday efficiency of C_3_ products on SAA systems remains at a very low level (∼5%; Figures 3a, Figure S1 and Figure S3). These analyses confirm the Catalysis AI Agent's conclusions, where Cu‐based SAAs significantly promote ethylene and ethanol formation while suppressing methane production. Other C_2+_ compounds, C_1_ products, and H_2_ exhibit comparable Faraday efficiencies to that of Cu, which is not the origin of diversity of C_2+_ Faraday efficiencies among Cu‐based SAAs. As outlined in Figure 3b, we mapped key reaction pathways from CO_2_ to C_2+_ and C_1_ products. This includes: (i) CO_2_ activation to CO, (ii) deep hydrogenation of CO (C_1_ pathway), (iii) C‐C coupling (C_2_ pathway), and (iv) the competing hydrogen evolution reaction (HER). Our computational scheme prioritizes C‐C coupling processes (the rate‐determining step for C_2+_ generation [36, 37, 38, 39, 40]) and CO deep hydrogenation (critical for CH_4_ formation), explicitly excluding HER. Based on the guidance from AI, we formulated our computational strategy, only focusing on activity for C_2+_ products and methane formation, guaranteeing efficient exploration of structure‐selectivity relationships.

### 2.3 Diverse C‐C Coupling Mechanisms in Cu‐based SAAs

We first calculated the thermodynamic reaction energies for CHO* hydrogenation to CHOH* on Cu‐based SAAs (Figure S14) since CHOH* was previously identified as the key species for CH_4_ formation [38, 39, 41]. Our calculations reveal that the CHOH* formation energies at both dopant (M) and first‐nearest‐neighbor copper (Cu_1_) sites on most of Cu‐based SAAs exceed those on pure Cu surfaces, with Pt_1_/Cu being the sole exception. These results align with the observed high methane selectivity on pure Cu and Pt_1_/Cu surfaces. The concentrated distributions of CH_4_ experimental Faraday efficiency, as well as thermodynamic reaction energies for CHO* hydrogenation to CHOH*, indicate that fluctuant C_2+_ products selectivity across Cu‐based SAAs originates primarily from the diversity of C‐C coupling elementary steps rather than CH_4_ formation pathway.

**FIGURE 4:**
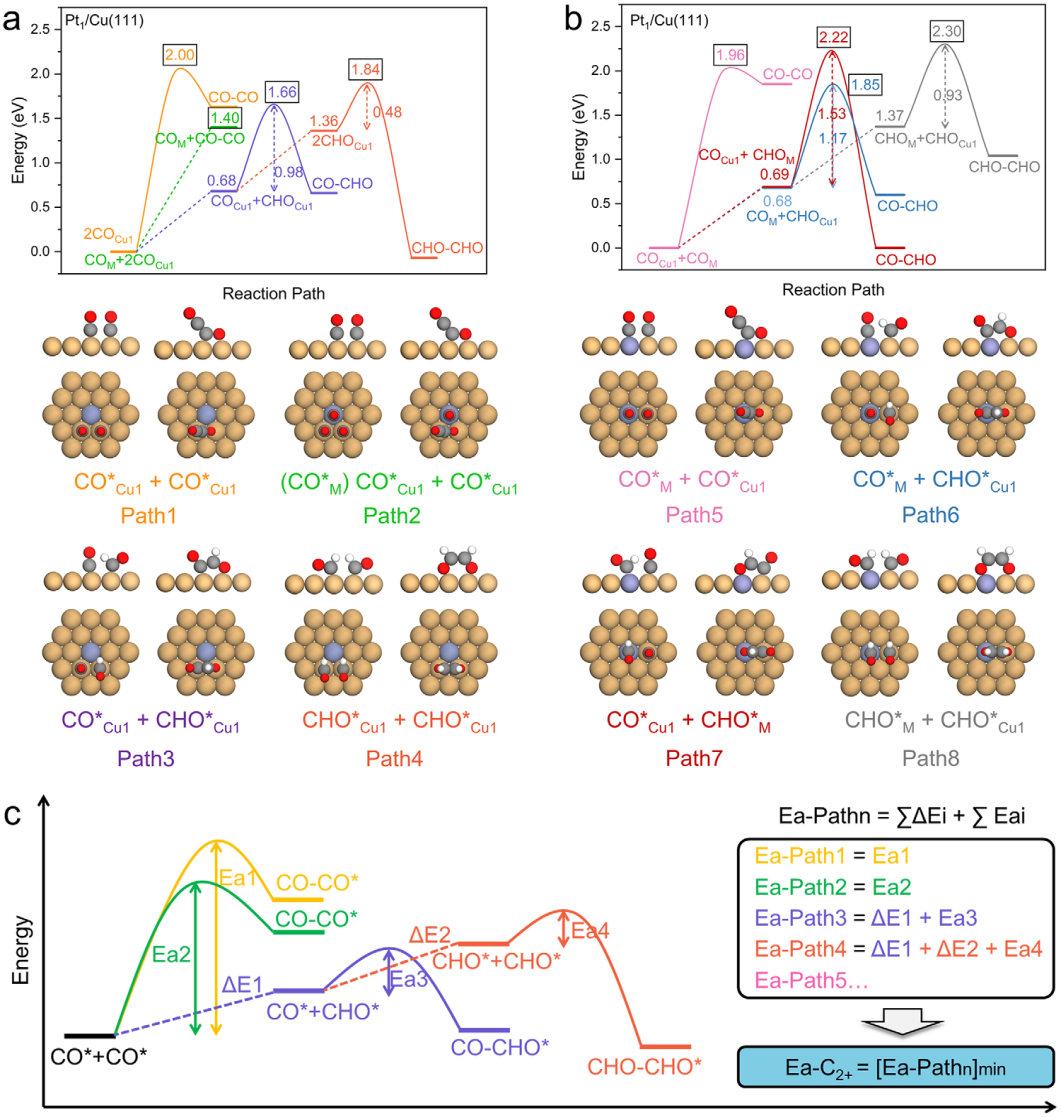
Reaction energy profiles for eight distinct C‐C coupling pathways on the Pt_1_/Cu(111) surface. (a) Uninvolved with M site: CO_Cu1_+CO_Cu1_, (CO_M_) CO_Cu1_+CO_Cu1_, CO_Cu1_+CHO_Cu1_, CHO_Cu1_+CHO_Cu1_. (b) Involved with M site: CO_Cu1_+CO_M_, CO_Cu1_+CHO_M_, CO_M_+CHO_Cu1_, CHO_Cu1_+CHO_M_. The bottom of the figure presents the configuration of initial and final states for different C‐C coupling pathways on SAAs. Atomic color code: Cu‐yellow, Pt‐purple, C‐gray, O‐red, H‐white. (c) The definition and calculation methodology of Ea‐C_2+_ by considering all the C‐C coupling pathways.

We then systematically evaluated eight distinct C‐C coupling pathways mediated by *CO or *CHO precursors (Figures 4a,b). These pathways are denoted as Path1 to Path8: CO*+CO*: CO*_M_+CO*_Cu1_(Path5), CO*_Cu1_+CO*_Cu1_(Path1), (CO*_M_)‐CO*_Cu1_+CO*_Cu1_(Path2); CO*+CHO*: CO*_M_+CHO*_Cu1_(Path6), CO*_Cu1_+CHO*_M_(Path7), CO*_Cu1_+CHO*_Cu1_(Path3); CHO*+CHO*:CHO*_M_+CHO*_Cu1_(Path8), CHO*_Cu1_+CHO*_Cu1_(Path4), where *CO_M_ and *CO_Cu1_ denote adsorption at dopant (M) and Cu_1_ sites, respectively. For instance, CO*_M_+CO*_Cu1_ (Path5) signifies that the C‐C coupling process occurs *via* the CO dimerization mechanism (the pink path in Figure 4b), forming the COCO* intermediate, with CO* residing at the M and Cu_1_ sites, respectively. CO*_Cu1_+CHO*_M_ (Path7) denotes that the C‐C coupling process proceeds through the CO + CHO coupling mechanism (red path in Figure 4b). In this case, CO* at the M site is initially hydrogenated to form CHO*, which subsequently couples with CO* at the Cu_1_ site to generate the COCHO* intermediate. CHO*_M_+CHO*_Cu1_ (Path8) indicates that the C‐C coupling process occurs *via* the CHO + CHO coupling mechanism (the grey path in Figure 4b). Herein, CO* located at the M and Cu_1_ sites is first hydrogenated to form CHO*, followed by CHO* dimerization to produce the CHOCHO* intermediate. The (CO*_M_)‐CO*_Cu1_+CO*_Cu1_ (Path2) also represents CO dimerization at Cu_1_ site, forming a COCO intermediate (the green path in Figure 4a). Although all reacting CO species reside at Cu_1_ sites, this mechanism involves pre‐adsorption of CO at the M site which attributes to a consequence of dopants' strong CO* adsorption affinity. However, the M‐adsorbed CO* acts as a spectator during the dimerization event itself. The dominant pathway to form C_2+_ products, as well as theoretical reactivity to C_2+_ products, are determined by the minimal activation energy to generate C_2+_ precursor among competing C‐C coupling pathways (Ea‐C_2+_, Figure 4c):

(1) $$Ea-C_{2+}={\left(Ea-Path_{n}\right)}_{\min}$$

where *Ea* − *Path_n_* incorporates both the reaction heat of *CHO formation and the kinetic barrier of C‐C coupling. For Pt_1_/Cu(111) SAA, Path2 exhibits the lowest Ea‐C_2+_ (1.40 eV), attributed to its unique spectator mechanism, where *CO remains strongly adsorbed at the Pt dopant site while adjacent Cu_1_ sites facilitate energetically favorable *CO dimerization (Figures 4a,b). Recently, Zhao et al. developed the embedded correlated wavefunction (ECW) theory, and discovered that COH* can coexist with CHO* as potential CO* hydrogenation intermediates on the Cu(111) surface [42]. This insight facilitated the identification of several novel C‐C coupling pathways [43]. Building on this breakthrough, we conducted a detailed analysis of the thermodynamic formation energies of COH* and CHO* at both M and Cu_1_ sites in Cu‐based SAA systems (Figure S15). Our investigation identified several SAAs (Co, Nb, Mo, Tc, Ru, Re, Mn, Fe, Os) where COH* serves as the predominant intermediate. Furthermore, we calculated the energy barriers for COH*‐involved C‐C coupling pathways (Figure S18g, S19f, S20d, S20f, and S20h). It determines that the preferred C‐C coupling mechanism in Cu‐based SAAs with Tc and Re as dopants is the CO_Cu1_‐COH_M_ pathway (Figures S19f and 20f). However, CO‐COH undergoes dissociation on the surface of M‐doped SAAs (M = Nb, Mo, Mn, Fe). Comprehensive calculations across various Cu‐based SAAs (Figures S16–S24, Tables S3–S6) reveal C‐C coupling dominant pathway over most of Cu‐based SAAs demonstrate lower Ea‐C_2+_ values compared to pristine Cu(111). This general reduction of Ea‐C_2+_ accounts for the higher experimental C_2+_ Faraday efficiency, confirming the intrinsic advantage of Cu‐based SAA compared to pure Cu catalyst.

To evaluate the influence of crystal plane orientation on C‐C coupling activity in SAAs, we systematically examined the energy barriers of several representative SAAs on Cu(100) (Figure S25). First, the C‐C coupling energy barrier on the pristine Cu(100) surface is lower than that on the Cu(111) surface, which aligns well with experimental observations. For dopants following the CO‐CHO coupling mechanism (M = Y, Ag, Al, Sb, Bi), the effect of crystal plane orientation on the C‐C coupling is little. Moreover, for SAAs operating via the CO‐CO coupling path (M = Pt, Co), the Cu(111) surface exhibits more favorable energetics. Although our current calculations suggest that the overall conclusions are not altered by crystal plane orientation, we acknowledge the potential influence of coordination environment variations on adsorption behaviors and reaction pathways [7, 21, 44].

### 2.4 Energy Scaling Relationship Construction Guided by the Catalysis AI Agent

**FIGURE 5:**
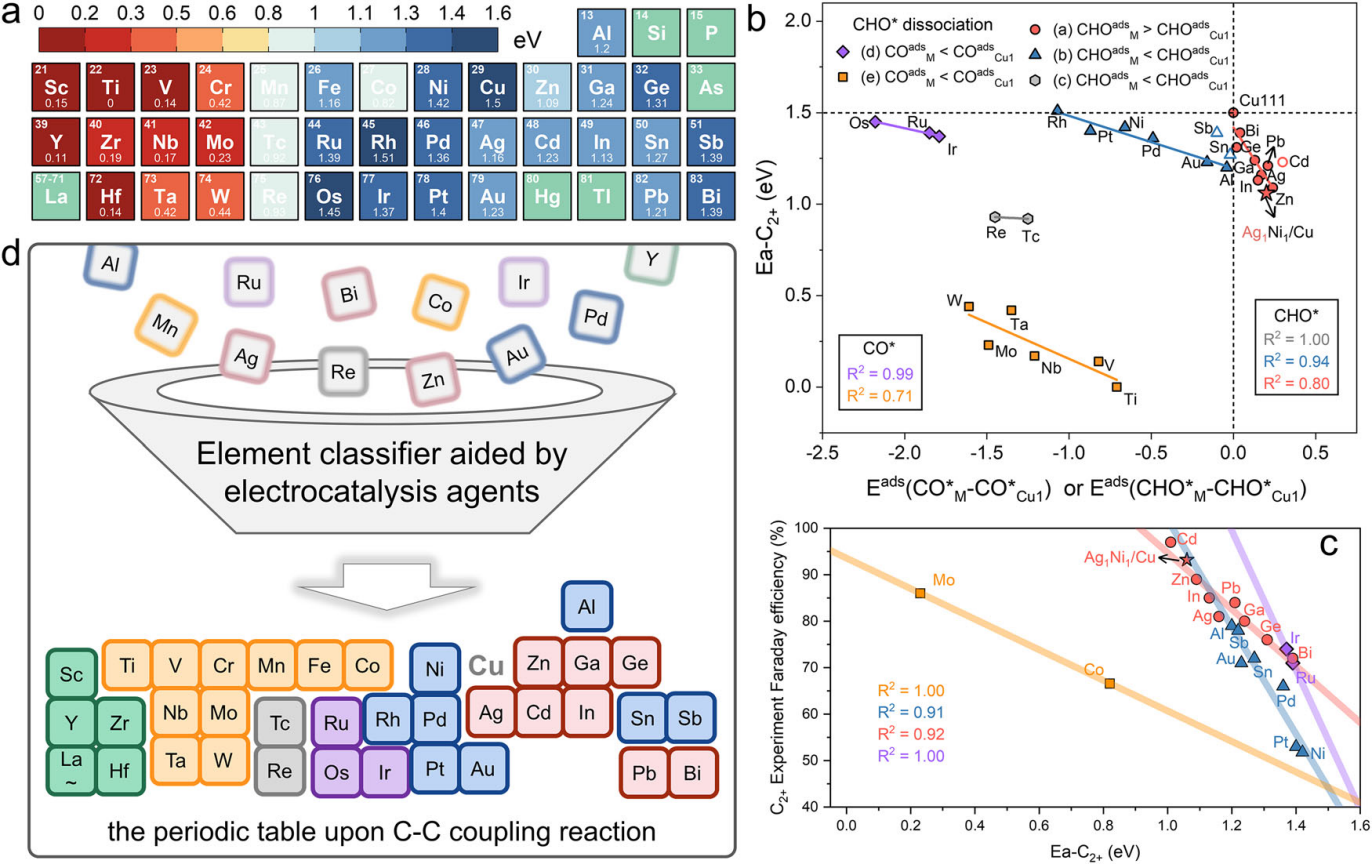
Theoretical selectivity trend of C_2+_ products on Cu‐based SAAs. (a) The theoretical reactivity heatmap of C_2+_ products (Ea‐C_2+_) on Cu‐based SAAs. Red squares indicate a low barrier, while blue squares indicate a high barrier. (b) Energy descriptors for Ea‐C_2+_ of Cu‐based SAAs and their corresponding linear relationships. (c) The linear correlation between the experimental Faraday efficiency and Ea‐C_2+_ of Cu‐based SAAs. (d) The classification of Cu‐based SAAs in the periodic table upon foreign elements with guidance from AI agent, according to the linear relationships in (b,c).

However, there is no significant negative correlation between the theoretical barrier for C‐C coupling (Ea‐C_2+_) and the experimental Faraday efficiency of C_2+_ products among all Cu‐based SAAs (Figure S26). Therefore, we subsequently provided computational and experimental data to the Catalysis AI Agent (see Video 2 and Part 1.5 Supplementary Notes). The Catalysis AI Agent conducted a comprehensive analysis of computational and experimental performance data, and strikingly, quickly suggested that a classification analysis of the guest elements is essential to establish a more accurate and meaningful structure‐selectivity relationship. To further investigate the varying trends in the activity for C‐C coupling dominant pathways over Cu‐based SAAs (Figure 5a), we employed retrieval‐augmented generation technology to integrate domain knowledge from established catalytic descriptors [45] with AI‐driven insights (see Video 3, 4 and Part 1.5 Supplementary Notes). This approach yielded several promising research directions. A strong correlation exists between $E_{CHO*}^{ads}\left(M-Cu_{1}\right)$ and Ea‐C_2+_. A weak correlation is observed between $E_{CO*}^{ads}\left(M-Cu_{1}\right)$ and Ea‐C_2+_. Cu‐based SAAs facilitate CHO* dissociation at M‐sites exhibit lower Ea‐C_2+_ values. Cu‐based SAAs where CO* desorption occurs preferentially at M‐sites demonstrate higher Ea‐C_2+_. The magnitude of Ea‐C_2+_ depends on both the coupling mechanism and reaction sites. Faraday efficiency for C_2+_ products depend on the dominant C‐C coupling mechanism. Therefore, we established four key classification metrics for Cu‐based SAAs: (1) the propensity for CHO* dissociation at M‐sites; (2) the tendency for CO* desorption at M‐sites; (3) the dominant C‐C coupling mechanism governing reaction pathways; and (4) adsorption energy differentials ($E_{CO*}^{ads}\left(M-Cu_{1}\right)$ or $E_{CHO*}^{ads}\left(M-Cu_{1}\right)$). This multidimensional framework enables systematic categorization of Cu‐based SAA catalysts.

To elucidate C‐C coupling mechanisms across Cu‐based SAAs and rationalize the observed differentiation of energy barriers, we introduced energy descriptors defined as the differential adsorption energies of C‐C coupling precursors between dopant (M) and Cu_1_ sites (Table S7):

(2) $$E_{CO*}^{ads}\left(M-Cu_{1}\right)\;or\;E_{CHO*}^{ads}\left(M-Cu_{1}\right)$$

Firstly, we categorized the reaction pathways into two distinct groups based on the adsorption behavior of CHO at the M site, where CHO remains adsorbed stably are characterized using the descriptor $E_{CHO*}^{ads}\left(M-Cu_{1}\right)$ while pathways involving CHO* dissociation employ the descriptor $E_{CO*}^{ads}\left(M-Cu_{1}\right)$. It reveals five distinct regions (part1: a‐c; part2: d‐e) (Figure 5b) and five distinct linear relationships. Ea‐C_2+_ in **Regions a** determined by $E_{CHO*}^{ads}\left(M-Cu_{1}\right)$, corresponds to an asymmetric coupling mechanism (CO‐CHO). Ea‐C_2+_ values in **Regions b** correlate with $E_{CHO*}^{ads}\left(M-Cu_{1}\right)$, corresponds to an asymmetric coupling mechanism (CO‐CHO) or a spectator mechanism induced by strong CO* adsorption originating at M sites. Ea‐C_2+_ in **Regions c** are linearly related with $E_{CHO*}^{ads}\left(M-Cu_{1}\right)$, corresponding to an asymmetric coupling mechanism (CO‐COH). Ea‐C_2+_ in **Regions d** determined by $E_{CO*}^{ads}\left(M-Cu_{1}\right)$, correspond to CO‐CHO with CHO* at the Cu_1_ site or CO‐CO mechanism. Ea‐C_2+_ in **Region e** correlated with $E_{CO*}^{ads}\left(M-Cu_{1}\right)$, correspond to the CO dimerization mechanism arising from dopant induced *CHO dissociation adsorption. Notably, high‐magnetism dopants (*i.e*., Cr, Mn, Fe, Co) in **Region e** exhibit significant deviation from linear scaling due to strong spin‐polarization effects. Figure 5c illustrates several groups of strong linear correlation between the theoretical Ea‐C_2+_ values and the experimental C_2+_ Faraday efficiency, which are in good agreement with the classification based on energy descriptors and thereby validates the accuracy of our simulation of the C‐C coupling mechanism on Cu‐based SAAs. Prior to the publication of this work, the Cd_1_/Cu(111) SAA [28] was reported to exhibit a Faraday efficiency of 98% for C_2+_ products, thereby corroborating our theoretical predictions. Overall, the asymmetric precursor adsorption at M‐Cu_1_ sites governs both C‐C coupling mechanisms (Figure S27) and Ea‐C_2+_ values, serving as an elemental classifier that organizes dopants into a periodic “Tetris‐like” distribution (Figure 5d). Crucially, AI agents have significantly contributed to the discovery of this classification strategy. Our doping research on the Cu (111) surface offers feasible strategies and theoretical insights for enhancing the low selectivity of the original Cu (111) surface toward C_2+_ products. The high consistency between Ea‐C_2+_ and experimental Faraday efficiency also confirms the reasonability of Cu(111)‐based SAAs as representative surface models for Cu‐based SAAs in experiments.

We also employed machine learning approaches (Figure S28) to predict the experimental Faraday efficiency of C_2+_ products on SAAs. The training outcomes indicate that several conventional machine learning models fail to achieve accurate predictions (R^2^< 0.7, MAE > 5%). Using the random forest model, we further assessed feature importance (Figure S28e). Although certain key features were identified, critical factors such as reaction mechanisms were underrepresented. This underscores a distinct advantage of AI agents in data analysis—namely, their ability to preserve the intrinsic chemical significance of features without discarding incomplete or partially available feature values. Nevertheless, when handling large‐scale datasets, the integration of machine learning with AI methodologies can substantially enhance predictive performance.

To further investigate the theoretical activity of C_2+_ products, a group of Cu‐based SAAs incorporating Ag, Bi, Cd, In, Pb and Zn were systematically examined using microkinetics modeling (MKM) to assess the validity of predicting selectivity trends based solely on the barrier of C‐C coupling step. The MKM results (Figure S29) reveal a selectivity trend for ethylene that aligns well with the experimental C_2+_ Faraday efficiency. Furthermore, recent studies by Che et al., [40]., utilizing MKM and degree of rate control (DRC) analysis, have corroborated that the formation pathways of ethylene and ethanol are predominantly governed by the barrier of the C‐C coupling step (CO‐CHO).

It is worth noting that the high‐throughput screening study in this work primarily focuses on the C‐C coupling process responsible for the formation of C_2+_ products, while appropriately neglecting the competing C_1_ hydrogenation and HER pathways that lead to C_1_ products and H_2_. This simplification is justified by both experimental observations and the established understanding that the C‐C coupling step acts as the rate‐determining step (RDS) for C_2+_ product formation [36, 37, 38, 39, 40]. As illustrated in Figure S30, across a specific SAA group as same as Figure 5c, the Faradaic efficiency for C_2+_ products exhibits considerable variation among different SAAs, whereas the efficiencies for C_1_ products and H_2_ remain relatively concentrated. This dispersion in performance further supports the rationale for concentrating on C‐C coupling step. Similar theoretical simplified approaches have been adopted in recent studies [25, 29, 46, 47] on Cu‐based SAAs. Nevertheless, it should be emphasized that, the most accurate selectivity trends toward C_2+_ products must be evaluated by taking into account both the dominant and side reaction pathways in a comprehensive manner.

Accordingly, in the present study, we have not developed a sophisticated explicit electrochemical microenvironment model (incorporating factors such as cations, solvents, hydrogen‐bonding networks, and other double‐layer effects) to investigate the formation of C_1_ and H_2_ products, which are closely associated with the proton transfer process. Notably, emerging evidence has demonstrated the promotional effects of cationic species in both CO_2_ activation and subsequent C‐C coupling processes [48, 49, 50, 51]. Nevertheless, the variance in C_2+_ products selectivity across different catalysts under identical cationic conditions remains predominantly governed by the intrinsic structural characteristics of the catalytic materials rather than explicit cationic effect [52, 53, 54]. We further investigated the influence of electric potential and electrolyte environment on the C‐C coupling process. The computational results indicate that electrochemical factors may alter the absolute reaction energies, however they do not affect the identification of the dominant C‐C coupling pathway on SAAs or its relative trend of energy (Figure S31). However, we still remind readers of the critical importance of explicitly modeling cationic, solvent environments, and electrode potential when probing the detailed C‐C bond formation mechanisms for a single catalyst model.

Our theoretical framework also demonstrates broader applicability to DSAAs. For instance, Jia et al. recently reported an Ag_1_Ni_1_/Cu DSAA catalyst [55] that notably demonstrated superior C_2+_ selectivity compared to Ag_1_/Cu or Ni_1_/Cu SAAs (Table S1). By calculating energy descriptors and Ea‐C_2+_ for the Ag_1_Ni_1_/Cu model, we confirmed its alignment with our prediction from linear relationship (Figures 5b, c). To elucidate the enhanced C_2+_ selectivity in Ag_1_Ni_1_/Cu DSAAs relative to monometallic SAA counterparts, we analyzed their electronic structures via charge density difference and Bader charge calculations. Figure S32a,b reveal preferential charge redistribution localized at the first coordination shell of Cu atoms adjacent to Ni dopants, with minimal perturbation near Ag sites. Bader charge analysis further quantifies this Ni‐induced electronic modulation on neighboring Cu atoms (Figure S32c,d). These findings collectively demonstrate that electronic fine‐tuning of Cu sites through SAAs governs reactive intermediate adsorption energetics, directly linking microscopic electronic perturbations to macroscopic catalytic performance adjustment. Building upon this finding, we introduced a secondary dopant (M_2_: Ni, Pt, Pd, Rh, Au, Al, Sb, Sn) into M_1_/Cu SAAs (M_1_: Ag, Zn, In, Bi, Ga, Ge, Pb, Cd), constructing DSAAs denoted M_1_M_2_/Cu. The calculated energy descriptors for these DSAAs are summarized in Table S8. As indicated by the red linear fit in Figure 5b, higher energy descriptor values correspond to lower activation energies for the dominant C‐C coupling pathway (Ea‐C_2+_), signifying greater potential for C_2+_ forming products. Comparative analysis between these DSAAs and their parent M_1_/Cu SAAs identify candidate configurations that may enhance C_2+_ selectivity (Table S8). M_2_ = Ni with M_1_ = Ag, Zn, In, Bi, Ga, Ge, Pb; M_2_ = Pt with M_1_ = Zn; M_2_ = Rh with M_1_ = Ag, Zn; M_2_ = Al with M_1_ = Ag, Bi; M_2_ = Sb with M_1_ = Pb. Notably, the Zn_1_Ni_1_/Cu and Zn_1_Rh_1_/Cu DSAAs exhibit the highest potential for CO_2_RR to C_2+_ products.

### 2.5 Structural Descriptor of Cu‐based SAAs for C‐C Coupling

We further investigated the electronic structure of dopant metals to clarify the adsorption behaviors of precursors in C‐C coupling. As illustrated in Figure S33a, CHO* exhibits three distinct adsorption configurations at M sites, namely side‐on, end‐on, and dissociative configurations. These behaviors can be rationalized through the 10‐electron rule [56], which posits optimal stability when the sum of valence electrons from the metal dopant (n1) and interacting adsorbate orbitals (n2) equals ten. The systematic analysis (Figure S33b and Table S9) reveals periodicity‐governed trends. Metals located to the right of Ru‐group in the periodic table (n1>8) prefer end‐on CHO* adsorption (*e.g*., Rh, Pd, Ag), maximizing orbital overlap while limiting electron contribution to satisfy n1 + n2 close to 10. Metals positioned to the left of Ru‐group on the periodic table (n1<8), with dominant side‐on CHO* adsorption, enable greater electron donation to achieve electronic saturation. For example, early transition metals (*e.g*., Sc, Y, Zr, Hf, n1 = 3 or 4) stabilize side‐on CHO* configurations through enhanced π‐backdonation. As for the Ru‐group metals (n1 = 8), they possess pronounced CO* (n2 = 2) affinity with concomitant CHO* dissociation (*i.e*., Fe, Ru, Os), as CO's dual electron contribution optimally satisfies the 10‐electron criterion. Notably, co‐adsorption scenarios amplify these effects (CHO* dissociation) for SAAs with n1 = 5∼6 (*i.e*., Mo, V, Cr, Nb, Tc, Ta, W, and Re). This electronic structure‐adsorption correlation provides a universal framework for predicting precursor stabilization in C‐C coupling reactions, enabling rational Cu‐based SAA catalyst design.

While Berger et al., [57]. recently demonstrated that co‐adsorption of intermediates at dopant sites in early transition metal‐doped SAAs enhances C‐C coupling reactions via the 10‐electron rule, our computational investigations reveal critical nuances. As shown in Figure S34, dual *CO adsorption at dopant site does occur in Ti, Zr, Nb, Ta, and Hf doped systems. However, the associated CO‐CO coupling energy barriers remain prohibitively high (Ea> 2.0 eV). Therefore, we did not further discuss the phenomenon of reaction promotion caused by species co‐adsorption at a single point.

**FIGURE 6:**
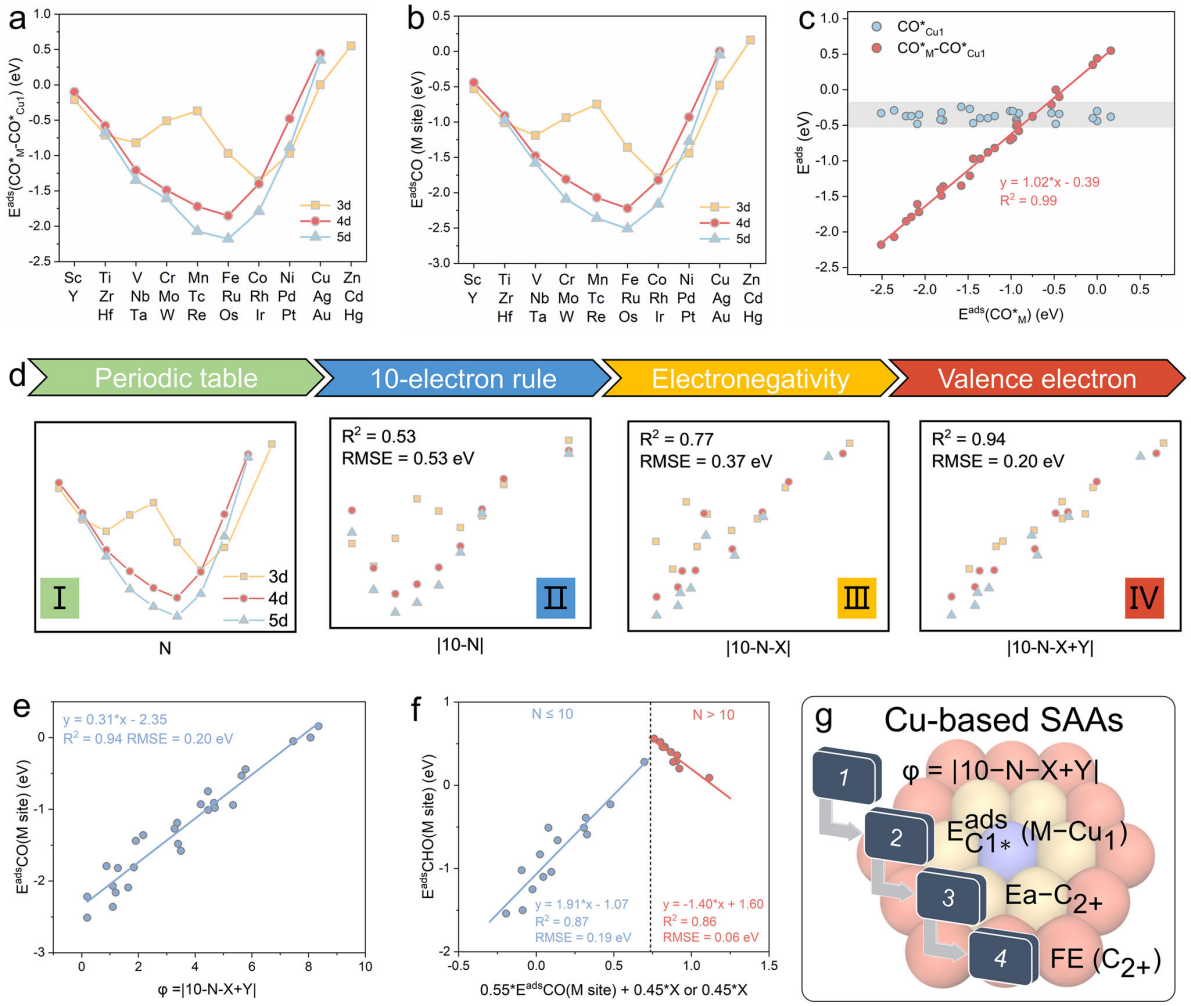
Structural descriptor for the adsorption strength of the precursor in C‐C coupling reactions on Cu‐based SAAs. (a) The difference in CO* adsorption energies between the M site and the Cu_1_ site of the SAAs. (b) Adsorption energies of CO* at the M site on SAAs. (c) Correlation of CO* adsorption energies across M and Cu_1_ sites. (d) The process of identifying structural descriptors. (e) Structural descriptor of CO* adsorption energies at M site of Cu‐based SAAs. (f) The linear correlation between the adsorption energies of CO* and CHO*. (g) Schematic illustration of the design principles for enhancing CO_2_RR toward C_2+_ products in Cu‐based SAAs.

We further found that the variations in energy descriptors among different Cu‐based SAAs are primarily attributed to the adsorption strength at the M site, as evidenced by the strong correlation between the energy descriptor and the adsorption energies of precursors in C‐C coupling reaction at dopant sites. Periodic trends for $E_{CO*}^{ads}\left(M-Cu_{1}\right)$ and $E_{CO*}^{ads}\left(M\right)$ also exhibit near‐identical profiles (Figures 6a,b), and a linear regression with a R^2^ of 0.99 exist between them (Figure 6c). Little variation in $E_{CO*}^{ads}\left(Cu_{1}\right)$ across Cu‐based SAAs (Figure S35a) further supports this relationship. The analogous behavior for CHO* adsorption is observed (Figure S35b–e), excluding rare earth elements and Zr/Hf due to their unique orbital hybridization. To be noted, calculated adsorption strength trends for CO* and CHO* (Figure 6b and Figure S35c) align quantitatively with 10‐electron rule predictions. CHO* in end‐on configuration maximize interaction with n1 = 9 metals (Rh and Ir). CHO* in side‐on configuration favored by n1 = 5 metals (Nb and Ta), and CO* dominance for n1 = 8 metals (Ru and Os). On this basis, we further proposed the recognition procedure for interpretable structural descriptors to predict the adsorption energies of CO* at M site (Figure 6d), which is grounded in the periodic table of elements and physicochemical principles. Firstly, we found that the adsorption strength of CO* at dopant sites exhibits distinct periodic trends, where there is a W‐shaped pattern in the third period and V‐shaped trends across the fourth and fifth periods. These observations align with the 10‐electron rule, correlating with the element's group number (N) or valence electron count (n). To rationalize this symmetry, we established a linearization framework by designating the element with the strongest CO* adsorption as the central axis of symmetry. This transformation yielded the descriptor |10−N|, where substantial deviations existed for elements in same group and different period (R^2^ = 0.53, RMSE = 0.53 eV), attributable to inherent periodic variations. We further quantified the electron transfer and discovered that the number of electron transfers at M site or Cu_1_ site is closely correlated with the electronegativity of the dopant (Figure S36). Therefore, we incorporated elemental electronegativity (X) into the descriptor, generating the modified parameter |10−N−X|. As recently reported by Ren [58] and Chang [59] et al., the influence that alloy catalysts have on small molecules's adsorption energies can primarily be attributed to electron transfer, which arises from the electronegativity difference between the introduced dopant and the original host metal. This adjustment significantly enhanced linear correlation (R^2^ = 0.77, RMSE = 0.37 eV). Residual outliers were subsequently traced to elements with electron configurations approaching half‐filled or fully‐filled states in their valence shells. Building upon this observation, we introduced an electronic state‐dependent correction term (Y) accounting for these anomalies. The structural descriptor φ for guest metal elements in periodic table columns, defined as:

(3) φ=|10−N−X+Y|

where N, X, and n represent the column number, Pauling electronegativity, and valence d‐electron count of element M, respectively. The term Y (= 8‐n) applies exclusively to elements with half‐filled (Cr, Mn, Fe) or fully filled d‐orbitals (Pd, Pt) (Figure S37), while Y = 0 for others. Moreover, when N>10, φ=|10‐X+Y|. This structural descriptor φ achieves remarkable accuracy in predicting $E_{CO*}^{ads}\left(M\right)$ (R^2^ = 0.94, RMSE = 0.20 eV, Figure 6e) and reveals a linear correlation between $E_{CHO*}^{ads}\left(M\right)$ and $E_{CO*}^{ads}\left(M\right)$ (R^2^ >0.85, Figure 6f).

We also verified the rationality of the current descriptor φ through machine learning methods. First, we summarized the characteristic parameters related to the dopants and the first nearest‐neighbor Cu atoms (Table S10) and conducted a correlation analysis (Figure S38). It revealed strong correlations between the column number of dopants in periodic table (N) and multiple features, and between the average distance of M‐Cu1 (d_M_) and the average distance of Cu1‐Cu1 (d_Cu1_). After eliminating redundant features, we selected dopant electronegativity (X), first ionization energy (E_fi_), atomic radius (R), the column number of dopants in periodic table (N), and the average distance of M‐Cu1 (d_M_) as feature parameters for random forest regression modeling using E^ads^(CO*_M_) as the target property. Feature importance analysis (Figure S39) identified X and N as the most critical predictors for E^ads^(CO*_M_), consistent with the key parameters in our proposed structural descriptor (φ = |10‐N‐X+Y|).

Eventually, we proposed a universal design principle of Cu‐based SAA catalysts for CO_2_R toward C_2+_ products (Figure 6g). First, we established a simple but effective structural descriptor (φ) for Cu‐based SAAs based on the intrinsic properties of the dopants. Second, linear correlations were identified between φ and the adsorption energies of C_1_ precursors in C‐C coupling steps, enabling direct prediction of CO* and CHO* adsorption energies. Third, we established the correlation between C_1_ adsorption strength and Ea‐C_2+_, a theoretical activity index for C_2+_ products. Fourth, experimental validation confirmed the consistency between Ea‐C_2+_ and the Faraday efficiency of C_2+_ products, demonstrating its effectiveness in identifying selectivity trends. In a word, this universal design principle enables quick direct prediction of experimental catalytic performance based solely on intrinsic properties of guest metal of Cu‐based SAAs.

### 2.6 Application of Universal Design Principle of Cu‐based SAAs

**FIGURE 7:**
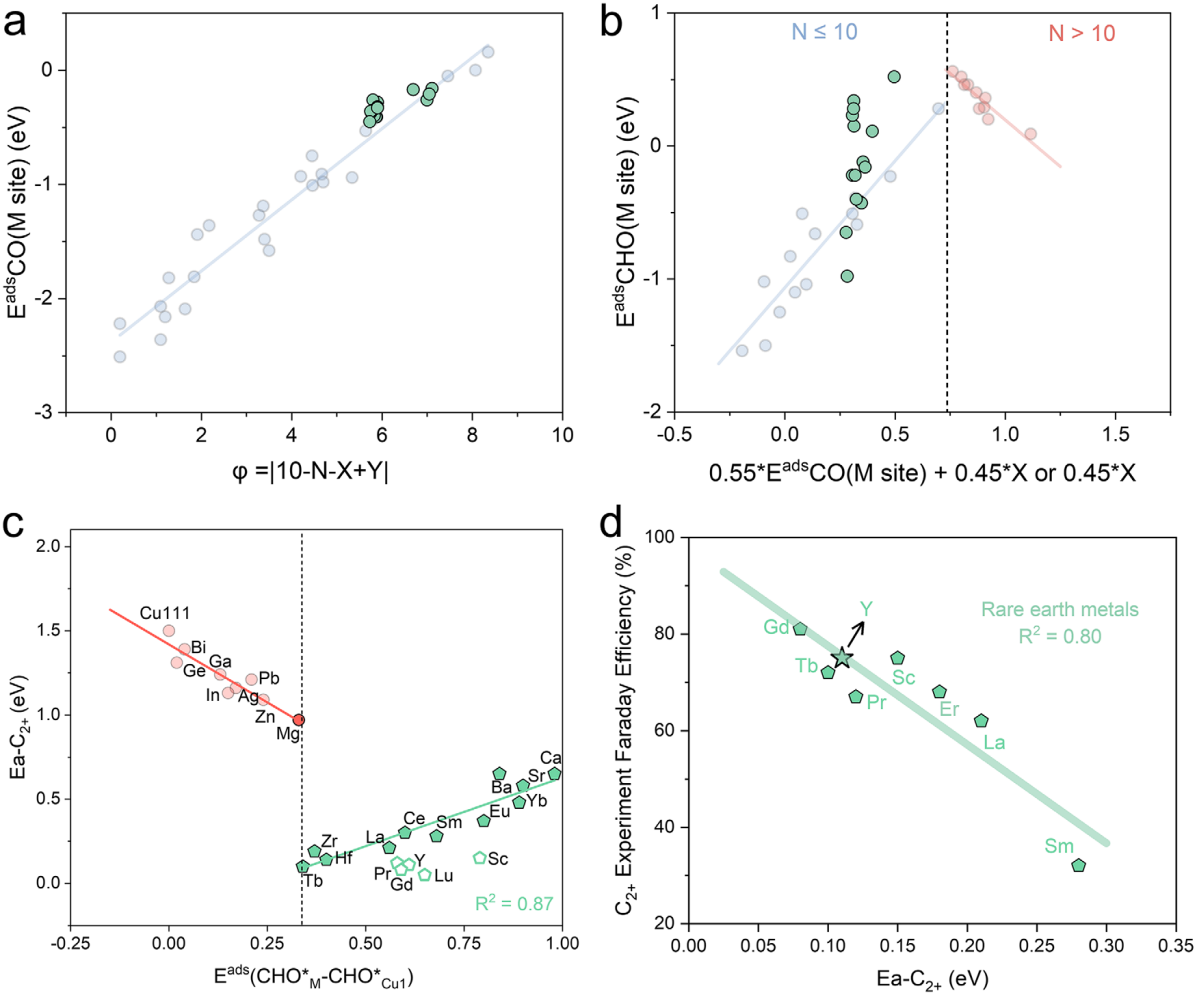
The universality of the design principles of Cu‐based SAAs. (a) The feasibility of structural descriptor of CO* adsorption energies at M sites for Cu‐based SAAs with rare earth or alkali earth metals as dopants. (b) The linear correlation between the adsorption energies of CO* and CHO*. (c) Energy descriptors for Ea‐C_2+_ of Cu‐based SAAs with rare earth or alkali earth metals as dopants and their corresponding linear relationships. (d) The linear correlation between the experimental Faraday efficiency and Ea‐C_2+_ for C_2+_ products of Cu‐based SAAs with rare earth metals as dopants.

We demonstrated the transferability of our universal design principles by applying them to alkali earth and rare earth dopants (Figure 7). Our proposed structural descriptor (φ) accurately describes the CO* adsorption energies at M sites for both alkali earth and rare earth metal dopants on Cu‐based SAAs (Figure 7a, green data points). The correlation between CHO* and CO* also lies near the previously established linear relationship (Figure 7b, green data points). Similarly, our proposed energetic descriptor ($E_{CHO*}^{ads}\left(M-Cu_{1}\right)$) effectively exhibits a linear relationship with Ea‐C_2+_ (Figure 7c and Figures S40, S41). Furthermore, Ea‐C_2+_ successfully predicts experimental C_2+_ selectivity trends among rare earth metal dopants from available references (Figure 7d) including Gadolinium (Gd), Terbium (Tb), Praseodymium (Pr), Scandium (Sc), Erbium (Er), Lanthanum (La), and Samarium (Sm). This broad applicability spans most metallic elements across the periodic table, establishing an accelerated discovery framework for Cu‐based SAA catalysts. Combined with thermodynamic stability analysis, we further identified that Cu‐based SAAs doped with the Yttrium (Y) or Lutetium (Lu) exhibits significant potential in promoting the formation of C_2+_ products. We further employed Y_1_/Cu(111) as a representative example to investigate the influence of H* coverage on CO_2_R and HER over SAAs (Figure S42). The results indicate that high H* coverage can effectively suppress HER, whereas its impact on the reaction energetics of CO_2_R is comparatively minor and does not alter the conclusion that Y_1_/Cu(111) prefers C‐C coupling rather than hydrogenation toward C_1_ products or HER, which are consistent with recent reports [46, 60].

To further validate the predictive capability of our theoretical framework, we synthesized a Y‐doped Cu‐based SAA catalyst (YCu‐SAA, Figure S43). Figure S43a and S43b show that the average diameter of the YCu particles is approximately about 40 nm. The energy‐dispersive x‐ray spectroscopy (EDS) mappings (Figure S43c) demonstrate a uniform distribution of Y elements, indicating homogeneous Y doping throughout the Cu nanostructure. EDS analysis determines the Y content to be 0.97wt%, which is comparable to the 0.68wt% obtained from inductively coupled plasma optical emission spectroscopy (ICP‐OES). Furthermore, the powder x‐ray diffraction (XRD) patterns confirm that the material after electrochemical reduction is metallic Cu, with no diffraction peaks corresponding to Y‐containing species observed (Figure S43d). X‐ray photoelectron spectroscopy (XPS) analysis was conducted to further confirm the presence of trace Y in the YCu‐SAA catalyst (Figure S43e, f). An H‐cell was used to assess the CO_2_RR performance of YCu under different electrode potentials, and aqueous solution containing 0.1 M KHCO_3_ was used as catholyte. Figure S44 presents the Faraday efficiency of YCu SAA catalysts across various electrode potential ranges. The Faraday efficiency of C_2+_ products reach 60% at ‐1.04 V *vs*. RHE (corresponding current density 15.2 mA/cm^2^), exhibiting C_2+_ selectivity closely aligned with the predicted linear trend (Figure 7d).

Further characterizations were subsequently performed to verify the presence of the Y_1_/Cu(111) SAA structure. The high‐resolution TEM(HR‐TEM) image (Figure S45) of the YCu‐SAA catalyst discerned only a stretched lattice fringe spacing of 0.21 nm of Cu (111), while no lattice stripes related to Y species were observed, which indicated that Y nanoparticles or clusters were not formed in the YCu‐SAA catalyst. Aberration‐corrected high‐angle annular dark‐field scanning transmission electron microscopy (AC HAADF‐STEM) shows structure at the atomic scale, resolving Y (marked by the red circles) dispersed in Cu (Figure S46a,b). The energy‐dispersive x‐ray spectroscopy (EDS) element mappings (Figure S46c) further certified the uniform distribution of Y elements, indicating the homogeneous doping of Y over the YCu‐SAA catalyst. The electronic information and coordination environment of the Y atom in the YCu‐SAA catalyst were further investigated by x‐ray absorption spectroscopy (XAS) measurements. As shown in Figure S46d, the Y K‐edge x‐ray absorption near‐edge structure (XANES) spectra of YCu‐SAA was between those of Y foil and Y_2_O_3_, suggesting that a portion of the Y atoms may have been oxidized during preservation and characterization. Furthermore, Fourier‐transformed extended x‐ray absorption fine structure (FT‐EXAFS) spectra of the YCu‐SAA catalyst showed that no Y‐Y coordination was present, indicating that the Y atoms were atomically dispersed in YCu‐SAA (Figure S46e). EXAFS spectral fitting results (Figure S47a,b, and Table S11) showed that the average coordination number (CN) of the Y‐O bond and the Y‐Cu bond in YCu‐SAA were 5 and 6. From the wavelet transform EXAFS (WT‐EXAFS) contour plots of YCu‐SAA (Figure S46f and Figure S47c,d), the maximum intensity at around 1.75 Å was associated with Y‐O contributions, 3.5 Å was associated with Y‐Cu contributions and no signals corresponding to Y‐Y coordination were detected, further confirming that some of Y atoms were attached to Cu in the form of single‐atom dispersions.

## 3 Conclusions

In summary, this study has elucidated the promotional mechanism and proposed universal design principle of Cu‐based SAAs for CO_2_RR to C_2+_ products through a systematic three‐stage investigation. **Stage One**: A powerful LLM‐based Catalysis AI Agent was developed and utilized the *DigCat* database for experimental data mining. It identified that Cu‐based SAAs enhanced C_2+_ selectivity by primarily promoting the formation of ethylene and ethanol rather than suppressing C_1_ and H_2_ by‐products. Subsequent computational studies addressed the stability of SAAs, adsorption behaviors of key reactive intermediates, and the thermodynamics/kinetics of C‐C coupling elementary steps. **Stage Two**: The Catalysis AI Agent was leveraged to analyze correlations between experimental and theoretical data. This analysis strikingly highlighted that classifying guest metal dopants is a prerequisite to elucidating structure‐selectivity relationships. Consequently, we established an energy descriptor ($E_{CO*}^{ads}\left(M-Cu_{1}\right)$ or $E_{CHO*}^{ads}\left(M-Cu_{1}\right)$) enabling the classification of SAAs into five distinct categories. Crucially, this energy descriptor correlates with Ea‐C_2+_ across different Cu‐based SAA types, which accurately captures the selective trends toward C_2+_ products. Additionally, a remarkably simple structural descriptor was developed to predict C‐C coupling precursor species' adsorption strength, allowing direct estimation of Ea‐C_2+_ from intrinsic properties of guest metal. **Stage Three**: We demonstrated the universality of the structural descriptor by extending its application to alkaline‐earth and rare‐earth dopants. This principle unravels the promotional mechanism and structure‐selectivity relationships governing Cu‐based SAAs for CO_2_RR toward C_2+_ products. This paradigm shift, moving from empirical trial‐and‐error toward AI‐accelerated and theory‐guided catalyst design, holds substantial promise for expediting the discovery of next‐generation materials. Most strikingly, our study highlights a transformative paradigm in materials science, where a well‐trained scientific AI Agent and large‐scale experimental database not only predict and rationalize catalyst performance, but also inspire generalizable design principles for future discovery. The integration of AI with active learning, inverse design, and other advanced methodologies is expected to significantly enhance the research process for novel catalysts. However, it should be emphasized that when LLMs are extensively integrated into the catalyst development process, their application must be accompanied by rigorous human oversight. This is necessary to ensure the validity of data analysis and model outputs, as well as to mitigate potential risks arising from LLM‐generated hallucinations or inaccuracies.

## Conflicts of Interest

The authors declare no conflicts of interest.

## Supporting information




**Supporting File 1**: anie71535‐sup‐0001‐SuppMat.docx.

**Supporting File 2**: anie71535‐sup‐0002‐Data.zip.

## Data Availability

All data that support the findings of this study are available within the article and the Supplementary Information, or from the corresponding author upon reasonable request. The key experimental and computational data from this work are also available in the Digital Catalysis Platform (*DigCat*: www.digcat.org).
