# Supplementary material for: Catalysis AI Agent Guides Discovering the Universal Design Principle of Cu‐Based Single‐Atom Alloy Catalysts for CO2 Electroreduction
Source: Angew Chem Int Ed Engl. 2026 Feb 24;65(21):e24612. doi: 10.1002/anie.202524612 (PMC13182201; doi:10.1002/anie.202524612)
Supplement: Supplementary file 1 — Supporting File 1: anie71535‐sup‐0001‐SuppMat.docx. [file ANIE-65-e24612-s002.docx]

**Supporting Information**

**Catalysis AI Agent Guides Discovering the** **Universal Design Principle of Cu-based Single-atom Alloy Catalysts for CO_2_ Electroreduction**

Xuning Wang^1#^, Zhong Li^1,4#^, Di Zhang^2^, Hao Li^2^*, Haoxiang Xu^1,3*^ and Daojian Cheng^1,3*^

^1^Beijing Key Laboratory of Intelligent Design and Manufacturing for Hydrogen Energy Materials, State Key Laboratory of Organic-Inorganic Composites, Beijing University of Chemical Technology, Beijing 100029, China

^2^Advanced Institute for Materials Research (WPI-AIMR), Tohoku University, Sendai 980-8577, Japan.

^3^Deep Intelligence Experiment Technology (Beijing) Co., Ltd., Beijing 100029, China

^4^College of Chemistry and Chemical Engineering, Tarim University, Xinjiang 843300, China

^*^E-mail: c[hengdj@mail.buct.edu.cn](mailto:chengdj@mail.buct.edu.cn); [xuhx@mail.buct.edu.cn](mailto:xuhx@mail.buct.edu.cn); [li.hao.b8@tohoku.ac.jp](mailto:li.hao.b8@tohoku.ac.jp)

^#^These authors contribute equally.

**Part 1 Supplementary Notes**

**1.1 Computational details**

All calculations in this work were carried out with the Vienna Ab Initio Simulation Package (VASP).^1^ The Cu(111) surface was modeled using a four-layer slab and a (4x4) unit cell. One of the surface Cu atoms was replaced by a dopant for the SAA model (M_1_/Cu111). The uppermost two layers in the models are relaxed. A vacuum space with a height of 15 Å in the vertical direction was employed to avoid the possible interaction between two periodic slabs. The interaction between ion and core electrons was described by the projector augmented wave (PAW) method,^2^ and plane waves with an energy cutoff of 500 eV were used to expand the Kohn-Sham (KS) wave functions by using a 3x3x1 Monkhorst-Pack mesh to sample the first Brillouin zone. The revised Perdew-Burke-Ernzerhof (RPBE)^3^ form of the generalized gradient approximation (GGA) exchange and correlation functional was used in all calculations reported herein. To assess the influence of RPBE on the energy description of f-electron correlated systems, we employed the key energy descriptor E^ads^(CHO*_M_–CHO*_Cu1_) as a representative example to evaluate the performance of the DFT+U method (U-J = 5.0 eV) for rare earth systems (**Figure S48**). The DFT+U approach may slightly adjust the absolute energy values of individual systems due to suboptimal U parameter calibration for these elements, but it exerts a little effect on the overall trend of adsorption energies. All calculations were converged to 0.02 eV Å^−1^ for maximum force and 1.0x10^−5^ eV atom^−1^ for the self-consistent field with spin polarization. Climbing-image Nudged Elastic Band (CI-NEB)^4^ and dimer^5^ methods were used to find reaction path way and transition state. The convergence criteria for electronic energy and force were 1.0x10^−7^ eV atom^−1^ and 0.1 eV/Å during the search for transition state, respectively.

We evaluated the thermodynamic and electrochemical stabilities of SAAs by the formation energy (E_for_), segregation energies (E_seg_), aggregation energies (E_agg_), adatom formation energies (E_for_^adatom^) and dissolution potential (U_diss_)^6^, which are defined as

E_for_ = E_SAA_ + E_Cu1-bulk_ - E_Cu-slab_ - E_M1-bulk_

E_seg_ = E_SAA_ - E_SAA-sub_

E_agg_ = E_dimer_ + E_Cu-slab_ -2E_SAA_

U_diss_ = U^0^_diss_(metal, bulk) - E_for_/N_e_ + 0.0592 x pH

where E_M_ is the DFT energy of the metal atom in its most stable bulk structure, E_SAA_ and E_Cu-slab_ are the DFT energies of SAAs and Cu(111) or Cu(100) slabs, E_SAA-sub_ is the DFT energy when the dopant is in the subsurface layer, E_dimer_ is the DFT energy of the metal slab when two surface dopant atoms are brought together, U^0^diss(metal, bulk) and N_e_ are the standard dissolution potential of bulk metal and the number of electrons involved in the dissolution, respectively.

We used the JDFTx code^7^ to conduct grand-canonical DFT calculations for the initial, transition, and final states of the C-C coupling reaction to determine the energy at the operating electrode potential of the CO_2_RR with the implicit CANDLE solvation model^8^. We set the concentrations of K+ and F- ions to 0.1 M in JDFTx to simulate the ionic effects in the fluid model. All other settings are similar to those in VASP calculations. The impact of solvent effects on adsorption energy was assessed using the VASPsol package^9^.

All simulations with neural network potential were carried out by LASP software (www.lasphub.com)^10^. Molecular dynamic simulation with neural network potential (NN-MD) was performed at a constant temperature (T = 300 K) within the NVT ensemble. The NN potential^11^ used in this calculation is provided by LASP software.

**1.2 Machine learning.**

The feature set for machine learning training was constructed using data derived from theoretical calculations and the intrinsic physicochemical properties of the dopants, while the experimentally measured Faraday efficiency values were designated as the target variable. Regarding data preprocessing, samples associated with CHO* dissociation were excluded from the training dataset, and the CO* adsorption energy values for systems exhibiting CO* desorption were approximated using estimated values. The input data were randomly partitioned into training and testing sets in a 4:1 ratio. To evaluate the performance of various regression models, a comparative analysis was conducted using the coefficient of determination (R²) and the Mean Absolute Error (MAE) as evaluation metrics. The models assessed included Linear Regression, Random Forest (RF), and Neural Network (NN). Each model was implemented with either default or predefined hyperparameters to ensure consistency and reproducibility across evaluations. To optimize the hyperparameters of the Random Forest model, grid search combined with cross-validation was employed for all tunable parameters. The best-performing estimator identified through this process was selected as the final RF model for predicting chemical properties.

**1.3 Microkinetics modelling.**

All the elementary reaction steps of C_2_H_4_ formation are as follows:

R1 CO(aq) + * ⇔ CO*

R2 CO* + H_2_O(aq) + e- ⇔ CHO* + OH-(aq)

R3 CO* + CO* ⇔ COCO**

R4 CO* + CHO* ⇔ COCHO**

R5 COCO** + 6H_2_O(aq) + 8e- → 2* + C_2_H_4_(g) + 8OH-(aq)

R6 COCHO** + 5H_2_O(aq) + 7e- → 2* + C_2_H_4_ (g) + 7OH-(aq)

The ordinary differential equations under intermediate steady state hypothesis:

$$\theta_{CO}{{= K_{1}\theta}_{*}a}_{CO}$$

0 =$\frac{{d\theta}_{CHO}}{dt}$=$rate(R2)-rate(R4)=$ $\left( k_{2}^{+}{a_{H2O}\theta}_{CO}-{k_{2}^{-}\theta}_{CHO}a_{OH-} \right)-\left( k_{4}^{+}{\theta_{CO}\theta}_{CHO}-k_{4}^{-}\theta_{COCHO} \right)$

0 = $\frac{{d\theta}_{COCO}}{dt}$ = $rate(R3)-rate(R5)$= $(k_{3}^{+}\theta_{CO}\theta_{CO}-k_{3}^{-}\theta_{COCO})-(k_{5}^{+}\theta_{COCO}a_{H_{2}O})$

0 = $\frac{{d\theta}_{COCHO}}{dt}$ = $rate(R4)-rate(R6)$= $(k_{4}^{+}\theta_{CO}\theta_{CHO}-k_{4}^{-}\theta_{COCHO})-(k_{6}^{+}\theta_{COCHO}a_{H_{2}O})$

1 = $\theta_{*}{+\theta}_{CO}{+\theta}_{CHO}{+\theta}_{COCO}{+\theta}_{COCHO}$

where $a_{H_{2}O}$ is 1 and $a_{{OH}^{-}}$ is 10^-7.2^ (pH = 6.8). It is worth noting that we employ CO as a reactant instead of CO_2_ due to the pivotal role of C-C coupling in generating C_2+_ products. And the facile activation of CO_2_ to CO by Cu allows us to disregard it here, aligning with the simplification made by Head-Gordon et al.^1^ Furthermore, we assume that the solution is fully saturated with CO, thereby ensuring that the concentration of CO in the liquid phase remains constant over time. Consequently, when solving the equation, $a_{CO}$ can be eliminated.^1^ For the equilibrium constant K, it can be obtained by the following equation:

K_i_=$\exp\left( \frac{\text{-∆E}_{i}^{\#}}{\text{RT}} \right)$

where ${\text{-∆}\text{E}}_{i}^{\#}$ represents the adsorption energy of the adsorbate *i*, R is the gas constant and T represents the temperature. The adsorption free energy is calculated as follows:

$$E^{ads}=E_{ads/slab}-E_{slab}{-E}_{ads}$$

where $E_{ads/slab}$ is the total energy of the slab with adsorbate, $E_{slab}$ is the energy of the slab, and $E_{ads}$ is the energy of the adsorbate in gas phase. The reaction rate constant (*k*_i_) is given by the following equation based on transition state theory:

*k*_i_ =$\frac{\text{kT}}{\text{h}}\exp\left( \frac{\text{-E}_{a}}{\text{kT}} \right)\text{=}\frac{\text{kT}}{\text{h}}\exp\left( \frac{\text{-(}E_{\text{TS}}\text{-E}_{\text{IS}}\text{)}}{\text{kT}} \right)$

where Ea is the change in standard molar free energies between the transition state and the initial state for step i. And $E_{\mathrm{TS}}$ and $E_{\mathrm{IS}}$ are the free energies at the transition state and initial reactants, k is the Boltzmann constant, and h is the Planck constant.

The energy barrier associated with the hydrogenation step of CO* is approximately replaced by the thermodynamic reaction energy. It should be noted that for the multi-step hydrogenation processes of COCO** and COCHO**, we simplify the reaction process by integrating the following multi-step PECT reactions to a single step with no kinetic barriers. This simplification is justified since the multi-step hydrogenation following the selectivity-determining step tend to occur rapidly at the electrode potential of CO_2_RR, leading to the production of ethylene (or other C_2+_ product).^12-14^ Zhang et al.^15^ also employed the same simplified method to define the reaction energy for each step following C-C coupling.

The rate of C_2_H_4_ formation is given by:

$$\text{rate}\left( \text{R5} \right)\text{+rate}\left( \text{R6} \right)\text{ = }k_{5}^{+}\theta_{COCO}a_{H_{2}O}+k_{6}^{+}\theta_{COCHO}a_{H_{2}O}$$

**1.4 Experimental details.**

**Materials.** Copric chloride dihydrate Cu(NO_3_)_2_·5H_2_O, purity > 99%, Macklin Inc.), Yttrium(III) nitrate hexahydrate YCl_3_·6H_2_O, purity >99.5%, Macklin Inc.), Ethanol(CH_3_CH_2_OH, purity > 99.7%, Macklin Inc.) Ethylene glycol (HOCH_2_CH_2_OH,purity > 98%, Macklin Inc.) Potassium bicarbonate (KHCO_3_, purity = 99.5%, Macklin Inc.), deuterium oxide (D_2_O, purity > 99.9, Anangi Chemical Reagent Co., Ltd. ), gas diffusion electrode (GDE, YLS-30T), Nafion 117 dispersion (5 wt%,, Macklin Inc.). All materials were used directly with no further purification.

**Catalyst synthesis.** Firstly, the catalyst was fabricated by the solvothermal method, mixture comprising 400 mg Cu(NO_3_)_2_·5H_2_O, and 190 mg YCl_3_·6H_2_O, was added into 20 mL ethylene glycol, and stirred overnight. Then, the above mixture was transferred into 50 mL hydrothermal autoclave reactor and heated at 160 °C for 6 h in electric thermostaticdrying oven, the precipitate obtained was thoroughly washed multiple times with ethanol and deionized water, followed by drying at 60 °C overnight. Subsequently, after 5 mg the particles was dispersed on GDE, a current density of 100 mA cm^-2^ was carried out for a duration of 1800 s, during which they were in situ transformed into Y-doped metallic copper particles (Y-Cu).

**Electrochemical test electrode preparation**. A uniformly dispersed ink-like dispersion solution was obtained by adding 5 mg of catalyst and 20 uL of 5% Nafion 117 solution in 1000 uL of ethanol followed by sonication for 30 min. The catalyst ink was uniformly dripped onto GDE with a pipette gun under a baking lamp to obtain carbon paper with a loading of 1.5 mg/cm^2^ as the working electrode.

**Catalysts characterization.** Aberration-corrected high-angle annular dark field scanning transmission electron microscopy (AC HAADF-STEM) images were performed on JEM-ARM300F (JEOL) with accelerating voltage of 300 kV. X-ray diffraction (XRD) pattern was recorded using an Ultima IV diffractometer equipped with a high speed detector D/teX-Ultra using Cu Kα radiation from 10° to 90° with 0.02° step at a scan speed of 10°/min. Chemical compositions and elemental valence of as-synthesized catalyst were analyzed by X-ray photoelectron spectroscopy (XPS) based on Thermo Scientific K-Alpha with a monochromatic Al Kα (1486.6 eV) X-ray source at an operating source power of 12 kV × 6 mA. All XPS peaks were calibrated with the surface contamination C1s binding energy at 284.8 eV. The loading of Y was measured by ICP-OES/MS using Thermo ICAP PRO. The Y K-edge extended X-ray absorption fine structure (EXAFS) spectra were recorded in the total electron yield (TEY), The spectra were processed and analyzed by the software codes Athena and Artemis..

**XAFS analysis.** The obtained XAFS data was processed in Athena (version 0.9.26) for background, pre-edge line and post-edge line calibrations. Then Fourier transformed fitting was carried out in Artemis (version 0.9.26). The k^3^ weighting, k-range of 3-14 Å^-1^ and R range of 1 - ~3 Å were used for the fitting of Cu foil; k-range of 3 - 11 Å^-1^ and R range of 1 - ~3 Å were used for the fitting of samples. The four parameters, coordination number, bond length, Debye-Waller factor and E_0_ shift (CN, R, ΔE_0_) were fitted without anyone was fixed, the σ^2^ was set. For Wavelet Transform analysis, the χ(k) exported from Athena was imported into the Hama Fortran code. The parameters were listed as follow: R range, 1 - 4 Å, k range, 0 - 12 Å^-1^ for samples; k weight, 3; and Morlet function with κ=10, σ=1 was used as the mother wavelet to provide the overall distribution

**1.5 Catalysis AI Agent**

**Development of the Catalysis AI Agent.** The Catalysis AI Agent was developed using OpenAI’s custom GPTs and Actions, enabling integration with local knowledge bases and automated analysis tools.^16, 17^ It specializes in Cu-based single-atom catalysts (SAAs) for C₂ formation, evaluating efficiency, selectivity, and C–C coupling pathways (CO-CO, CO-CHO). The Catalysis AI Agent processes structured datasets (CSV files), classifies elements by mechanistic roles, identifies scaling relationships, and proposes descriptors. It provides clear, scientifically rigorous explanations, and when data are absent, offers conceptual insights and suggests useful data types.

**Video 1**


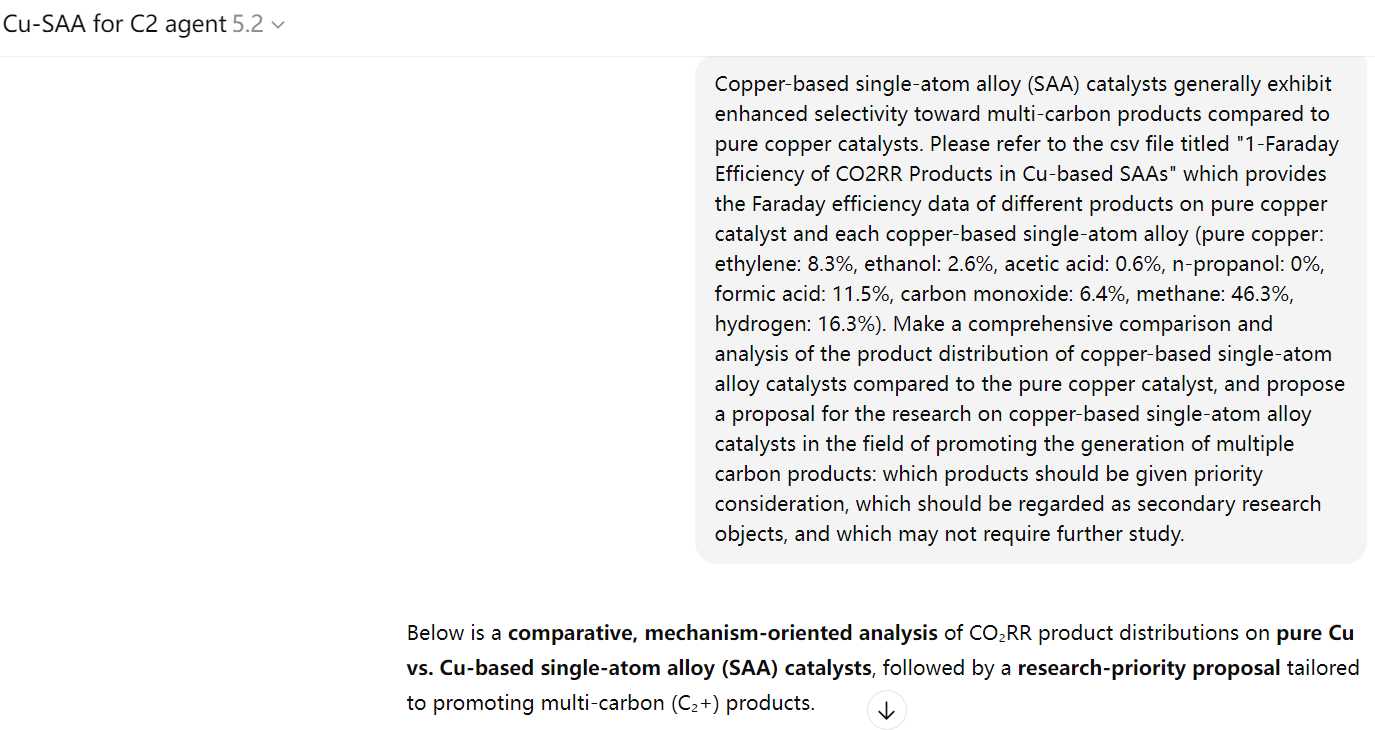

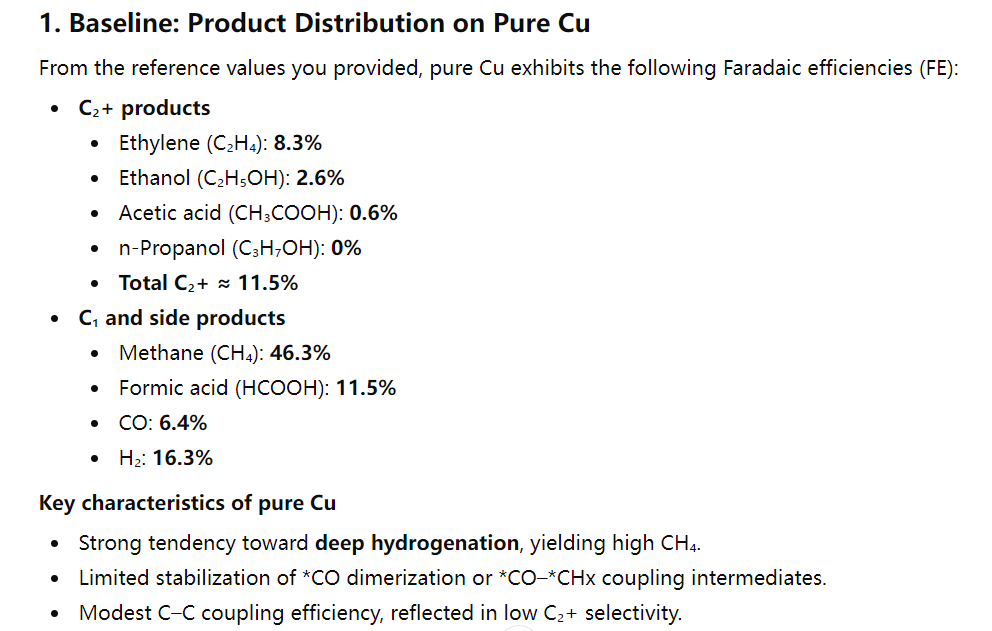

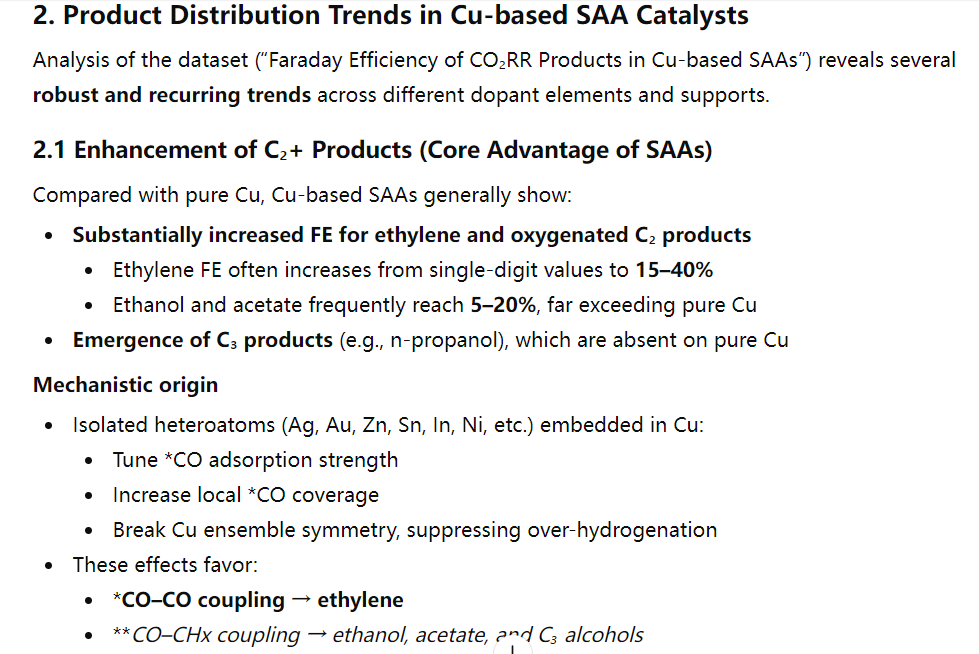

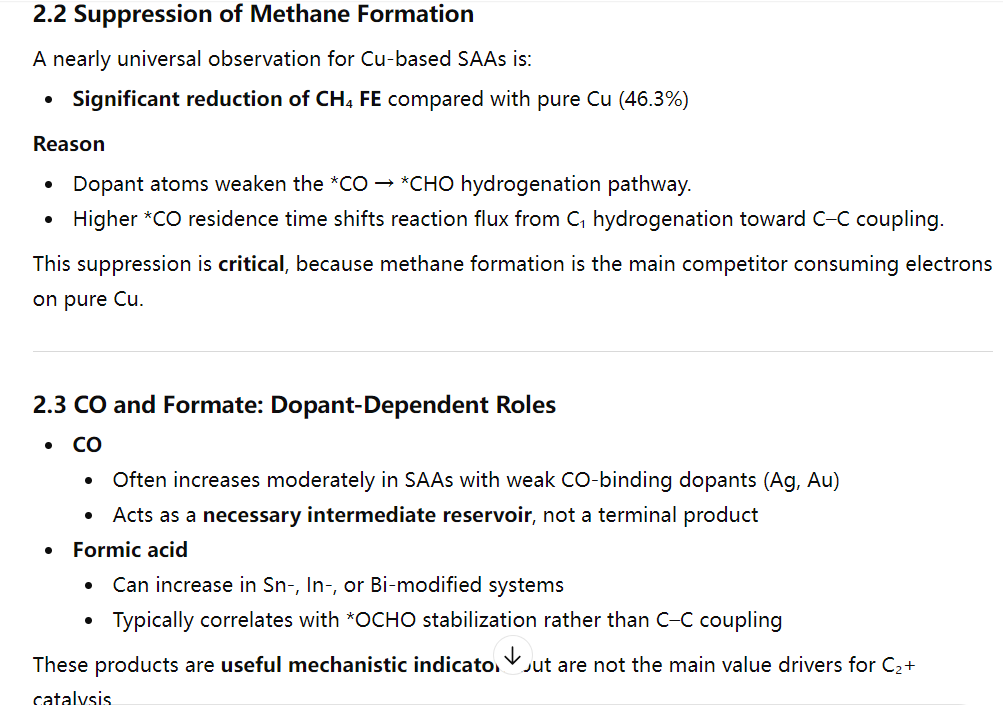

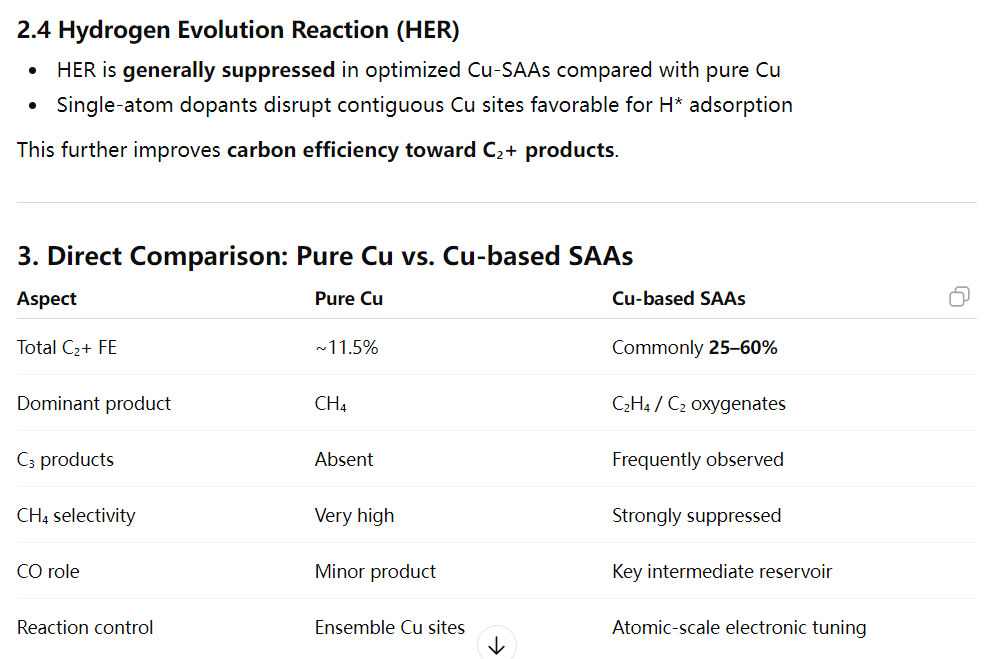

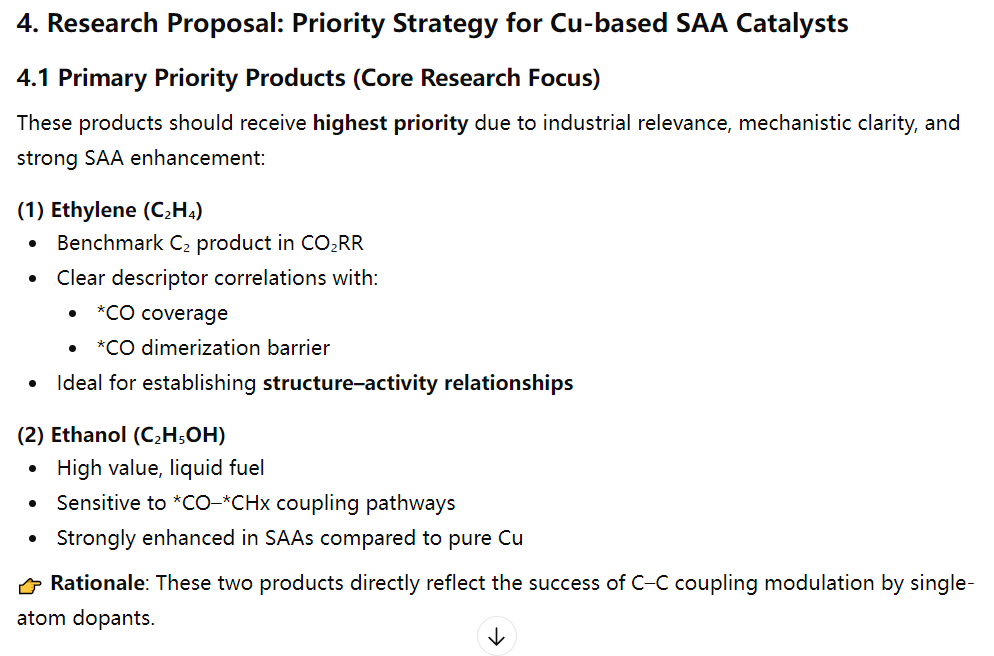

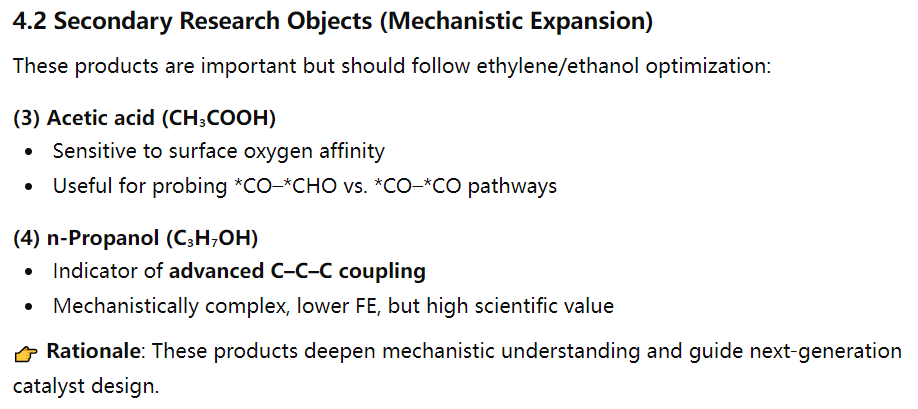

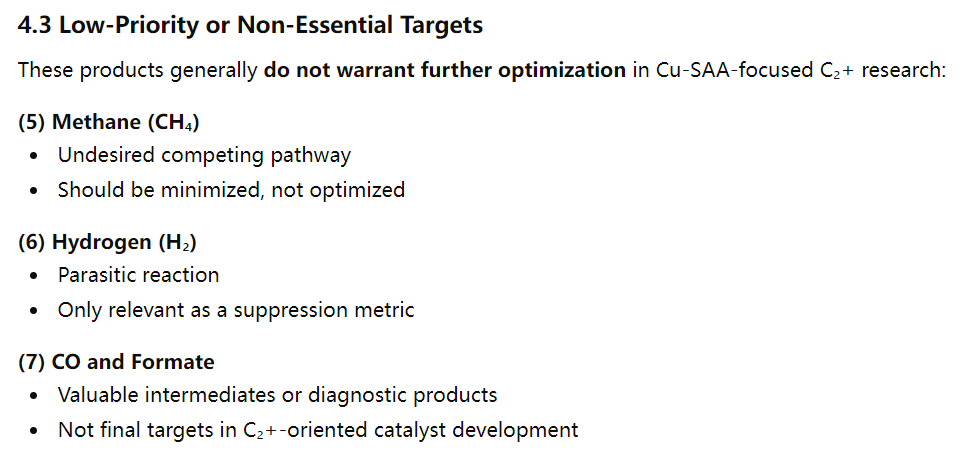

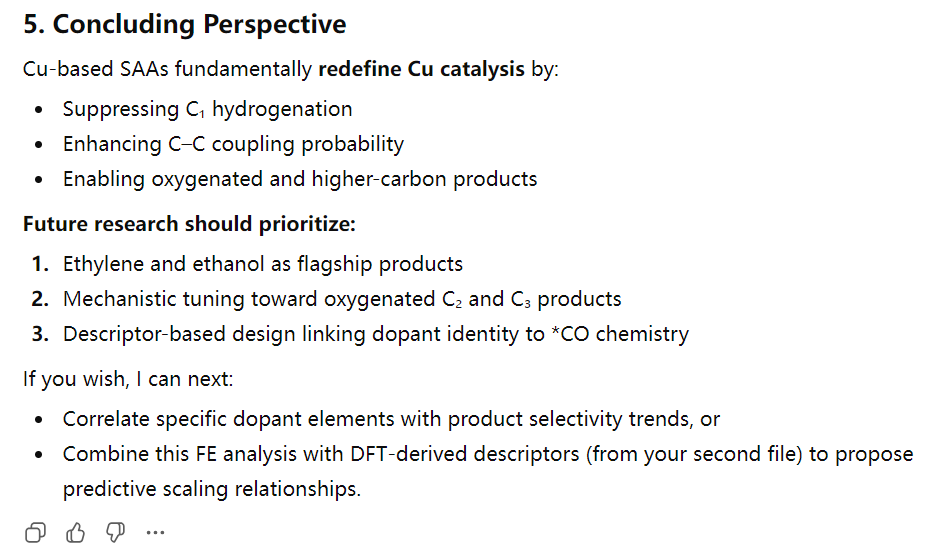


**Video 2**


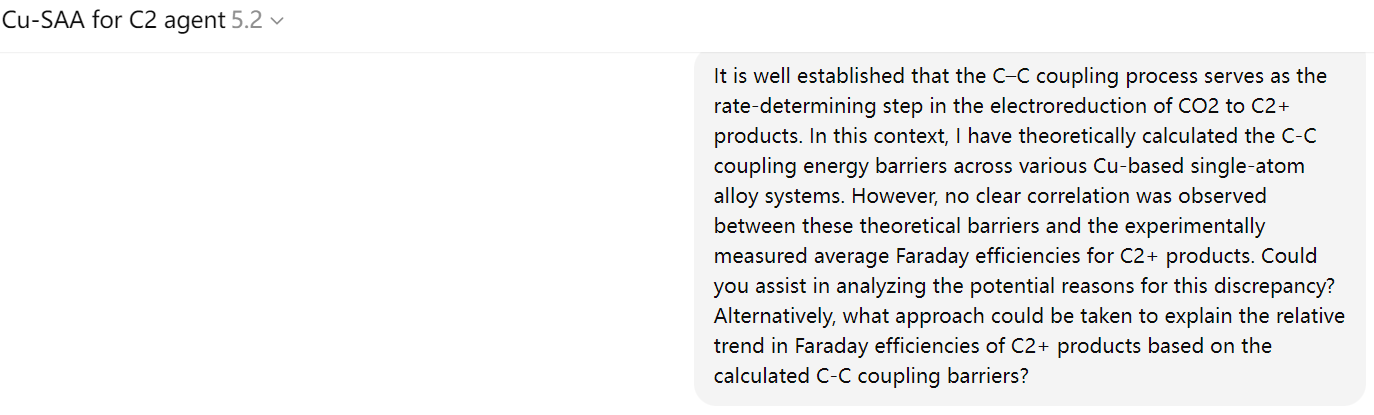

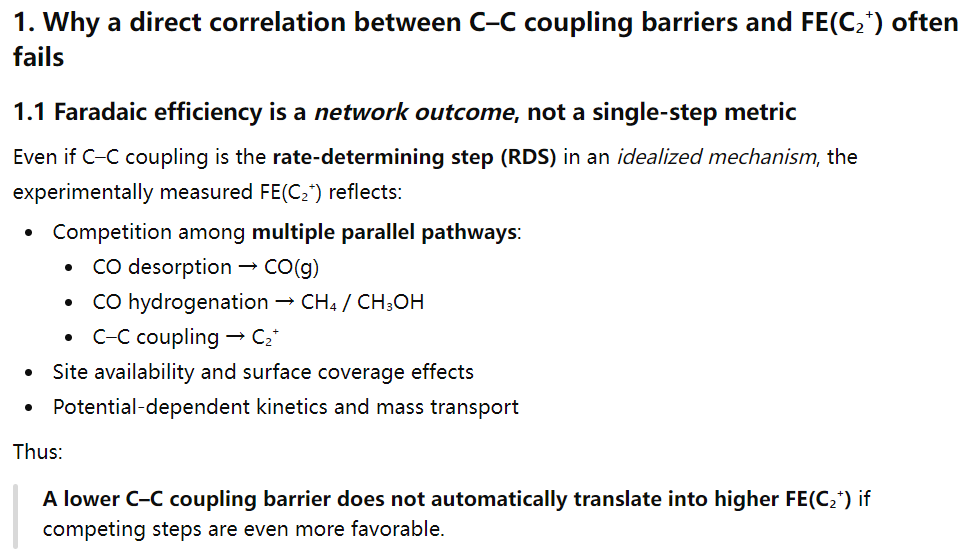

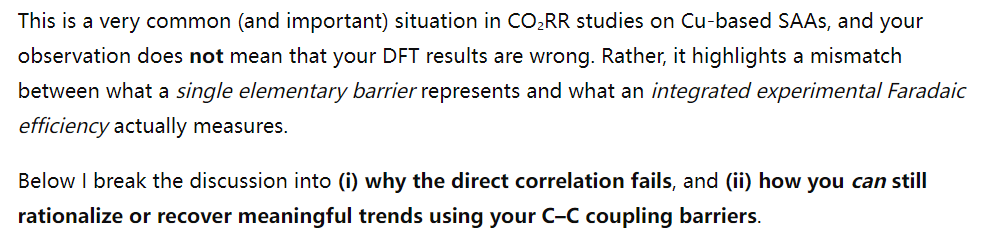

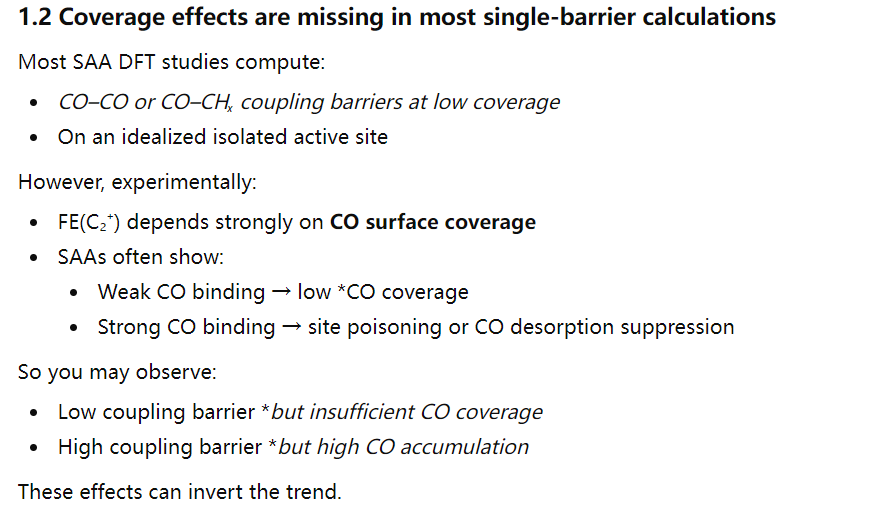

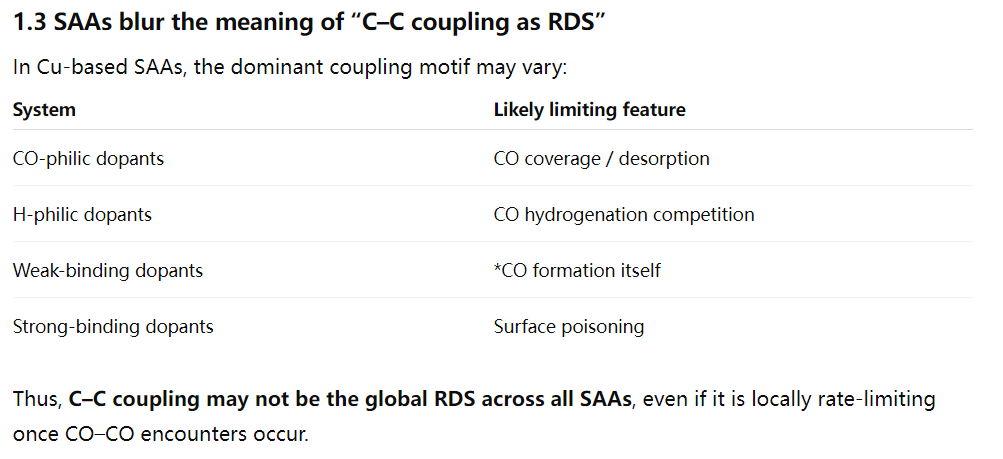

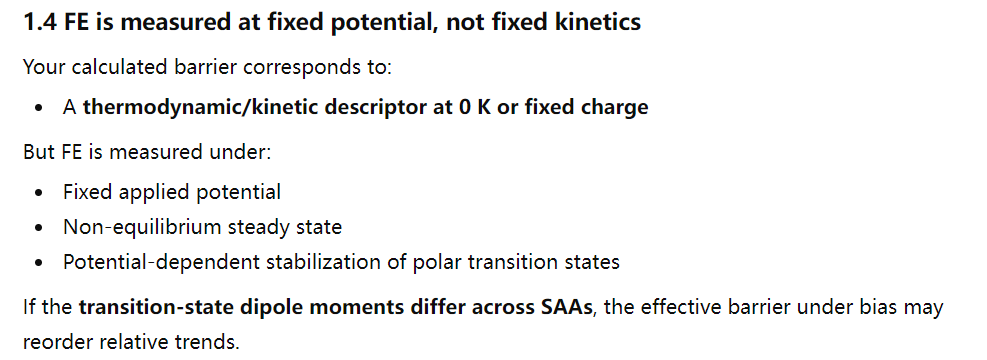

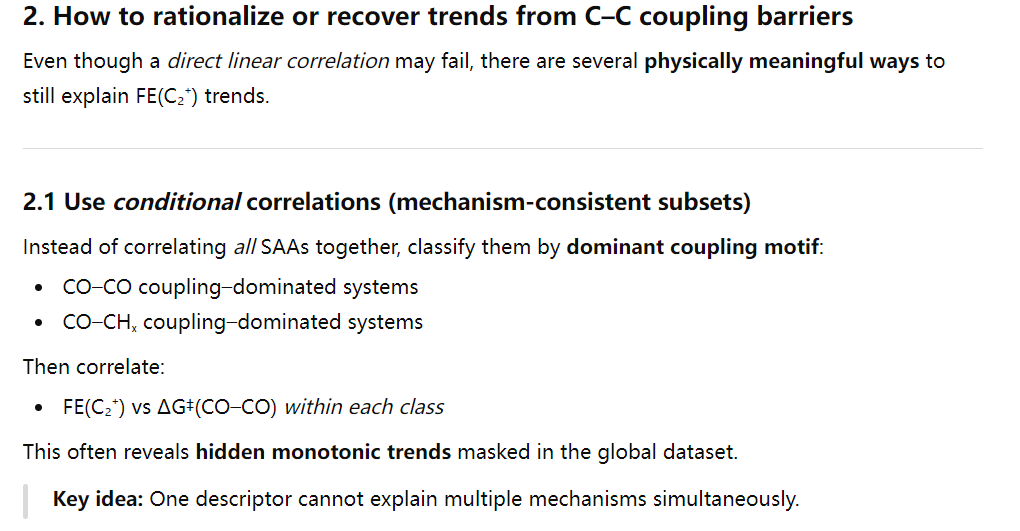

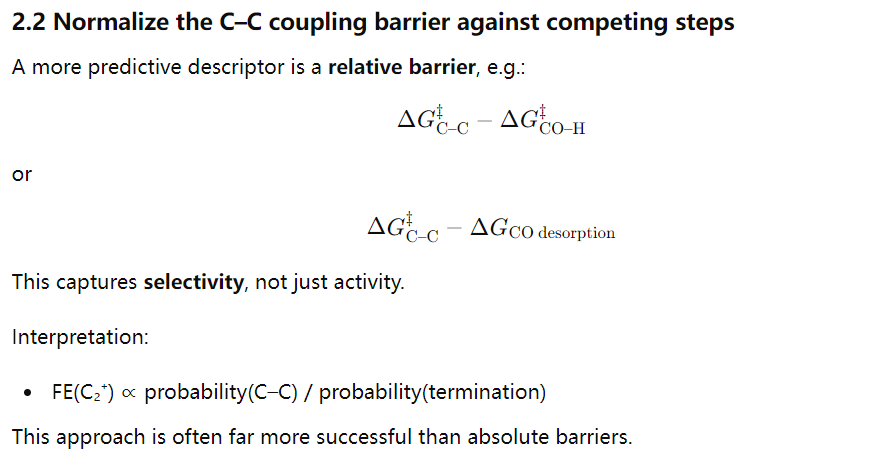

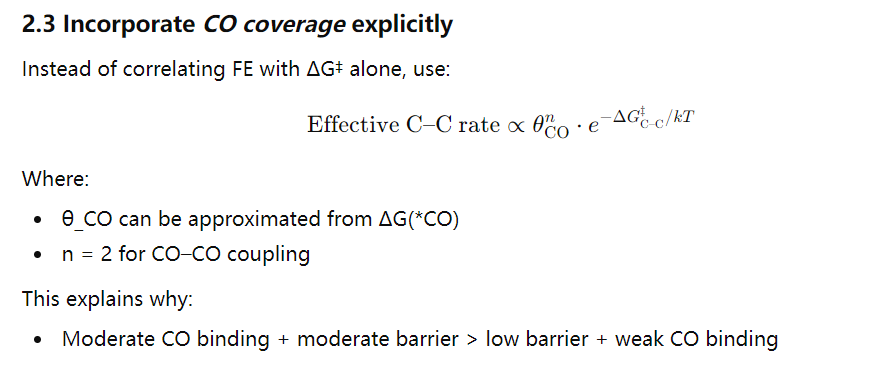

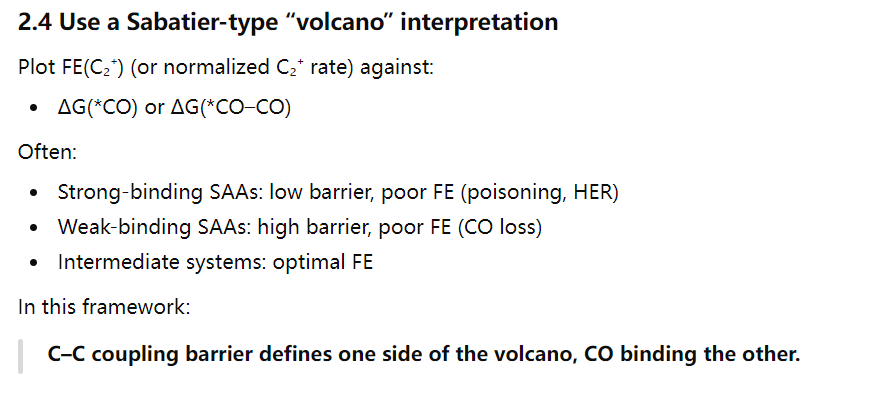

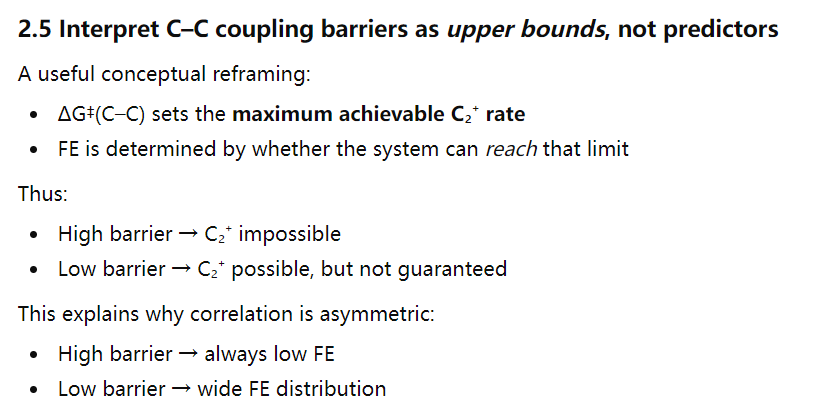

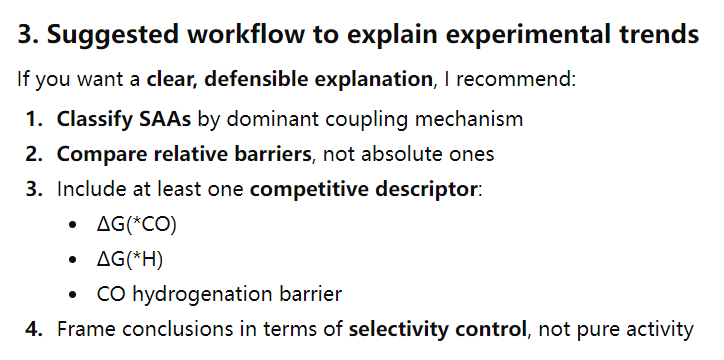


**Video 3**


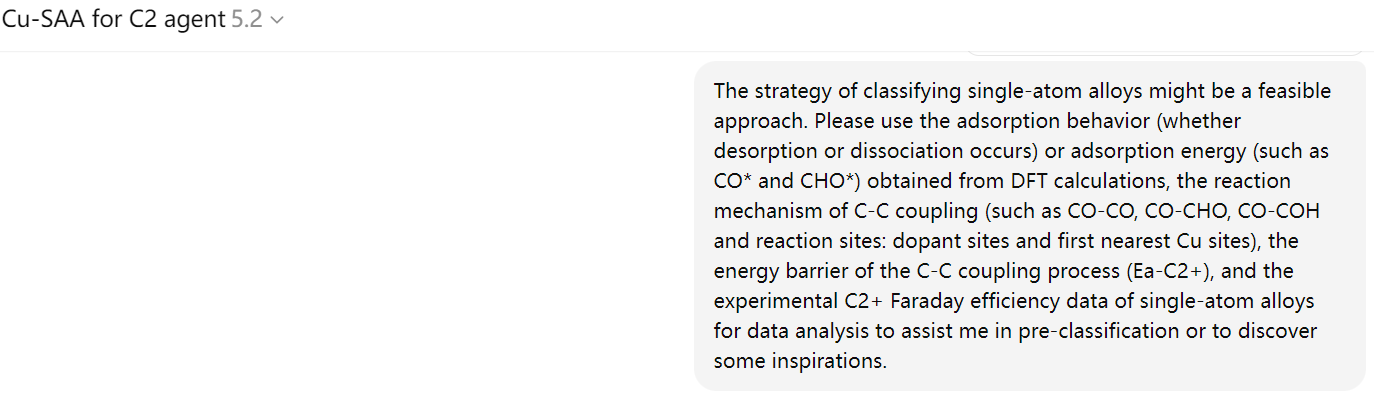

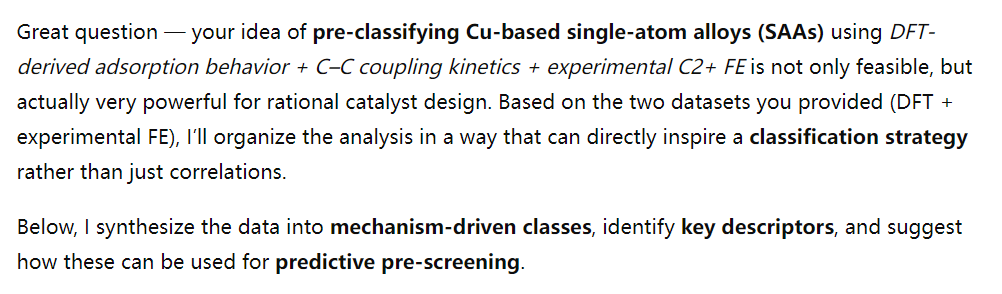

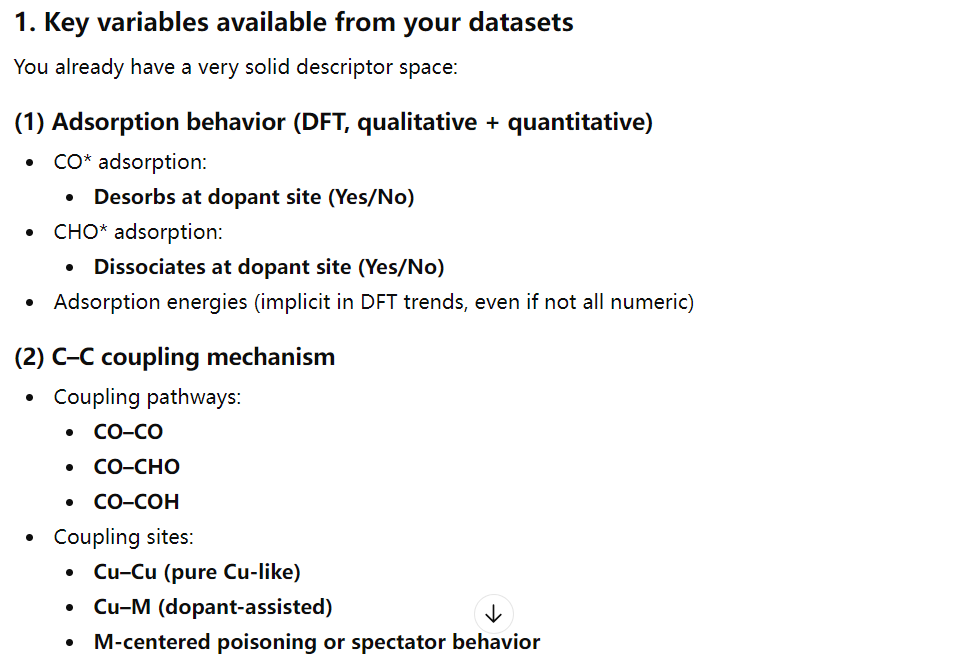

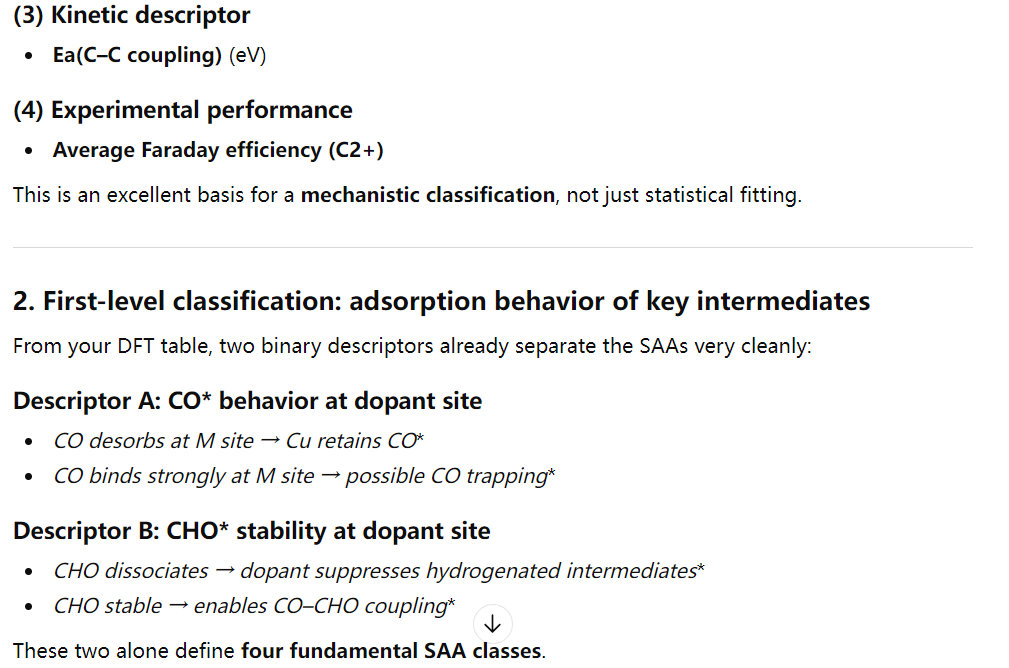

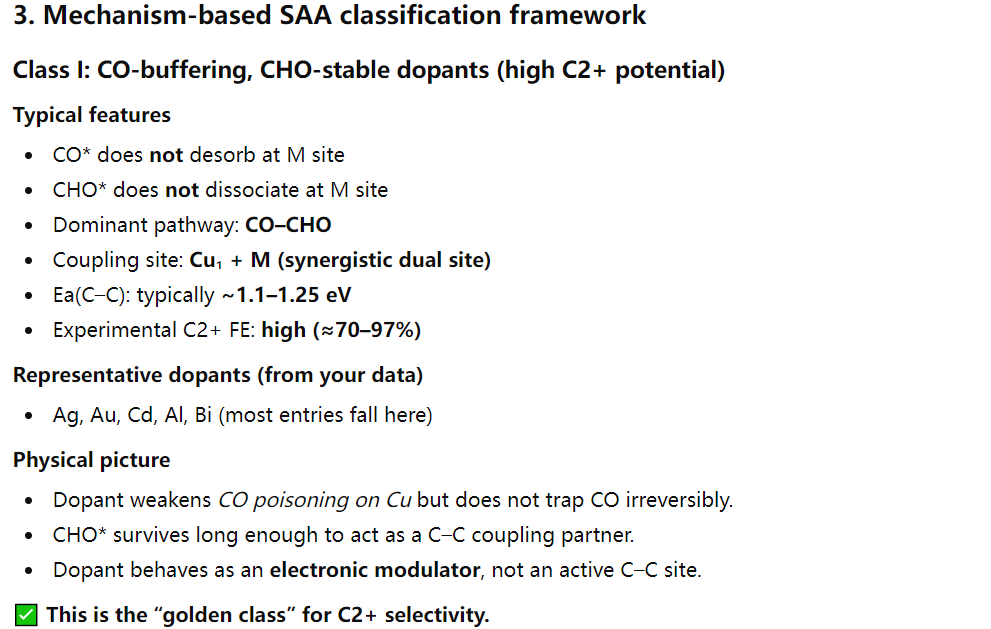

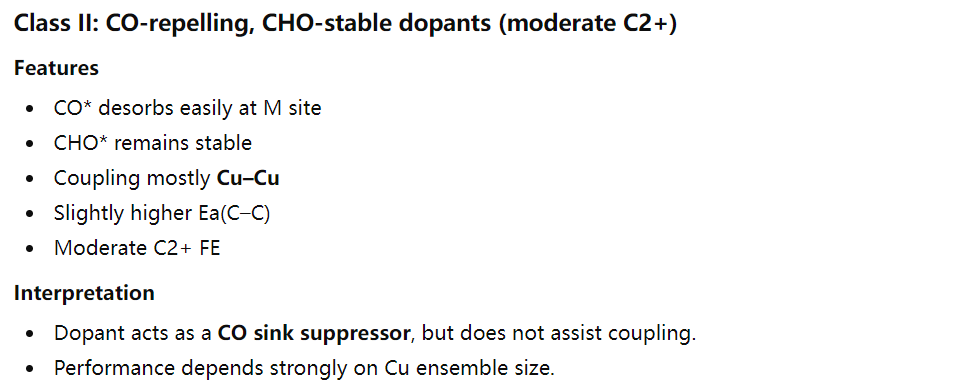

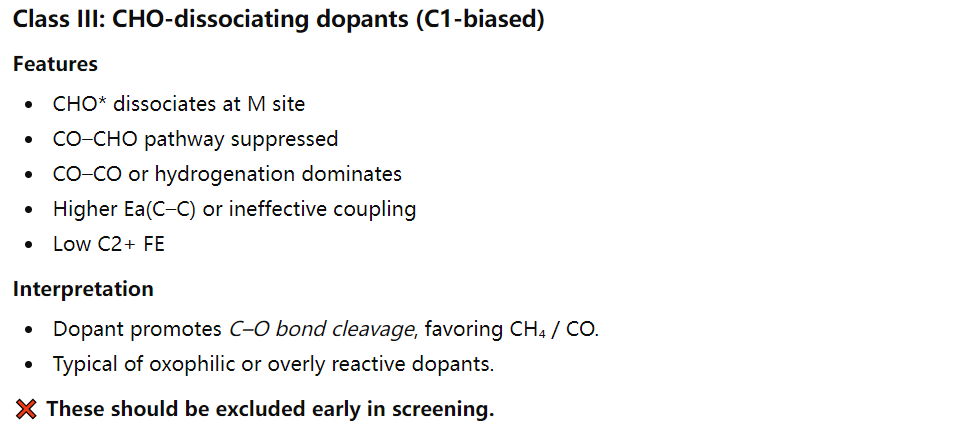

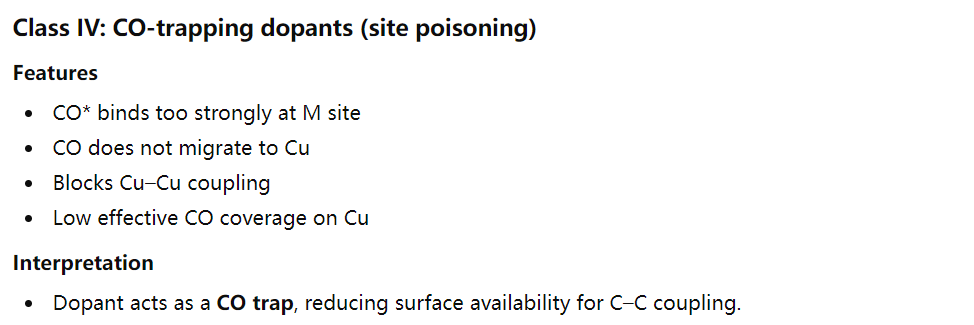

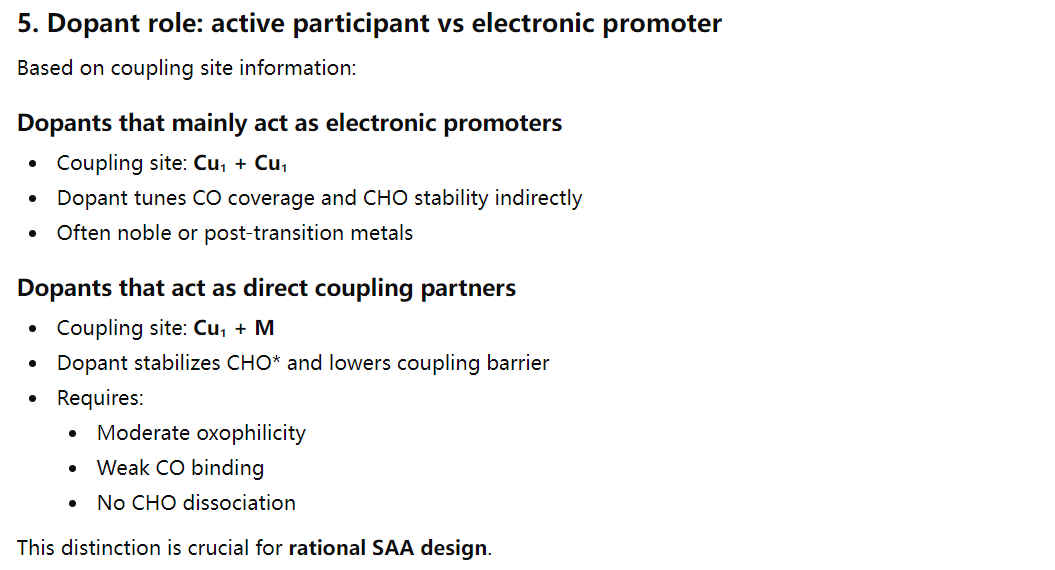

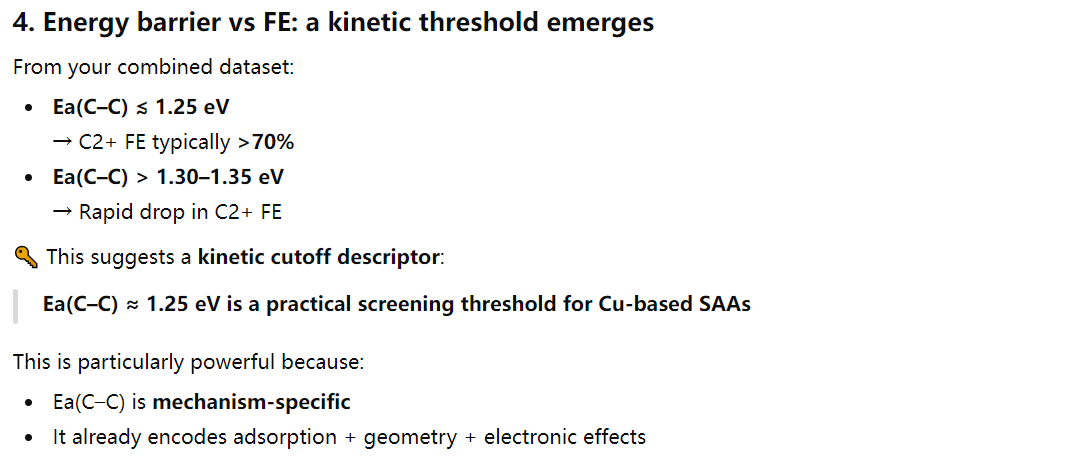

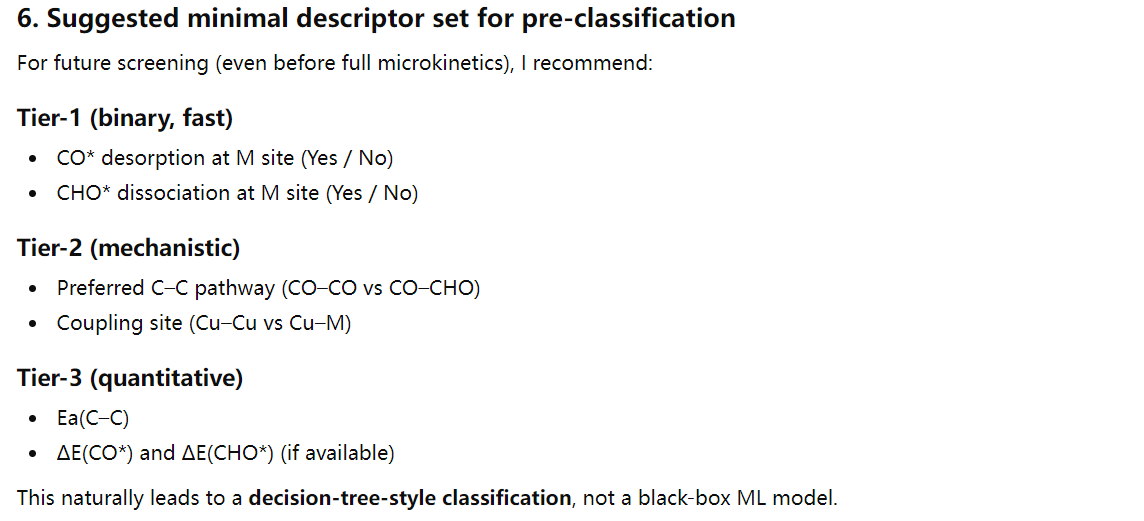

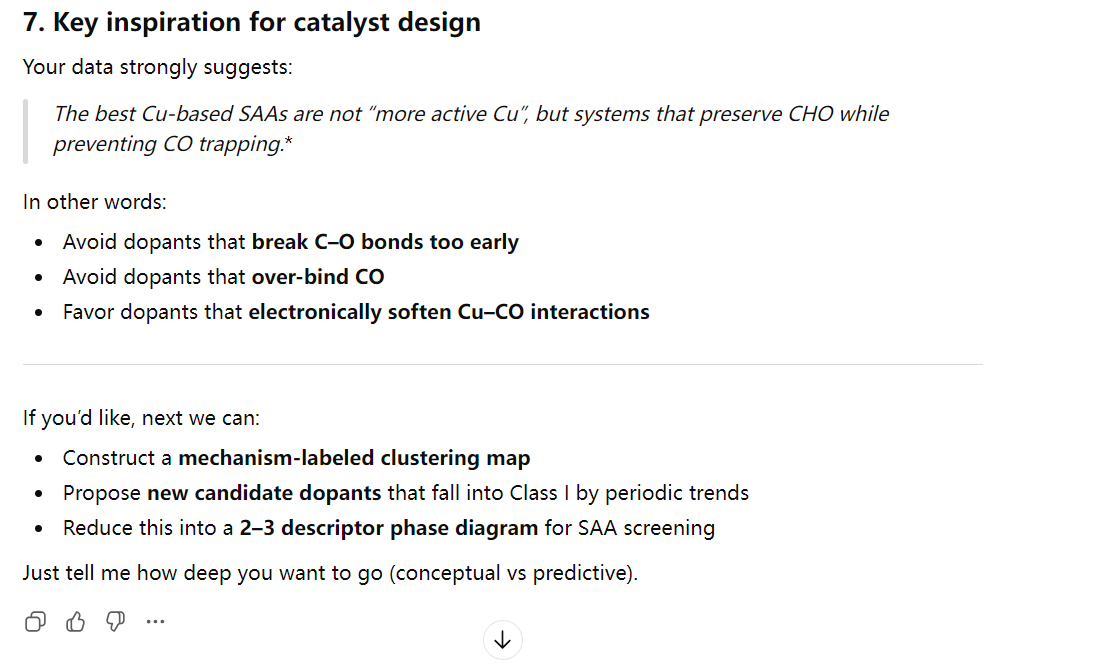


**Video 4**


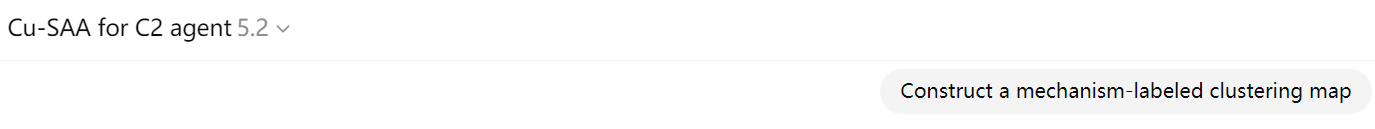

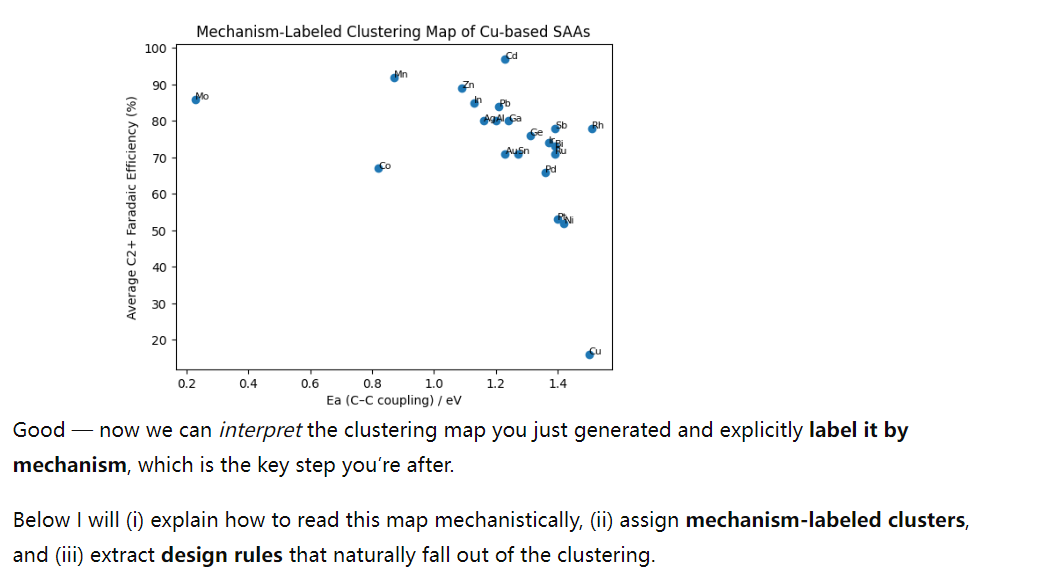

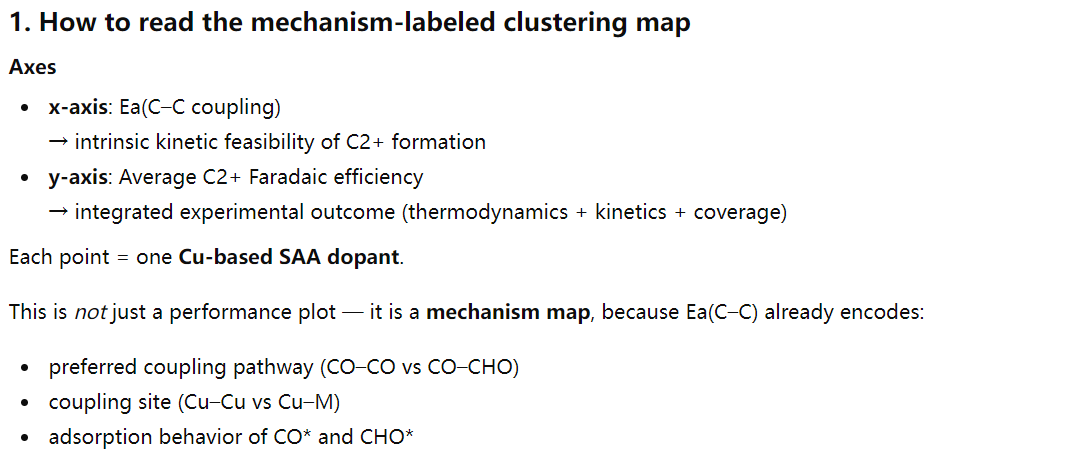

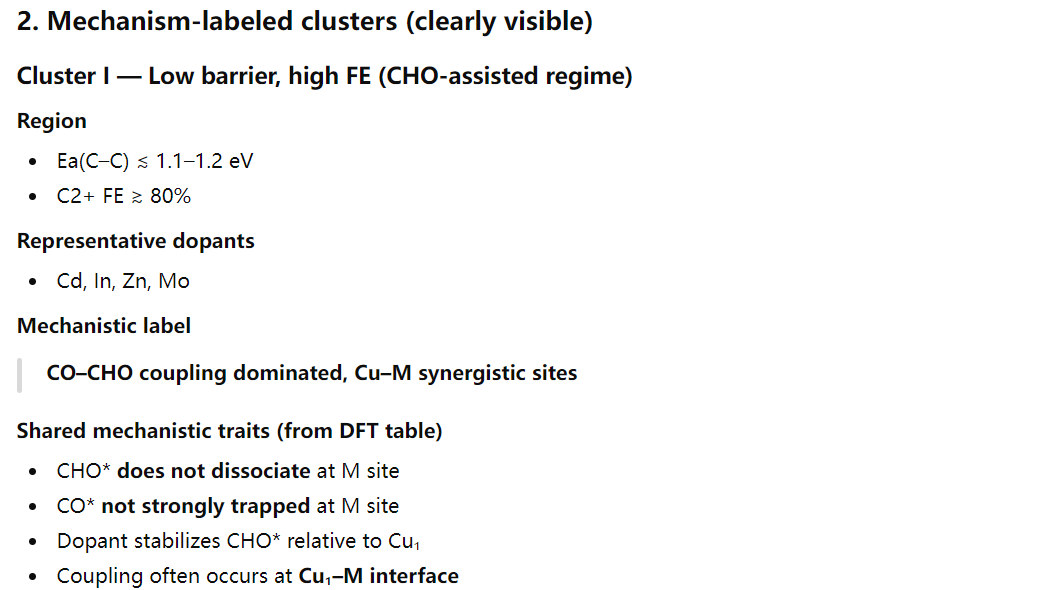

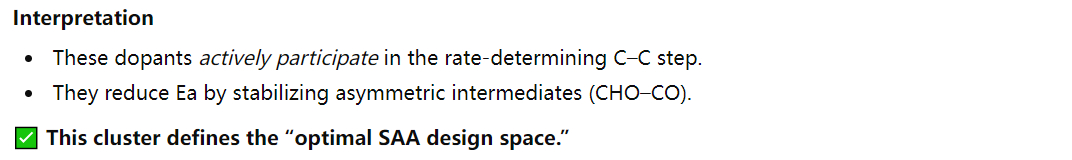

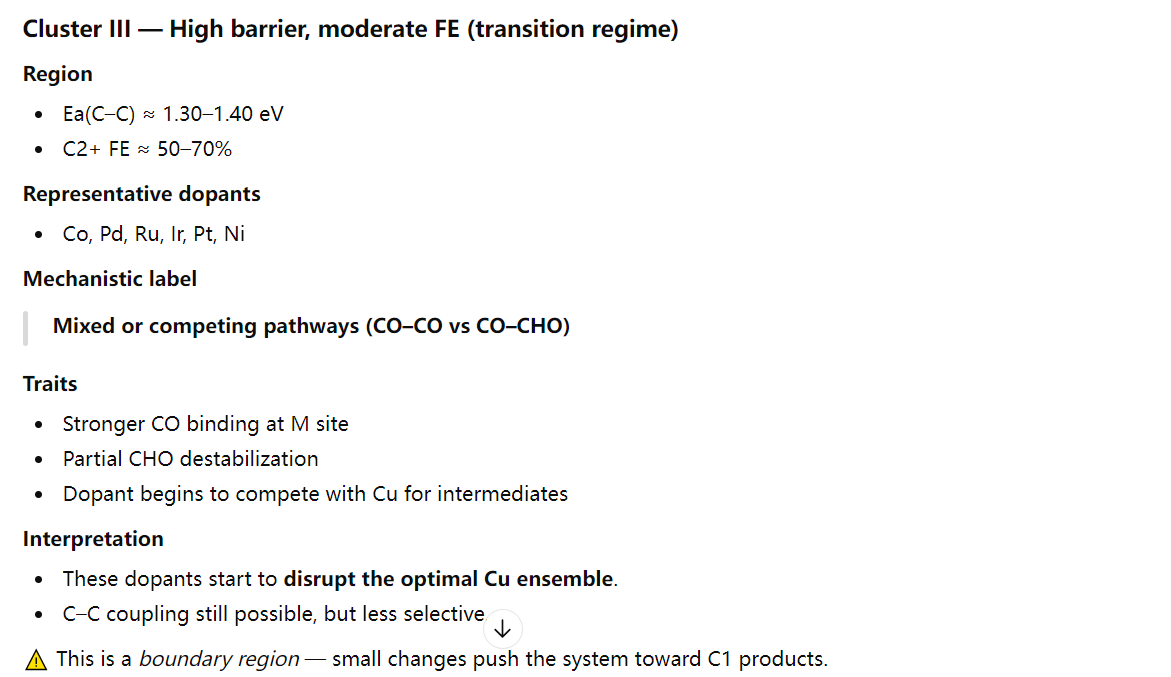

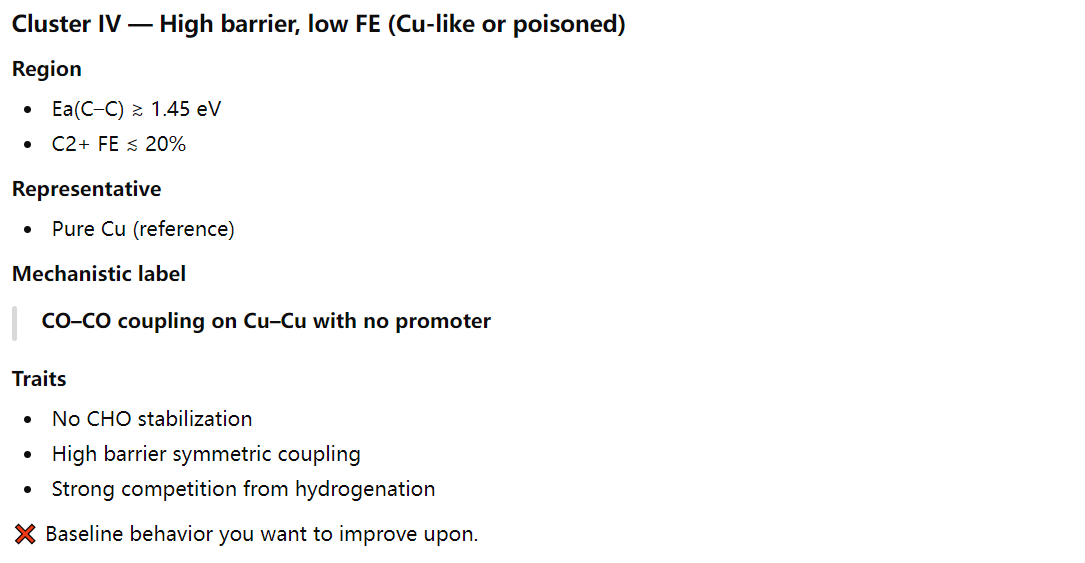

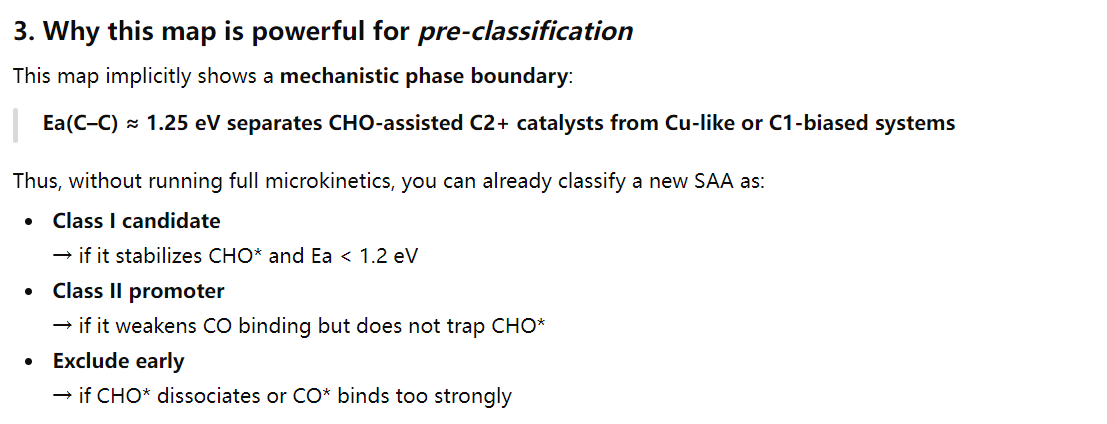

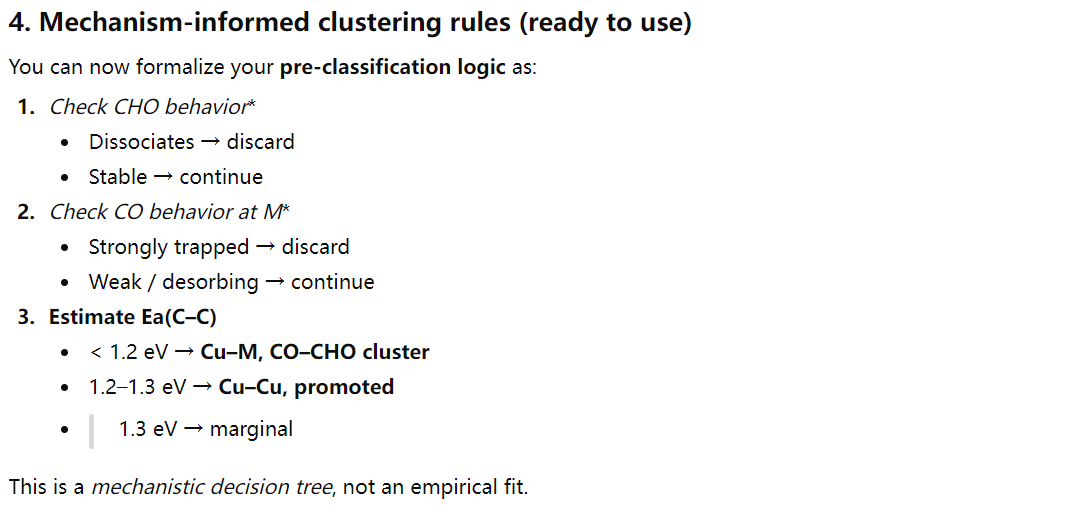

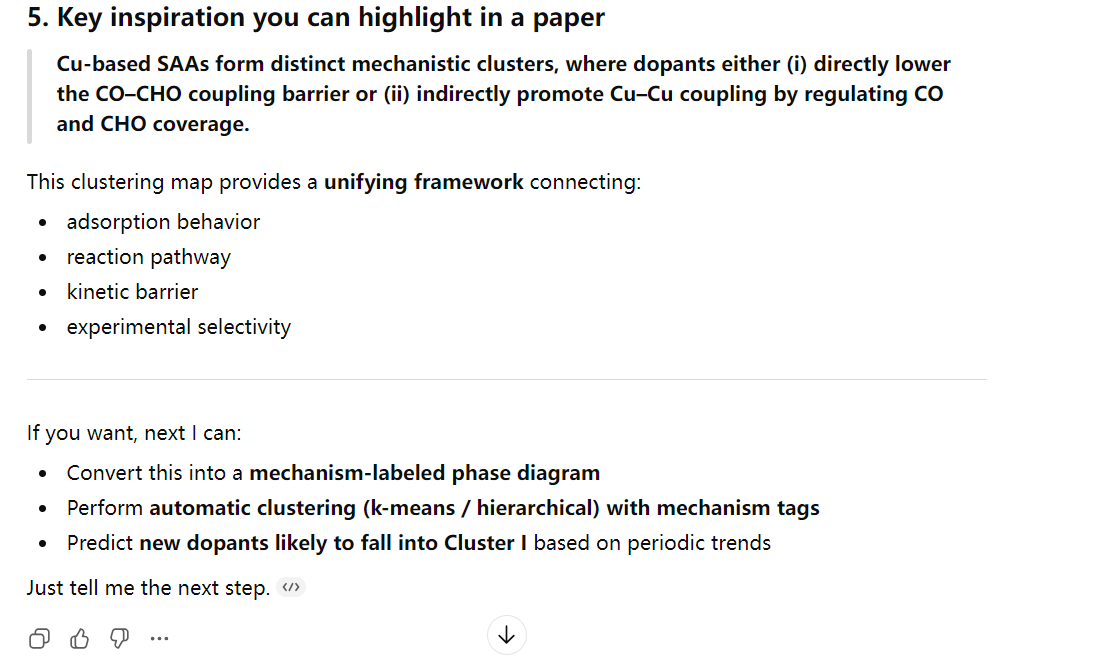


**Part 2 Supplementary Figures**


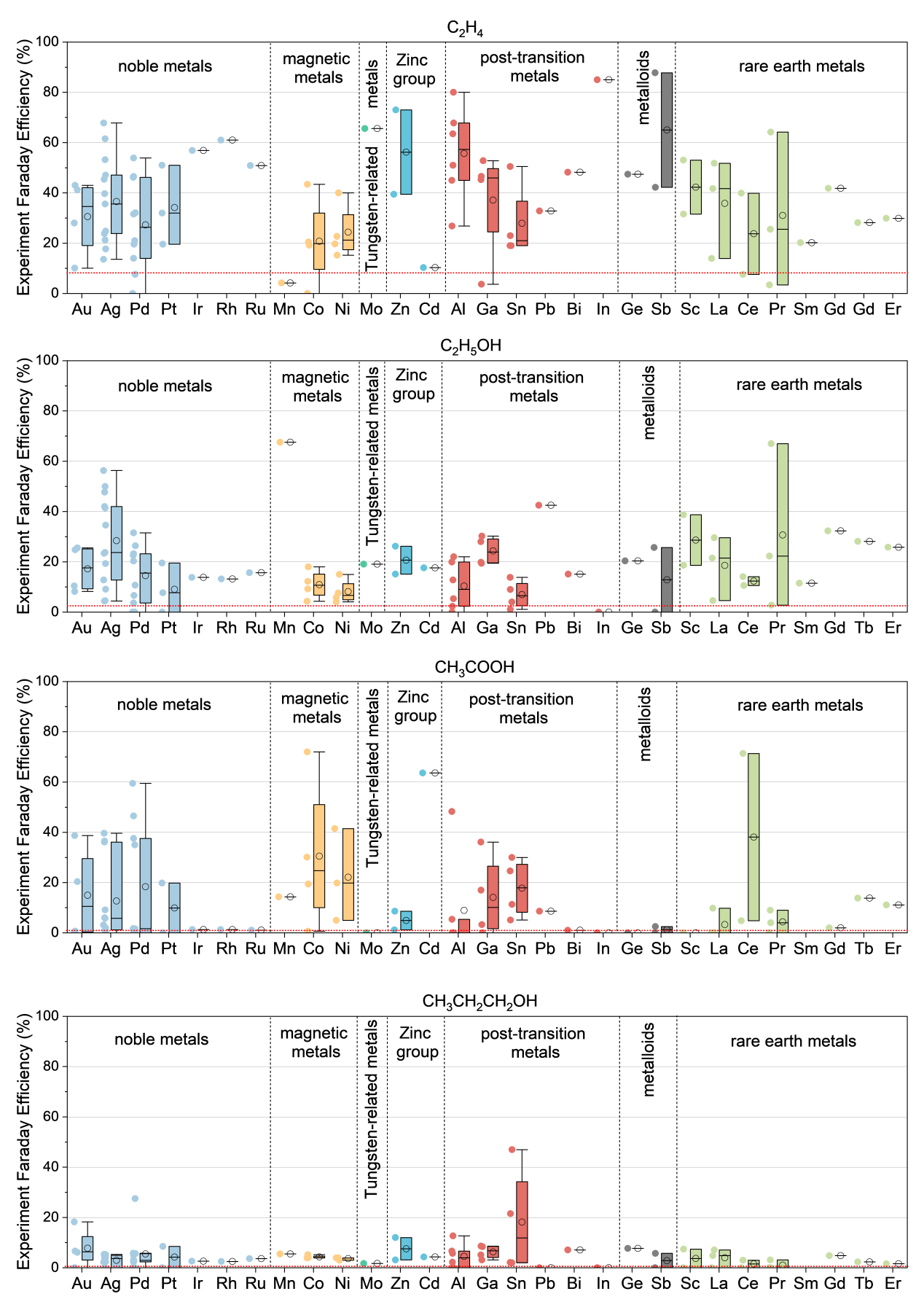


**Figure S1.** Summary of experimental Faraday efficiency of multi-carbon products (C_2+_) from CO_2_RR over Cu-based SAAs from the Digital Catalysis Platform (DigCat). (a) Ethylene (C_2_H_4_). (b) Ethanol (C_2_H_5_OH). (c) Acetic acid (CH_3_COOH). (d) n-Propanol (CH_3_CH_2_CH_2_OH).


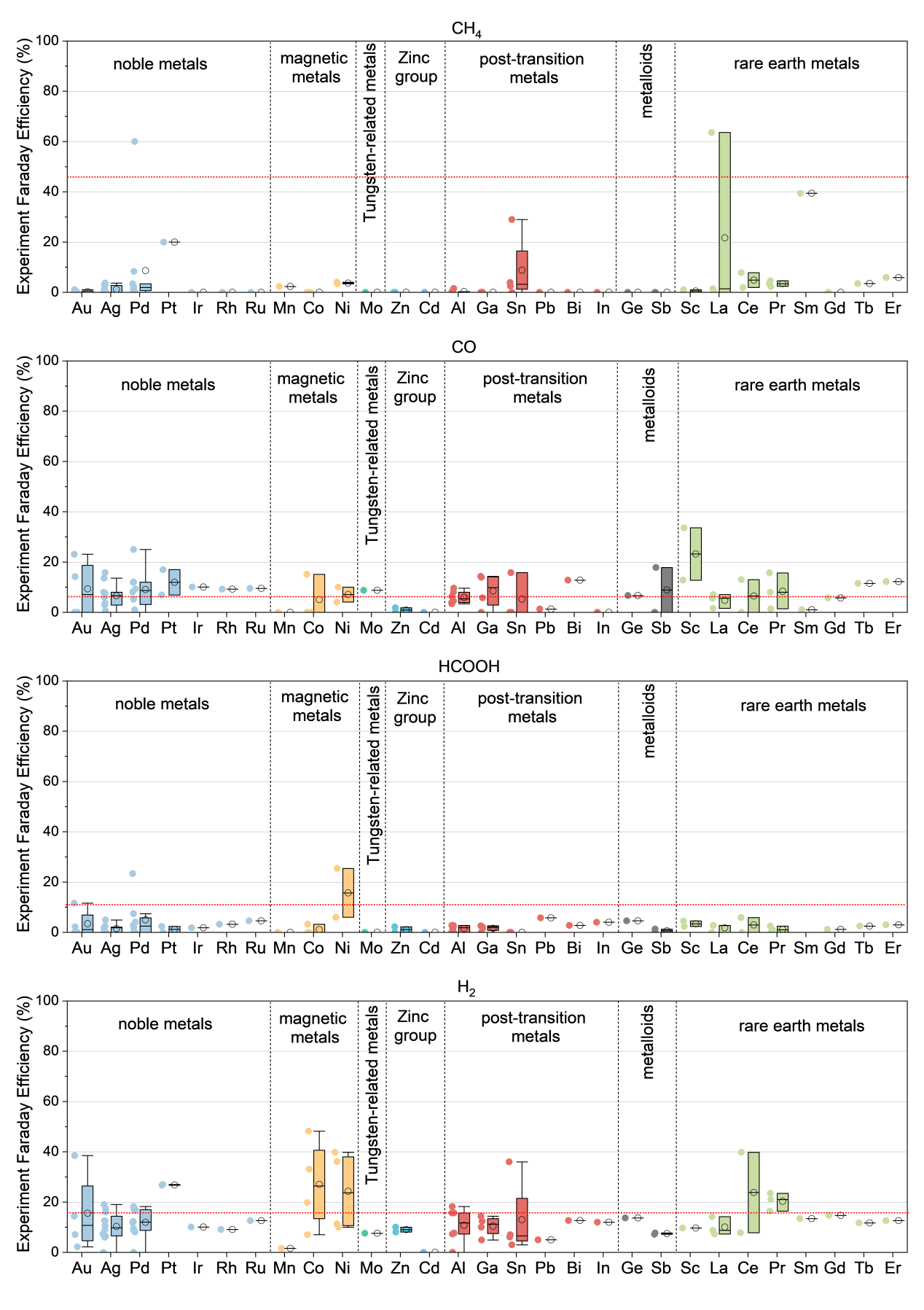


**Figure S2.** Summary of experimental Faraday efficiency of C_1_ products from CO_2_RR and H_2_ from HER over Cu-based SAAs from the Digital Catalysis Platform (DigCat). (a) Methane (CH_4_). (b) Carbon monoxide (CO). (c) Formic acid (HCOOH). (d) Hydrogen (H_2_).


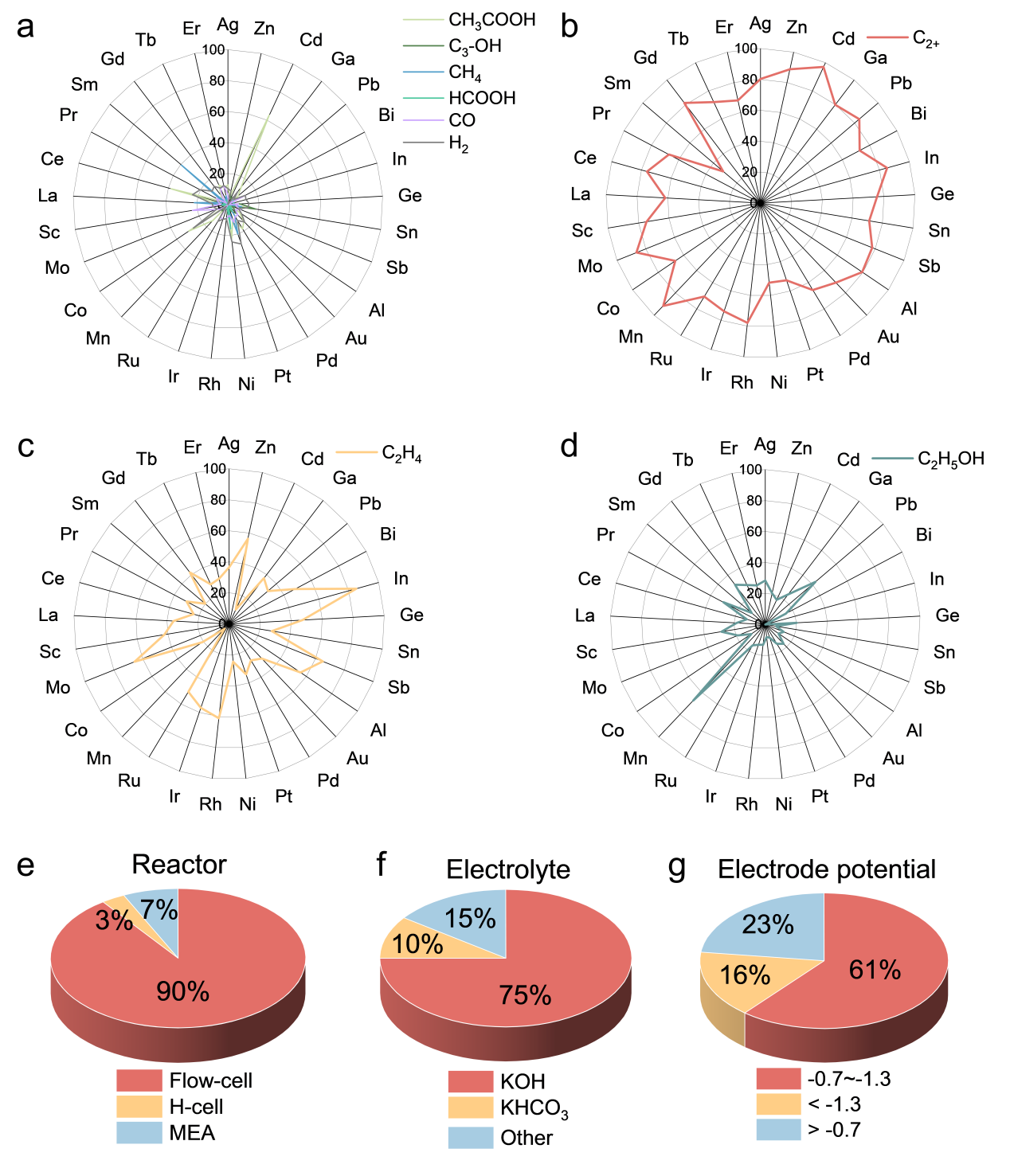


**Figure S3.** Radar chart of experimental Faraday efficiency of CO_2_RR main products, including (a) secondary products, (b) C_2+_, (c) C_2_H_4_, and (d) C_2_H_5_OH. Statistical analysis of the selected experimental data samples in the Digcat platform: (e) Reactor, (f) Electrolyte, and (g) Electrode potential (V vs. RHE).


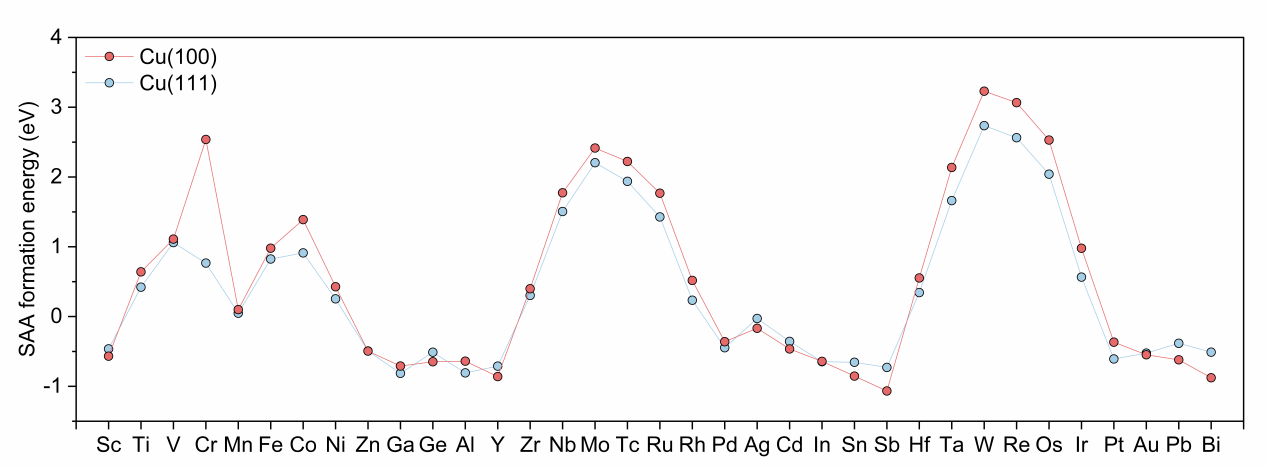


**Figure S4.** The calculated formation energies of Cu-based SAAs based on Cu(111) or Cu(100) facet.

**Figure S5.** The calculated (a) formation energies, (b) segregation energies and (c) aggregation energies of Cu-based single-atom alloys (SAAs).


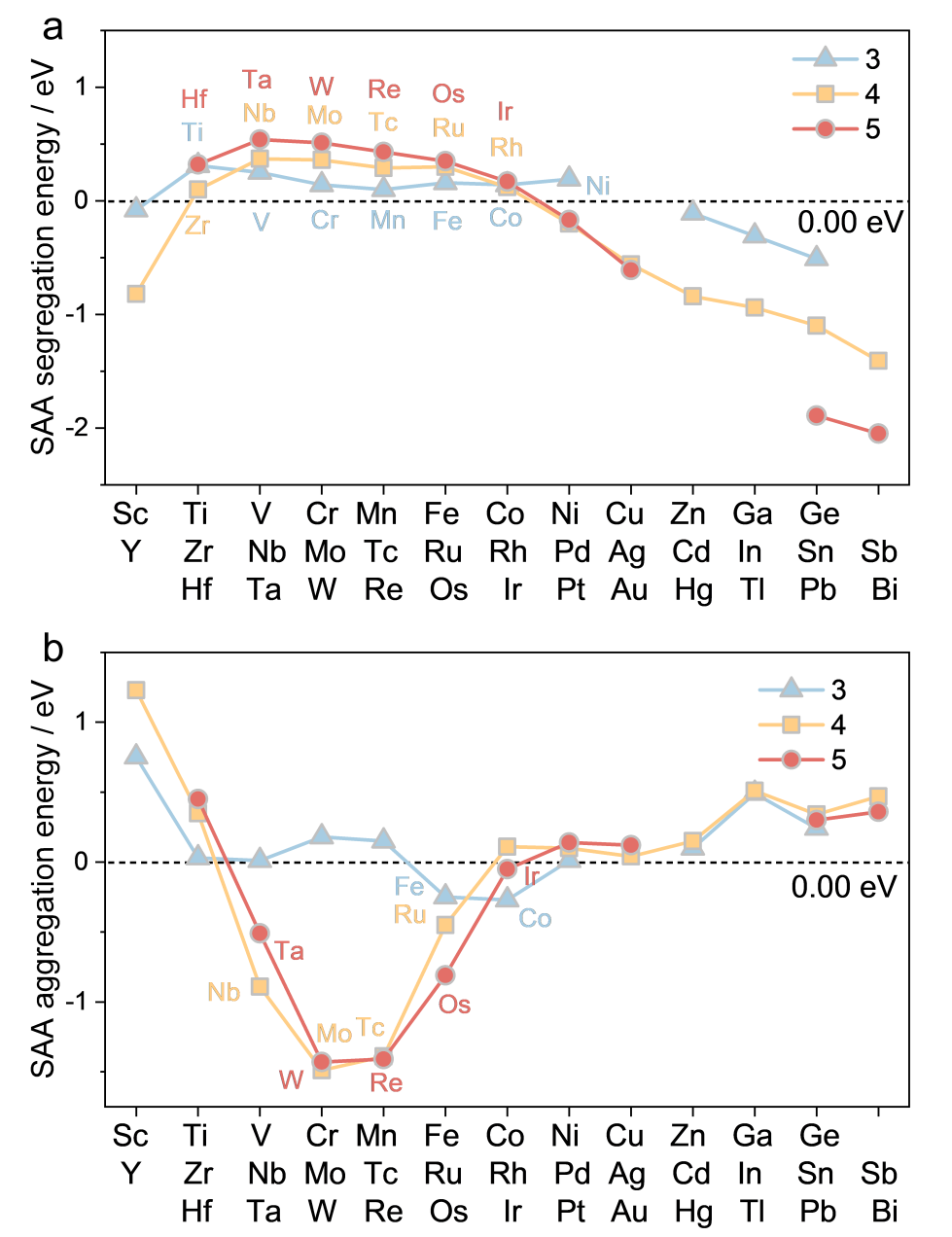


**Figure S6.** The calculated (a) segregation energies and (b) aggregation energies of Cu-based SAAs. The blue, yellow and red dots respectively represent the elements of the third, fourth and fifth periods in the periodic table.


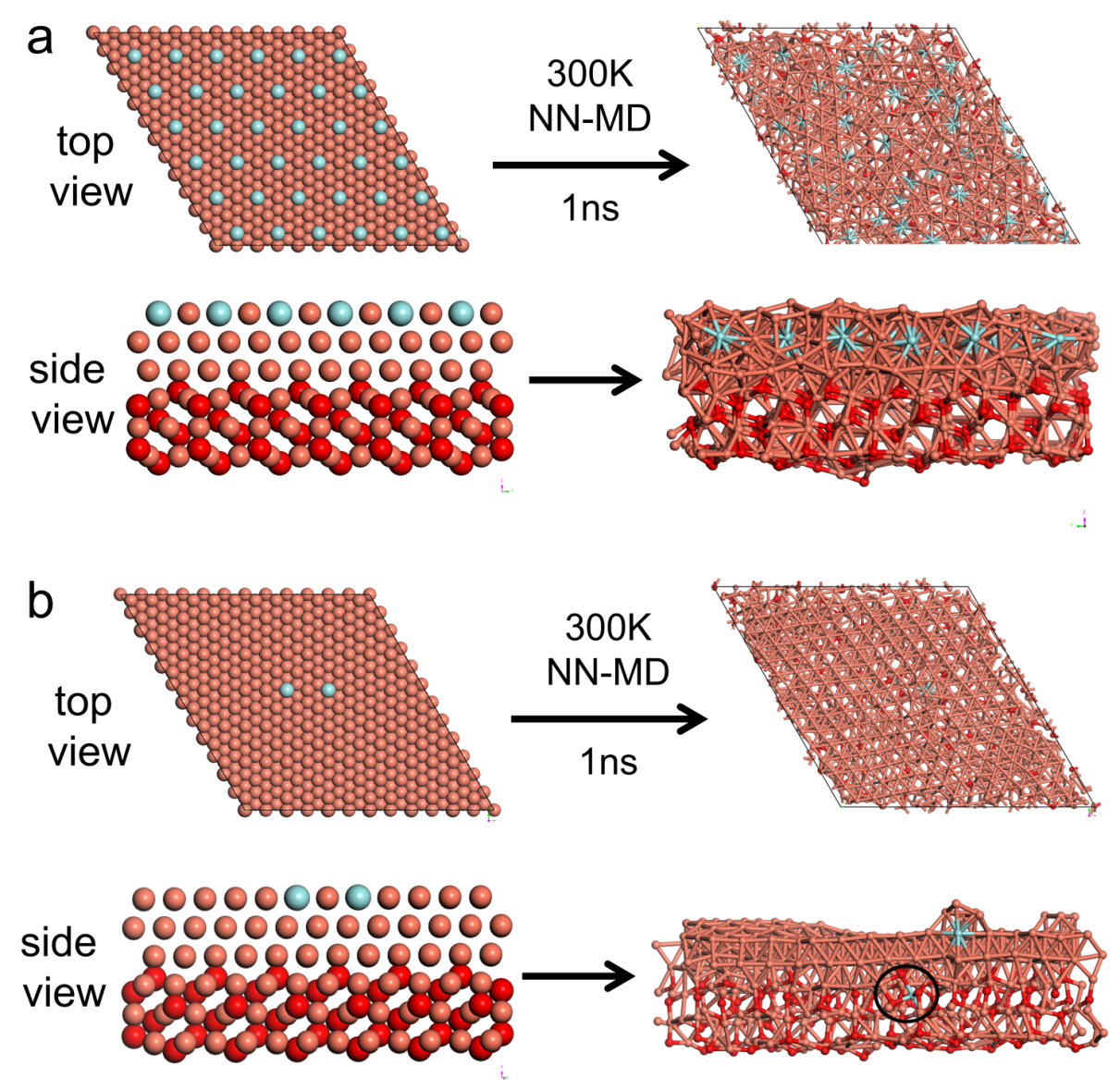


**Figure S7.** The initial and final states of Zr_1/_Cu SAA in the NN-MD simulation of 1ns. (a) Zr_36_/Cu (36 Zr atoms). (b) Zr_2_/Cu (2 Zr atoms). A partially reduced Cu_2_O(111) plane was employed as the simulated single-atom carrier to simulate residual oxygen species in pre-catalyst systems. The partially reduced Cu_2_O(111) model was constructed by removing half of the oxygen atoms from a 6-layer, 12x12 Cu_2_O(111) surface model. We constructed a Zr_1_/Cu(111) SAA model with moderate density (36 dopant atoms is uniformly distributed across the Cu surface) on the partially reduced Cu_2_O(111) surface and performed a 1 ns NN-MD simulation at a temperature of 300 K. To expedite the simulation process, we developed a low-density Zr_1_/Cu(111) SAA model (**Figure S7b**) and assessed its stability under identical MD conditions. The results demonstrated that one Zr atom stabilized near the surface, while another Zr atom migrated to a deeper subsurface region and coordinated with an O atoms after the simulation.


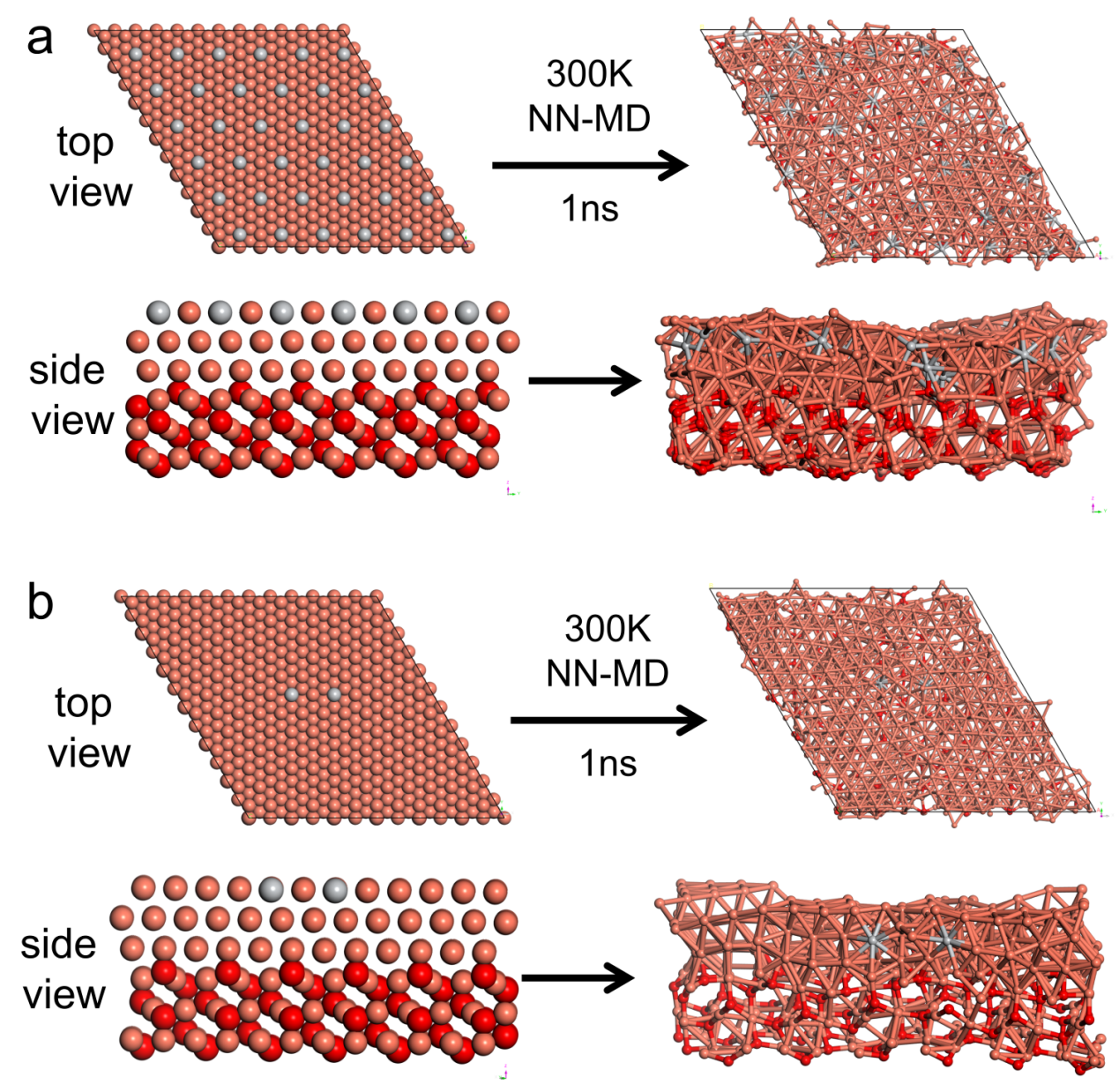


**Figure S8.** The initial and final states of Ti_1/_Cu SAA in the NN-MD simulation of 1ns. (a) Ti_36_/Cu (36 Ti atoms). (b) Ti_2_/Cu (2 Ti atoms).


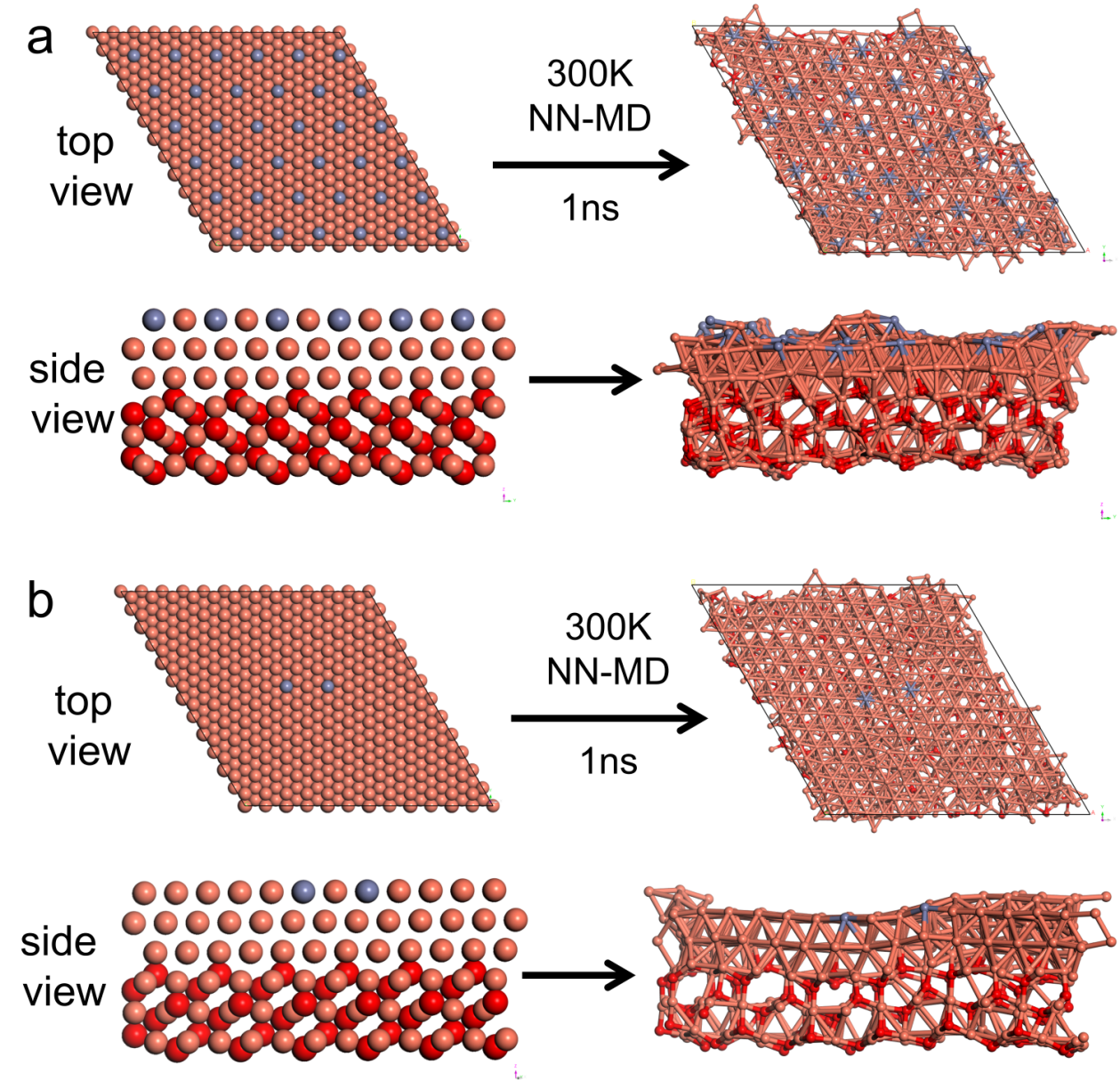


**Figure S9.** The initial and final states of Zn_1/_Cu SAA in the NN-MD simulation of 1ns. (a) Zn_36_/Cu (36 Zn atoms). (b) Zn_2_/Cu (2 Zn atoms).


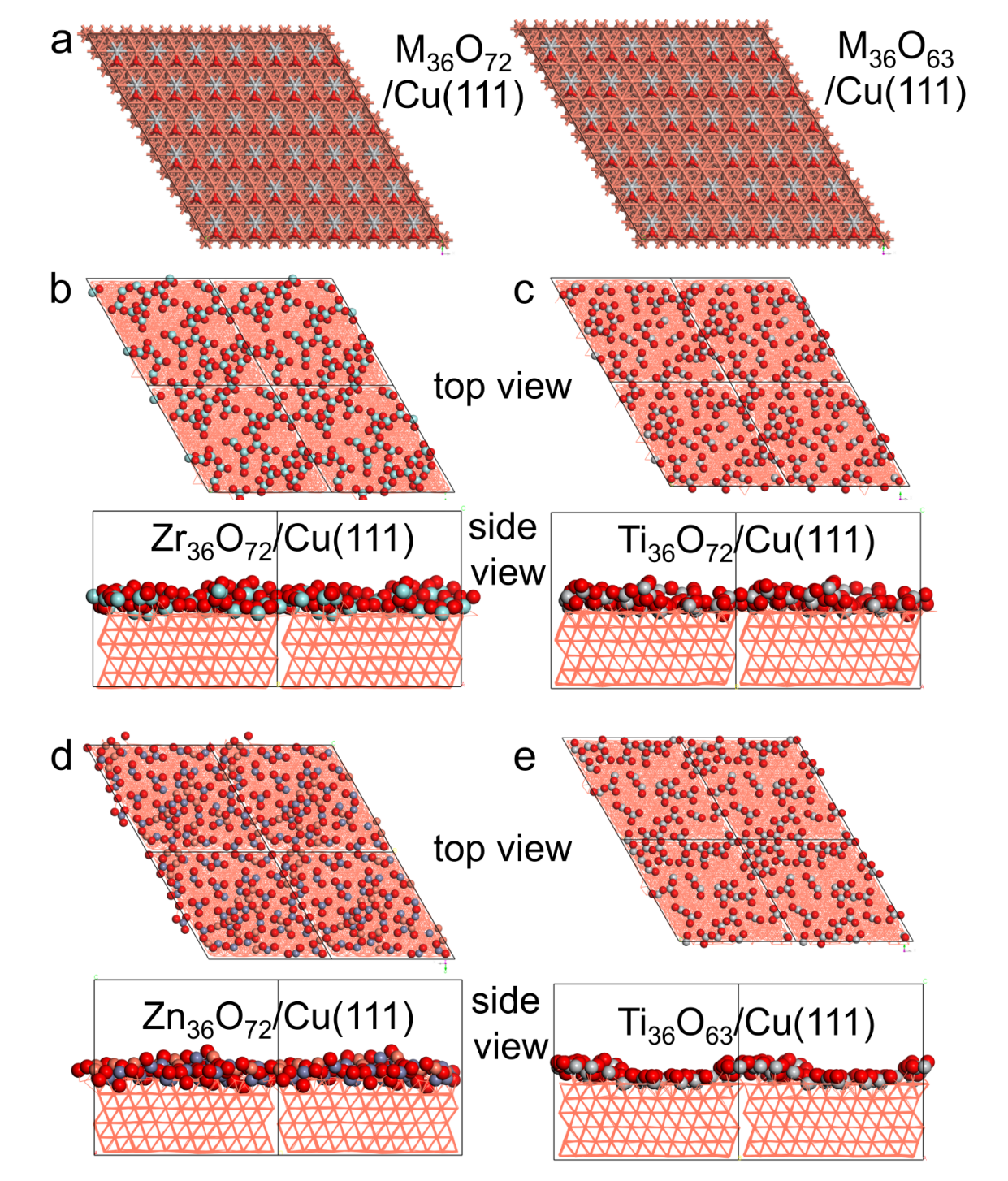


**Figure S10.** The initial and final states during the surface oxidation process of SAAs during the 1ns NN-MD simulation. Initial states: (a) M_36_/Cu(111)-72O and M_36_/Cu(111)-63O. Final states: (b) Zr_36_/Cu(111)-72O*. (c) Ti_36_/Cu(111)-72O*. (d) Zn_36_/Cu(111)-72O*. (e) Ti_36_/Cu(111)-63O*.


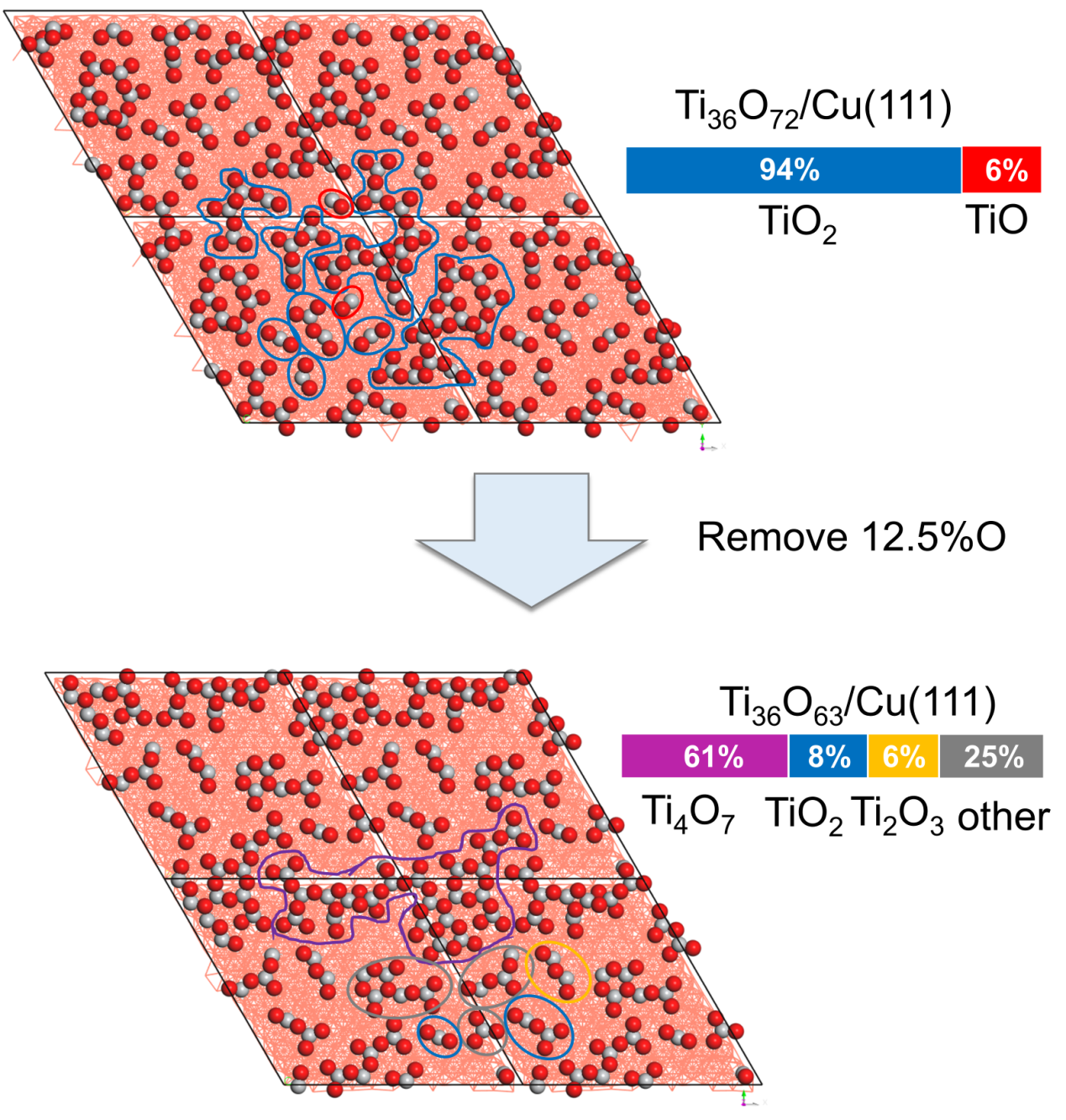


**Figure S11.** The types and proportions of TiO_x_ clusters in the oxidized process of the Ti_1_/Cu(111) SAA surface during the 1ns NN-MD simulation. (a) Ti_36_/Cu(111)-72O*. (b) Ti_36_/Cu(111)-63O*.


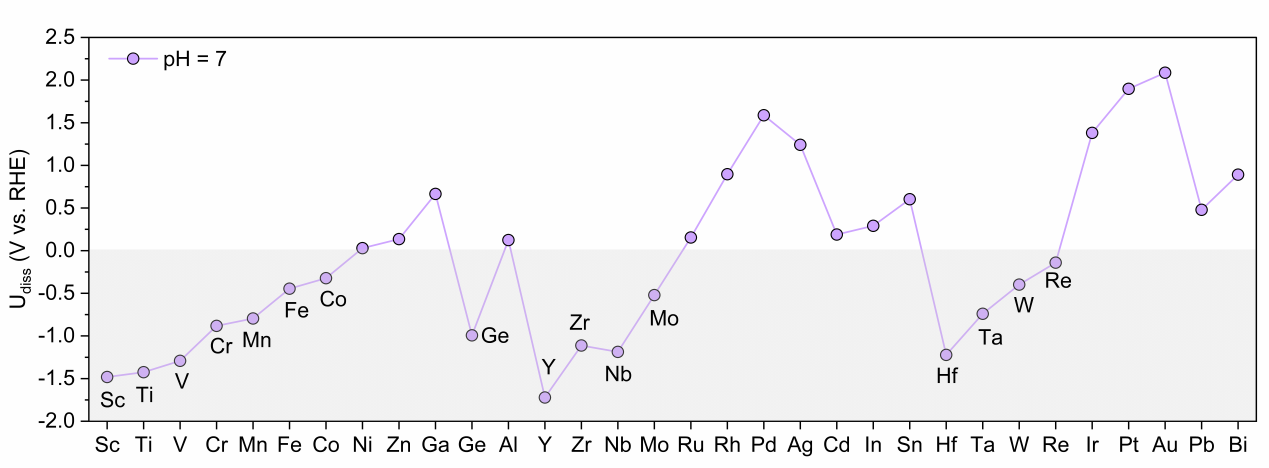


**Figure S12.** The dissolution potential (U_diss_, V vs. RHE) for M in M_1_/Cu(111) at pH = 7.


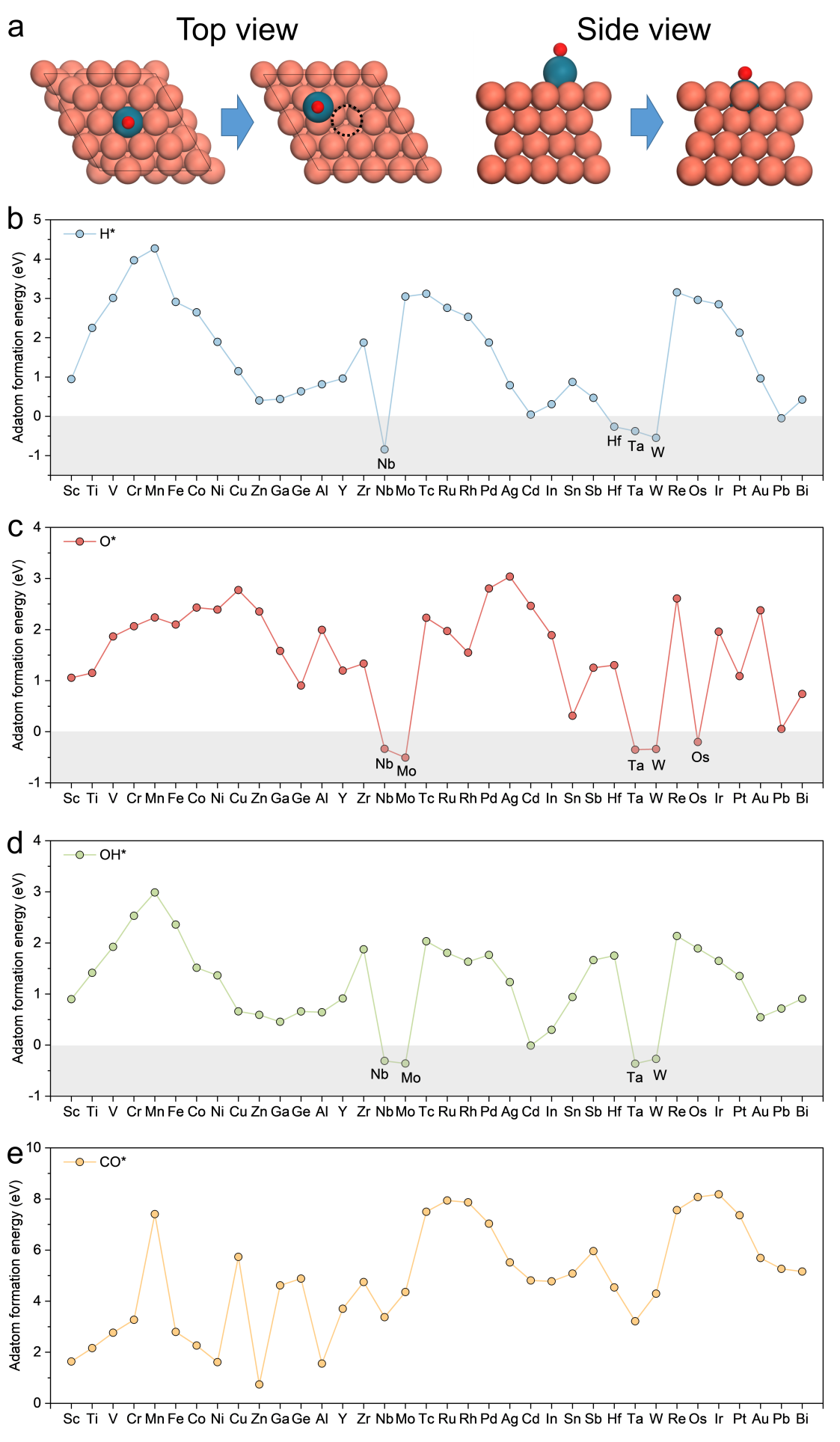


**Figure S13.** The calculation process (a) and results of the formation energy of adatom induced by (b) H*, (c) O*, (d) OH*, and (e) CO*.


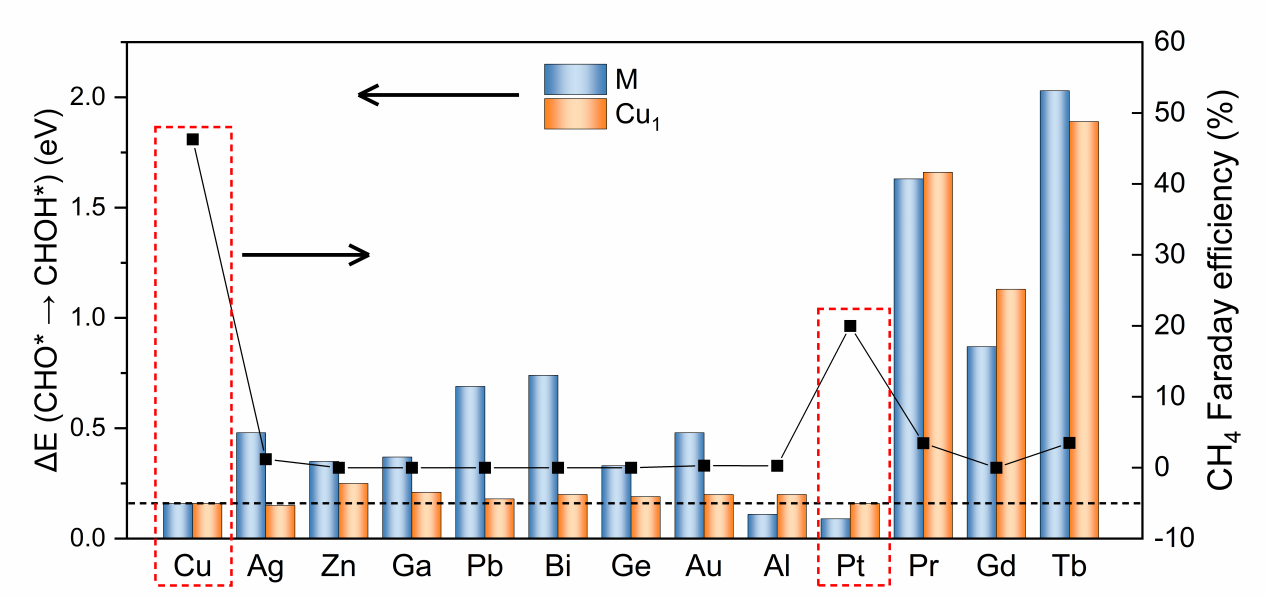


**Figure S14.** The thermodynamic reaction energies for CHO* hydrogenation to CHOH* on Cu-based SAAs and their CH_4_ experimental Faraday efficiency from the Digital Catalysis Platform (DigCat).


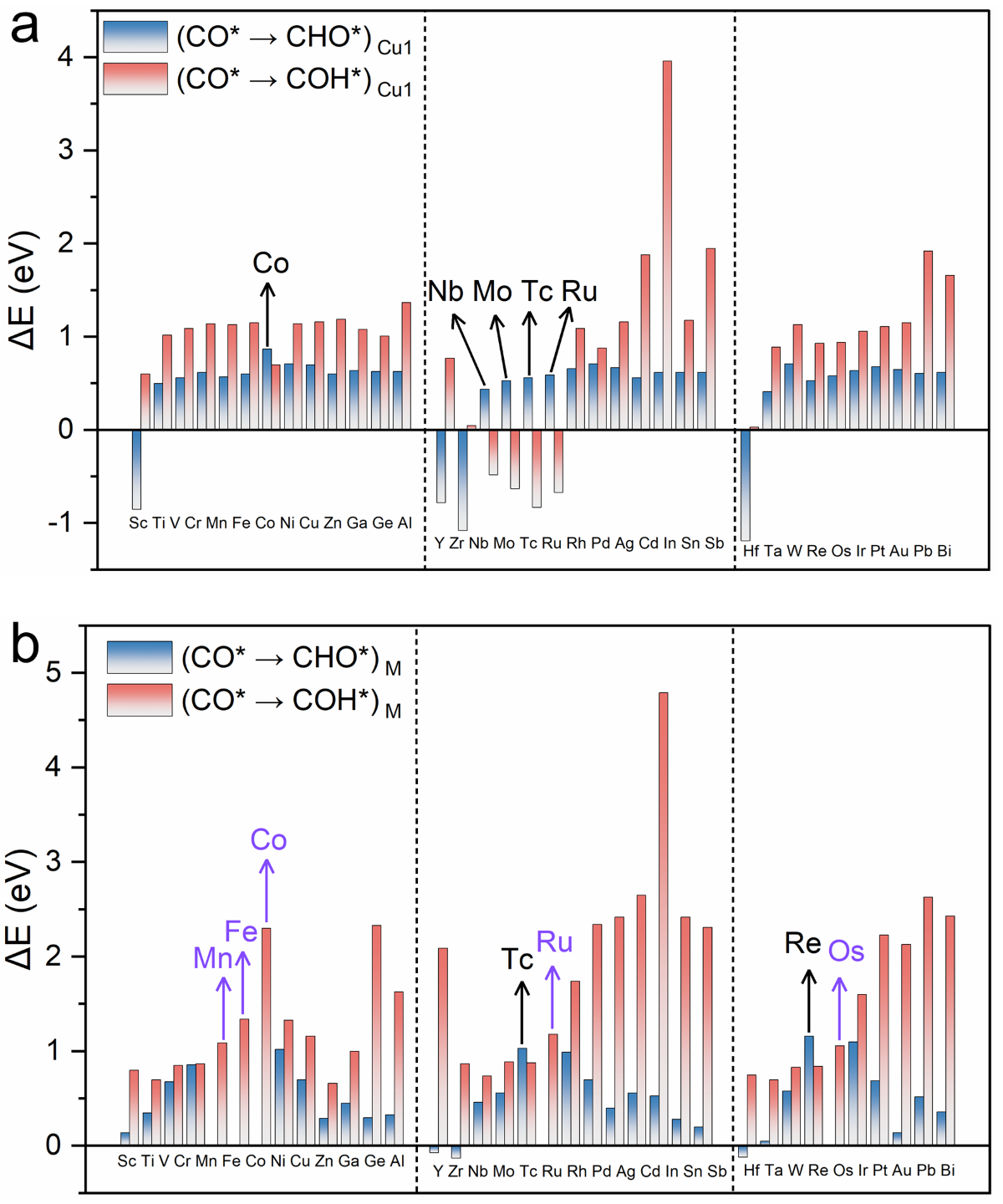


**Figure S15.** Thermodynamic reaction energies (ΔE) of CO* to CHO* or COH* at (a) Cu_1_ and (b) M sites. Elements marked in black signify that COH* is a preferred species. The elements in purple indicate that CHO* will undergo dissociative adsorption.


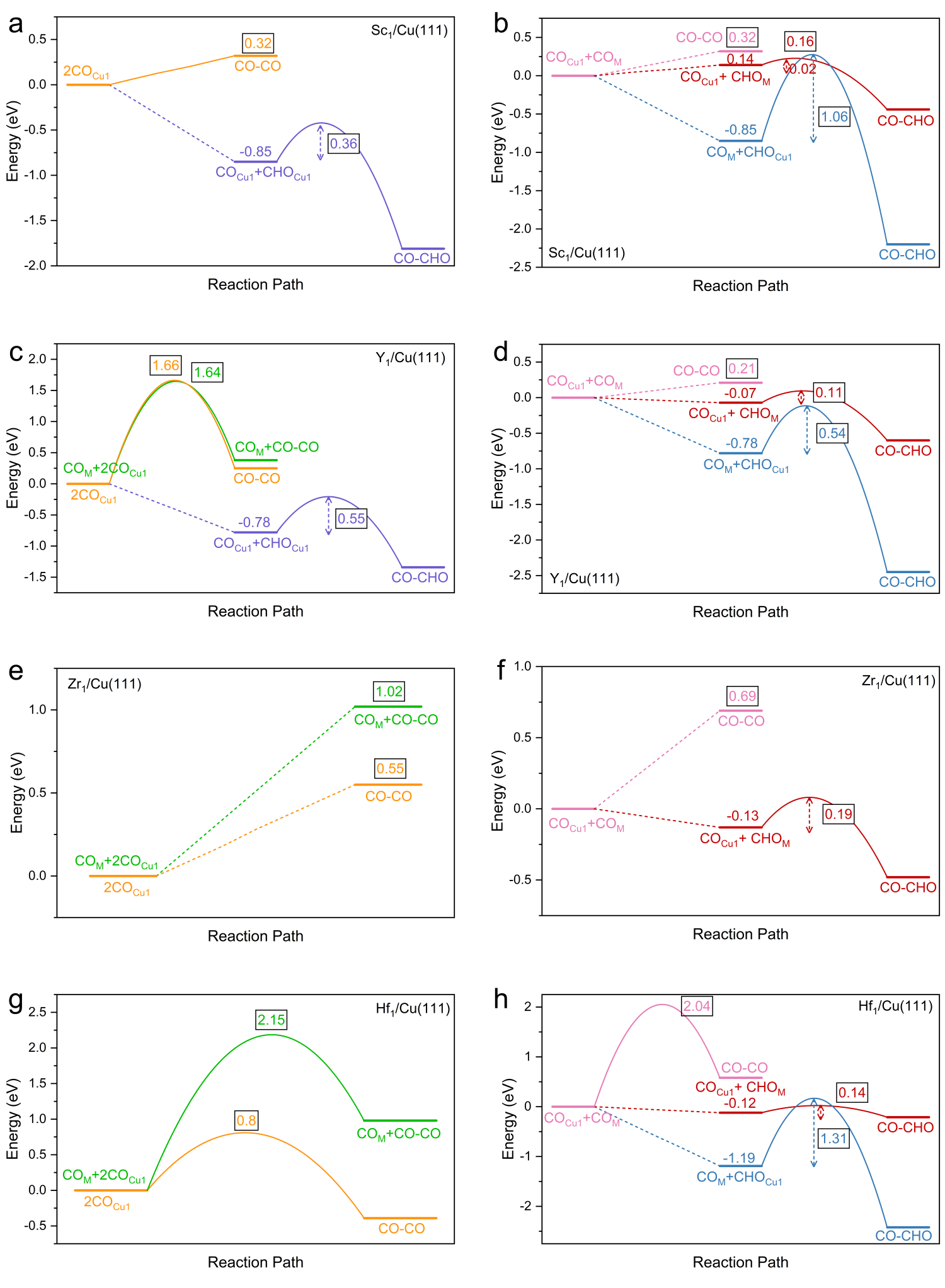


**Figure S16.** Reaction energy profiles for C-C coupling on Cu-based SAAs. (a~b) Sc_1_/Cu(111). (c~d) Y_1_/Cu(111). (e~f) Zr_1_/Cu(111). (g~h) Hf_1_/Cu(111).


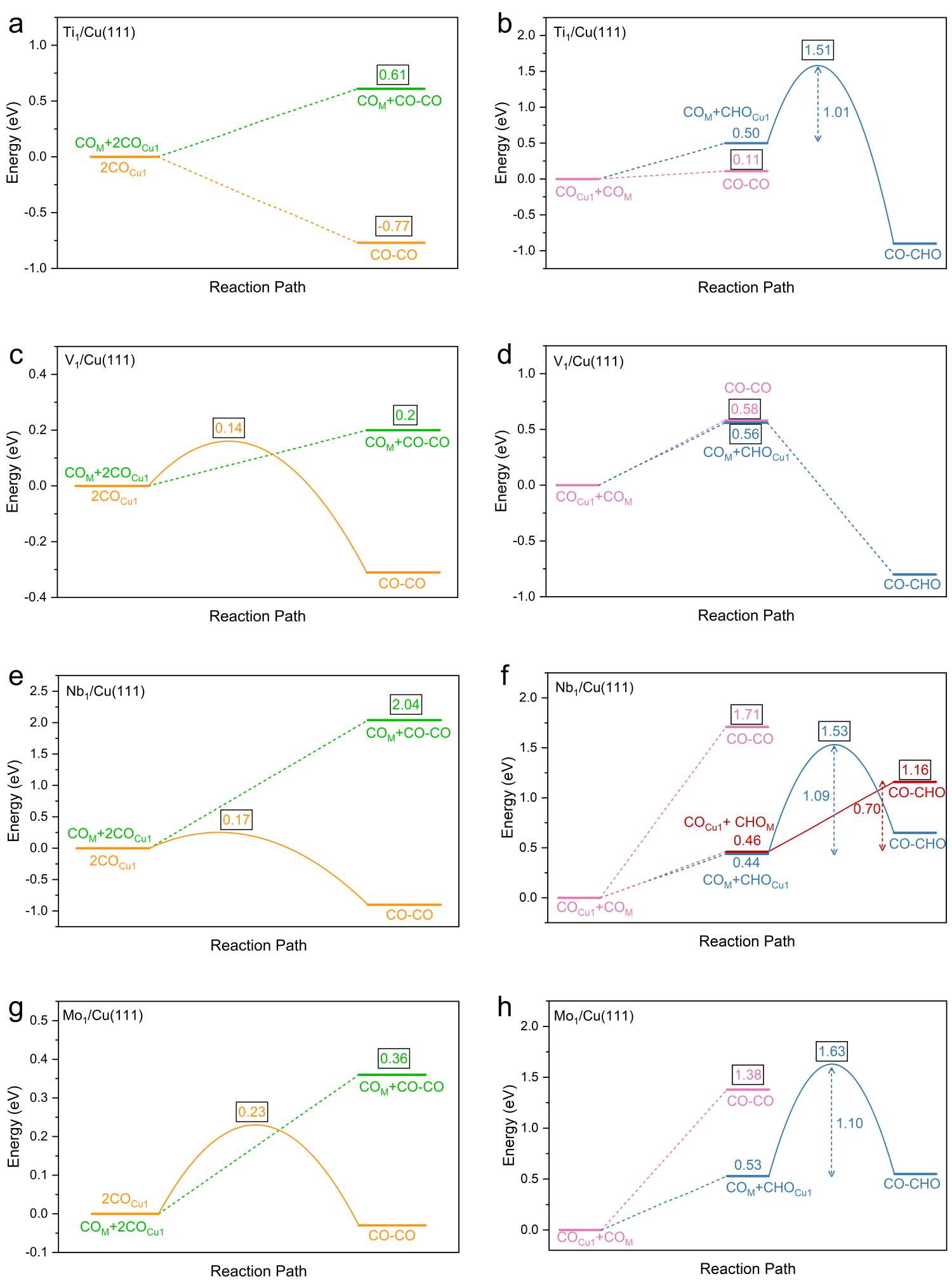


**Figure S17.** Reaction energy profiles for C-C coupling on Cu-based SAAs. (a~b) Ti_1_/Cu(111). (c~d) V_1_/Cu(111). (e~f) Nb_1_/Cu(111). (g~h) Mo_1_/Cu(111).


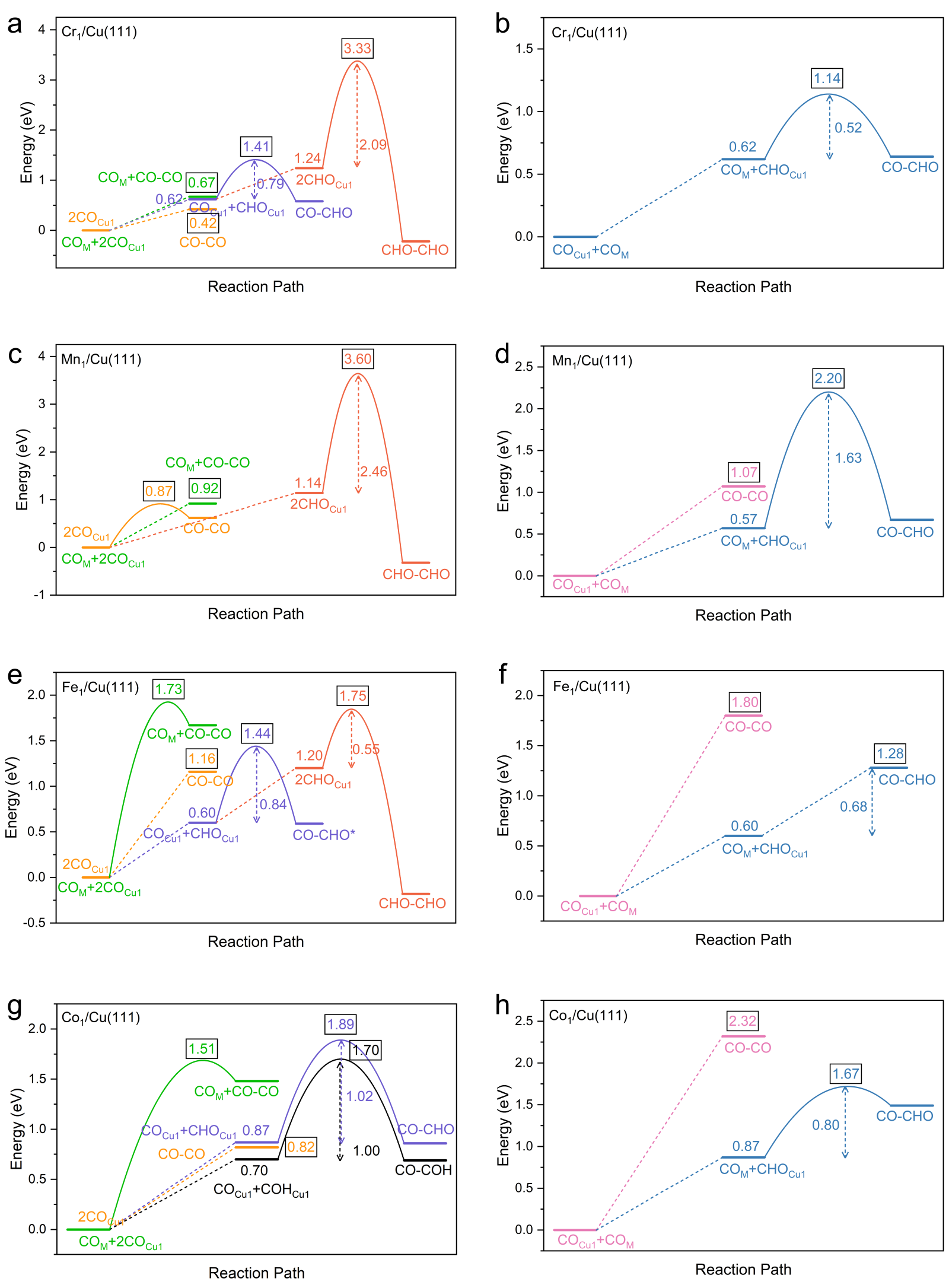


**Figure S18.** Reaction energy profiles for C-C coupling on Cu-based SAAs. (a~b) Cr_1_/Cu(111). (c~d) Mn_1_/Cu(111). (e~f) Fe_1_/Cu(111). (g~h) Co_1_/Cu(111).


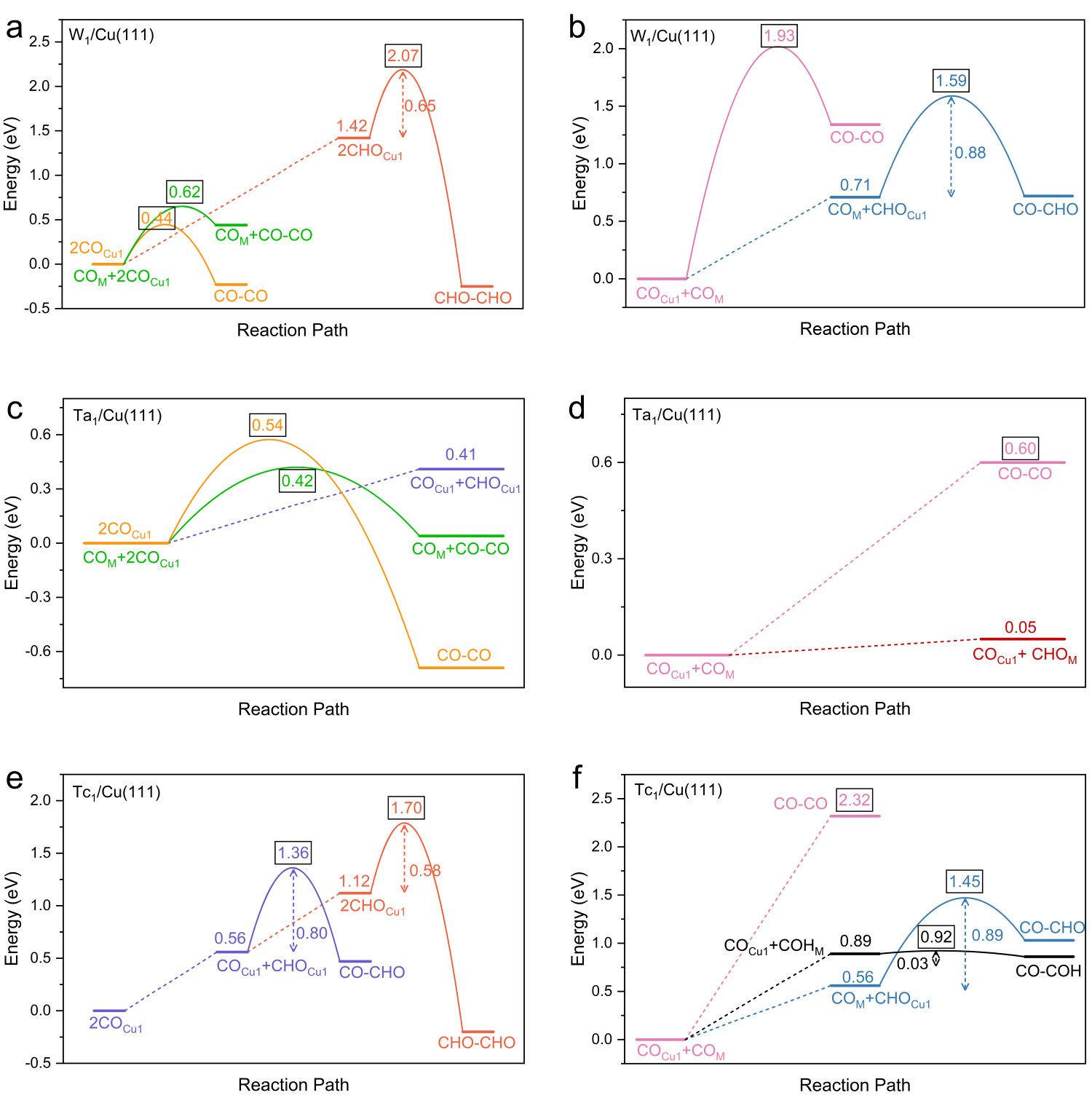


**Figure S19.** Free energy profile for C-C coupling on Cu-based SAAs. (a~b) W_1_/Cu(111). (c~d) Ta_1_/Cu(111). (e~f) Tc_1_/Cu(111).


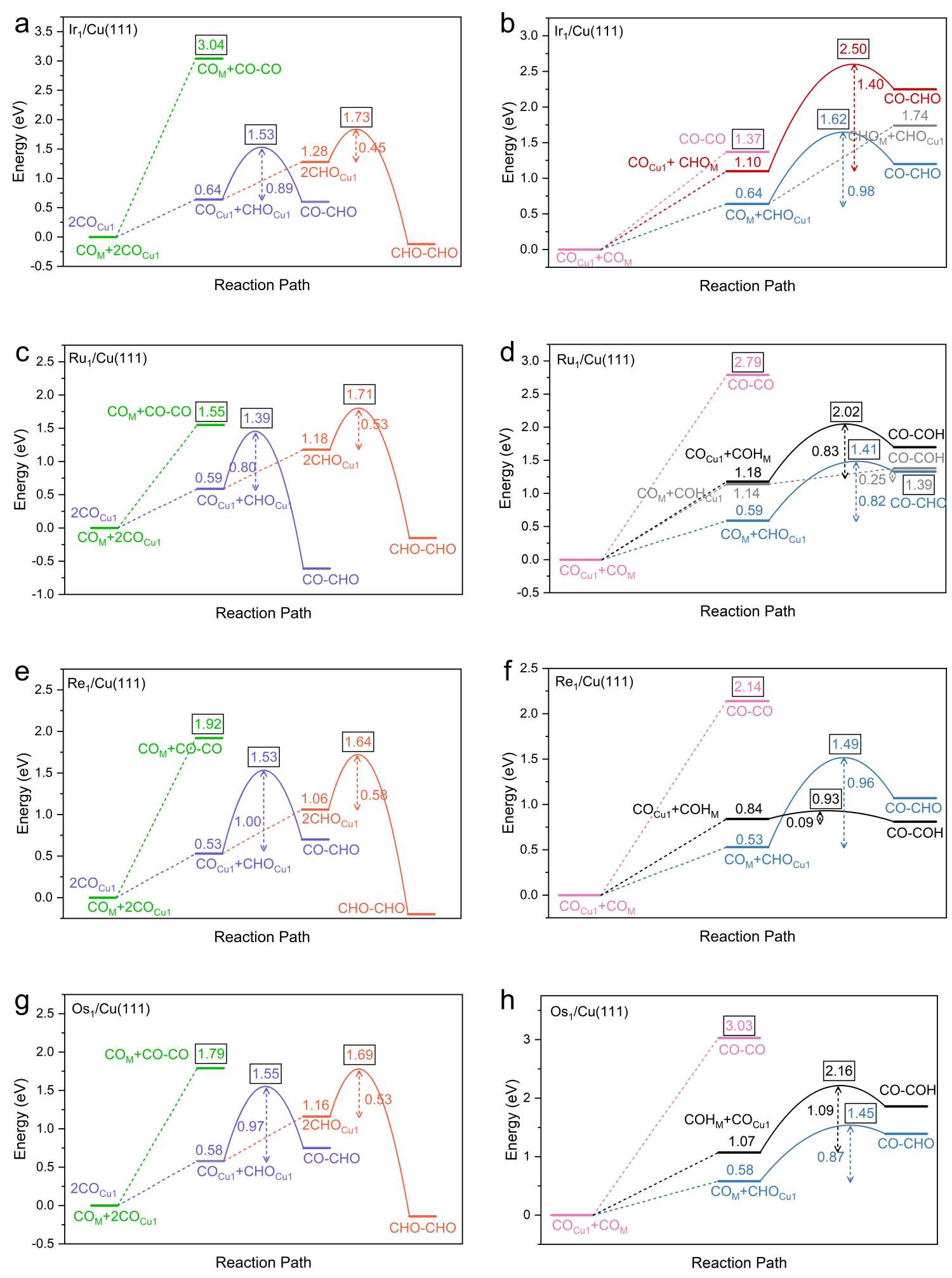


**Figure S20.** Reaction energy profiles for C-C coupling on Cu-based SAAs. (a~b) Ir_1_/Cu(111). (c~d) Ru_1_/Cu(111). (e~f) Re_1_/Cu(111). (g~h) Os_1_/Cu(111).


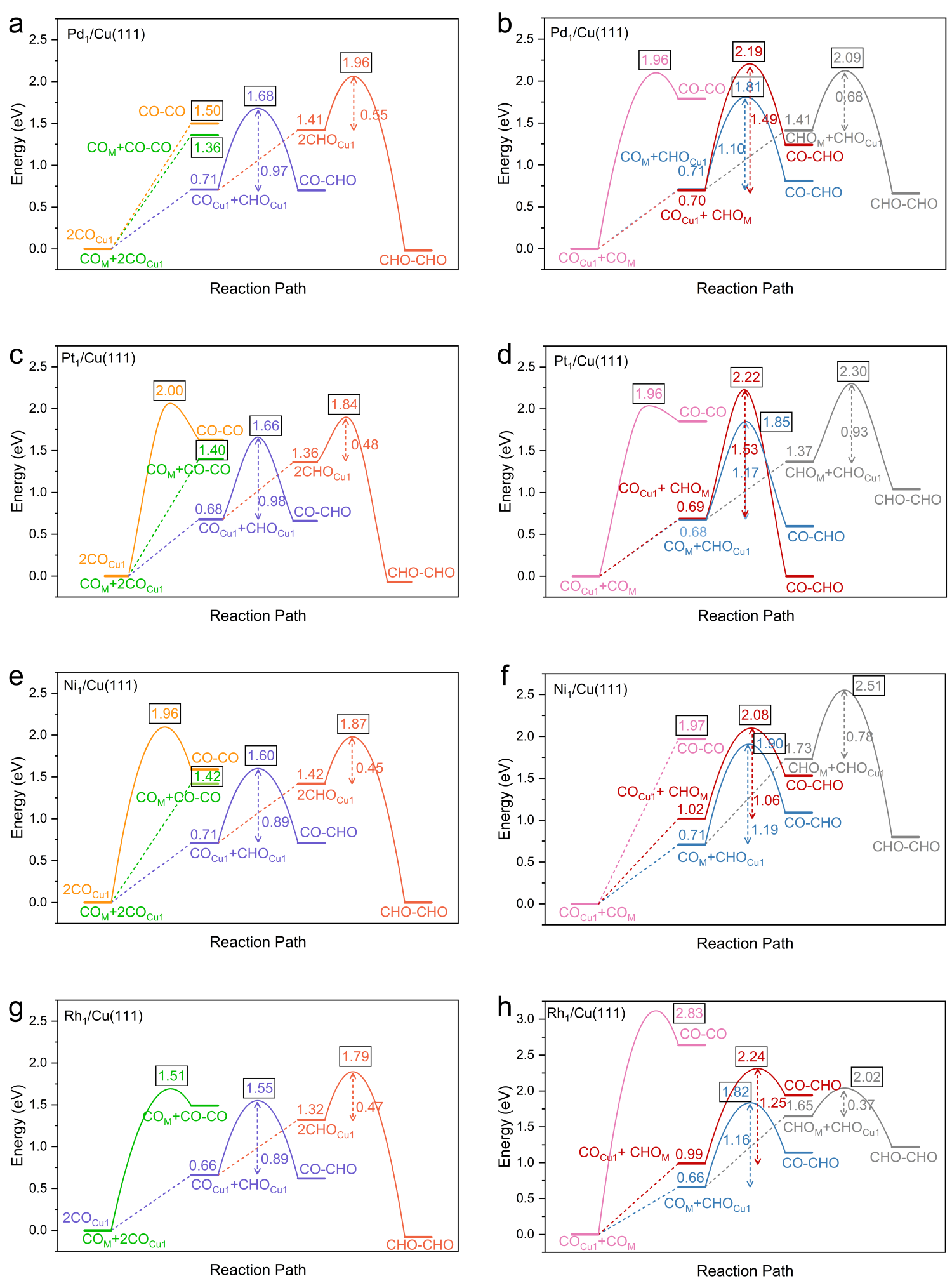


**Figure S21.** Reaction energy profiles for C-C coupling on Cu-based SAAs. (a~b) Pd_1_/Cu(111). (c~d) Pt_1_/Cu(111). (e~f) Ni_1_/Cu(111). (g~h) Rh_1_/Cu(111).


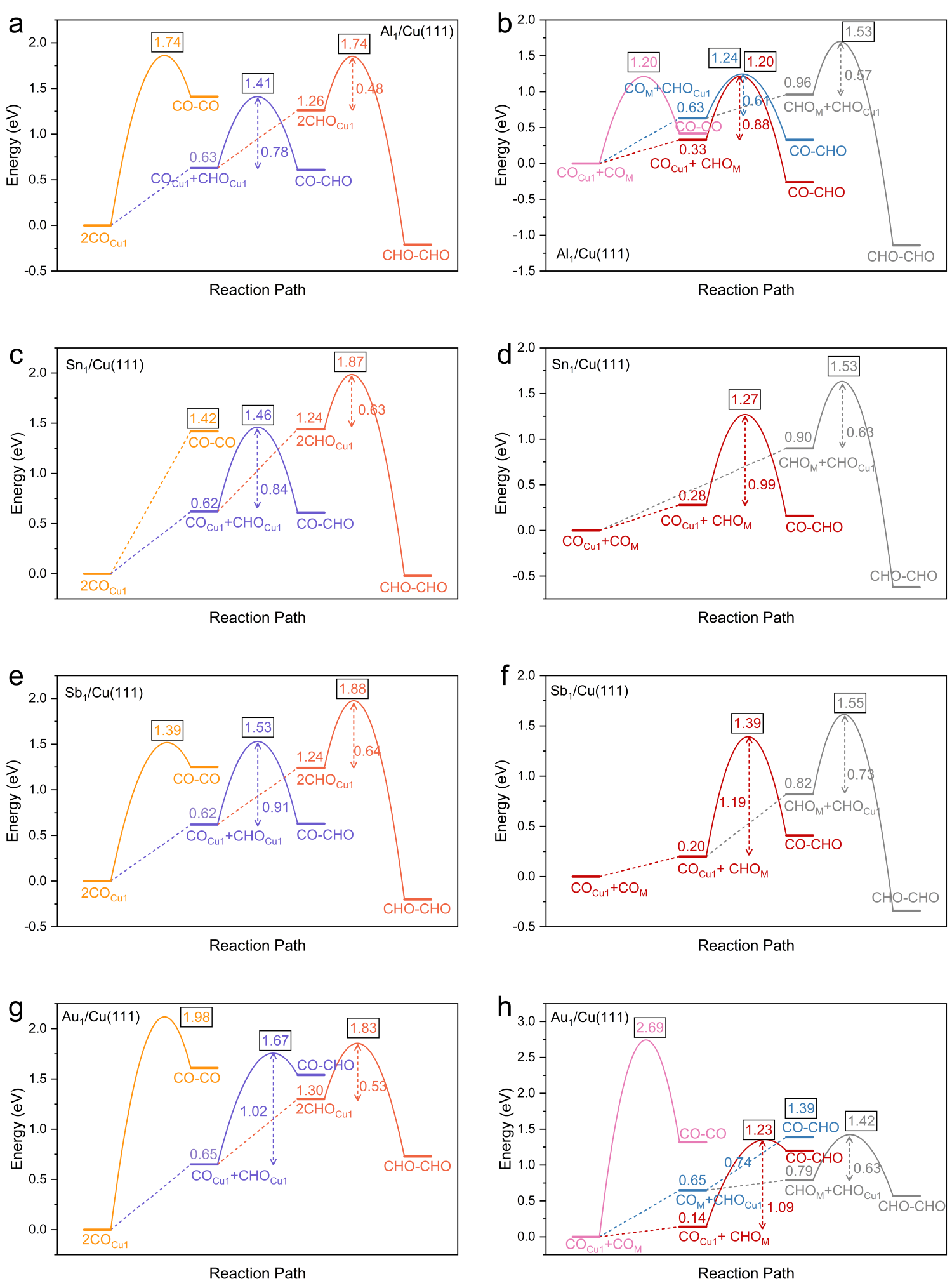


**Figure S22.** Reaction energy profiles for C-C coupling on Cu-based SAAs. (a~b) Al_1_/Cu(111). (c~d) Sn_1_/Cu(111). (e~f) Sb_1_/Cu(111). (g~h) Au_1_/Cu(111).


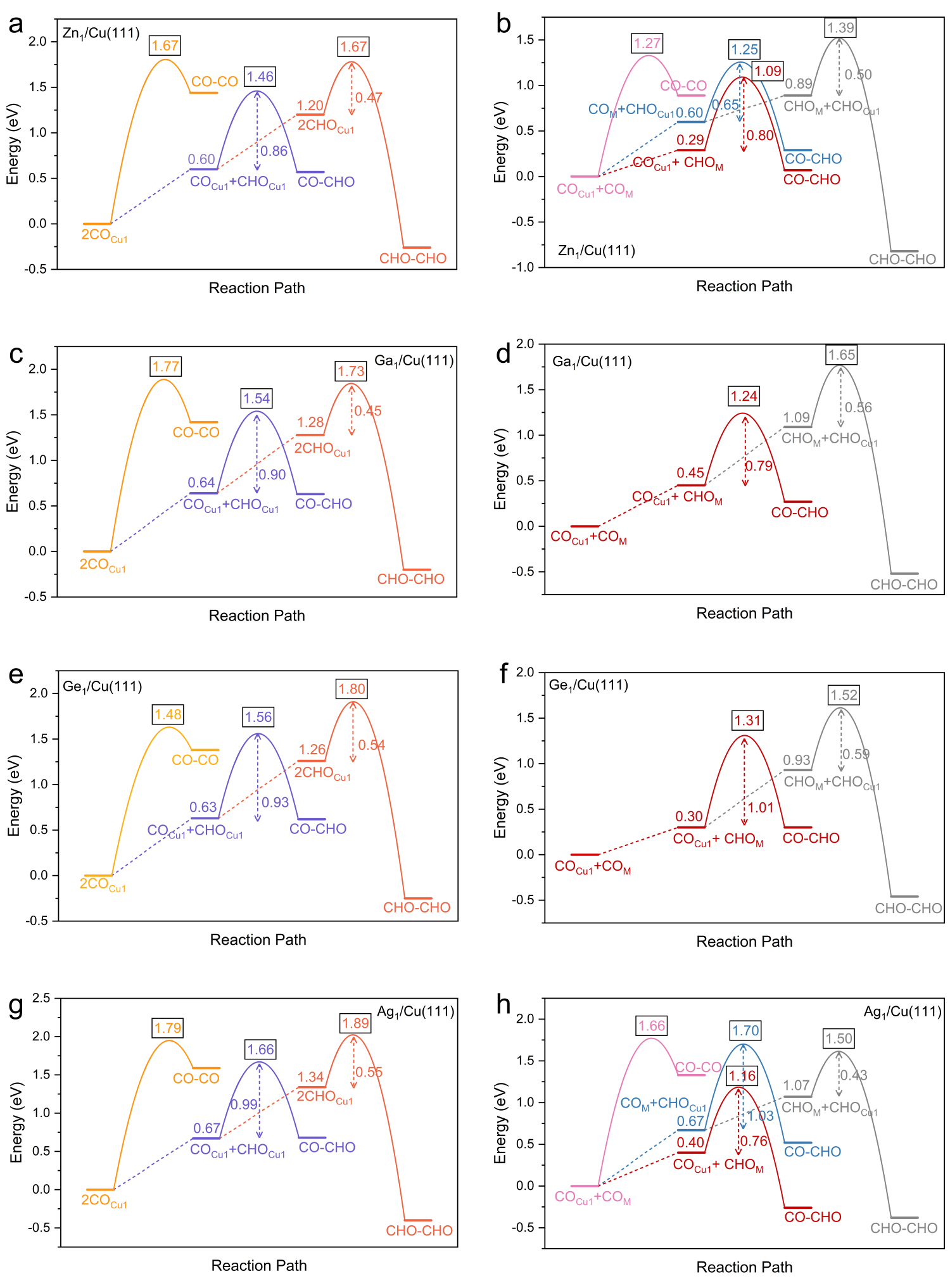


**Figure S23.** Reaction energy profiles for C-C coupling on Cu-based SAAs. (a~b) Zn_1_/Cu(111). (c~d) Ga_1_/Cu(111). (e~f) Ge_1_/Cu(111). (g~h) Ag_1_/Cu(111).


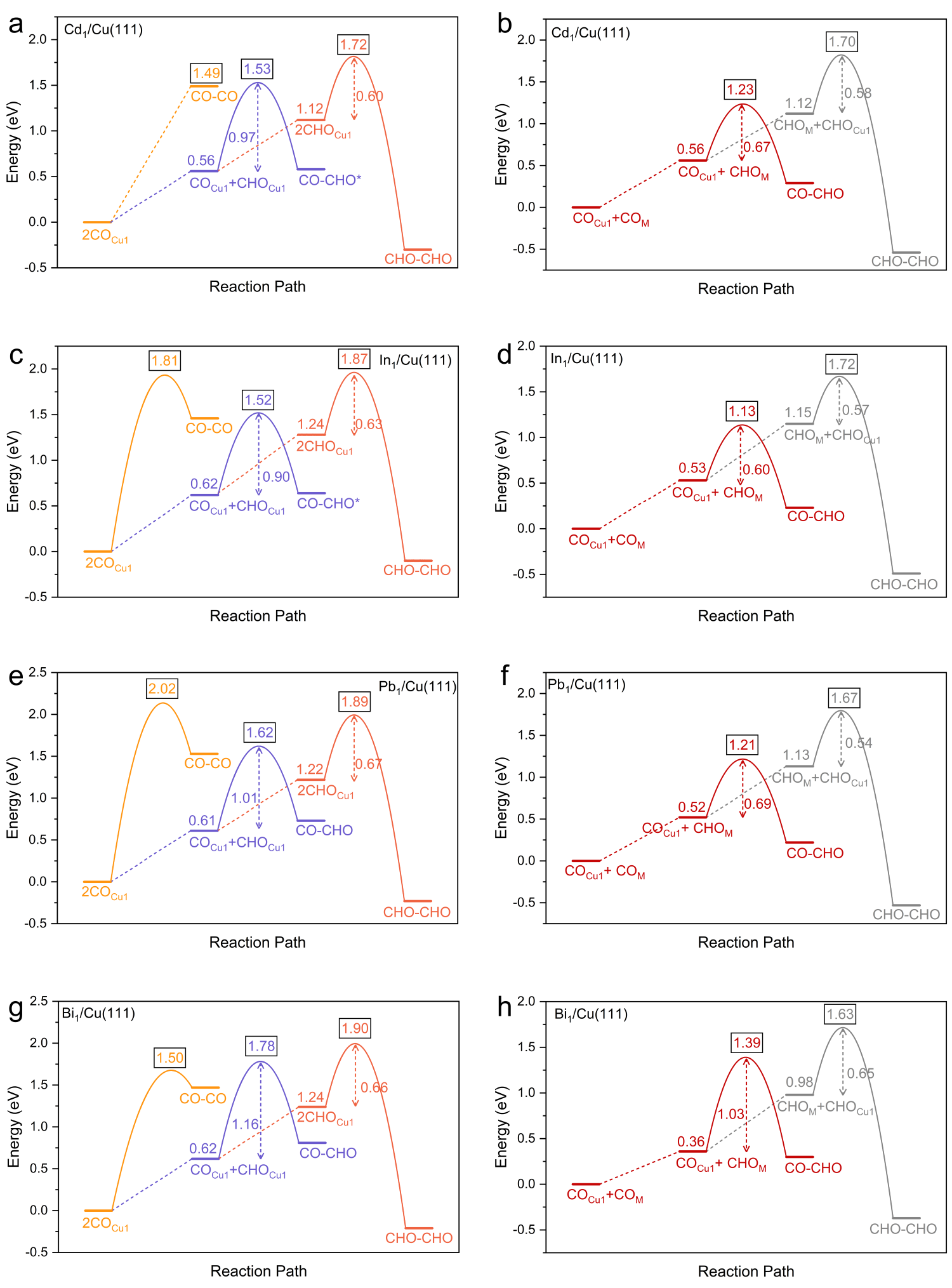


**Figure S24.** Reaction energy profiles for C-C coupling on Cu-based SAAs. (a~b) Cd_1_/Cu(111). (c~d) In_1_/Cu(111). (e~f) Pb_1_/Cu(111). (g~h) Bi_1_/Cu(111).

**
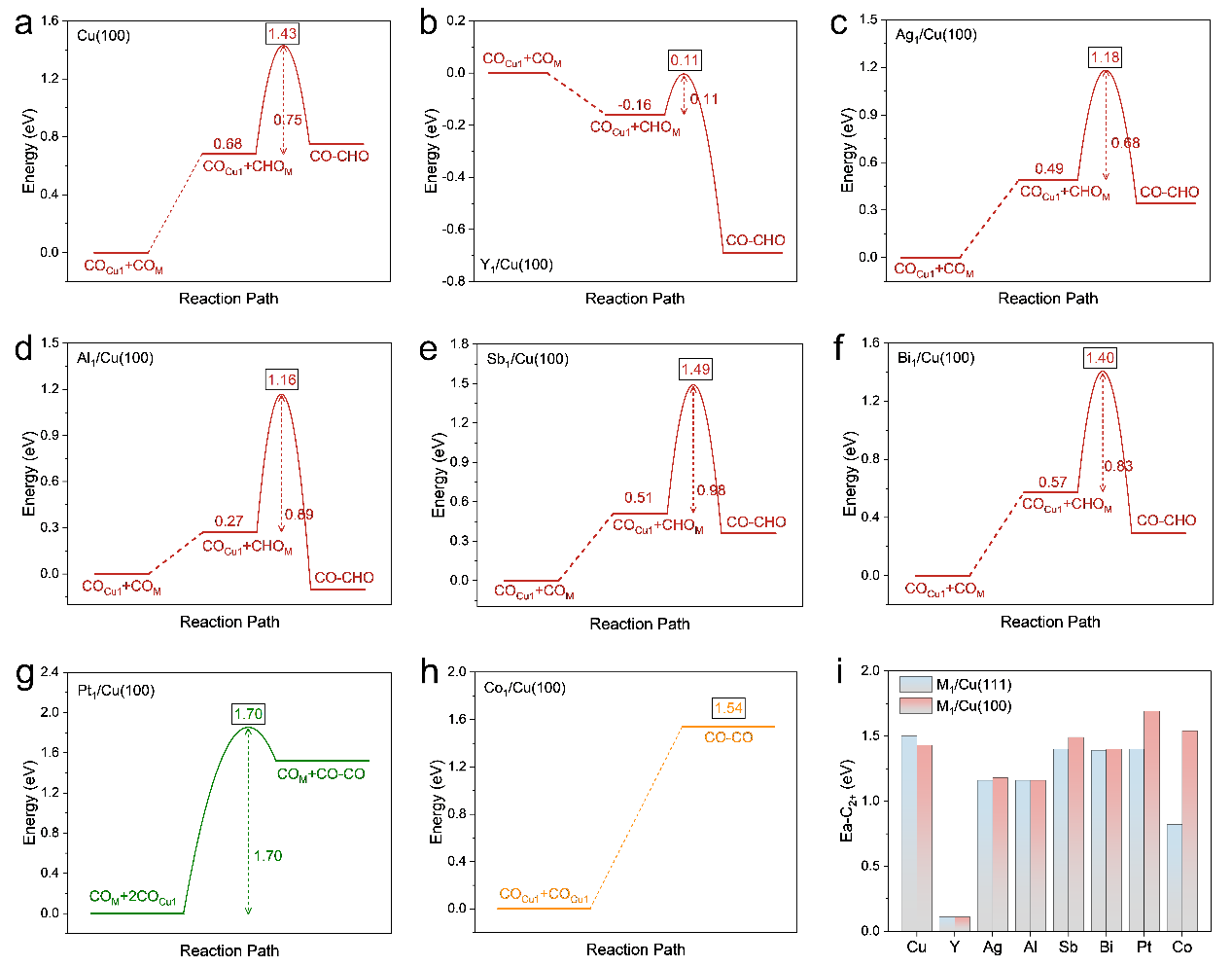
**

**Figure S25.** Reaction energy profiles for C-C coupling on Cu-based SAAs based on Cu(111) or Cu(100) facet. (a) Cu(100). (b) Y_1_/Cu(100). (c) Ag_1_/Cu(100). (d) Al_1_/Cu(100). (e) Sb_1_/Cu(100). (f) Bi_1_/Cu(100). (g) Pt_1_/Cu(100). (h) Co_1_/Cu(100). (i) Ea-C_2+_ summary of the C-C coupling on Cu-based SAAs based on Cu(111) or Cu(100) facet.


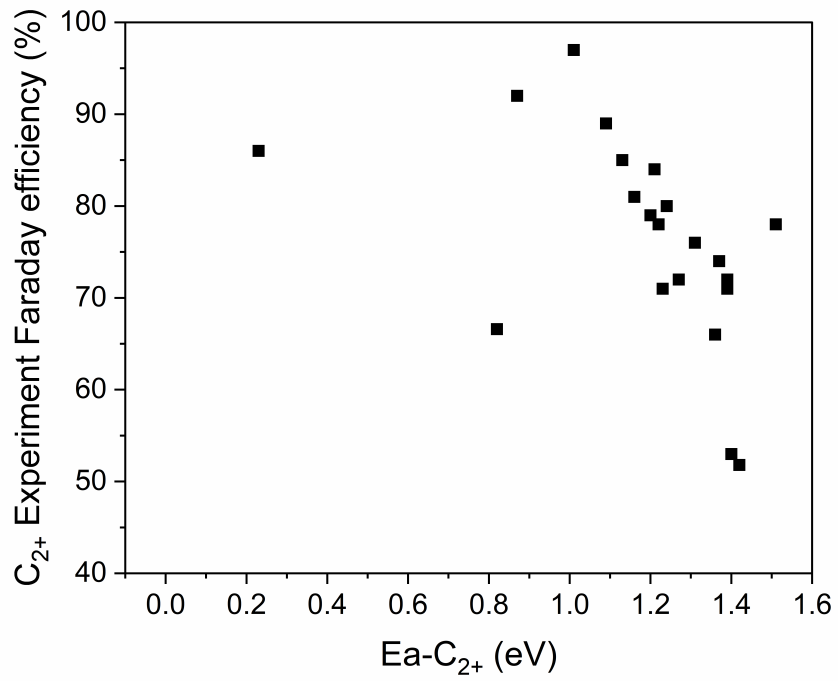


**Figure S26.** The weak correlation trend between the experimental Faraday efficiency of C_2+_ products and Ea-C_2+_.


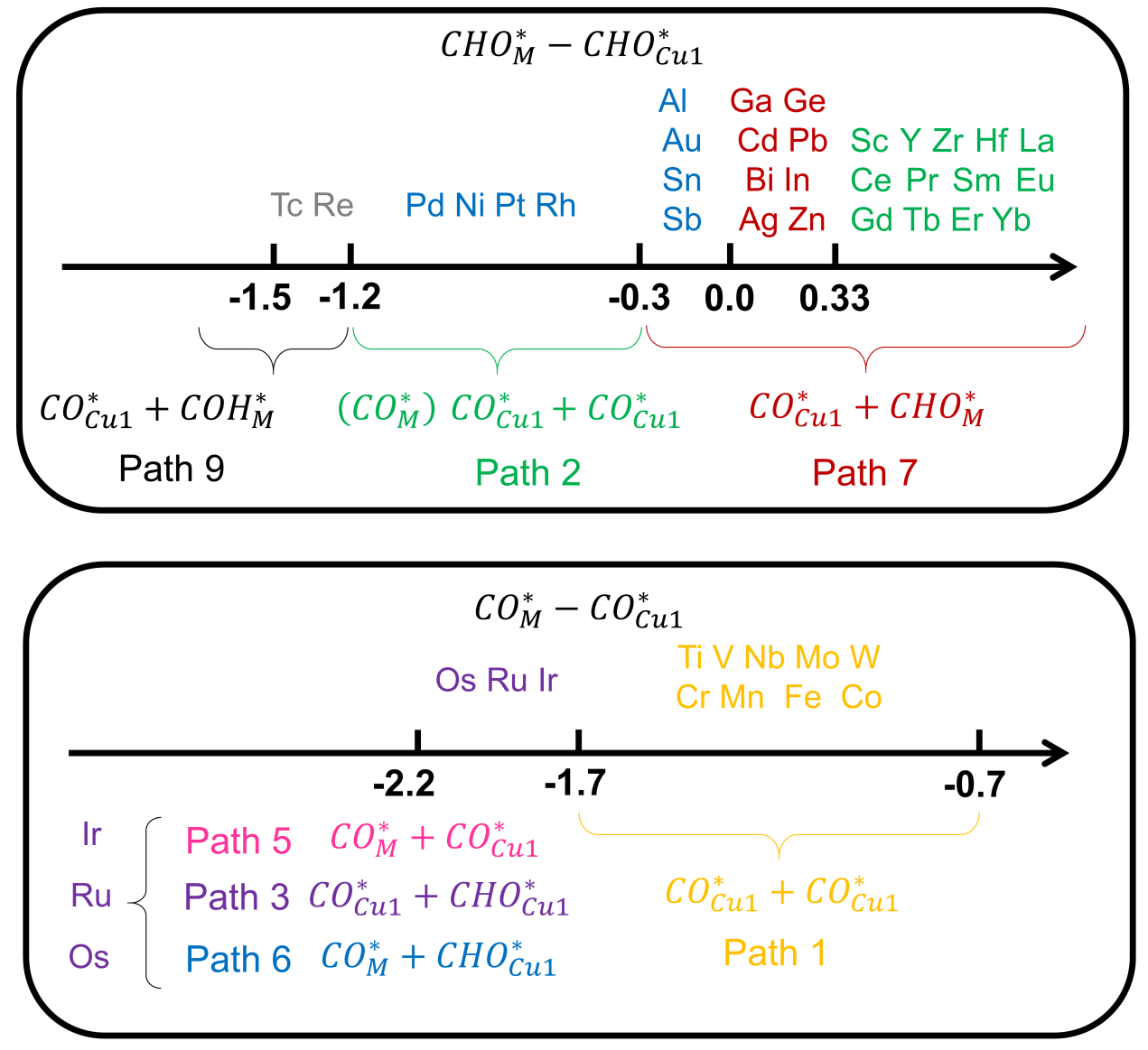


**Figure S27.** Schematic diagram of C-C coupling mechanisms based on numerical coordinates of energy descriptors. The reaction mechanism color code follows Figure 4, and the element color code follows Figure 5b.


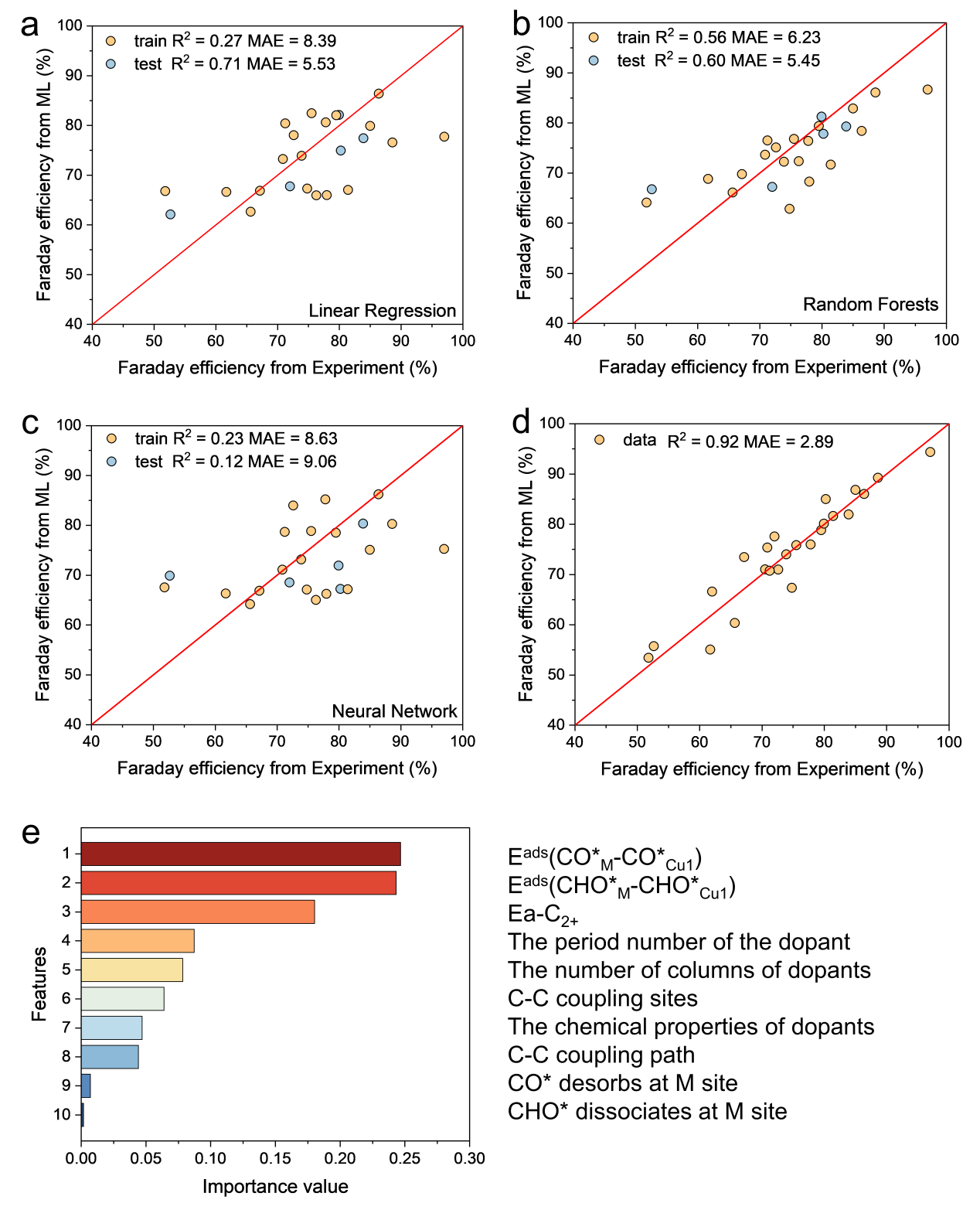


**Figure S28.** Accuracy comparison between ML-predicted and real Faraday efficiency of C_2+_ products. a. Linear Regression. b. Random Forests. c. Neural Network. d. AI agent-aided method. e. Feature importance analysis for predictive model by Random Decision Forests method.


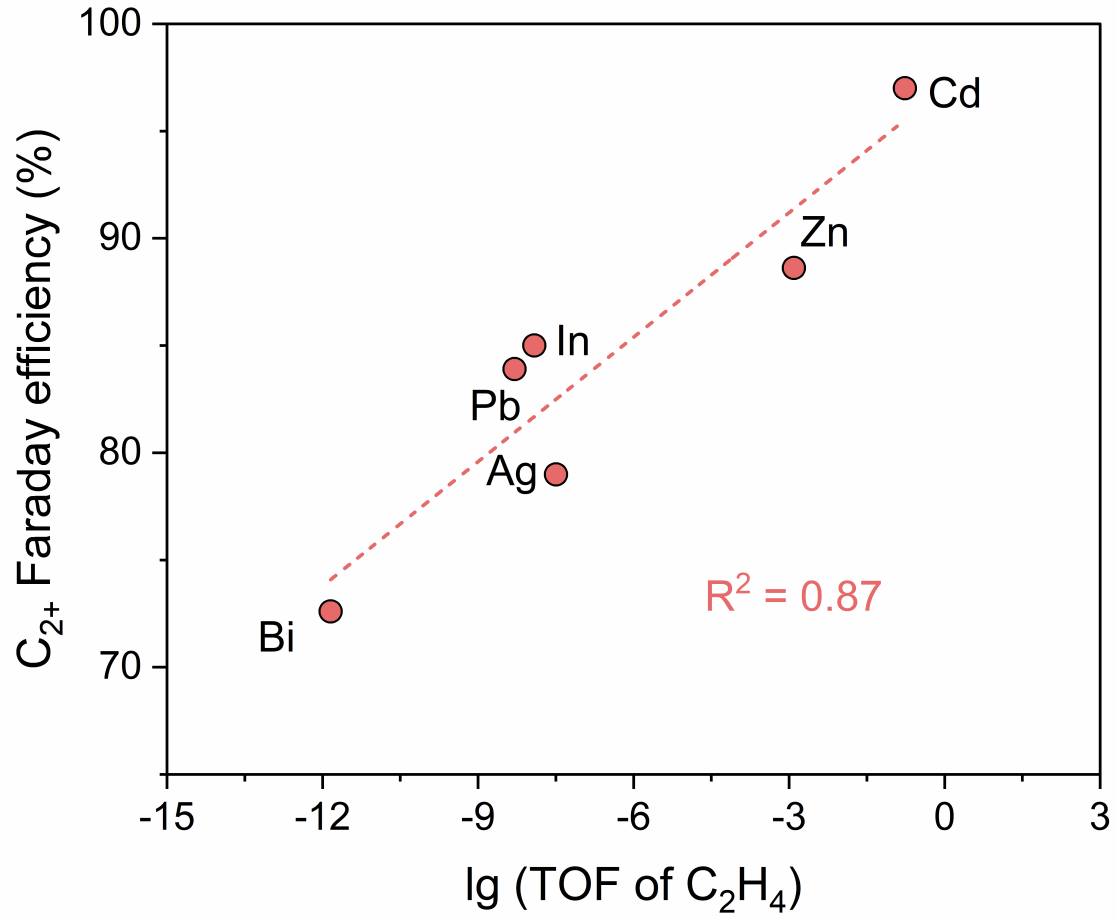


**Figure S29.** The microkinetic modelling of C_2+_ products (with ethylene as a representative example) formation on Cu-based SAAs and its corelation with the experimental Faraday efficiency.


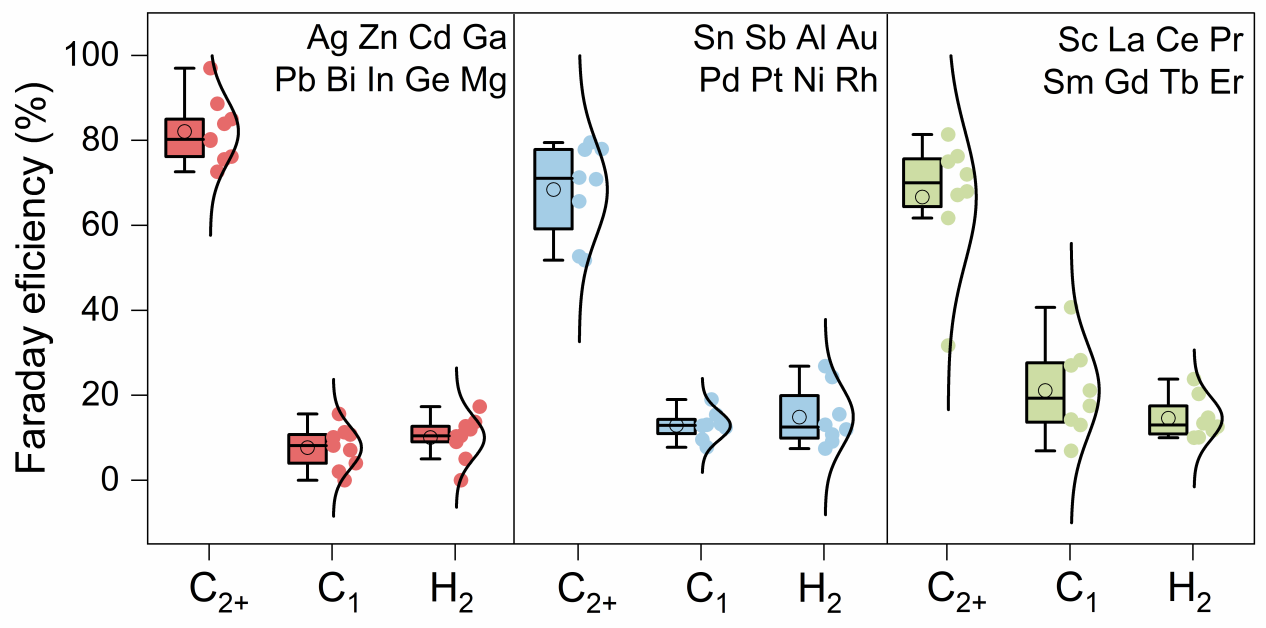


**Figure S30.** Summary of experimental Faraday efficiency of CO_2_RR products over different groups of Cu-based SAAs.


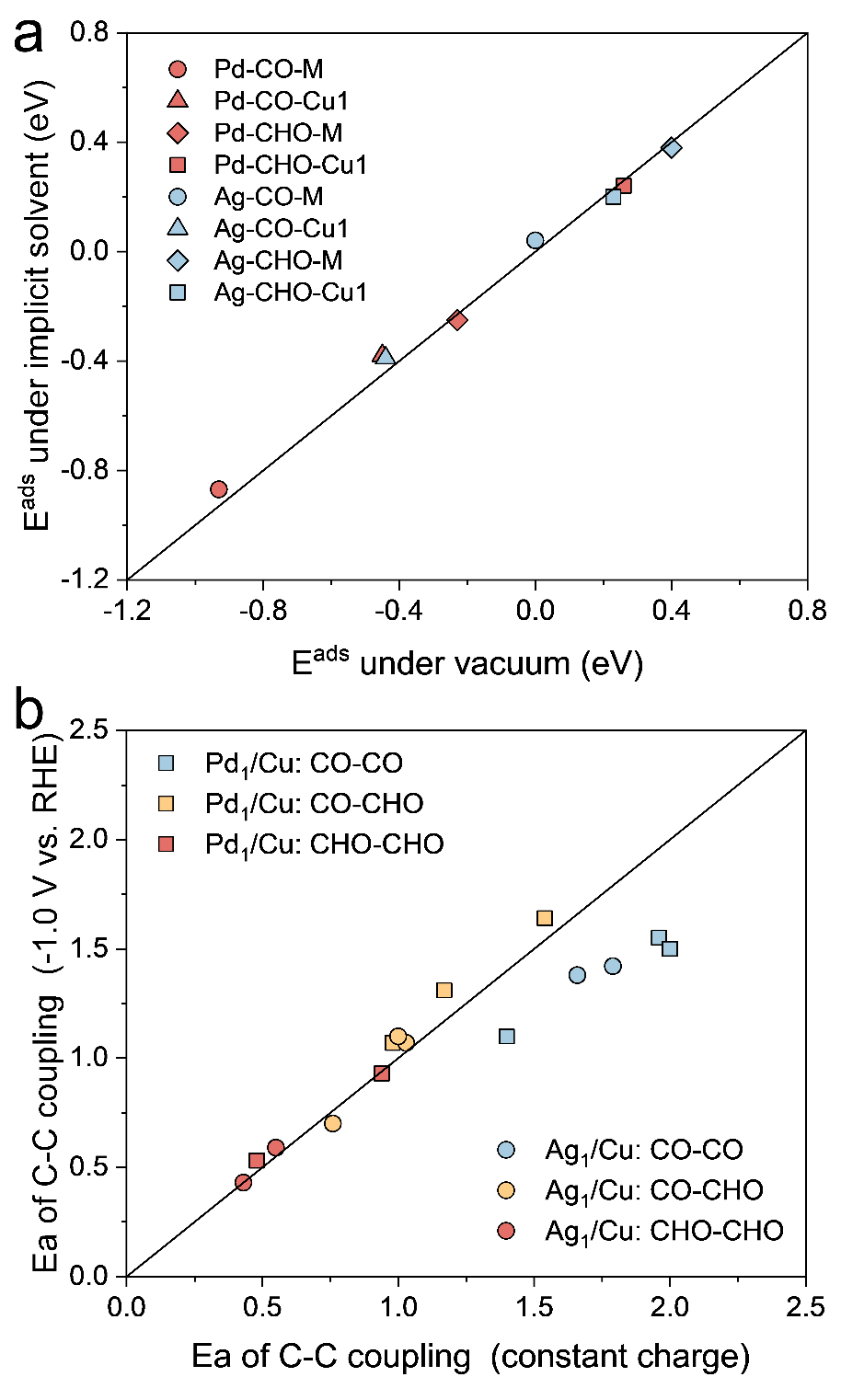


**Figure S31.** (a) Comparison of the adsorption energies of C_1_ Molecules in a vacuum atmosphere versus an implicit solvent environment. (b) The C-C coupling energy barrier (Ea / eV) under constant charge and constant-potential condition.


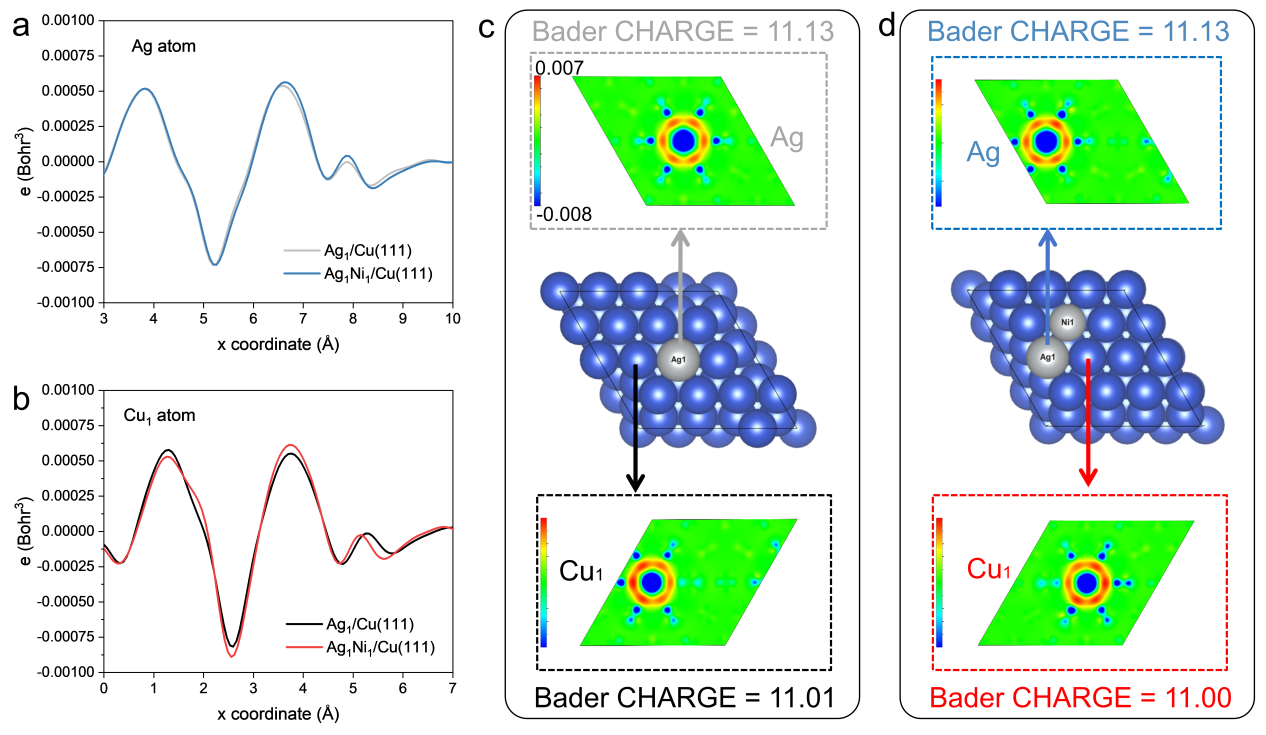


**Figure S32.** The charge density distribution of Ag atom and their first nearest neighbor Cu atom (Cu_1_) in (a) Ag_1_/Cu(111) SAA and (b) Ag_1_Ni_1_/Cu(111) DSAA. Charge density differences of Ag atom and Cu_1_ atoms in (c) Ag_1_/Cu(111) and (d) Ag_1_Ni_1_/Cu(111).


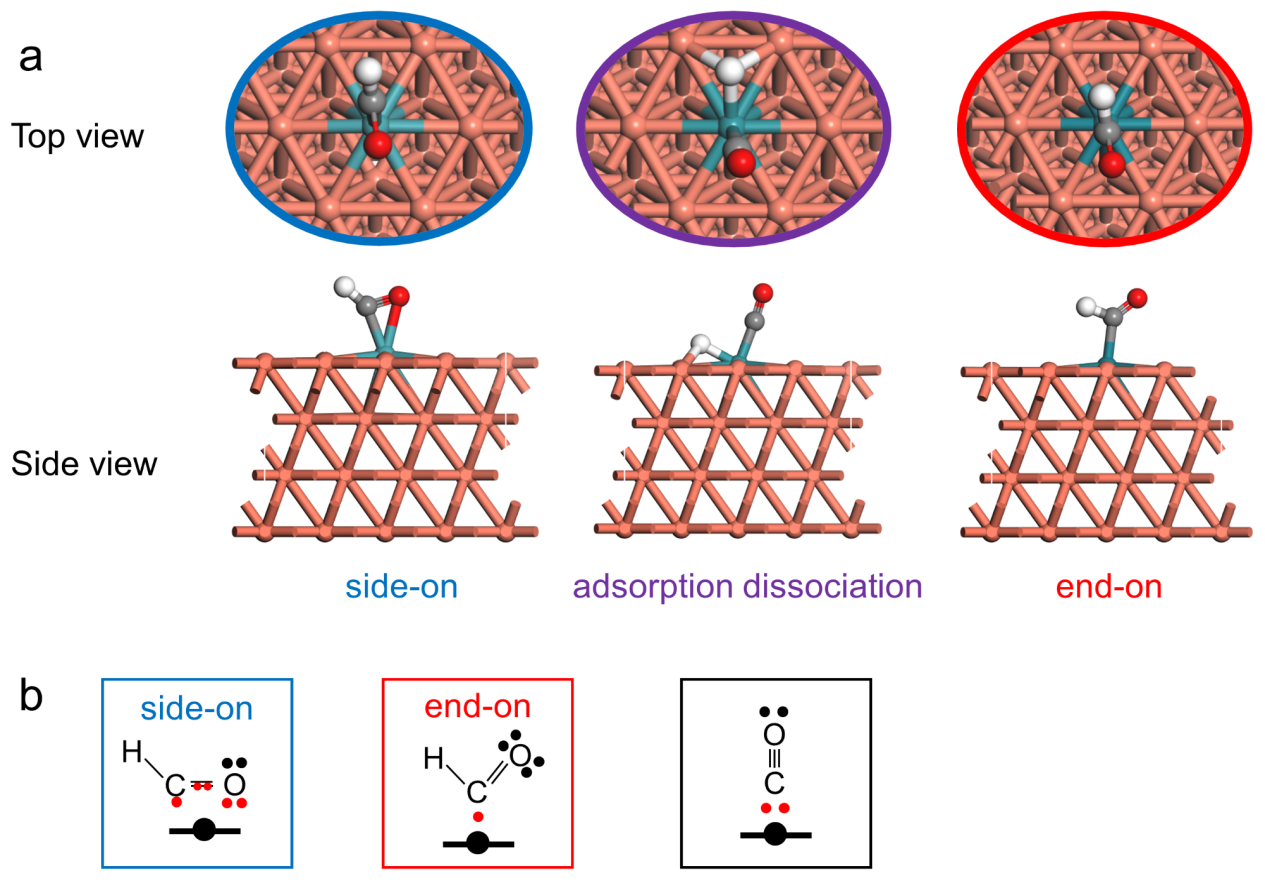


**Figure S33.** (a) Three adsorption configurations of CHO* at M site of Cu-based SAAs. Blue: side-on, purple: CHO* adsorption dissociation, red: end on. (b) Electron distribution in diverse adsorption configurations of CHO* or CO* on Cu-based SAAs.


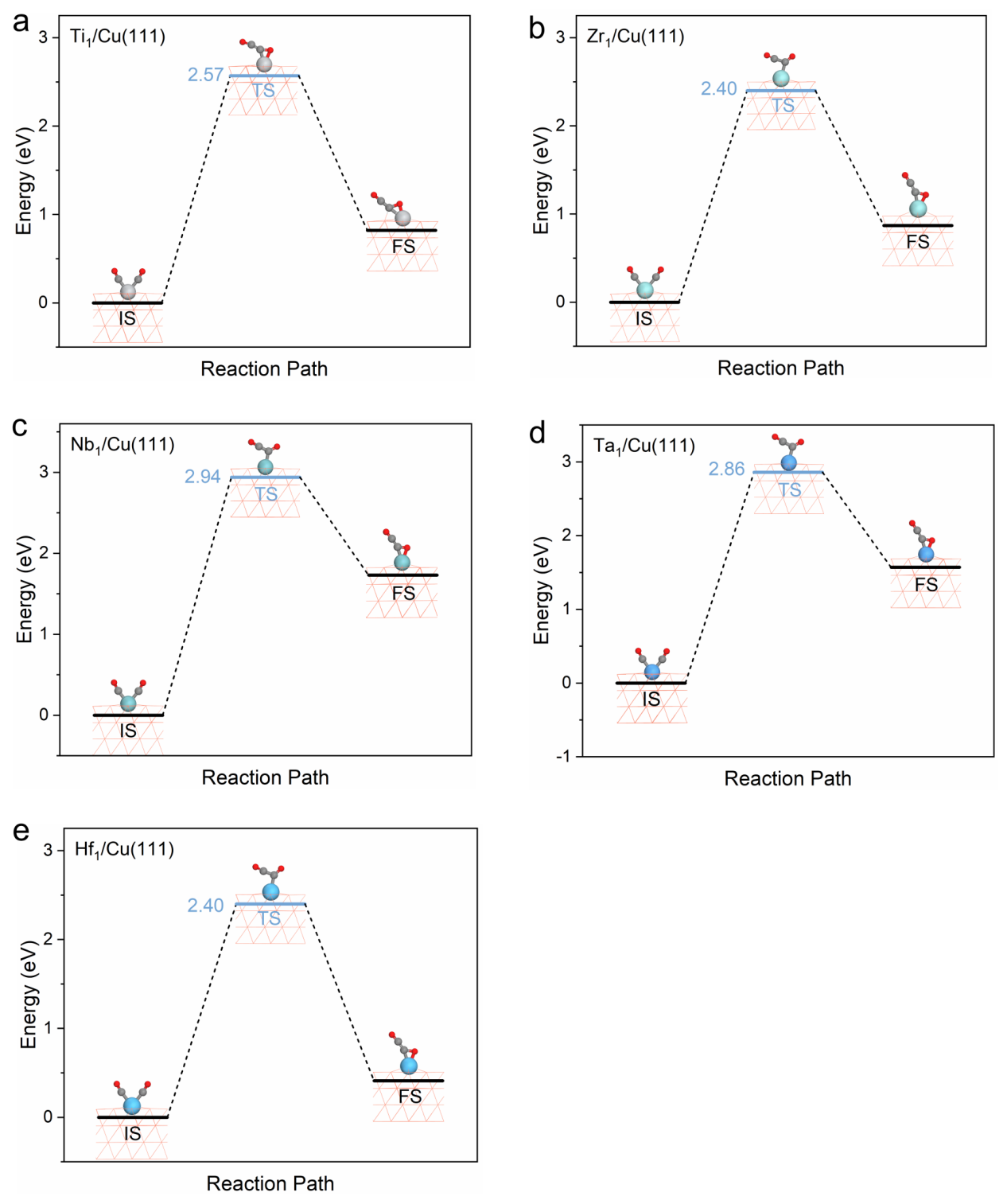


**Figure S34.** Reaction energy profile for the CO* dimerization reaction in a co-adsorbed state on the dopant site of Cu-based SAAs. (a) Ti_1_/Cu(111), (b) Zr_1_/Cu(111), (c) Nb_1_/Cu(111), (d) Ta_1_/Cu(111) and (e) Hf_1_/Cu(111).


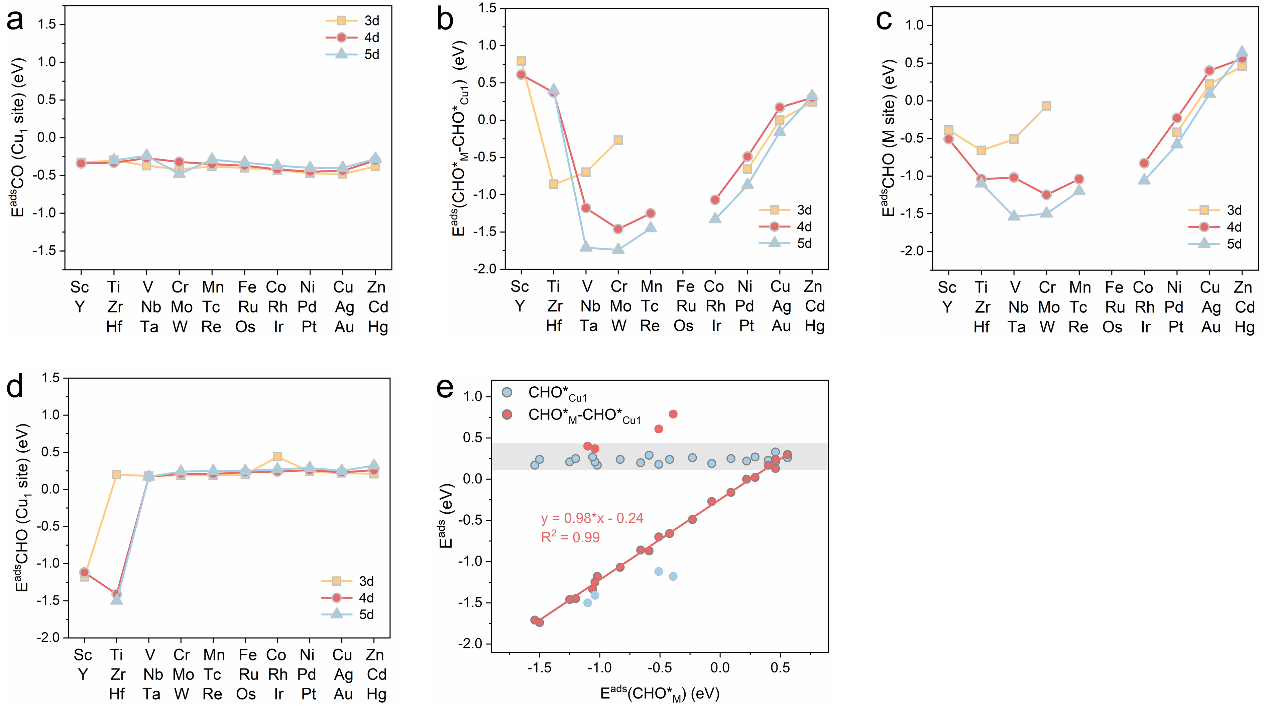


**Figure S35.** Adsorption energies of (a) CO* and (d) CHO* at the Cu_1_ site on Cu-based SAAs. (b) The difference in CHO* adsorption energies between the M site and the Cu_1_ site of the Cu-based SAAs. (c) Adsorption energies of CHO* at the M site on Cu-based SAAs. (e) Correlation of CHO* adsorption energies across M and Cu_1_ Sites.

**Figure S36.** Electron transfer from M to Cu_1_ (or Cu_1_ to M) as a function of the Δχ (electronegativity) between M and Cu_1_.


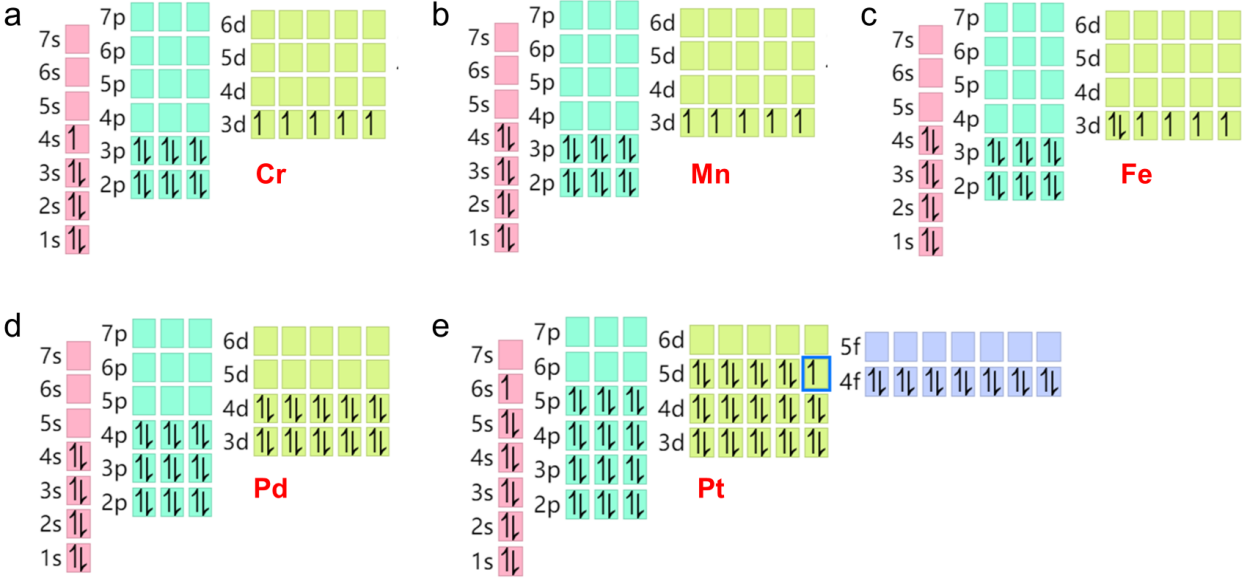


**Figure S37.** The electron configuration of a transition metal with a non-zero Y value (Y = 8-n, n: the outermost d electron number of element M). (a) Cr, (b) Mn, (c) Fe, (d) Pd and (e) Pt.


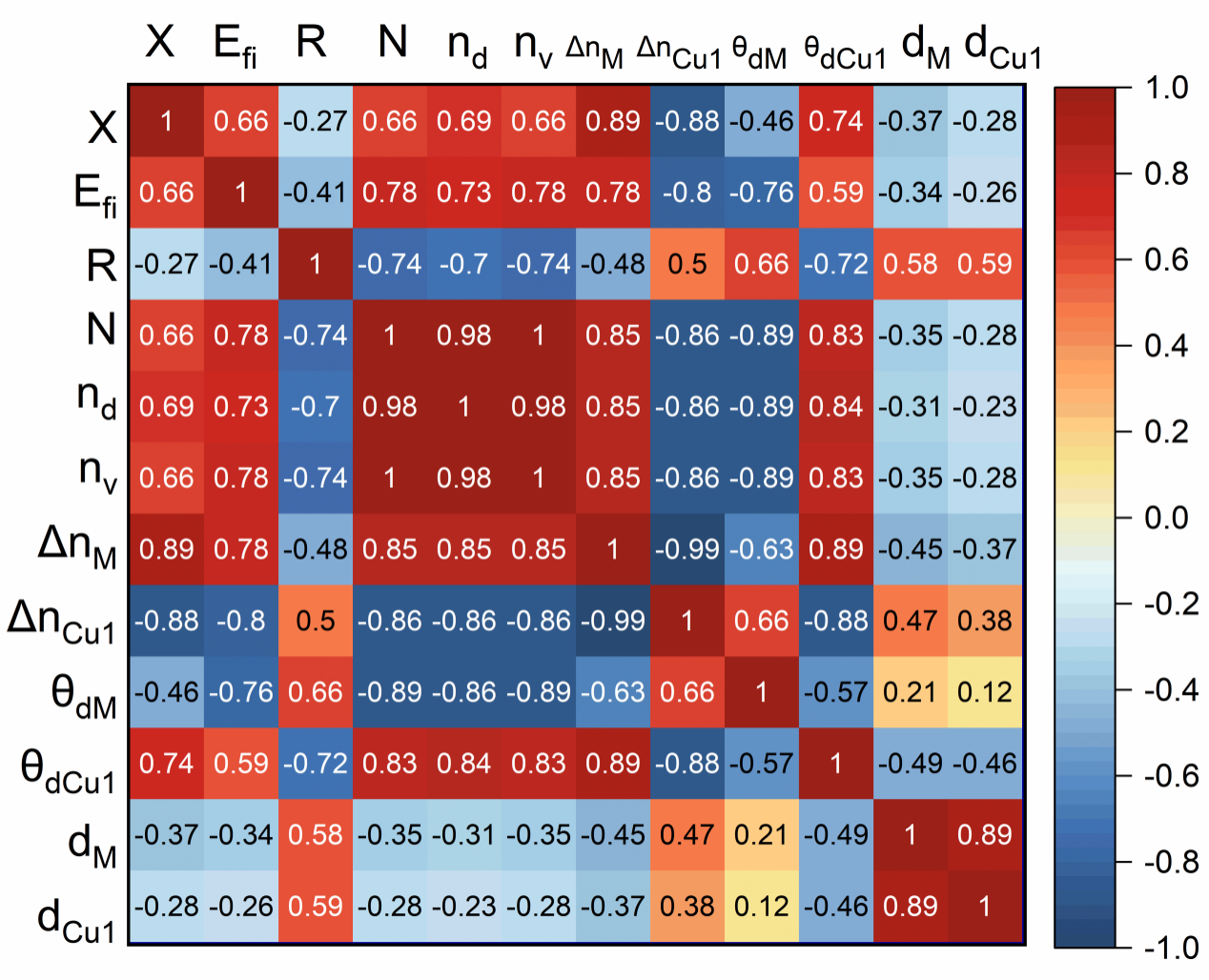


**Figure S38.** Pearson correlation coefficient of feature parameters.


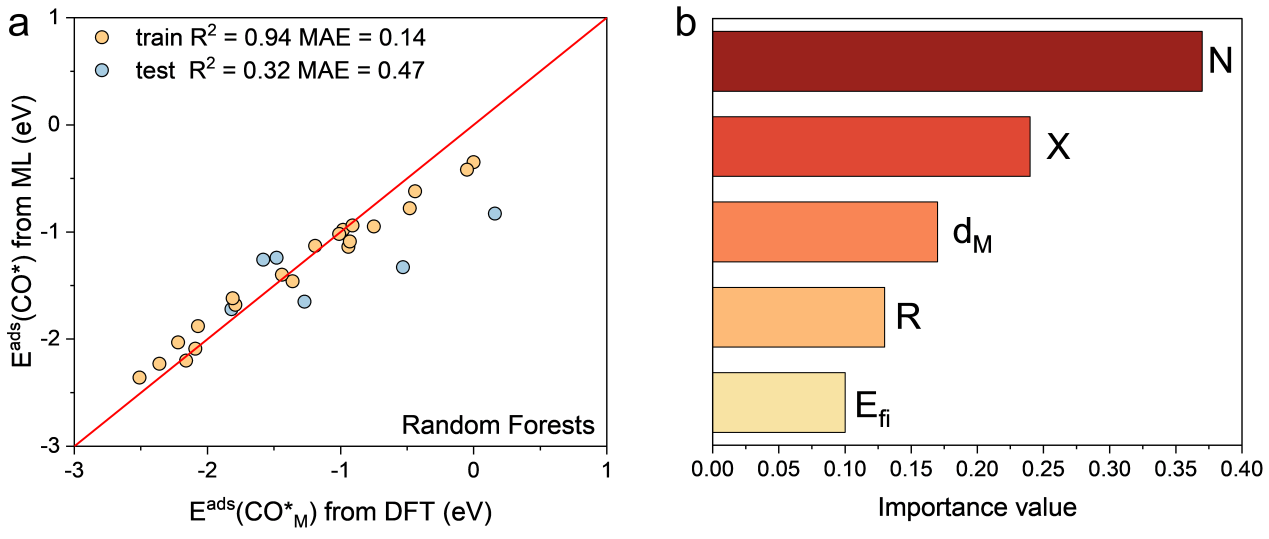


**Figure S39.** The E^ads^(CO*_M_) training results of machine learning and the importance values of feature parameters.


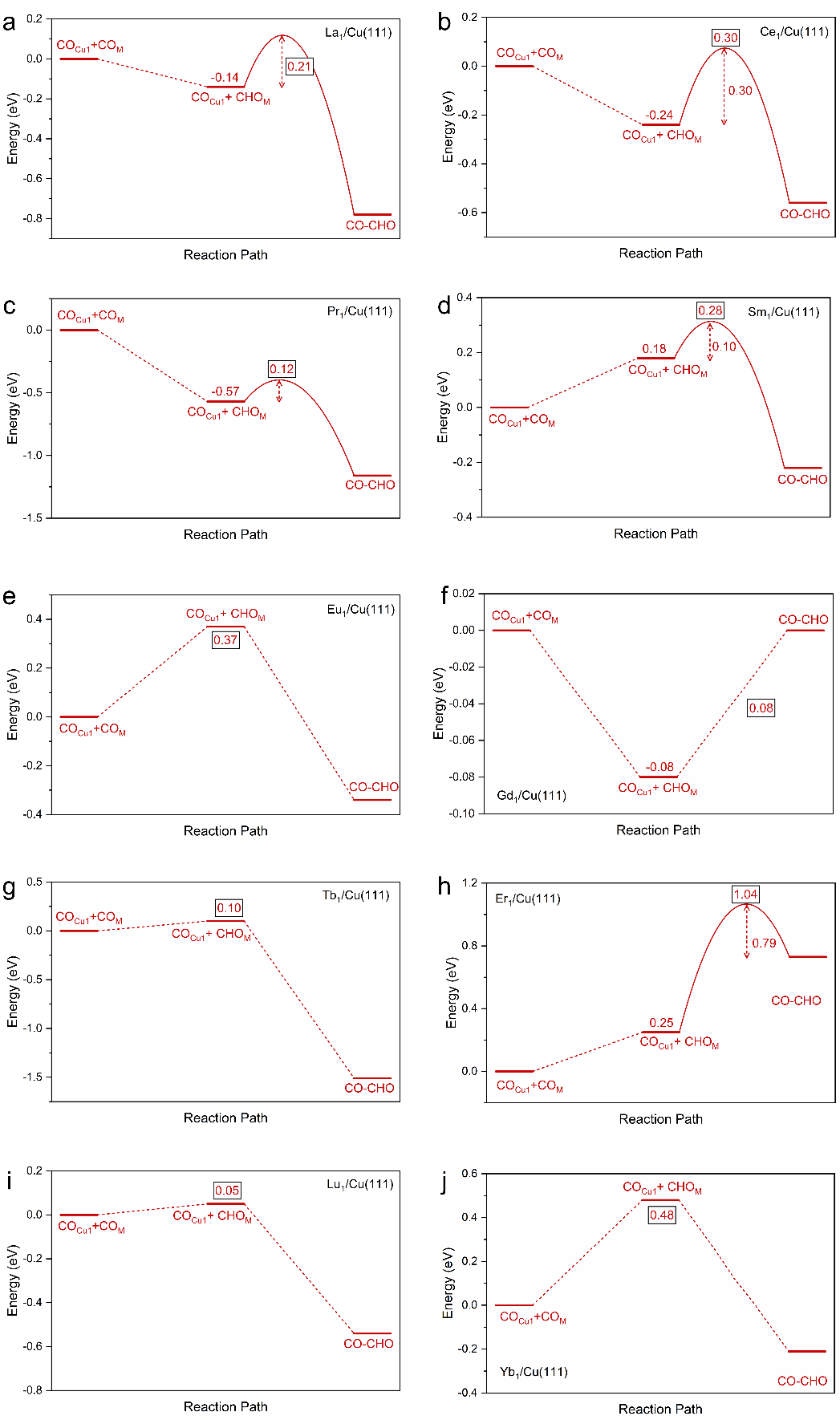


**Figure S40.** Reaction energy profile for C-C coupling on M_1_/Cu(111) (M are rare earth metals). (a) La_1_/Cu(111), (b) Ce_1_/Cu(111), (c) Pr_1_/Cu(111), (d) Sm_1_/Cu(111), (e) Eu_1_/Cu(111), (f) Gd_1_/Cu(111), (g) Tb_1_/Cu(111), (h) Er_1_/Cu(111), (i) Lu_1_/Cu(111) and (j) Yb_1_/Cu(111).


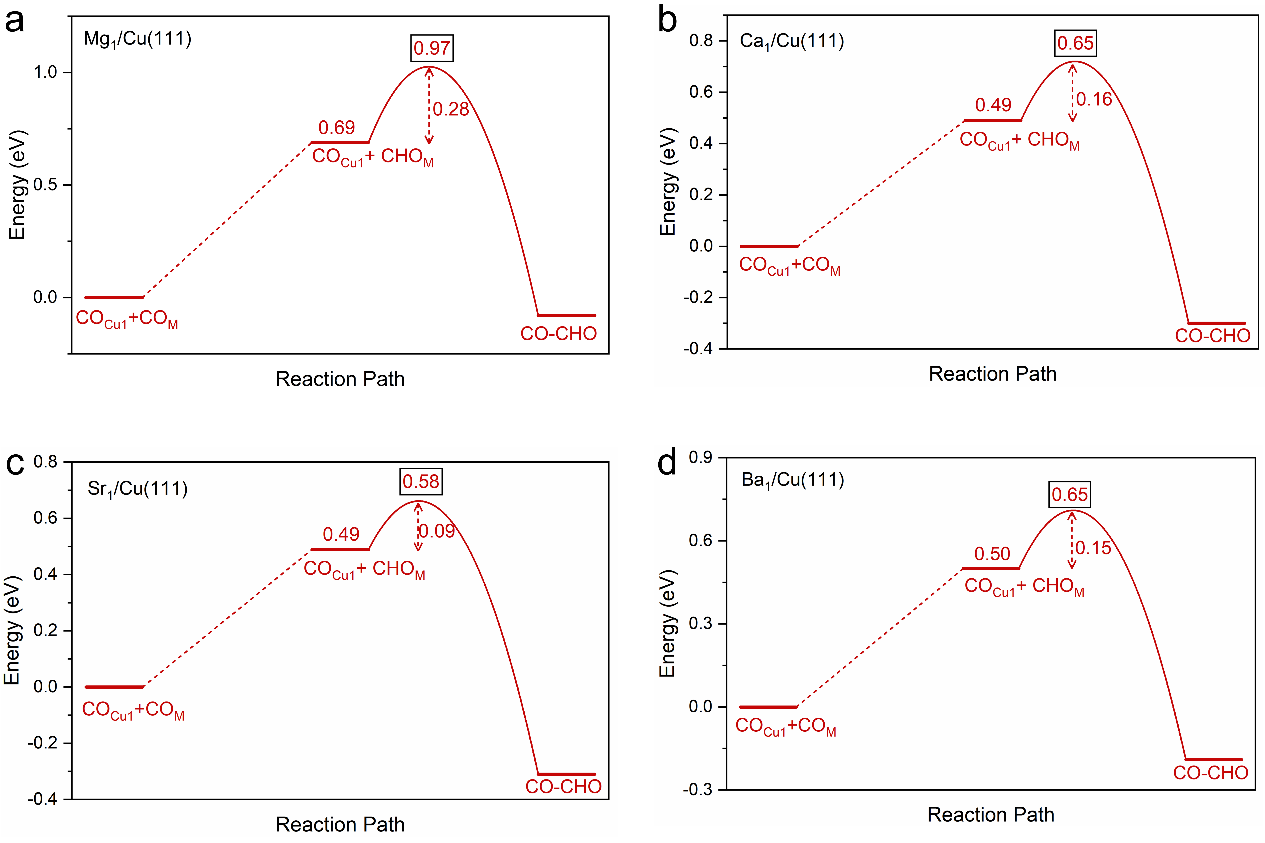


**Figure S41.** Reaction energy profile for C-C coupling on M_1_/Cu(111) (M are alkali metals). (a) Mg_1_/Cu(111), (h) Ca_1_/Cu(111), (i) Sr_1_/Cu(111) and (j) Ba_1_/Cu(111).


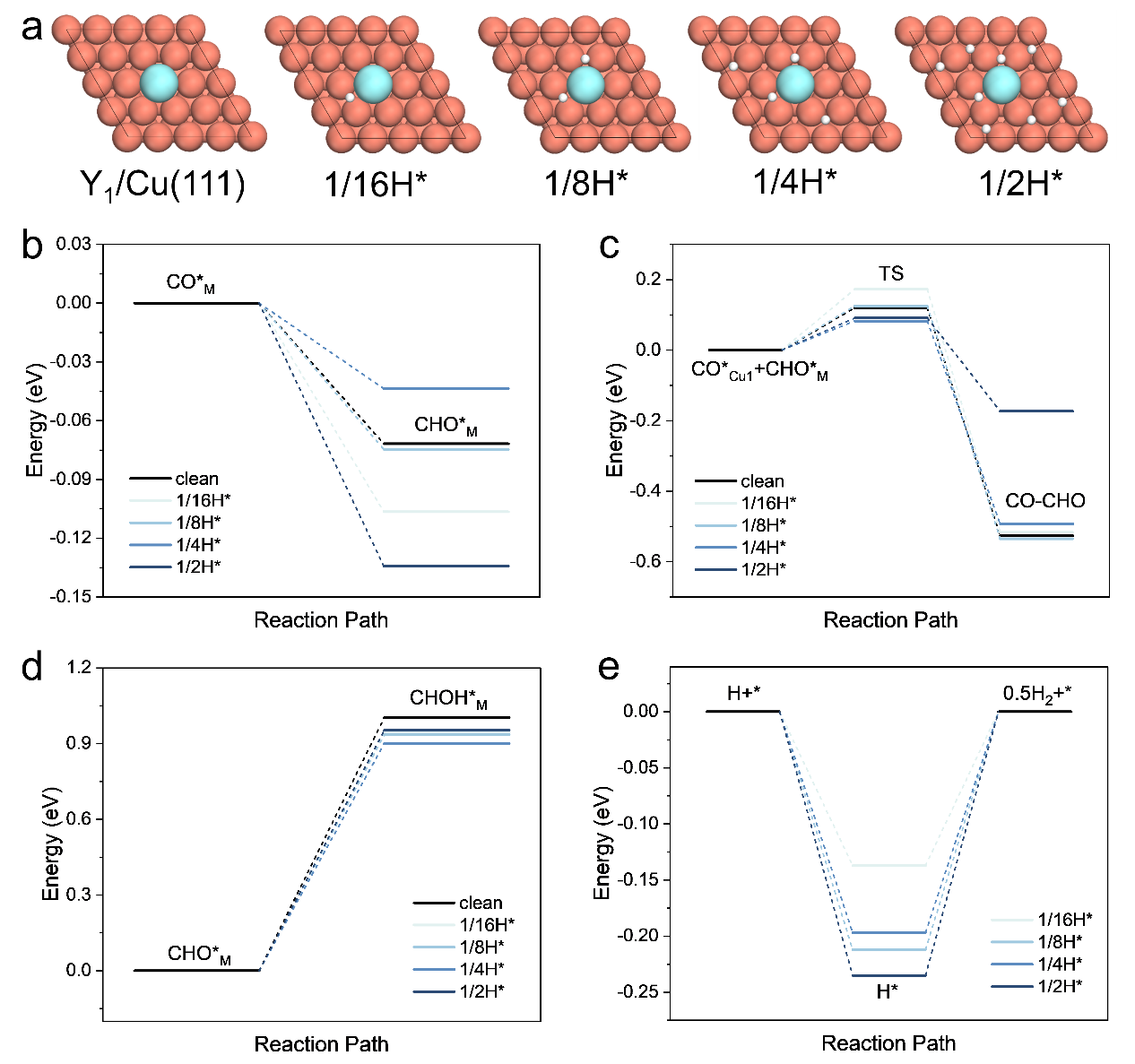


**Figure S42.** The coverage effect of H*. a. Y_1_/Cu(111) models with varying degrees of H* coverage. b. Hydrogenation of CO*. c. CO-CHO coupling. d. Hydrogenation of CHO*. e. HER.


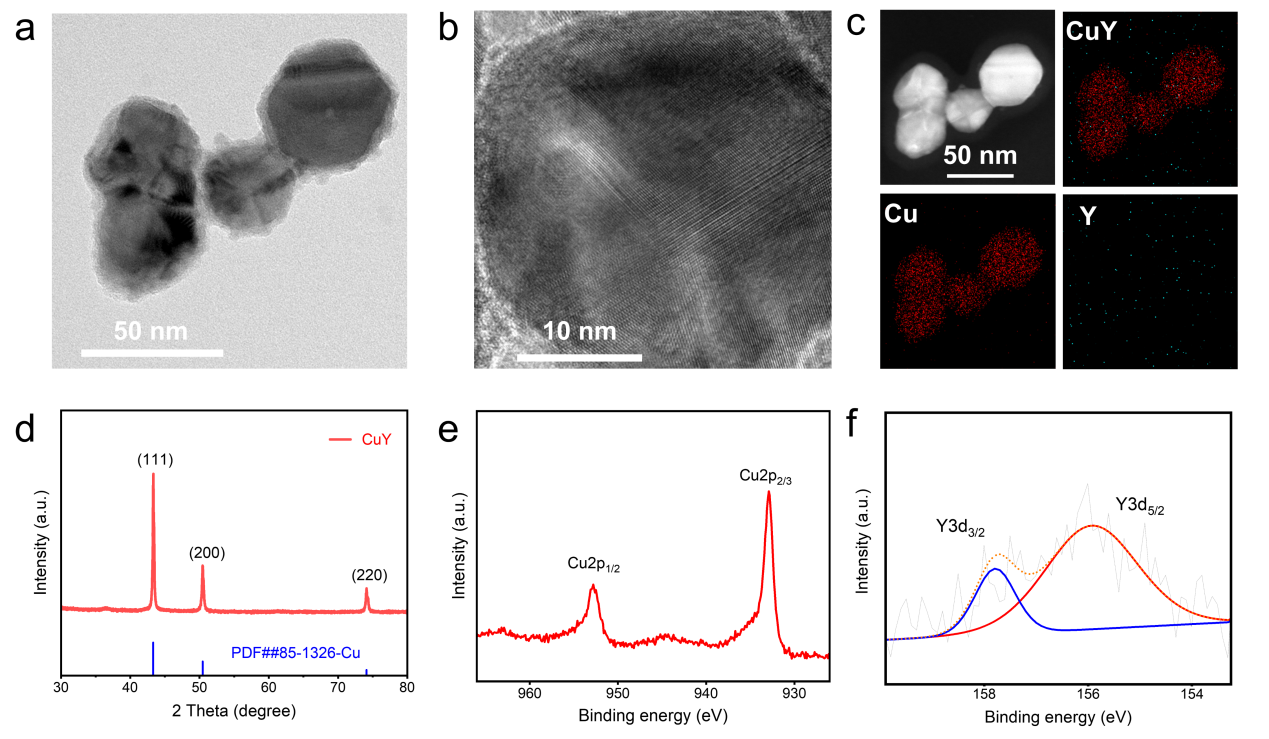


**Figure S43.** Morphology and structural characterization of CuY-SAA catalyst. a and b. HAADF-STEM images of CuY-SAA. c. EDS element mapping images of CuY-SAA (red and blue represent Cu and Y elements, respectively). d. XRD patterns of CuY-SAA. e. Cu 2p and (f) Y 3d XPS spectra of CuY-SAA.


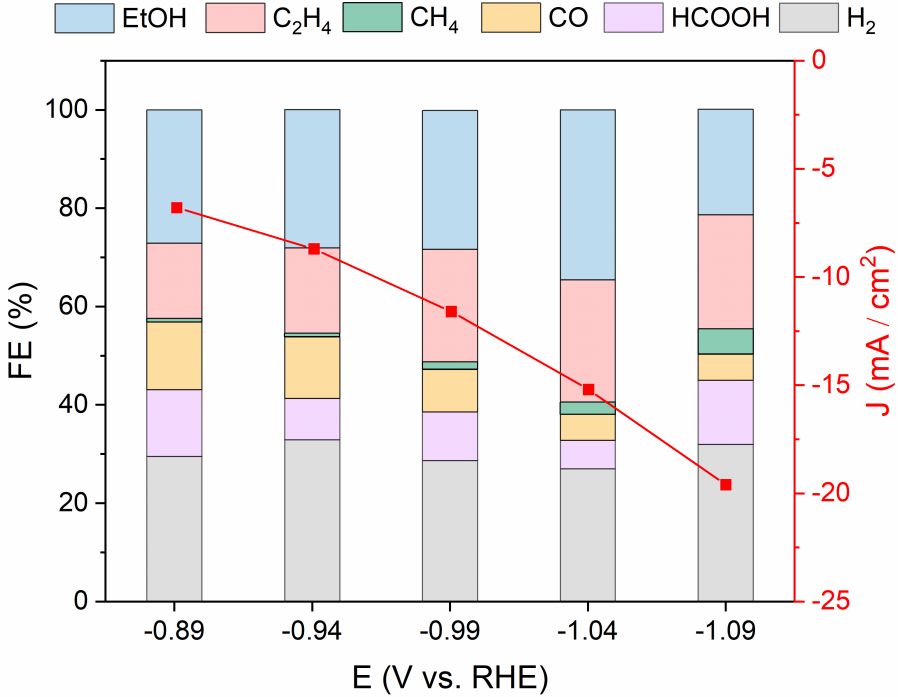


**Figure S44.** Product distributions of CO_2_RR on CuY-SAA catalyst.


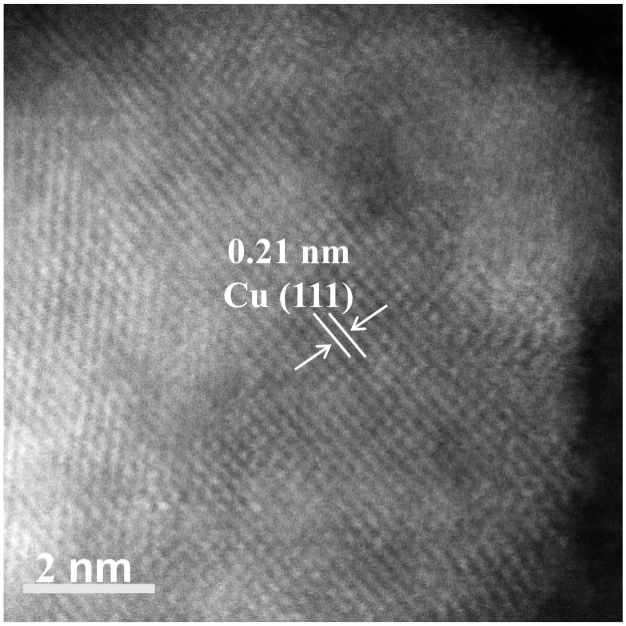


**Figure S45.** HR-TEM image of the CuY-SAA catalyst.


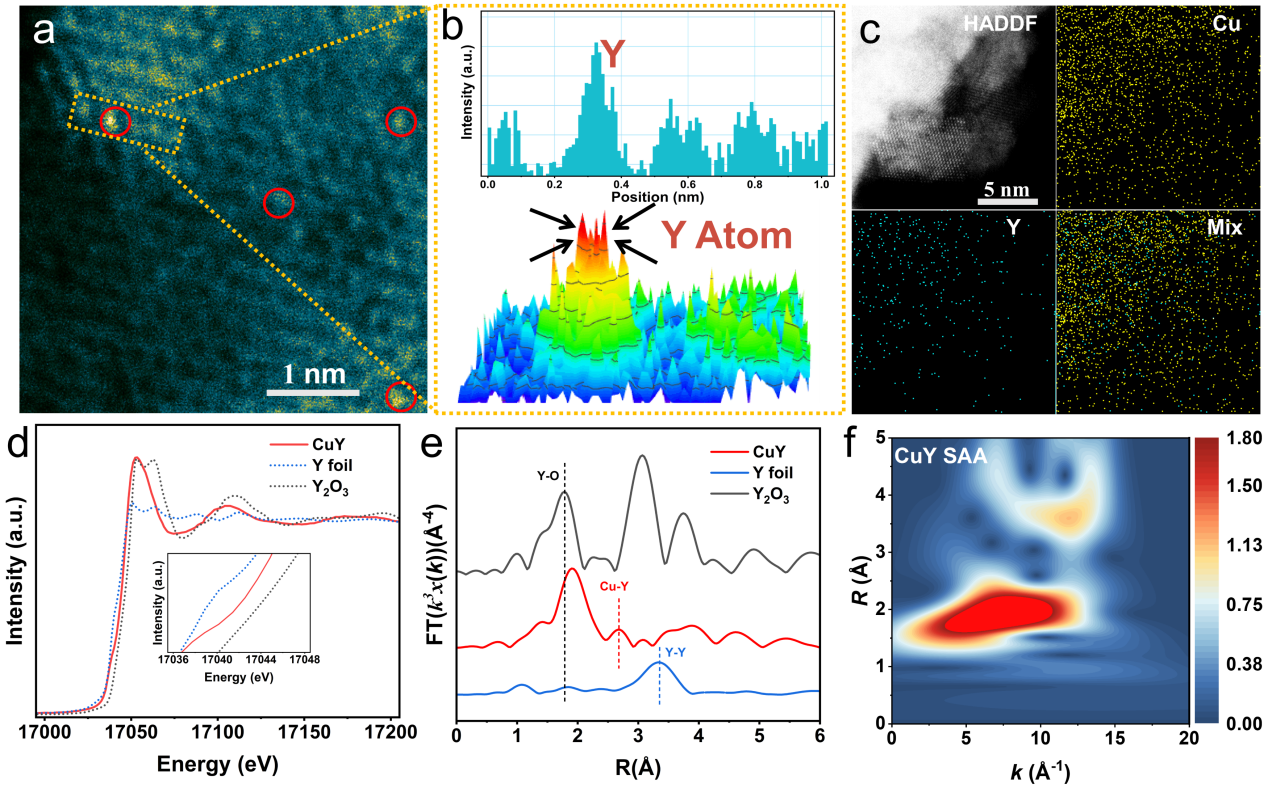


**Figure S46.** Morphology and structural characterization. (a) AC-HAADF-STEM image of the CuY-SAA catalyst. (b) Line intensity profile and 3D topographic atom image along the yellow dotted lines in a. (c) EDS element mapping imYes of the CuY-SAA. (d) Y K-edge XANES spectra of the CuY-SAA. (E) Y K-edge FT-EXAFS spectra of the CuY-SAA. (f) Wavelet transform image of the CuY-SAA.


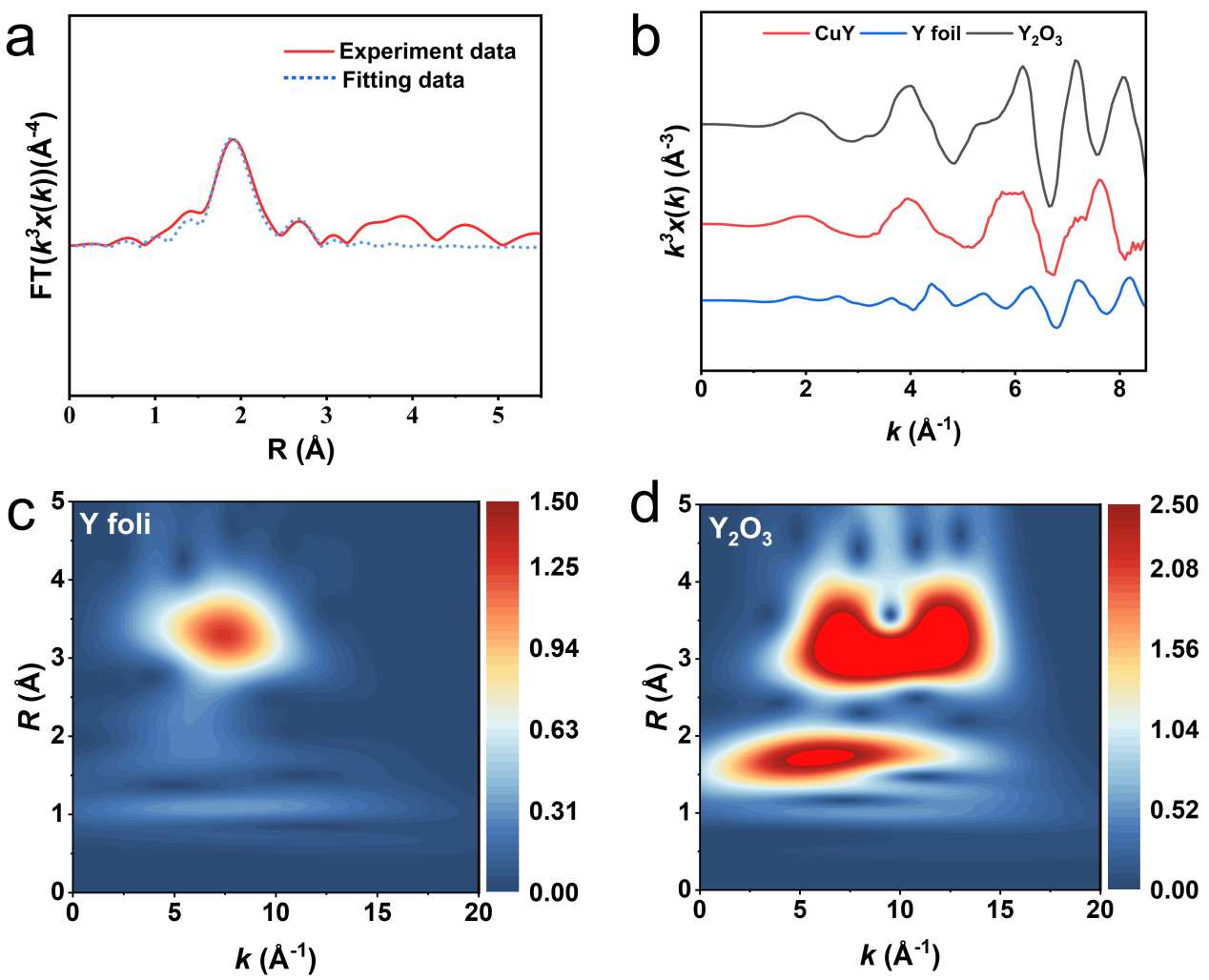


**Figure S47.** (a) Quantitative EXAFS curve fitting in the R space of CuY-SAA catalyst. (b) Fitted EXAFS spectra of CuY-SAA at K-space. (c) Wavelet transform image of the Y foli. (d) Wavelet transform image of the Y_2_O_3_.


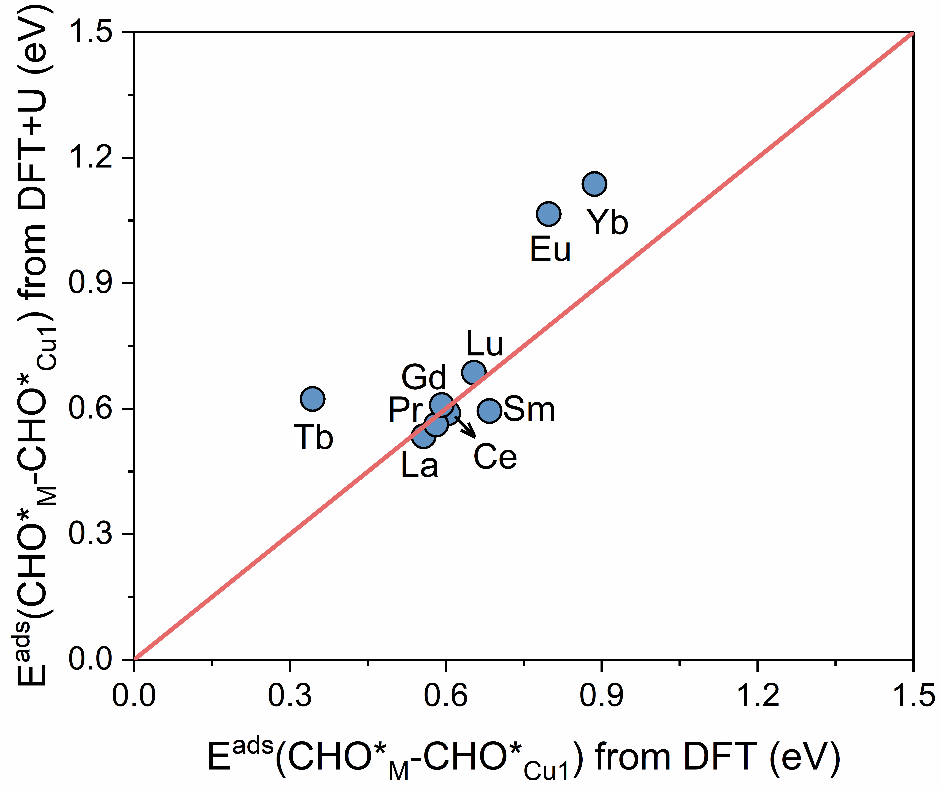


**Figure S48.** Comparison of the adsorption energies of Eads(CHO*M-CHO*Cu1) on M_1_/Cu(111) (M= La, Ce, Pr, Sm, Eu, Gd, Tb, Yb, Lu) from DFT or DFT+U method.

**Part 3 Supplementary Tables**

**Table S1-1.** Experimental Faraday efficiency of C_2+_ products on Cu-based SAAs.

| M | FE(C_2+_) / % | | Electrode potential  V vs. RHE | Electrolyte solution | Electrolytic cell | DOI |
| --- | --- | --- | --- | --- | --- | --- |
|  | average |  |  |  |  |  |
| Cu | 15.8 | 15.8^18^ | -1.15 | 0.1 M KHCO_3_ | H-cell | 10.1016/S1381-1169(03)00016-5 |
| Au | 71.0 | 93.0^19^ | / | 2 M KOH  +1M CsOH | Flow-cell | 10.1038/s41467-024-44727-z |
|  |  | 76.0^20^ | -0.66 | 1 M KOH | Flow-cell | 10.1038/s44286-023-00018-w |
|  |  | 58.0^21^ | / | 1 M KOH | Flow-cell | 10.1021/acscatal.2c06145 |
|  |  | 57.0^22^ | -0.51 | 1 M KOH | Flow-cell | 10.1021/jacs.3c11013 |
| Ag | 81.0 | 94.0^23^ | -0.65 | 1 M KOH | Flow-cell | 10.1038/s41467-023-41871-w |
|  |  | 90.0^24^ | -1.60 | 1 M KOH | Flow-cell | 10.1002/adma.202411498 |
|  |  | 84.0^25^ | -0.67 | 1 M KOH | Flow-cell | 10.1021/jacs.9b02945 |
|  |  | 76.0^20^ | -0.66 | 1 M KOH | Flow-cell | 10.1038/s44286-023-00018-w |
|  |  | 72.0^26^ | -0.78 | 2 M KOH | Flow-cell | 10.1021/jacs.4c11276 |
|  |  | 70.0^27^ | -1.00 | 1 M KOH | Flow-cell | 10.1038/s41467-024-54636-w |
|  |  | 83.0^28^ | / | 1 M KOH | Flow-cell | 10.1007/s11426-024-2292-3 |
|  |  | 83.0^29^ | / | 1 M KOH | Flow-cell | 10.1021/jacs.5c03057 |
|  |  | 77.0^30^ | / | 1 M KOH | Flow-cell | 10.1002/adfm.202503497 |
|  |  | 98.0^31^ | / | 0.5 M KOH | MEA | 10.1002/anie.202507062 |
|  |  | 70.0^32^ | -0.71 | 1 M KOH | Flow-cell | 10.1126/sciadv.aaz6844 |
|  |  | 80.0^33^ | -1.80 | 0.05 M H2SO4  + 3 M KCl | Flow-cell | 10.1002/anie.202408873 |
|  |  | 66.0^34^ | / | 0.1 M KHCO3 | MEA | 10.1038/s41929-025-01411-9 |
| Pd | 66.0 | 84.0^19^ | / | 2 M KOH  +1M CsOH | Flow-cell | 10.1038/s41467-024-44727-z |
|  |  | 81.0^35^ | -0.88 | 1 M KOH | Flow-cell | 10.1002/anie.202400439 |
|  |  | 80.0^26^ | -0.78 | 2 M KOH | Flow-cell | 10.1021/jacs.4c11276 |
|  |  | 76.0^20^ | -0.66 | 1 M KOH | Flow-cell | 10.1038/s44286-023-00018-w |
|  |  | 66.0^36^ | -0.60 | 1 M KOH | Flow-cell | 10.1002/smll.202401656 |
|  |  | 66.0^37^ | -0.75 | 1 M KOH | Flow-cell | 10.1002/smll.202302530 |
|  |  | 47.0^38^ | -0.70 | 0.5 M KHCO_3_ | H-cell | 10.1021/jacs.4c07711 |
|  |  | 21.0^39^ | -0.90 | 0.5 M KHCO_3_ | Flow-cell | 10.1038/s41467-023-38777-y |
|  |  | 72.0^40^ | -0.81 | 1 M KOH | Flow-cell | 10.1016/j.apcatb.2025.125412 |
|  |  | 64.0^32^ | -0.74 | 1 M KOH | Flow-cell | 10.1126/sciadv.aaz6844 |
| Pt | 53.0 | 56.0^26^ | -0.78 | 2 M KOH | Flow-cell | 10.1021/jacs.4c11276 |
|  |  | 32.0^39^ | -0.90 | 0.5 M KHCO_3_ | Flow-cell | 10.1038/s41467-023-38777-y |
|  |  | 70.0^41^ | -1.20 | 1 M KOH | Flow-cell | 10.1002/anie.202424749 |
| Ir | 74.0 | 74.0^20^ | -0.66 | 1 M KOH | Flow-cell | 10.1038/s44286-023-00018-w |
| Rh | 78.0 | 78.0^20^ | -0.66 | 1 M KOH | Flow-cell | 10.1038/s44286-023-00018-w |
| Ru | 71.0 | 71.0^20^ | -0.66 | 1 M KOH | Flow-cell | 10.1038/s44286-023-00018-w |
| Mn | 92.0 | 92.0^42^ | / | 1 M KOH | MEA | 10.1021/acs.nanolett.5c01205 |
| Co | 67 | 62.0^43^ | -1.01 | 1 M KHCO_3_ | Flow-cell | 10.1021/acsenergylett.3c00418 |
|  |  | 58.0^26^ | -0.78 | 2 M KOH | Flow-cell | 10.1021/jacs.4c11276 |
|  |  | 53.0^19^ | / | 2 M KOH  +1M CsOH | Flow-cell | 10.1038/s41467-024-44727-z |
|  |  | 94.0^44^ | / | 1 M KOH | Flow-cell | 10.1039/D4EE06192F |
| Ni | 52.0 | 63.0^45^ | -0.90 | 1 M KOH | Flow-cell | 10.1021/acsami.1c19224 |
|  |  | 48.0^26^ | -0.78 | 2 M KOH | Flow-cell | 10.1021/jacs.4c11276 |
|  |  | 21.0^46^ | -0.97 | 0.5 M NaHCO_3_ | H-cell | 10.1016/j.apcatb.2021.120030 |
|  |  | 76.0^32^ | -0.70 | 1 M KOH | Flow-cell | 10.1126/sciadv.aaz6844 |
| Mo | 86.0 | 86.0^47^ | -1.61 | 1 M KOH | Flow-cell | 10.1002/adma.202412658 |
| Zn | 89.0 | 91.0^15^ | -0.55 | 0.75 M KOH | Flow-cell | 10.1038/s41467-023-36926-x |
|  |  | 86.0^48^ | / | 1 M KOH | Flow-cell | 10.1002/anie.202420283 |
| Cd | 97.0 | 97.0^19^ | / | 2 M KOH  +1M CsOH | Flow-cell | 10.1038/s41467-024-44727-z |
| Al | 79.0 | 84.0^49^ | -1.00 | 1 M KOH | Flow-cell | 10.1021/jacs.3c06697 |
|  |  | 92.0^50^ | / | 1 M KOH | Flow-cell | 10.1038/s41586-020-2242-8 |
|  |  | 77.0^51^ | -1.07 | 1 M KOH | Flow-cell | 10.1021/jacs.2c12743 |
|  |  | 72.0^52^ | -0.84 | 1 M KHCO_3_ | Flow-cell | 10.1016/j.apsusc.2022.152518 |
|  |  | 82.0^28^ | / | 1 M KOH | Flow-cell | 10.1007/s11426-024-2292-3 |
|  |  | 69.0^34^ | / | 0.1 M KHCO3 | MEA | 10.1038/s41929-025-01411-9 |
| Ga | 80.0 | 90.0^53^ | / | 1 M KOH | Flow-cell | 10.1038/s41467-024-51466-8 |
|  |  | 82.0^51^ | -1.07 | 1 M KOH | Flow-cell | 10.1021/jacs.2c12743 |
|  |  | 75.0^49^ | -1.00 | 1 M KOH | Flow-cell | 10.1021/jacs.3c06697 |
|  |  | 73.0^54^ | / | 1 M KOH | Flow-cell | 10.1002/adfm.202404274 |
| Sn | 72.0 | 96.0^55^ | / | 3 M KOH | MEA | 10.1038/s41929-025-01301-0 |
|  |  | 79.0^56^ | -0.51 | 1 M KOH | Flow-cell | 10.1002/smll.202409259 |
|  |  | 50.0^32^ | -0.74 | 1 M KOH | Flow-cell | 10.1126/sciadv.aaz6844 |
|  |  | 61.0^57^ | -1.39 | 0.5M K_2_SO_4_ | Flow-cell | 10.1016/j.apcatb.2025.125584 |
| Pb | 84.0 | 84.0^58^ | -1.10 | 3 M KOH | Flow-cell | 10.1039/d3gc01506h |
| Bi | 72.0 | 72.0^59^ | -1.10 | 1 M KOH | Flow-cell | 10.1002/anie.202303048 |
| In | 85.0 | 85.0^34^ | / | 0.1 M KHCO3 | MEA | 10.1038/s41929-025-01411-9 |
| Ge | 76.0 | 76.0^51^ | -1.07 | 1 M KOH | Flow-cell | 10.1021/jacs.2c12743 |
| Sb | 78.0 | 76.0^60^ | -1.10 | 1 M KOH | Flow-cell | 10.1021/jacs.4c10472 |
|  |  | 80.0^61^ | -1.00 | 1 M KOH | Flow-cell | 10.1002/adma.202500343 |
| Sc | 75.0 | 78.0^36^ | -0.60 | 1 M KOH | Flow-cell | 10.1002/smll.202401656 |
|  |  | 72.0^62^ | -1.35 | 1 M KOH | Flow-cell | 10.1002/adfm.202415940 |
| La | 62.0 | 86.0^63^ | / | 0.05M H_2_SO_4_  + 3MKCl | Flow-cell | 10.1038/s41467-024-49308-8 |
|  |  | 80.0^64^ | -1.37 | 1 M KCl | Flow-cell | 10.1016/j.apcatb.2024.124839 |
|  |  | 19.0^65^ | -1.72 | 1 M KOH | Flow-cell | 10.1021/jacs.2c12006 |
| Ce | 76.0 | 60.0^66^ | -1.20 | 1 M KOH | Flow-cell | 10.1002/anie.202419796 |
|  |  | 93.0^67^ | -0.80 | 3 M KOH | Flow-cell | 10.1038/s41467-025-58109-6 |
| Pr | 67.0 | 71.0^68^ | -1.10 | 1 M KOH | Flow-cell | 10.1021/acs.nanolett.4c05719 |
|  |  | 60.0^63^ | / | 0.05M H_2_SO_4_  + 3MKCl | Flow-cell | 10.1038/s41467-024-49308-8 |
|  |  | 71.0^69^ | -1.60 | 1 M KOH | Flow-cell | 10.1021/jacs.5c02896 |
| Sm | 32.0 | 32.0^65^ | -1.72 | 1 M KOH | Flow-cell | 10.1021/jacs.2c12006 |
| Gd | 81.0 | 81.0^70^ | -0.80 | 2 M KOH | Flow-cell | 10.1021/jacs.3c02428 |
| Tb | 72.0 | 72.0^63^ | / | 0.05 M H_2_SO_4_  + 3MKCl | Flow-cell | 10.1038/s41467-024-49308-8 |
| Er | 68.0 | 68.0^63^ | / | 0.05M H_2_SO_4_  + 3MKCl | Flow-cell | 10.1038/s41467-024-49308-8 |
| Ag+Ni | 93.0 | 93.0^71^ | -1.38 | 1 M KOH | Flow-cell | 10.1002/anie.202501833 |

**Table S1-2.** Average experimental Faraday efficiency of CO_2_RR products on Cu-based SAAs.

| M | C_2+_ | C_1_ | C_2_H_4_ | C_2_H_5_OH | CH_3_COOH | C_3_-OH | CH_4_ | HCOOH | CO | H_2_ |
| --- | --- | --- | --- | --- | --- | --- | --- | --- | --- | --- |
| Cu | 16 | 64 | 8 | 3 | 1 | 0 | 46 | 12 | 6 | 16 |
| Ag | 80 | 8 | 37 | 28 | 13 | 3 | 1 | 1 | 7 | 10 |
| Zn | 89 | 2 | 56 | 21 | 5 | 8 | 0 | 1 | 1 | 9 |
| Cd | 97 | 0 | 10 | 18 | 64 | 4 | 0 | 0 | 0 | 0 |
| Ga | 80 | 10 | 37 | 24 | 14 | 6 | 0 | 2 | 9 | 10 |
| Pb | 84 | 7 | 33 | 43 | 9 | 0 | 0 | 6 | 1 | 5 |
| Bi | 73 | 16 | 48 | 15 | 1 | 7 | 0 | 3 | 13 | 13 |
| In | 85 | 4 | 85 | 0 | 0 | 0 | 0 | 4 | 0 | 12 |
| Ge | 76 | 11 | 47 | 20 | 0 | 8 | 0 | 5 | 7 | 14 |
| Sn | 71 | 13 | 28 | 7 | 18 | 18 | 9 | 0 | 5 | 13 |
| Sb | 78 | 10 | 65 | 13 | 1 | 3 | 0 | 1 | 9 | 7 |
| Al | 80 | 8 | 56 | 10 | 9 | 5 | 0 | 2 | 6 | 11 |
| Au | 71 | 13 | 31 | 17 | 15 | 8 | 0 | 3 | 9 | 16 |
| Pd | 66 | 19 | 27 | 14 | 18 | 5 | 9 | 5 | 9 | 12 |
| Pt | 53 | 15 | 34 | 9 | 10 | 4 | 20 | 1 | 12 | 27 |
| Ni | 52 | 13 | 24 | 8 | 22 | 4 | 4 | 16 | 7 | 24 |
| Rh | 78 | 12 | 61 | 13 | 1 | 3 | 0 | 3 | 9 | 9 |
| Ir | 74 | 12 | 57 | 14 | 1 | 3 | 0 | 2 | 10 | 10 |
| Ru | 71 | 14 | 51 | 16 | 1 | 4 | 0 | 5 | 10 | 13 |
| Mn | 92 | 2 | 4 | 68 | 14 | 6 | 2 | 0 | 0 | 2 |
| Co | 67 | 5 | 21 | 11 | 31 | 4 | 0 | 1 | 5 | 27 |
| Mo | 86 | 9 | 66 | 19 | 0 | 2 | 0 | 0 | 9 | 8 |
| Sc | 75 | 27 | 42 | 29 | 0 | 4 | 1 | 3 | 23 | 10 |
| La | 62 | 28 | 36 | 19 | 3 | 4 | 22 | 2 | 5 | 10 |
| Ce | 76 | 14 | 24 | 12 | 38 | 2 | 5 | 3 | 7 | 24 |
| Pr | 67 | 13 | 31 | 31 | 4 | 1 | 3 | 1 | 8 | 20 |
| Sm | 32 | 41 | 20 | 12 | / | / | 39 | / | 1 | 13 |
| Gd | 81 | 7 | 42 | 32 | 2 | 5 | 0 | 1 | 6 | 15 |
| Tb | 72 | 18 | 28 | 28 | 14 | 2 | 4 | 3 | 12 | 12 |
| Er | 68 | 21 | 30 | 26 | 11 | 2 | 6 | 3 | 12 | 13 |

**Table S1-3.** Experimental Faraday efficiency of CO_2_RR products on Cu-based SAAs.

| M | FE | | | | | | | | DOI |
| --- | --- | --- | --- | --- | --- | --- | --- | --- | --- |
|  | C_2_H_4_ | C_2_H_5_OH | CH_3_COOH | C_3_-OH | CH_4_ | HCOOH | CO | H_2_ |  |
| Cu | 8 | 3 | 1 | 0 | 46 | 12 | 6 | 16 | 10.1016/S1381-1169(03)00016-5 |
| Au | 10 | 8 | 39 | 0 | 1 | 0 | 0 | 38 | 10.1021/acscatal.2c06145 |
|  | 43 | 25 | 1 | 7 | 0 | 2 | 14 | 7 | 10.1038/s44286-023-00018-w |
|  | 41 | 25 | 20 | 6 | 0 | 0 | 0 | 2 | 10.1038/s41467-024-44727-z |
|  | 28 | 10 | 0 | 18 | 0 | 12 | 23 | 14 | 10.1021/jacs.3c11013 |
| Ag | 36 | 41 | 6 | 0 | 4 | 0 | 4 | 6 | 10.1021/jacs.9b02945 |
|  | 40 | 48 | 3 | 4 | 0 | 2 | 3 | 0 | 10.1038/s41467-023-41871-w |
|  | 14 | 56 |  |  |  |  |  |  | 10.1038/s41467-024-54636-w |
|  | 68 | 13 | 0 | 2 | 0 | 0 | 8 | 7 | 10.1007/s11426-024-2292-3 |
|  | 47 | 19 | 2 | 4 | 0 | 5 | 1 | 16 | 10.1002/adma.202411498 |
|  | 18 | 9 | 40 | 5 |  |  |  | 19 | 10.1021/jacs.4c11276 |
|  | 45 | 23 | 1 | 5 |  | 2 | 14 | 9 | 10.1038/s44286-023-00018-w |
|  | 53 | 24 | 6 | 0 | 0 | 3 | 7 | 11 | 10.1021/jacs.5c03057 |
|  | 21 | 50 |  |  |  |  | 16 | 6 | 10.1002/adfm.202503497 |
|  | 25 | 35 | 36 | 2 | 0 | 0 | 0 | 7 | 10.1002/anie.202507062 |
|  | 24 | 4 | 37 | 5 | 3 |  |  | 13 | 10.1126/sciadv.aaz6844 |
|  | 62 | 5 | 0 | 0 | 3 | 0 | 8 | 17 | 10.1038/s41929-025-01411-9 |
|  | 24 | 42 | 9 | 5 | 1 | 2 | 6 | 13 | 10.1002/anie.202408873 |
| Pd | 21 | 0 | 0 | 0 | 60 | 0 | 1 | 17 | 10.1038/s41467-023-38777-y |
|  | 32 | 26 | 2 | 6 |  | 4 | 9 | 10 | 10.1002/smll.202302530 |
|  | 14 | 4 | 60 | 3 | 2 |  |  | 12 | 10.1021/jacs.4c11276 |
|  | 0 | 0 | 47 | 0 | 3 | 23 | 8 | 18 | 10.1021/jacs.4c07711 |
|  | 54 | 23 | 2 | 2 | 1 | 2 | 5 | 12 | 10.1002/anie.202400439 |
|  | 46 | 23 | 2 | 6 | 0 | 3 | 12 | 8 | 10.1038/s44286-023-00018-w |
|  | 8 | 11 | 38 | 28 | 0 | 0 | 0 | 9 | 10.1038/s41467-024-44727-z |
|  | 32 | 32 | 0 | 3 | 3 | 7 | 25 | 0 | 10.1002/smll.202401656 |
|  | 47 | 20 | 0 | 5 | 1 | 0 | 12 | 17 | 10.1016/j.apcatb.2025.125412 |
|  | 20 | 7 | 35 | 3 | 8 |  |  | 17 | 10.1126/sciadv.aaz6844 |
| Pt | 32 | 0 | 0 | 0 | 20 | 0 | 17 | 27 | 10.1038/s41467-023-38777-y |
|  | 20 | 8 | 20 | 9 |  |  |  | 27 | 10.1021/jacs.4c11276 |
|  | 51 | 20 |  |  |  | 2 | 7 |  | 10.1002/anie.202424749 |
| Ir | 57 | 14 | 1 | 3 | 0 | 2 | 10 | 10 | 10.1038/s44286-023-00018-w |
| Rh | 61 | 13 | 1 | 3 | 0 | 3 | 9 | 9 | 10.1038/s44286-023-00018-w |
| Ru | 51 | 16 | 1 | 4 | 0 | 5 | 10 | 13 | 10.1038/s44286-023-00018-w |
| Mn | 4 | 68 | 14 | 6 | 2 | 0 | 0 | 2 | 10.1021/acs.nanolett.5c01205 |
| Co | 43 | 12 | 1 | 5 | 0 | 3 | 15 | 20 | 10.1021/acsenergylett.3c00418 |
|  | 19 | 4 | 30 | 4 |  |  |  | 33 | 10.1021/jacs.4c11276 |
|  | 20 | 9 | 19 | 4 | 0 | 0 | 0 | 48 | 10.1038/s41467-024-44727-z |
|  | 0 | 18 | 72 | 4 | 0 | 0 | 0 | 7 | 10.1039/D4EE06192F |
| Ni | 15 | 6 |  |  |  | 25 | 4 | 36 | 10.1016/j.apcatb.2021.120030 |
|  | 40 | 15 | 5 | 3 | 4 | 6 | 10 | 10 | 10.1021/acsami.1c19224 |
|  | 20 | 4 | 20 | 4 |  |  |  | 40 | 10.1021/jacs.4c11276 |
|  | 23 | 8 | 42 | 4 | 3 |  |  | 11 | 10.1126/sciadv.aaz6844 |
| Mo | 66 | 19 | 0 | 2 | 0 | 0 | 9 | 8 | 10.1002/adma.202412658 |
| Zn | 73 | 15 | 1 | 3 | 0 | 2 | 2 | 8 | 10.1038/s41467-023-36926-x |
|  | 39 | 26 | 9 | 12 | 0 | 0 | 0 | 10 | 10.1002/anie.202420283 |
| Cd | 10 | 18 | 64 | 4 | 0 | 0 | 0 | 0 | 10.1038/s41467-024-44727-z |
| Al | 51 | 20 | 0 | 6 | 0 | 3 | 4 | 16 | 10.1021/jacs.2c12743 |
|  | 27 | 2 | 48 | 7 | 0 | 1 | 10 | 0 | 10.1021/jacs.3c06697 |
|  | 68 | 13 | 0 | 2 | 0 | 0 | 8 | 7 | 10.1007/s11426-024-2292-3 |
|  | 80 | 0 | 0 | 13 | 0 | 0 | 3 | 8 | 10.1038/s41586-020-2242-8 |
|  | 64 | 5 | 0 | 0 | 2 | 3 | 6 | 16 | 10.1038/s41929-025-01411-9 |
|  | 45 | 22 | 5 | 0 | 0 | 2 | 5 | 18 | 10.1016/j.apsusc.2022.152518 |
| Ga | 45 | 28 | 0 | 8 | 0 | 2 | 6 | 10 | 10.1021/jacs.2c12743 |
|  | 4 | 30 | 36 | 5 | 0 | 3 | 14 | 14 | 10.1021/jacs.3c06697 |
|  | 47 | 20 | 3 | 3 | 0 | 2 | 14 | 12 | 10.1002/adfm.202404274 |
|  | 53 | 19 | 17 | 9 | 0 | 0 | 0 | 5 | 10.1038/s41467-024-51466-8 |
| Sn | 51 | 1 | 5 | 21 | 3 | 0 | 16 | 6 | 10.1002/smll.202409259 |
|  | 23 | 14 | 11 | 47 | 0 | 0 | 0 | 3 | 10.1038/s41929-025-01301-0 |
|  | 19 | 4 | 25 | 2 | 4 |  |  | 36 | 10.1126/sciadv.aaz6844 |
|  | 19 | 9 | 30 | 2 | 29 | 0 | 0 | 7 | 10.1016/j.apcatb.2025.125584 |
| Pb | 33 | 43 | 9 | 0 | 0 | 6 | 1 | 5 | 10.1039/d3gc01506h |
| Bi | 48 | 15 | 1 | 7 | 0 | 3 | 13 | 13 | 10.1002/anie.202303048 |
| In | 85 | 0 | 0 | 0 | 0 | 4 | 0 | 12 | 10.1038/s41929-025-01411-9 |
| Ge | 47 | 20 | 0 | 8 | 0 | 5 | 7 | 14 | 10.1021/jacs.2c12743 |
| Sb | 42 | 26 | 3 | 6 | 0 | 1 | 18 | 7 | 10.1021/jacs.4c10472 |
|  | 88 | 0 | 0 | 0 | 0 | 0 | 0 | 8 | 10.1002adma.202500343 |
| Sc | 53 | 19 | 0 | 0 | 0 | 2 | 13 | 10 | 10.1002/adfm.202415940 |
|  | 32 | 39 | 0 | 7 | 1 | 5 | 34 |  | 10.1002/smll.202401656 |
| La | 14 | 5 | 0 | 0 | 64 | 0 | 2 | 14 | 10.1021/jacs.2c12006 |
|  | 42 | 30 | 10 | 5 | 1 | 3 | 6 | 7 | 10.1038/s41467-024-49308-8 |
|  | 52 | 22 | 0 | 7 | 0 | 3 | 7 | 9 | 10.1016/j.apcatb.2024.124839 |
| Ce | 40 | 14 | 5 | 0 | 8 | 6 | 13 | 40 | 10.1002/anie.202419796 |
|  | 8 | 11 | 71 | 3 | 2 | 0 | 0 | 8 | 10.1038/s41467-025-58109-6 |
| Pr | 26 | 22 | 9 | 3 | 5 | 3 | 16 | 16 | 10.1038/s41467-024-49308-8 |
|  | 3 | 67 | 0 | 0 | 3 | 0 | 1 | 24 | 10.1021/acs.nanolett.4c05719 |
|  | 64 | 3 | 4 | 0 | 2 | 1 | 8 | 21 | 10.1021jacs.5c02896 |
| Sm | 20 | 12 |  |  | 39 |  | 1 | 13 | 10.1021/jacs.2c12006 |
| Gd | 42 | 32 | 2 | 5 | 0 | 1 | 6 | 15 | 10.1021/jacs.3c02428 |
| Tb | 28 | 28 | 14 | 2 | 4 | 3 | 12 | 12 | 10.1038/s41467-024-49308-8 |
| Er | 30 | 26 | 11 | 2 | 6 | 3 | 12 | 13 | 10.1038/s41467-024-49308-8 |
| Ag+Ni | 32 | 46 | 12 | 3 | 0 | 0 | 3 | 5 | 10.1002/anie.202501833 |

**Table S2.** The calculated formation energy (E_for_), segregation energies (E_seg_) and aggregation energies (E_agg_) of Cu-based SAAs. Dissolution potential (U_diss_) of metals, number of transferred electrons (N_e_) during the dissolution and standard dissolution potential (U^0^_diss_) of metal atoms are also listed. The calculated formation energy (E^for^_adatom_) of adatoms induced by H*, O*, OH* and CO*.

| M | E_for_ (eV) | | E_seg_ (eV) | E_agg_ (eV) | U^0^_diss_  (V) | N_e_ | U_diss_  (V VS. RHE) | E_for_^adatom^ (eV) | | | |
| --- | --- | --- | --- | --- | --- | --- | --- | --- | --- | --- | --- |
|  | 111 | 100 |  |  |  |  |  | H* | O* | OH* | CO* |
| Sc | -0.55 | -0.57 | -0.08 | 0.75 | -2.08 | 3 | -1.48 | 0.95 | 1.06 | 0.90 | 1.64 |
| Ti | 0.42 | 0.64 | 0.31 | 0.03 | -1.63 | 2 | -1.42 | 2.25 | 1.15 | 1.42 | 2.16 |
| V | 1.06 | 1.11 | 0.25 | 0.01 | -1.18 | 2 | -1.29 | 3.01 | 1.87 | 1.92 | 2.76 |
| Cr | 0.77 | 2.54 | 0.14 | 0.18 | -0.91 | 2 | -0.88 | 3.97 | 2.06 | 2.53 | 3.27 |
| Mn | 0.05 | 0.10 | 0.10 | 0.15 | -1.19 | 2 | -0.80 | 4.27 | 2.24 | 2.99 | 7.41 |
| Fe | 0.82 | 0.98 | 0.16 | -0.25 | -0.45 | 2 | -0.45 | 2.91 | 2.10 | 2.36 | 2.80 |
| Co | 0.91 | 1.39 | 0.14 | -0.27 | -0.28 | 2 | -0.32 | 2.65 | 2.43 | 1.51 | 2.26 |
| Ni | 0.25 | 0.43 | 0.19 | 0.01 | -0.26 | 2 | 0.03 | 1.89 | 2.39 | 1.36 | 1.61 |
| Zn | -0.49 | -0.49 | -0.11 | 0.10 | -0.76 | 2 | -0.10 | 0.40 | 2.35 | 0.59 | 0.74 |
| Ga | -0.81 | -0.71 | -0.31 | 0.49 | -0.55 | 3 | 0.14 | 0.44 | 1.58 | 0.46 | 4.62 |
| Ge | -0.51 | -0.65 | -0.51 | 0.24 | 0.12 | 4 | 0.67 | 0.63 | 0.90 | 0.66 | 4.88 |
| Al | -0.81 | -0.64 | 0.08 | 0.47 | -1.68 | 3 | -0.99 | 0.81 | 2.00 | 0.64 | 1.56 |
| Y | -0.71 | -0.86 | -0.82 | 1.23 | -2.37 | 3 | -1.72 | 0.96 | 1.20 | 0.91 | 3.70 |
| Zr | 0.30 | 0.40 | 0.10 | 0.35 | -1.45 | 4 | -1.11 | 1.88 | 1.33 | 1.88 | 4.75 |
| Nb | 1.51 | 1.77 | 0.37 | -0.89 | -1.10 | 3 | -1.19 | -0.84 | -0.33 | -0.31 | 3.37 |
| Mo | 2.21 | 2.42 | 0.36 | -1.49 | -0.20 | 3 | -0.52 | 3.05 | -0.51 | -0.36 | 4.36 |
| Tc | 1.94 | 2.22 | 0.29 | -1.39 | / | / | / | 3.12 | 2.23 | 2.03 | 7.50 |
| Ru | 1.43 | 1.77 | 0.30 | -0.45 | 0.46 | 2 | 0.15 | 2.76 | 1.97 | 1.81 | 7.94 |
| Rh | 0.23 | 0.52 | 0.12 | 0.11 | 0.60 | 2 | 0.90 | 2.53 | 1.55 | 1.63 | 7.87 |
| Pd | -0.45 | -0.36 | -0.20 | 0.10 | 0.95 | 2 | 1.59 | 1.88 | 2.80 | 1.77 | 7.03 |
| Ag | -0.03 | -0.17 | -0.56 | 0.04 | 0.80 | 1 | 1.24 | 0.79 | 3.04 | 1.23 | 5.52 |
| Cd | -0.36 | -0.46 | -0.84 | 0.15 | -0.40 | 2 | 0.19 | 0.04 | 2.46 | -0.01 | 4.81 |
| In | -0.65 | -0.64 | -0.94 | 0.51 | -0.34 | 3 | 0.29 | 0.31 | 1.89 | 0.30 | 4.78 |
| Sn | -0.65 | -0.85 | -1.10 | 0.34 | -0.14 | 2 | 0.60 | 0.87 | 0.31 | 0.94 | 5.08 |
| Sb | -0.73 | -1.07 | -1.41 | 0.47 | / | / | / | 0.47 | 1.25 | 1.67 | 5.96 |
| Hf | 0.34 | 0.55 | 0.32 | 0.45 | -1.55 | 4 | -1.22 | -0.27 | 1.30 | 1.75 | 4.54 |
| Ta | 1.66 | 2.14 | 0.54 | -0.51 | -0.60 | 3 | -0.74 | -0.38 | -0.35 | -0.36 | 3.21 |
| W | 2.74 | 3.23 | 0.51 | -1.43 | 0.10 | 3 | -0.40 | -0.55 | -0.34 | -0.27 | 4.29 |
| Re | 2.56 | 3.07 | 0.43 | -1.41 | 0.30 | 3 | -0.14 | 3.15 | 2.61 | 2.14 | 7.56 |
| Os | 2.04 | 2.53 | 0.35 | -0.81 | / | / | / | 2.96 | -0.20 | 1.89 | 8.07 |
| Ir | 0.57 | 0.98 | 0.17 | -0.05 | 1.16 | 3 | 1.38 | 2.85 | 1.96 | 1.65 | 8.18 |
| Pt | -0.61 | -0.37 | -0.17 | 0.14 | 1.18 | 2 | 1.90 | 2.13 | 1.09 | 1.35 | 7.36 |
| Au | -0.52 | -0.55 | -0.61 | 0.12 | 1.50 | 3 | 2.09 | 0.96 | 2.38 | 0.54 | 5.69 |
| Pb | -0.38 | -0.62 | -1.89 | 0.30 | -0.13 | 2 | 0.48 | -0.05 | 0.05 | 0.71 | 5.27 |
| Bi | -0.51 | -0.88 | -2.05 | 0.36 | 0.31 | 3 | 0.89 | 0.42 | 0.74 | 0.91 | 5.16 |

**Table S3.** The thermodynamic reaction energies of the C-C coupling and CO* hydrogenation on Cu-based SAAs.

| M | CO*→CHO* | | CO*→COH* | | CO*+CO* | | | CO*+CHO* | | | CHO*+CHO* | |
| --- | --- | --- | --- | --- | --- | --- | --- | --- | --- | --- | --- | --- |
|  | M | Cu_1_ | M | Cu_1_ | M+  Cu_1_ | Cu_1_+ Cu_1_ | (CO*_M_)  Cu_1_+  Cu_1_ | M+  Cu_1_ | Cu_1_+M | Cu_1_+ Cu_1_ | M+  Cu_1_ | Cu_1_+  Cu_1_ |
| 3d transition metals | | | | | | | | | | | | |
| Sc | 0.14 | -0.85 | 0.80 | 0.60 | 0.32 | 0.32 | 0.39 | -1.35 | -0.58 | -0.96 | F | B |
| Ti | 0.35 | 0.50 | 0.70 | 1.02 | 0.11 | -0.77 | 0.61 | -1.40 | B | F | B | B |
| V | 0.68 | 0.56 | 0.85 | 1.09 | 0.58 | -0.31 | 0.20 | -0.80 | B | B | B | B |
| Cr | 0.86 | 0.62 | 0.87 | 1.14 | 0.95 | 0.42 | 0.67 | 0.02 | B | -0.04 | B | -1.46 |
| Mn | B | 0.57 | 1.09 | 1.13 | 1.07 | 0.62 | 0.92 | 0.10 | B | B | B | -1.46 |
| Fe | B | 0.60 | 1.34 | 1.15 | 1.80 | 1.16 | 1.67 | 0.68 | B | -0.01 | B | -1.38 |
| Co | B | 0.87 | 2.30 | 0.70 | 2.32 | 0.82 | 1.48 | 0.62 | B | -0.01 | B | -1.16 |
| Ni | 1.02 | 0.71 | 1.33 | 1.14 | 1.97 | 1.59 | 1.42 | 0.38 | 0.51 | 0.00 | -0.93 | -1.42 |
| Cu | 0.70 | 0.70 | 1.16 | 1.16 | 1.60 | / | C | / | 0.03 | / | / | -1.41 |
| Zn | 0.29 | 0.60 | 0.66 | 1.19 | 0.89 | 1.44 | C | -0.31 | -0.22 | -0.03 | -1.71 | -1.46 |
| 4d transition metals | | | | | | | | | | | | |
| Y | -0.07 | -0.78 | 2.09 | 0.77 | 0.21 | 0.25 | 0.38 | -1.67 | -0.53 | -0.56 | D | B |
| Zr | -0.13 | -1.08 | 0.87 | 0.05 | 0.69 | 0.55 | 1.02 | -0.31 | -0.35 | B | B | B |
| Nb | 0.46 | 0.44 | 0.74 | -0.48 | 1.71 | -0.90 | 2.04 | -1.09 | 0.70 | B | B | B |
| Mo | 0.56 | 0.53 | 0.89 | -0.63 | 1.38 | -0.03 | 0.36 | 0.02 | B | B | B | B |
| Tc | 1.03 | 0.56 | 0.88 | -0.83 | 2.32 | B | B | 0.47 | B | -1.03 | B | -1.32 |
| Ru | B | 0.59 | 1.18 | -0.67 | 2.79 | B | 1.55 | 0.74 | B | -1.20 | B | -1.33 |
| Rh | 0.99 | 0.66 | 1.74 | 1.09 | 2.64 | B | 1.49 | 0.48 | 0.95 | -0.04 | -0.43 | -1.40 |
| Pd | 0.70 | 0.71 | 2.34 | 0.88 | 1.79 | 1.50 | 1.36 | 0.10 | 0.54 | -0.01 | -0.75 | -1.44 |
| Ag | 0.40 | 0.67 | 2.42 | 1.16 | 1.33 | 1.59 | C | -0.15 | -0.14 | 0.01 | -1.45 | -1.47 |
| Cd | 0.56 | 0.56 | 2.65 | 1.88 | A | 1.49 | C | A | -0.27 | 0.02 | -1.66 | -1.42 |
| 5d transition metals | | | | | | | | | | | | |
| Hf | -0.12 | -1.19 | 0.75 | 0.03 | 0.58 | -0.39 | 0.98 | -1.23 | -0.09 | B | F | B |
| Ta | 0.05 | 0.41 | 0.70 | 0.89 | 0.60 | -0.69 | 0.04 | F | B | B | B | B |
| W | 0.58 | 0.71 | 0.83 | 1.13 | 1.34 | -0.23 | 0.44 | 0.01 | B | B | B | -1.17 |
| Re | 1.16 | 0.53 | 0.84 | 0.93 | 2.14 | B | 1.92 | 0.54 | B | 0.17 | B | -1.26 |
| Os | B | 0.58 | 1.06 | 0.94 | 3.03 | B | 1.79 | 0.81 | B | 0.17 | B | -1.30 |
| Ir | 1.10 | 0.64 | 1.60 | 1.06 | 1.37 | -0.39 | 3.04 | 0.56 | 1.15 | -0.04 | -0.24 | -1.40 |
| Pt | 0.69 | 0.68 | 2.23 | 1.11 | 1.85 | 1.63 | 1.40 | 0.08 | 0.00 | -0.02 | -0.33 | -1.43 |
| Au | 0.14 | 0.65 | 2.13 | 1.15 | 1.32 | 1.61 | C | 0.75 | 1.06 | 0.89 | -0.22 | -0.57 |
| Post-transition metal | | | | | | | | | | | | |
| Al | 0.33 | 0.63 | 1.63 | 1.37 | 0.42 | 1.41 | C | -0.30 | -0.59 | -0.02 | -2.10 | -1.47 |
| Ga | 0.45 | 0.64 | 1.00 | 1.08 | A | 1.42 | C | A | -0.18 | -0.01 | -1.61 | -1.48 |
| In | 0.53 | 0.62 | 4.79 | 3.96 | A | 1.46 | C | A | -0.30 | 0.02 | -1.64 | -1.38 |
| Sn | 0.28 | 0.62 | 2.42 | 1.18 | A | 1.42 | C | A | -0.12 | -0.01 | -1.52 | -1.46 |
| Pb | 0.52 | 0.61 | 2.63 | 1.92 | A | 1.53 | C | A | -0.30 | 0.12 | -1.66 | -1.49 |
| Bi | 0.36 | 0.62 | 2.43 | 1.66 | A | 1.47 | C | A | -0.06 | 0.19 | -1.35 | -1.45 |
| Metalloid | | | | | | | | | | | | |
| Ge | 0.30 | 0.63 | 2.33 | 1.01 | A | 1.38 | C | A | 0.00 | -0.01 | -1.39 | -1.51 |
| Sb | 0.20 | 0.62 | 2.31 | 1.95 | A | 1.25 | C | A | 0.21 | 0.01 | -1.16 | -1.44 |
| Lanthanide metals | | | | | | | | | | | | |
| La | -0.14 | -0.55 | / | / | / | / | / | / | -0.64 | / | / | / |
| Ce | -0.24 | -0.60 | / | / | / | / | / | / | -0.32 | / | / | / |
| Pr | -0.57 | -1.03 | / | / | / | / | / | / | -0.59 | / | / | / |
| Sm | 0.18 | -0.44 | / | / | / | / | / | / | -0.40 | / | / | / |
| Eu | 0.37 | -0.34 | / | / | / | / | / | / | -0.71 | / | / | / |
| Gd | -0.08 | -0.56 | / | / | / | / | / | / | 0.08 | / | / | / |
| Tb | 0.10 | -0.19 | / | / | / | / | / | / | -1.61 | / | / | / |
| Er | 0.25 | -0.44 | / | / | / | / | / | / | 0.54 | / | / | / |
| Yb | 0.48 | -0.39 | / | / | / | / | / | / | -0.69 | / | / | / |
| Lu | 0.05 | -0.57 | / | / | / | / | / | / | -0.59 | / | / | / |
| alkaline earth metals | | | | | | | | | | | | |
| Mg | 0.69 | 0.57 | / | / | / | / | / | / | -0.77 | / | / | / |
| Ca | 0.49 | -0.40 | / | / | / | / | / | / | -0.79 | / | / | / |
| Sr | 0.49 | -0.30 | / | / | / | / | / | / | -0.80 | / | / | / |
| Ba | 0.50 | -0.15 | / | / | / | / | / | / | -0.69 | / | / | / |

[A] CO* desorption

[B] CHO*/COCO* dissociation

[C] E^ads^CO(Cu_1_ site) < CO^ads^CO(M site)

[D] reconstruction

[E] the ΔE(CO*→CHO*) is too high

[F] the adsorption migration to other sites

**Table S4.** The energy barrier of C-C coupling processes on SAAs. [Ea] Ea=ΔE; the ΔE is too high or higher than other C-C coupling.

| M | CO*+CO* | | | CO*+CHO* | | | CHO*+CHO* | |
| --- | --- | --- | --- | --- | --- | --- | --- | --- |
|  | M+  Cu1 | Cu1  +Cu1 | (CO*_M_)  Cu1+Cu1 | M+  Cu1 | Cu1+M | Cu1+  Cu1 | M+  Cu1 | Cu1+  Cu1 |
| 3d transition metals | | | | | | | | |
| Sc | [0.32] | [0.32] | [0.39] | 1.06 | 0.02 | 0.36 | F | B |
| Ti | [0.11] | 0.00 | [0.61] | 1.01 | B | F | B | B |
| V | [0.58] | 0.14 | [0.20] | 0.00 | B | B | B | B |
| Cr | [0.95] | 0.42 | [0.67] | 0.52 | B | 0.79 | B | 0.63 |
| Mn | [1.07] | 0.87 | [0.92] | 1.00 | B | B | B | 0.56 |
| Fe | [1.80] | 1.16 | 1.73 | 0.68 | B | 0.84 | B | 0.55 |
| Co | [2.32] | 0.82 | 1.51 | 0.80 | B | 1.02 | B | E |
| Ni | 1.97 | 1.96 | 1.42 | 1.19 | 1.06 | 0.89 | 0.78 | 0.46 |
| Cu | 1.69 | / | / | / | 0.80 | / | / | 0.52 |
| Zn | 1.27 | 1.67 | C | 0.65 | 0.80 | 0.86 | 0.50 | 0.47 |
| 4d transition metals | | | | | | | | |
| Y | [0.21] | 1.66 | 1.64 | 0.54 | 0.11 | 0.55 | D | B |
| Zr | [0.69] | [0.55] | [1.02] | F | 0.19 | B | B | B |
| Nb | [1.71] | 0.17 | [2.04] | 1.09 | [0.70] | B | B | B |
| Mo | [1.38] | 0.23 | [0.36] | 1.10 | B | B | B | B |
| Tc | [2.32] | B | B | 0.89 | B | 0.80 | B | 0.58 |
| Ru | [2.79] | B | [1.55] | 0.82 | B | 0.80 | B | 0.53 |
| Rh | 2.83 | B | 1.51 | 1.16 | 1.25 | 0.89 | 0.37 | 0.47 |
| Pd | 1.90 | 2.04 | 1.36 | 1.10 | 0.95 | 0.97 | 0.68 | 0.55 |
| Ag | 1.66 | 1.79 | C | 1.03 | 0.76 | 0.99 | 0.43 | 0.55 |
| Cd | A | [1.49] | C | A | 0.67 | 0.97 | 0.58 | 0.60 |
| 5d transition metals | | | | | | | | |
| Hf | 2.04 | 0.10 | 2.15 | 1.31 | 0.14 | B | F | B |
| Ta | [0.60] | 0.54 | 0.42 | F | B | B | B | B |
| W | 1.93 | 0.44 | 0.62 | 0.88 | B | B | B | 0.65 |
| Re | [2.14] | B | [1.92] | 0.95 | B | 1.00 | B | 0.58 |
| Os | [3.03] | B | [1.79] | 0.87 | B | 0.97 | B | 0.53 |
| Ir | 1.37 | F | 3.04 | 0.98 | 1.40 | 0.89 | E | 0.45 |
| Pt | 1.96 | 2.00 | 1.40 | 1.17 | 1.53 | 0.98 | 0.93 | 0.48 |
| Au | 2.69 | 1.98 | C | 0.74 | 1.09 | 1.02 | 0.63 | 0.53 |
| Post-transition metal | | | | | | | | |
| Al | 1.20 | 1.74 | C | 0.61 | 0.87 | 0.78 | 0.57 | 0.48 |
| Ga | A | 1.77 | C | A | 0.79 | 0.90 | 0.56 | 0.45 |
| In | A | 1.81 | C | A | 0.60 | 0.90 | 0.57 | 0.63 |
| Sn | A | 1.42 | C | A | 0.99 | 0.84 | 0.63 | 0.63 |
| Pb | A | 2.02 | C | A | 0.68 | 1.01 | 0.54 | 0.67 |
| Bi | A | 1.50 | C | A | 1.03 | 1.16 | 0.65 | 0.66 |
| Metalloid | | | | | | | | |
| Ge | A | 1.48 | C | A | 1.01 | 0.93 | 0.59 | 0.54 |
| Sb | A | 1.39 | C | A | 1.20 | 0.91 | 0.73 | 0.64 |
| Lanthanide metals | | | | | | | | |
| La | / | / | / | / | 0.21 | / | / | / |
| Ce | / | / | / | / | 0.30 | / | / | / |
| Pr | / | / | / | / | 0.12 | / | / | / |
| Sm | / | / | / | / | 0.10 | / | / | / |
| Eu | / | / | / | / | 0.00 | / | / | / |
| Gd | / | / | / | / | 0.08 | / | / | / |
| Tb | / | / | / | / | 0.00 | / | / | / |
| Er | / | / | / | / | 0.79 | / | / | / |
| Yb | / | / | / | / | 0.00 | / | / | / |
| Lu | / | / | / | / | 0.00 | / | / | / |
| alkaline earth metals | | | | | | | | |
| Mg | / | / | / | / | 0.28 | / | / | / |
| Ca | / | / | / | / | 0.16 | / | / | / |
| Sr | / | / | / | / | 0.09 | / | / | / |
| Ba | / | / | / | / | 0.15 | / | / | / |

[A] CO* desorption

[B] CHO*/COCO* dissociation

[C] E^ads^CO(Cu1 site) < CO^ads^CO(M site)

[D] reconstruction

[E] the ΔE(CO*→CHO*) is too high

[F] the adsorption migration to other sites

**Table S5.** The C-C coupling mechanisms in diverse Cu-based SAAs. The reaction mechanism color code follows Figure 4, and the element color code follows Figure 5b.

| CHO*_M_-CHO*_Cu1_  (eV) | C-C coupling reaction mechanism | | M |
| --- | --- | --- | --- |
| > 0.33 | Path 7 | CO_Cu1_+CHO_M_ | Sc Y Zr Hf La Ce Pr Sm Eu Gd Tb Er Yb |
| 0.0 ~ 0.33 |  |  | Ga Ge Cd Pb Bi In Ag Zn |
| -0.3 ~ 0.0 |  |  | Al  Au Sn Sb |
| -1.2 ~ -0.3 | Path 2 | (CO_M_) CO_Cu1_+CO_Cu1_ | Pd Ni Pt Rh Ta |
| -1.5 ~ -1.2 | Path 9 | CO_Cu1_+COH_M_ | Tc Re |
| CO*_M_-CO*_Cu1_ |  | C-C coupling reaction mechanism | M |
| -1.7 ~ -0.7 | Path 1 | CO_Cu1_+CO_Cu1_ | Ti V Nb Mo W Cr Mn  Fe  Co |
| -2.2 ~ -1.7 | Path 5 | CO_M_+CO_Cu1_ | Ir |
|  | Path 3 | CO_Cu1_+CHO_Cu1_ | Ru |
|  | Path 6 | CO_M_+CHO_Cu1_ | Os |

**Table S6.** The Ea-C_2+_ (eV) for C-C coupling processes on diverse Cu-based SAAs.

| 3d transition metals | | 4d transition metals | | 5d transition metals | | Post-transition metal | | Lanthanide metals | |
| --- | --- | --- | --- | --- | --- | --- | --- | --- | --- |
| Sc | 0.15 | Y | 0.11 | Hf | 0.14 | Al | 1.20 | La | 0.21 |
| Ti | 0.00 | Zr | 0.19 | Ta | 0.42 | Ga | 1.24 | Ce | 0.30 |
| V | 0.14 | Nb | 0.17 | W | 0.44 | In | 1.13 | Pr | 0.12 |
| Cr | 0.42 | Mo | 0.23 | Re | 0.93 | Sn | 1.27 | Sm | 0.28 |
| Mn | 0.87 | Tc | 0.92 | Os | 1.45 | Pb | 1.21 | Eu | 0.37 |
| Fe | 1.16 | Ru | 1.39 | Ir | 1.37 | Bi | 1.39 | Gd | 0.08 |
| Co | 0.82 | Rh | 1.51 | Pt | 1.40 |  |  | Tb | 0.10 |
| Ni | 1.42 | Pd | 1.36 | Au | 1.23 |  |  | Er | 1.03 |
| Cu | 1.50 | Ag | 1.16 |  |  |  |  | Yb | 0.48 |
| Zn | 1.09 | Cd | 1.23 |  |  |  |  | Lu | 0.05 |
| alkaline earth metals | | Mg | 0.97 | Sr | 0.58 | Metalloid | | Ge | 1.31 |
|  |  | Ca | 0.65 | Ba | 0.65 |  |  | Sb | 1.39 |

**Table S7.** The structural descriptor parameter values of Cu-based SAAs and the adsorption energies of CO* and CHO*. X: Electronegativity, N: the group number in the periodic table of elements, n: the number of d electrons in the outermost shell, Y = 8-n.

| M | X | N | n | Y | φ | CO*_M_ | CO*_Cu1_ | CO*_M_-  CO*_Cu1_ | CHO*_M_ | CHO*_Cu1_ | CHO*_M_-  CHO*_Cu1_ |
| --- | --- | --- | --- | --- | --- | --- | --- | --- | --- | --- | --- |
| 3d transition metals | | | | | | | | | | | |
| Sc | 1.36 | 3 | 1 | 0 | 5.64 | -0.53 | -0.33 | -0.21 | -0.39 | -1.18 | 0.79 |
| Ti | 1.54 | 4 | 2 | 0 | 4.46 | -1.01 | -0.30 | -0.71 | -0.66 | 0.20 | -0.86 |
| V | 1.63 | 5 | 3 | 0 | 3.37 | -1.19 | -0.37 | -0.82 | -0.51 | 0.18 | -0.70 |
| Cr | 1.66 | 6 | 5 | 3 | 5.34 | -0.94 | -0.42 | -0.51 | -0.07 | 0.19 | -0.27 |
| Mn | 1.55 | 7 | 5 | 3 | 4.45 | -0.75 | -0.38 | -0.37 | B | 0.19 | B |
| Fe | 1.83 | 8 | 6 | 2 | 2.17 | -1.36 | -0.40 | -0.97 | B | 0.20 | B |
| Co | 1.88 | 9 | 7 | 0 | 0.88 | -1.79 | -0.43 | -1.36 | B | 0.44 | B |
| Ni | 1.91 | 10 | 8 | 0 | 1.91 | -1.44 | -0.47 | -0.97 | -0.42 | 0.24 | -0.66 |
| Cu | 1.90 | 11 | 10 | 0 | 8.10 | -0.48 | -0.48 | 0.00 | 0.22 | 0.22 | 0.00 |
| Zn | 1.65 | 12 | 10 | 0 | 8.35 | 0.16 | -0.38 | 0.55 | 0.46 | 0.21 | 0.24 |
| 4d transition metals | | | | | | | | | | | |
| Y | 1.22 | 3 | 1 | 0 | 5.78 | -0.44 | -0.34 | -0.10 | -0.51 | -1.12 | 0.61 |
| Zr | 1.33 | 4 | 2 | 0 | 4.67 | -0.91 | -0.33 | -0.58 | -1.04 | -1.41 | 0.37 |
| Nb | 1.60 | 5 | 4 | 0 | 3.40 | -1.48 | -0.27 | -1.21 | -1.02 | 0.17 | -1.18 |
| Mo | 2.16 | 6 | 5 | 0 | 1.84 | -1.81 | -0.32 | -1.49 | -1.25 | 0.21 | -1.46 |
| Tc | 1.90 | 7 | 5 | 0 | 1.10 | -2.07 | -0.35 | -1.72 | -1.04 | 0.21 | -1.25 |
| Ru | 2.20 | 8 | 7 | 0 | 0.20 | -2.22 | -0.37 | -1.85 | B | 0.23 | B |
| Rh | 2.28 | 9 | 8 | 0 | 1.28 | -1.82 | -0.42 | -1.40 | -0.83 | 0.24 | -1.07 |
| Pd | 2.20 | 10 | 10 | -2 | 4.20 | -0.93 | -0.45 | -0.48 | -0.23 | 0.26 | -0.49 |
| Ag | 1.93 | 11 | 10 | 0 | 8.07 | 0.00 | -0.44 | 0.44 | 0.40 | 0.23 | 0.17 |
| Cd | 1.69 | 12 | 10 | 0 | 8.31 | A | -0.30 | 0.30 | 0.56 | 0.26 | 0.30 |
| 5d transition metals | | | | | | | | | | | |
| Hf | 1.30 | 4 | 2 | 0 | 4.70 | -0.98 | -0.30 | -0.68 | -1.10 | -1.50 | 0.40 |
| Ta | 1.50 | 5 | 3 | 0 | 3.50 | -1.58 | -0.24 | -1.35 | -1.54 | 0.17 | -1.71 |
| W | 2.36 | 6 | 4 | 0 | 1.64 | -2.09 | -0.48 | -1.61 | -1.50 | 0.24 | -1.74 |
| Re | 1.90 | 7 | 5 | 0 | 1.10 | -2.36 | -0.29 | -2.07 | -1.20 | 0.25 | -1.45 |
| Os | 2.20 | 8 | 6 | 0 | 0.20 | -2.51 | -0.33 | -2.18 | B | 0.25 | B |
| Ir | 2.20 | 9 | 7 | 0 | 1.20 | -2.16 | -0.37 | -1.79 | -1.06 | 0.27 | -1.33 |
| Pt | 2.28 | 10 | 9 | -1 | 3.28 | -1.27 | -0.40 | -0.88 | -0.59 | 0.29 | -0.87 |
| Au | 2.54 | 11 | 10 | 0 | 7.46 | -0.05 | -0.40 | 0.35 | 0.09 | 0.25 | -0.16 |
| Post-transition metal | | | | | | | | | | | |
| Al | 1.61 | 13 | 0 | 0 | 8.39 | -0.05 | -0.31 | 0.26 | 0.28 | 0.32 | -0.04 |
| Ga | 1.81 | 13 | 10 | 0 | 8.19 | A | -0.31 | 0.32 | 0.46 | 0.33 | 0.13 |
| In | 1.78 | 13 | 10 | 0 | 8.22 | A | -0.25 | 0.25 | 0.52 | 0.37 | 0.15 |
| Sn | 1.96 | 14 | 10 | 0 | 8.04 | A | -0.32 | 0.32 | 0.28 | 0.30 | -0.02 |
| Pb | 2.33 | 14 | 10 | 0 | 7.67 | A | -0.30 | 0.30 | 0.52 | 0.31 | 0.21 |
| Bi | 2.02 | 15 | 10 | 0 | 7.98 | A | -0.30 | 0.30 | 0.36 | 0.32 | 0.04 |
| Metalloid | | | | | | | | | | | |
| Ge | 2.01 | 14 | 10 | 0 | 7.99 | A | -0.36 | 0.36 | 0.29 | 0.27 | 0.02 |
| Sb | 2.05 | 15 | 10 | 0 | 7.95 | A | -0.32 | 0.33 | 0.20 | 0.30 | -0.10 |
| Lanthanide metals | | | | | | | | | | | |
| La | 1.10 | 3 | 1 | 0 | 5.90 | -0.28 | -0.43 | 0.14 | -0.42 | -0.98 | 0.56 |
| Ce | 1.12 | 3 | 1 | 0 | 5.88 | -0.41 | -0.66 | 0.25 | -0.65 | -1.26 | 0.60 |
| Pr | 1.13 | 3 | 10 | 0 | 5.87 | -0.41 | -0.53 | 0.12 | -0.98 | -1.56 | 0.58 |
| Sm | 1.17 | 3 | 10 | 0 | 5.83 | -0.40 | -0.47 | 0.07 | -0.22 | -0.91 | 0.68 |
| Eu | 1.20 | 3 | 10 | 0 | 5.80 | -0.26 | -0.35 | 0.09 | 0.11 | -0.69 | 0.80 |
| Gd | 1.20 | 3 | 1 | 0 | 5.80 | -0.35 | -0.46 | 0.10 | -0.43 | -1.02 | 0.59 |
| Tb | 1.10 | 3 | 10 | 0 | 5.80 | -0.32 | -0.37 | 0.06 | -0.22 | -0.56 | 0.34 |
| Er | 1.24 | 3 | 10 | 0 | 5.76 | -0.37 | -0.12 | -0.24 | -0.12 | -0.56 | 0.44 |
| Yb | 1.10 | 3 | 10 | 0 | 5.90 | -0.33 | -0.35 | 0.02 | 0.15 | -0.74 | 0.89 |
| Lu | 1.27 | 3 | 1 | 0 | 5.73 | -0.45 | -0.49 | 0.03 | -0.40 | -1.05 | 0.65 |
| alkaline earth metals | | | | | | | | | | | |
| Mg | 1.31 | 2 | 0 | 0 | 6.69 | -0.17 | -0.38 | 0.21 | 0.52 | 0.19 | 0.33 |
| Ca | 1.00 | 2 | 0 | 0 | 7.00 | -0.26 | -0.36 | 0.10 | 0.23 | -0.75 | 0.98 |
| Ba | 0.89 | 2 | 10 | 0 | 7.11 | -0.16 | -0.35 | 0.19 | 0.34 | -0.50 | 0.84 |
| Sr | 0.95 | 2 | 10 | 0 | 7.05 | -0.21 | -0.33 | 0.12 | 0.28 | -0.63 | 0.90 |

[A] CO* desorption

[B] CHO*/COCO* dissociation

**Table S8.** The adsorption energies of CHO* at the M_1_ and Cu_1_ site with or without M_2_ and the predicted values of Ea-C_2+_ for DSAAs. The use of red font highlights the DSAAs capable of enhancing the value of the original SAAs' $E_{CHO*}^{ads}(M-{Cu}_{1})$. Green font signifies the potential for achieving high selectivity of C_2+_ products.

|  |  | Eads | Eads | ΔE_2_ | ΔE_1_ | ΔE_2_-ΔE_1_ | Ea-C_2+_ |
| --- | --- | --- | --- | --- | --- | --- | --- |
| M_2_ | M_1_ | CHO*_M1_ | CHO*_Cu1_ | CHO*_M1_-  CHO*_Cu1_  with M_2_ | CHO*_M1_-  CHO*_Cu1_  without M_2_ |  |  |
| **Ni** | **Ag** | 0.43 | 0.25 | 0.19 | 0.17 | **0.017** | 1.16 |
|  | **Zn** | 0.49 | 0.23 | 0.26 | 0.24 | **0.021** | **1.07** |
|  | **In** | 0.55 | 0.38 | 0.17 | 0.15 | **0.018** | 1.18 |
|  | **Bi** | 0.42 | 0.36 | 0.06 | 0.04 | **0.016** | 1.34 |
|  | **Ga** | 0.45 | 0.30 | 0.16 | 0.13 | **0.026** | 1.20 |
|  | **Ge** | 0.40 | 0.36 | 0.04 | 0.02 | **0.020** | 1.36 |
|  | **Pb** | 0.64 | 0.42 | 0.22 | 0.21 | **0.013** | 1.10 |
|  | Cd | 0.57 | 0.28 | 0.29 | 0.30 | -0.014 | 1.02 |
| **Pt** | Ag | 0.46 | 0.29 | 0.17 | 0.17 | -0.002 | 1.19 |
|  | **Zn** | 0.52 | 0.28 | 0.24 | 0.24 | **0.003** | 1.10 |
|  | In | 0.51 | 0.42 | 0.09 | 0.15 | -0.058 | 1.29 |
|  | Bi | 0.38 | 0.38 | 0.01 | 0.04 | -0.034 | 1.40 |
|  | Ga | 0.44 | 0.34 | 0.10 | 0.13 | -0.028 | 1.29 |
|  | Ge | 0.33 | 0.38 | -0.05 | 0.02 | -0.071 | 1.46 |
|  | Pb | 0.59 | 0.43 | 0.16 | 0.21 | -0.045 | 1.18 |
|  | Cd | 0.56 | 0.33 | 0.23 | 0.30 | -0.072 | 1.10 |
| **Pd** | **Ag** | 0.44 | 0.27 | 0.17 | 0.17 | **0.005** | 1.18 |
|  | **Zn** | 0.50 | 0.26 | 0.24 | 0.24 | **0.004** | 1.09 |
|  | In | 0.53 | 0.39 | 0.14 | 0.15 | -0.006 | 1.21 |
|  | Bi | 0.37 | 0.36 | 0.01 | 0.04 | -0.031 | 1.41 |
|  | **Ga** | 0.46 | 0.32 | 0.14 | 0.13 | **0.007** | 1.23 |
|  | Ge | 0.39 | 0.38 | 0.01 | 0.02 | -0.007 | 1.38 |
|  | Pb | 0.61 | 0.42 | 0.19 | 0.21 | -0.019 | 1.15 |
|  | Cd | -2.99 | -3.00 | 0.01 | 0.30 | -0.293 | 1.41 |
| **Rh** | **Ag** | 0.47 | 0.28 | 0.19 | 0.17 | **0.020** | 1.16 |
|  | **Zn** | 0.56 | 0.29 | 0.27 | 0.24 | **0.030** | **1.07** |
|  | In | 0.52 | 0.40 | 0.12 | 0.15 | -0.034 | 1.25 |
|  | Bi | 0.44 | 0.42 | 0.02 | 0.04 | -0.019 | 1.37 |
|  | Ga | 0.46 | 0.34 | 0.13 | 0.13 | -0.004 | 1.26 |
|  | Ge | 0.38 | 0.41 | -0.02 | 0.02 | -0.044 | 1.44 |
|  | Pb | 0.64 | 0.46 | 0.18 | 0.21 | -0.028 | 1.15 |
|  | Cd | 0.58 | 0.33 | 0.25 | 0.30 | -0.050 | 1.09 |
| Au | Ag | 0.42 | 0.26 | 0.16 | 0.17 | -0.010 | 1.20 |
|  | Zn | 0.50 | 0.27 | 0.23 | 0.24 | -0.008 | 1.10 |
|  | In | 0.48 | 0.42 | 0.07 | 0.15 | -0.084 | 1.32 |
|  | Bi | 0.36 | 0.35 | 0.01 | 0.04 | -0.033 | 1.40 |
|  | Ga | 0.45 | 0.38 | 0.07 | 0.13 | -0.057 | 1.31 |
|  | Ge | 0.24 | 0.31 | -0.07 | 0.02 | -0.088 | 1.51 |
|  | Pb | 0.48 | 0.34 | 0.14 | 0.21 | -0.068 | 1.21 |
|  | Cd | -2.85 | -2.85 | 0.00 | 0.30 | -0.300 | 1.40 |
| **Al** | **Ag** | 0.52 | 0.34 | 0.18 | 0.17 | **0.008** | 1.17 |
|  | Zn | 0.41 | 0.25 | 0.16 | 0.24 | -0.083 | 1.21 |
|  | In | 0.34 | 0.31 | 0.03 | 0.15 | -0.121 | 1.37 |
|  | **Bi** | 0.45 | 0.38 | 0.07 | 0.04 | **0.029** | 1.32 |
|  | Ga | 0.31 | 0.26 | 0.05 | 0.13 | -0.082 | 1.35 |
|  | Ge | 0.17 | 0.27 | -0.10 | 0.02 | -0.122 | 1.56 |
|  | Pb | 0.55 | 0.35 | 0.20 | 0.21 | -0.011 | 1.13 |
|  | Cd | 0.43 | 0.30 | 0.13 | 0.30 | -0.170 | 1.24 |
| **Sb** | Ag | 0.49 | 0.32 | 0.17 | 0.17 | -0.002 | 1.19 |
|  | Zn | 0.49 | 0.33 | 0.16 | 0.24 | -0.076 | 1.22 |
|  | In | 0.37 | 0.35 | 0.01 | 0.15 | -0.137 | 1.42 |
|  | Bi | 0.40 | 0.37 | 0.03 | 0.04 | -0.011 | 1.40 |
|  | Ga | 0.44 | 0.38 | 0.06 | 0.13 | -0.074 | 1.35 |
|  | Ge | 0.38 | 0.39 | -0.01 | 0.02 | -0.031 | 1.41 |
|  | **Pb** | 0.57 | 0.36 | 0.22 | 0.21 | **0.006** | 1.11 |
|  | Cd | 0.43 | 0.34 | 0.09 | 0.30 | -0.210 | 1.29 |
| Sn | Ag | 0.81 | 0.65 | 0.17 | 0.17 | -0.005 | 1.19 |
|  | Zn | 0.47 | 0.32 | 0.15 | 0.24 | -0.093 | 1.21 |
|  | In | 0.36 | 0.38 | -0.01 | 0.15 | -0.162 | 1.43 |
|  | Bi | 0.44 | 0.43 | 0.01 | 0.04 | -0.030 | 1.37 |
|  | Ga | 0.38 | 0.35 | 0.03 | 0.13 | -0.099 | 1.35 |
|  | Ge | 0.31 | 0.36 | -0.05 | 0.02 | -0.070 | 1.46 |
|  | Pb | 0.58 | 0.40 | 0.18 | 0.21 | -0.030 | 1.15 |
|  | Cd | 0.43 | 0.36 | 0.08 | 0.30 | -0.222 | 1.31 |

**Table S9.** The electron configuration of the outermost shell in a metal.

| 3 | | 4 | | 5 | | 6 | | 7 | | 8 | | 9 | | | 10 | | 11 | | 12 | | 13 | | 14 | | 15 | |
| --- | --- | --- | --- | --- | --- | --- | --- | --- | --- | --- | --- | --- | --- | --- | --- | --- | --- | --- | --- | --- | --- | --- | --- | --- | --- | --- |
| Mg | |  | |  | |  | |  | |  | |  | | |  | |  | |  | | Al | |  | |  | |
| 3s^2^ | |  | |  | |  | |  | |  | |  | | |  | |  | |  | | 3s² 3p¹ | |  | |  | |
| Sc | | Ti | | V | | Cr | | Mn | | Fe | | Co | | | Ni | | Cu | | Zn | | Ga | | Ge | |  | |
| 4s² 3d¹ | | 4s² 3d² | | 4s² 3d³ | | 4s¹ 3d⁵ | | 4s² 3d⁵ | | 4s² 3d⁶ | | 4s² 3d⁷ | | | 4s² 3d⁸ | | 4s¹3d¹⁰ | | 4s²3d¹⁰ | | 4s² 3d¹⁰ 4p¹ | | 4s² 3d¹⁰ 4p² | |  | |
| Y | | Zr | | Nb | | Mo | | Tc | | Ru | | Rh | | | Pd | | Ag | | Cd | | In | | Sn | | Sb | |
| 5s² 4d¹ | | 5s² 4d² | | 5s¹ 4d^4^ | | 5s¹ 4d⁵ | | 5s² 4d⁵ | | 5s¹ 4d⁷ | | 5s¹4d⁸ | | | 4d¹⁰ | | 5s¹ 4d¹⁰ | | 5s²4d¹⁰ | | 5s² 4d¹⁰ 5p¹ | | 5s² 4d¹⁰ 5p² | | 5s² 4d¹⁰ 5p³ | |
| Lanthanide metals | | Hf | | Ta | | W | | Re | | Os | | Ir | | | Pt | | Au | | Hg | | Tl | | Pb | | Bi | |
|  |  | 6s² 4f¹⁴ 5d² | | 6s² 4f¹⁴ 5d³ | | 6s² 4f¹⁴ 5d⁴ | | 6s² 4f¹⁴ 5d⁵ | | 6s² 4f¹⁴ 5d⁶ | | 6s² 4f¹⁴ 5d⁷ | | | 6s¹ 4f¹⁴ 5d⁹ | | 6s¹ 4f¹⁴ 5d¹⁰ | | 6s² 4f¹⁴ 5d¹⁰ | | 6s² 4f¹⁴ 5d¹⁰ 6p¹ | | 6s² 4f¹⁴ 5d¹⁰ 6p² | | 6s² 4f¹⁴ 5d¹⁰ 6p³ | |
| La | Ce | | Pr | | Nd | | Pm | | Sm | | Eu | | Gd | Tb | | Dy | | Ho | | Er | | Tm | | Yb | | Lu |
| 6s^2^5d^1^ | 6s^2^4f^1^5d^1^ | | 6s^2^4f^3^ | | 6s^2^4f^4^ | | 6s^2^4f^5^ | | 6s^2^4f^6^ | | 6s^2^4f^7^ | | 6s^2^4f^7^5d^1^ | 6s^2^4f^9^ | | 6s^2^4f^10^ | | 6s^2^4f^11^ | | 6s^2^4f^12^ | | 6s^2^4f^13^ | | 6s^2^4f^14^ | | 6s^2^4f^14^5d^1^ |

**Table S10.** Feature parameters for machine learning of E^ads^(CO*_M_) prediction.

| 1 | X | the Pauling electronegativity of dopants (M) |
| --- | --- | --- |
| 2 | E_fi_ | the first ionization energy of M |
| 3 | R | atomic radius of M |
| 4 | N | the column number of dopants in periodic system of M |
| 5 | n_d_ | d-electron count of M |
| 6 | n_v_ | valence electron of M |
| 7 | Δn_M_ | the electron transfers number of M |
| 8 | Δn_Cu1_ | the electron transfers number of Cu1 |
| 9 | θd_M_ | d-band center of dopants |
| 10 | θd_Cu1_ | d-band center of first-nearest-neighbor copper (Cu1) |
| 11 | d_M_ | average distance of M-Cu1 |
| 12 | d_Cu1_ | average distance of Cu1-Cu1 |

**Table S11.** EXAFS fitting parameters at the Y k-edge for various samples

| Sample | Shell | N ^a^ | R (Å) ^b^ | σ^2^ (Å^2^·10^-3^) ^c^ | ΔE_0_ (eV) ^d^ | *R* factor (%) |
| --- | --- | --- | --- | --- | --- | --- |
| YCu-SAA | Y-O | 4.9 | 2.33 | 1.6 | 0.61 | 0.6 |
|  | Y-Cu | 5.7 | 2.82 | 18.5 | 0.61 |  |
| Y foil | Y-Y | 12 | 3.59 | 3.6 | 2.17 | 1.4 |
| Y_2_O_3_ | Y-O | 6 | 2.29 | 7.1 | -4.78 | 1.4 |
|  | Y-Y | 6 | 3.53 | 4.8 | -9.95 |  |

*^a^N*: coordination numbers;

*^b^R*: bond distance;

*^c^σ*^2^: Debye-Waller factors;

*^d^*Δ*E*_0_: the inner potential correction.

*R* factor: goodness of fit.

**References**

1. Kresse; Furthmuller, Efficient iterative schemes for ab initio total-energy calculations using a plane-wave basis set. *Phys. Rev. B* **1996,** *54* (16), 11169-11186.

2. Blöchl, P. E., Projector augmented-wave method. *Phys. Rev. B* **1994,** *50* (24), 17953-17979.

3. Hammer, B.; Hansen, L. B.; Nørskov, J. K., Improved adsorption energetics within density-functional theory using revised Perdew-Burke-Ernzerhof functionals. *Phys. Rev. B* **1999,** *59* (11), 7413-7421.

4. Henkelman, G.; Uberuaga, B. P.; Jónsson, H., A climbing image nudged elastic band method for finding saddle points and minimum energy paths. *J. Chem. Phys.* **2000,** *113* (22), 9901-9904.

5. Henkelman, G.; Jónsson, H., A dimer method for finding saddle points on high dimensional potential surfaces using only first derivatives. *J. Chem. Phys.* **1999,** *111* (15), 7010-7022.

6. Greeley, J.; Norskov, J. K., Electrochemical dissolution of surface alloys in acids: Thermodynamic trends from first-principles calculations. *Electrochim. Acta* **2007,** *52* (19), 5829-5836.

7. Sundararaman, R.; Letchworth-Weaver, K.; Schwarz, K. A.; Gunceler, D.; Ozhabes, Y.; Arias, T. A., JDFTx: Software for joint density-functional theory. *SoftwareX* **2017,** *6*, 278-284.

8. Sundararaman, R.; Goddard, W. A., The charge-asymmetric nonlocally determined local-electric (CANDLE) solvation model. *J. Chem. Phys.* **2015,** *142* (6), 064107.

9. Mathew, K.; Sundararaman, R.; Letchworth-Weaver, K.; Arias, T. A.; Hennig, R. G., Implicit solvation model for density-functional study of nanocrystal surfaces and reaction pathways. *J. Chem. Phys.* **2014,** *140* (8), 084106.

10. Huang, S. D.; Shang, C.; Kang, P. L.; Zhang, X. J.; Liu, Z. P., LASP: Fast global potential energy surface exploration. *Wiley Interdiscip. Rev.-Comput. Mol. Sci.* **2019,** *9*, e1415.

11. Huang, S. D.; Shang, C.; Zhang, X. J.; Liu, Z. P., Material discovery by combining stochastic surface walking global optimization with a neural network. *Chem. Sci.* **2017,** *8* (9), 6327-6337.

12. Santatiwongchai, J.; Faungnawakij, K.; Hirunsit, P., Comprehensive Mechanism of CO_2_ Electroreduction toward Ethylene and Ethanol: The Solvent Effect from Explicit WaterCu(100) Interface Models. *ACS Catal.* **2021,** *11* (15), 9688-9701.

13. Goodpaster, J. D.; Bell, A. T.; Head-Gordon, M., Identification of Possible Pathways for C-C Bond Formation during Electrochemical Reduction of CO_2_: New Theoretical Insights from an Improved Electrochemical Model. *J. Phys. Chem. Lett.* **2016,** *7* (8), 1471-1477.

14. Cheng, T.; Xiao, H.; Goddard, W. A., Free-Energy Barriers and Reaction Mechanisms for the Electrochemical Reduction of CO on the Cu(100) Surface, Including Multiple Layers of Explicit Solvent at pH 0. *J. Phys. Chem. Lett.* **2015,** *6* (23), 4767-4773.

15. Zhang, J.; Guo, C. X.; Fang, S. S.; Zhao, X. T.; Li, L.; Jiang, H. Y.; Liu, Z. Y.; Fan, Z. Q.; Xu, W. G.; Xiao, J. P.; Zhong, M., Accelerating electrochemical CO_2_ reduction to multi-carbon products via asymmetric intermediate binding at confined nanointerfaces. *Nat. Commun.* **2023,** *14*, 1298.

16. Zhang, D.; Li, H., The hidden engine of AI in electrocatalysis: Databases and knowledge graphs at work. *Molecular Chemistry & Engineering* **2025,** *1* (1), 100003.

17. Wang, Q.; Yang, F.; Wang, Y.; Zhang, D.; Sato, R.; Zhang, L.; Cheng, E. J.; Yan, Y.; Chen, Y.; Kisu, K.; Orimo, S.-i.; Li, H., Unraveling the Complexity of Divalent Hydride Electrolytes in Solid-State Batteries via a Data-Driven Framework with Large Language Model. *Angew. Chem.-Int. Edit.* **2025,** *64*, e202506573.

18. Hori, Y.; Takahashi, I.; Koga, O.; Hoshi, N., Electrochemical reduction of carbon dioxide at various series of copper single crystal electrodes. *J. Mol. Catal. A-Chem.* **2003,** *199*, 39-47.

19. Wang, X. Y.; Chen, Y. J.; Li, F.; Miao, R. K.; Huang, J. E.; Zhao, Z. L.; Li, X. Y.; Dorakhan, R.; Chu, S. L.; Wu, J. H.; Zheng, S. X.; Ni, W. Y.; Kim, D.; Park, S.; Liang, Y. X.; Ozden, A.; Ou, P. F.; Hou, Y.; Sinton, D.; Sargent, E. H., Site-selective protonation enables efficient carbon monoxide electroreduction to acetate. *Nat. Commun.* **2024,** *15*, 616.

20. Li, Z.; Wang, P.; Lyu, X.; Kondapalli, V. K. R.; Xiang, S.; Jimenez, J. D.; Ma, L.; Ito, T.; Zhang, T.; Raj, J.; Fang, Y.; Bai, Y.; Li, J.; Serov, A.; Shanov, V.; Frenkel, A. I.; Senanayake, S. D.; Yang, S.; Senftle, T. P.; Wu, J., Directing CO_2_ electroreduction pathways for selective C_2_ product formation using single-site doped copper catalysts. *Nat. Chem. Eng.* **2024,** *1* (2), 159-169.

21. Sun, Q.; Zhao, Y.; Tan, X.; Jia, C.; Su, Z.; Meyer, Q.; Ahmed, M. I.; Zhao, C., Atomically Dispersed Cu-Au Alloy for Efficient Electrocatalytic Reduction of Carbon Monoxide to Acetate. *ACS Catal.* **2023,** *13* (8), 5689-5696.

22. Jeong, S.; Huang, C. L.; Levell, Z.; Skalla, R. X.; Hong, W.; Escorcia, N. J.; Losovyj, Y.; Zhu, B. X.; Butrum-Griffith, A. N.; Liu, Y.; Li, C. W.; Hickey, D. R.; Liu, Y. Y.; Ye, X. C., Facet-Defined Dilute Metal Alloy Nanorods for Efficient Electroreduction of CO_2_ to *n*-Propanol. *J. Am. Chem. Soc.* **2024,** *146* (7), 4508-4520.

23. Du, C.; Mills, J. P.; Yohannes, A. G.; Wei, W.; Wang, L.; Lu, S. Y.; Lian, J. X.; Wang, M. Y.; Guo, T.; Wang, X. Y.; Zhou, H.; Sun, C. J.; Wen, J. Z.; Kendall, B.; Couillard, M.; Guo, H. S.; Tan, Z. C.; Siahrostami, S.; Wu, Y. A., Cascade electrocatalysis via AgCu single-atom alloy and Ag nanoparticles in CO_2_ electroreduction toward multicarbon products. *Nat. Commun.* **2023,** *14*, 6142.

24. Zhang, Z. N.; Fang, Q.; Yang, X.; Zuo, S. W.; Cheng, T.; Yamauchi, Y.; Tang, J., Additives-Modified Electrodeposition for Synthesis of Hydrophobic Cu/Cu_2_O with Ag Single Atoms to Drive CO_2_ Electroreduction. *Adv. Mater.* **2025,** *37*, 2411498.

25. Li, Y. G. C.; Wang, Z. Y.; Yuan, T. G.; Nam, D. H.; Luo, M. C.; Wicks, J.; Chen, B.; Li, J.; Li, F. W.; de Arguer, F. P. G.; Wang, Y.; Dinh, C. T.; Voznyy, O.; Sinton, D.; Sargent, E. H., Binding Site Diversity Promotes CO_2_ Electroreduction to Ethanol. *J. Am. Chem. Soc.* **2019,** *141* (21), 8584-8591.

26. Li, S. Y.; Zhang, G.; Ma, X.; Gao, H.; Fu, D. L.; Wang, T.; Zeng, J. R.; Zhao, Z. J.; Zhang, P.; Gong, J. L., Atomically Isolated Pd Sites Promote Electrochemical CO Reduction to Acetate through a Protonation-Regulated Mechanism. *J. Am. Chem. Soc.* **2024,** *146* (46), 31927-31934.

27. Wang, S.; Li, F.; Zhao, J.; Zeng, Y.; Li, Y.; Lin, Z.-Y.; Lee, T.-J.; Liu, S.; Ren, X.; Wang, W.; Chen, Y.; Hung, S.-F.; Lu, Y.-R.; Cui, Y.; Yang, X.; Li, X.; Huang, Y.; Liu, B., Manipulating C-C coupling pathway in electrochemical CO_2_ reduction for selective ethylene and ethanol production over single-atom alloy catalyst. *Nat. Commun.* **2024,** *15*, 10247.

28. Cheng, D. F.; Zhang, G.; Li, L. L.; Shi, X. C.; Zhu, W. J.; Yuan, X. T.; Moskaleva, L.; Zhang, P.; Zhao, Z. J.; Gong, J. L., Why do CuAl catalysts outperform in CO_2_ electro-reduction to C_2_H_4_? *Sci. China-Chem.* **2025,** *68* (2), 763-771.

29. Wang, M.; Fang, M. H.; Liu, Y. X.; Chen, C. J.; Zhang, Y. C.; Jia, S. Q.; Wu, H. H.; He, M. Y.; Han, B. X., Enhanced Intermediates Inter-migration on Ag Single-Atom Alloys for Boosting Multicarbon Product Selectivity in CO_2_ Electroreduction. *J. Am. Chem. Soc.* **2025,** *147* (19), 16450-16458.

30. Wang, D. Y.; Li, Y. Z.; Geng, S.; Li, R. P.; Chen, K., Boosting Electrocatalytic CO_2_ Reduction to Multi-Carbon Products via Modulated Asymmetric Cu Sites. *Adv. Funct. Mater.* **2025,** *35*, 2503497.

31. Guo, X. H.; Liu, T. F.; Song, Y. P.; Li, R. T.; Wei, P. F.; Liao, Z. Q.; Wu, Z. C.; Gao, D. F.; Fu, Q.; Wang, G. X.; Bao, X. H., Selective CO Electroreduction to Multicarbon Oxygenates Over Atomically Dispersed Cu-Ag Sites in Alkaline Membrane Electrode Assembly Electrolyzer. *Angew. Chem.-Int. Edit.* **2025,** *64*, e202507062.

32. Yang, C. P.; Ko, B. H.; Hwang, S.; Liu, Z. Y.; Yao, Y. G.; Luc, W.; Cui, M. J.; Malkani, A. S.; Li, T. Y.; Wang, X. Z.; Dai, J. Q.; Xu, B. J.; Wang, G. F.; Su, D.; Jiao, F.; Hu, L. B., Overcoming immiscibility toward bimetallic catalyst library. *Sci. Adv.* **2020,** *6*, eaaz6844.

33. Yang, Y. S.; Zhang, J. L.; Tan, Z. H.; Yang, J.; Wang, S.; Li, M. L.; Su, Z. Z., Highly Selective Production of C_2+_ Oxygenates from CO_2_ in Strongly Acidic Condition by Rough Ag-Cu Electrocatalyst. *Angew. Chem.-Int. Edit.* **2024,** *63*, e202408873.

34. Huang, L.; Gao, G.; Zhao, J.; Roberts, W. L.; Lu, X., Electrocatalytic upcycling of high-pressure captured CO_2_ to ethylene. *Nat. Catal.* **2025,** *8*, 968-976.

35. Zhou, D. W.; Chen, C. J.; Zhang, Y. C.; Wang, M.; Han, S. T.; Dong, X.; Yao, T.; Jia, S. Q.; He, M. Y.; Wu, H. H.; Han, B. X., Cooperation of Different Active Sites to Promote CO_2_ Electroreduction to Multi-carbon Products at Ampere-Level. *Angew. Chem.-Int. Edit.* **2024,** *63*, e202400439.

36. Crandall, B. S.; Qi, Z.; Foucher, A. C.; Weitzner, S. E.; Akhade, S. A.; Liu, X.; Kashi, A. R.; Buckley, A. K.; Ma, S. C.; Stach, E. A.; Varley, J. B.; Jiao, F.; Biener, J., Cu Based Dilute Alloys for Tuning the C_2+_ Selectivity of Electrochemical CO_2_ Reduction. *Small* **2024,** *20*, 2401656.

37. Li, X. T.; Qin, M. K.; Wu, X. J.; Lv, X. Z.; Wang, J. H.; Wang, Y.; Wu, H. B., Enhanced CO Affinity on Cu Facilitates CO_2_ Electroreduction toward Multi-Carbon Products. *Small* **2023,** *19*, 2302530.

38. Nie, S. Y.; Wu, L.; Liu, Q. D.; Wang, X., Entropy-Derived Synthesis of the CuPd Sub-1nm Alloy for CO_2_-to-acetate Electroreduction. *J. Am. Chem. Soc.* **2024,** *146* (43), 29364-29372.

39. Chhetri, M.; Wan, M. Y.; Jin, Z. H.; Yeager, J.; Sandor, C.; Rapp, C.; Wang, H.; Lee, S.; Bodenschatz, C. J.; Zachman, M. J.; Che, F. L.; Yang, M., Dual-site catalysts featuring platinum-group-metal atoms on copper shapes boost hydrocarbon formations in electrocatalytic CO_2_ reduction. *Nat. Commun.* **2023,** *14*, 3075.

40. Xiao, J. W.; You, S. Y.; Huang, H. S.; Liang, S. Y.; Xie, W. F.; Li, M.; Zhang, T. Y.; Wang, Q., Regulation of Cu-MOF reconstruction for enhanced CO_2_ electroreduction. *Appl. Catal. B-Environ. Energy* **2025,** *375*, 125412.

41. Hou, T. L.; Zhu, J. X.; Gu, H. F.; Li, X. Y.; Sun, Y. Q.; Hua, Z.; Shao, R. W.; Chen, C.; Hu, B. T.; Mai, L. Q.; Chen, S. H.; Wang, D. S.; Zhang, J. T., Switching CO_2_ Electroreduction toward C_2+_ Products and CH_4_ by Regulating the Dimerization and Protonation in Platinum/Copper Catalysts. *Angew. Chem.-Int. Edit.* **2025,** *64*, e202424749.

42. Chen, Z. P.; Zhao, Y. S.; Liu, G.; Zhang, H. N.; Yan, Y.; Ke, Q. P.; Liu, M. K.; Liu, L. C.; Lin, Z. Q., Turning the Selectivity of CO Electroreduction from Acetate to Ethanol by Alloying FCC-Phased Cu with Atomically Dispersed Mn Atoms. *Nano Lett.* **2025,** *25* (16), 6771-6779.

43. Kim, B.; Tan, Y. C.; Ryu, Y.; Jang, K.; Abbas, H. G.; Kang; Choi, H.; Lee, K. S.; Park, S.; Kim, W.; Choi, P. P.; Ringe, S.; Oh, J., Trace-Level Cobalt Dopants Enhance CO_2_ Electroreduction and Ethylene Formation on Copper. *ACS Energy Lett.* **2023,** *8* (8), 3356-3364.

44. Liu, J. C.; Wen, Y.; Yan, W.; Huang, Z. L.; Liu, X. Z.; Huang, X.; Zhan, C. H.; Zhang, Y. Q.; Huang, W. H.; Pao, C. W.; Hu, Z. W.; Su, D.; Xie, S. J.; Wang, Y.; Han, J. J.; Xiong, H. F.; Huang, X. Q.; Chen, N. J., Single-atom mediated crystal facet engineering for the exceptional production of acetate in CO electrolysis. *Energy Environ. Sci.* **2025,** *18* (9), 4396-4404.

45. Song, H.; Tan, Y. C.; Kim, B.; Ringe, S.; Oh, J., Tunable Product Selectivity in Electrochemical CO_2_ Reduction on Well-Mixed Ni-Cu Alloys. *ACS Appl. Mater. Interfaces* **2021,** *13* (46), 55272-55280.

46. Zhang, X. L.; Liu, C. W.; Zhao, Y.; Li, L. B.; Chen, Y.; Raziq, F.; Qiao, L.; Guo, S. X.; Wang, C. Y.; Wallace, G. G.; Bond, A. M.; Zhang, J., Atomic nickel cluster decorated defect-rich copper for enhanced C_2_ product selectivity in electrocatalytic CO_2_ reduction. *Appl. Catal. B-Environ.* **2021,** *291*, 120030.

47. Jin, C. H.; Lin, Y.; Wang, Y. A.; Shi, J. B.; Li, R.; Liu, Y. J.; Yue, Z. Y.; Leng, K. Y.; Zhao, Y. F.; Wang, Y.; Han, X.; Qu, Y. T., Engineering Atom-Scale Cascade Catalysis via Multi-Active Site Collaboration for Ampere-Level CO_2_ Electroreduction to C_2+_ Products. *Adv. Mater.* **2025,** *37*, 2412658.

48. Wu, Z. T.; Meng, N. N.; Yang, R.; Chen, M. X.; Pan, J. H.; Chi, S. J.; Wu, C.; Xi, S. B.; Liu, Y.; Ou, Y. Q.; Wu, W. Y.; Han, S. H.; Zhang, B.; Yang, Q. H.; Loh, K. P., Boosting C_2+_ Alcohols Selectivity and Activity in High-Current CO Electroreduction using Synergistic Cu/Zn Co-Catalysts. *Angew. Chem.-Int. Edit.* **2025,** *64*, e202420283.

49. Zhang, L. B.; Feng, J. Q.; Wu, L. M.; Ma, X. D.; Song, X. N.; Jia, S. H.; Tan, X. X.; Jin, X. Y.; Zhu, Q. G.; Kang, X. C.; Ma, J.; Qian, Q. L.; Zheng, L. R.; Sun, X. F.; Han, B. X., Oxophilicity-Controlled CO_2_ Electroreduction to C_2+_ Alcohols over Lewis Acid Metal-Doped Cu^d+^ Catalysts. *J. Am. Chem. Soc.* **2023,** *145* (40), 21945-21954.

50. Zhong, M.; Tran, K.; Min, Y. M.; Wang, C. H.; Wang, Z. Y.; Dinh, C. T.; De Luna, P.; Yu, Z. Q.; Rasouli, A. S.; Brodersen, P.; Sun, S.; Voznyy, O.; Tan, C. S.; Askerka, M.; Che, F. L.; Liu, M.; Seifitokaldani, A.; Pang, Y. J.; Lo, S. C.; Ip, A.; Ulissi, Z.; Sargent, E. H., Accelerated discovery of CO_2_ electrocatalysts using active machine learning. *Nature* **2020,** *581*, 178-183.

51. Li, P. S.; Bi, J. H.; Liu, J. Y.; Wang, Y.; Kang, X. C.; Sun, X. F.; Zhang, J. L.; Liu, Z. M.; Zhu, Q. G.; Han, B. X., p-d Orbital Hybridization Induced by p-Block Metal-Doped Cu Promotes the Formation of C_2+_ Products in Ampere-Level CO_2_ Electroreduction. *J. Am. Chem. Soc.* **2023,** *145* (8), 4675–4682.

52. Kanase, R. S.; Lee, K. B.; Arunachalam, M.; Sivasankaran, R. P.; Oh, J.; Kang, S. H., Nanostructure engineering of Cu electrocatalyst for the selective C_2+_ hydrocarbons in electrochemical CO_2_ reduction. *Appl. Surf. Sci.* **2022,** *584*, 152518.

53. Chen, L.; Chen, J. M.; Fu, W. W.; Chen, J. Y.; Wang, D.; Xiao, Y. K.; Xi, S. B.; Ji, Y. F.; Wang, L., Energy-efficient CO_(2)_ conversion to multicarbon products at high rates on CuGa bimetallic catalyst. *Nat. Commun.* **2024,** *15*, 7053.

54. Wang, J. H.; Ji, Q. Y.; Zang, H.; Zhang, Y.; Liu, C. J.; Yu, N.; Geng, B. Y., Atomically Dispersed Ga Synergy Lewis Acid-Base Pairs in F-doped Mesoporous Cu_2_O for Efficient Eletroreduction of CO_2_ to C_2+_ Products. *Adv. Funct. Mater.* **2024,** *34*, 2404274.

55. Chen, Y. J.; Wang, X. Y.; Li, X. Y.; Miao, R. K.; Dong, J. C.; Zhao, Z. L.; Liu, C. H.; Huang, J. E.; Wu, J. H.; Chu, S. L.; Ni, W. Y.; Guo, Z. M.; Xu, Y.; Ou, P. F.; Xu, B. J.; Hou, Y.; Sinton, D.; Sargent, E. H., Electrified synthesis of *n*-propanol using a dilute alloy catalyst. *Nat. Catal.* **2025,** *8* (3), 239-247.

56. Liu, Y. J.; Yue, Z. Y.; Jin, C. H.; Zheng, L. R.; Shi, J. B.; Li, D. D.; Wang, Y.; Bai, J. B.; Leng, K. Y.; Wang, W. T.; Qu, Y. T.; Li, Q. Y., Isolated Tin Enhanced CO Coverage-Regulation on Sn_1_Cu Alloy for Selective CO_2_ Electroreduction to C_2+_ Products. *Small* **2025,** *21* (8), 2409259.

57. Pan, Y.; Duan, G. Y.; Li, R. L.; Fang, J.; Yu, Z. F.; Xu, B. H., Regulating selectivity of high-rate CO electroreduction on bimetallic catalysts. *Appl. Catal. B-Environ. Energy* **2025,** *378*, 125584.

58. Ma, X. D.; Song, X. N.; Zhang, L. B.; Wu, L. M.; Feng, J. Q.; Jia, S. H.; Tan, X. X.; Xu, L.; Sun, X. F.; Han, B. X., Stabilizing Cu^0^-Cu^+^ sites by Pb-doping for highly efficient CO_2_ electroreduction to C_2_ products. *Green Chem.* **2023,** *25* (19), 7635-7641.

59. Cao, Y. C.; Chen, S. Y.; Bo, S. W.; Fan, W. J.; Li, J. N.; Jia, C. M.; Zhou, Z.; Liu, Q. H.; Zheng, L. R.; Zhang, F. X., Single Atom Bi Decorated Copper Alloy Enables C-C Coupling for Electrocatalytic Reduction of CO_2_ into C_2+_ Products. *Angew. Chem.-Int. Edit.* **2023,** *62*, e202303048.

60. Li, P. S.; Liu, J. Y.; Wang, Y.; Zhang, X. D.; Hou, Y. Q.; Zhang, Y. C.; Sun, X. F.; Kang, X. C.; Zhu, Q. G.; Han, B. X., Manipulation of Oxygen Species on an Antimony-Modified Copper Surface to Tune the Product Selectivity in CO_2_ Electroreduction. *J. Am. Chem. Soc.* **2024,** *146* (38), 26525-26533.

61. Xu, Y. N.; Li, J. H.; Wu, J. C.; Li, W. B.; Yang, Y. W.; Wu, H. R.; Fu, H. Q.; Zhu, M. H.; Wang, X. L.; Dai, S.; Lian, C.; Liu, P. F.; Yang, H. G., Orbital Matching Mechanism-Guided Synthesis of Cu-Based Single Atom Alloys for Acidic CO_2_ Electroreduction. *Adv. Mater.* **2025,** *37* (18), 2500343.

62. Chen, R. Z.; Jiang, Y. H.; Zhu, Y. H.; Zhang, L.; Li, Y. H.; Li, C. Z., Atomically Dispersed Scandium in Cuprous Oxide Weakens *CO Adsorption to Boost Carbon Dioxide Electroreduction Toward C_2_ Products. *Adv. Funct. Mater.* **2025,** *35* (9), 2415940.

63. Feng, J. Q.; Wu, L. M.; Song, X. N.; Zhang, L. B.; Jia, S. H.; Ma, X. D.; Tan, X. X.; Kang, X. C.; Zhu, Q. G.; Sun, X. F.; Han, B. X., CO_2_ electrolysis to multi-carbon products in strong acid at ampere-current levels on La-Cu spheres with channels. *Nat. Commun.* **2024,** *15*, 4821.

64. Guo, Z. Y.; Zhu, H. W.; Yan, Z. J.; Lei, L.; Wang, D. G.; Xi, Z. Y.; Lian, Y. R.; Yu, J. H.; Fow, K. L.; Do, H.; Hirst, J. D.; Wu, T.; Xu, M. X., Manipulating adsorbed hydrogen on lanthanum-modified CuO_x_: Industrial-current-density CO_2_ electroreduction to C_2+_ products or CH_4_. *Appl. Catal. B-Environ. Energy* **2025,** *364*, 124839.

65. Zhao, J.; Zhang, P.; Yuan, T. H.; Cheng, D. F.; Zhen, S. Y.; Gao, H.; Wang, T.; Zhao, Z. J.; Gong, J. L., Modulation of *CH_X_O Adsorption to Facilitate Electrocatalytic Reduction of CO_2_ to CH_4_ over Cu-Based Catalysts. *J. Am. Chem. Soc.* **2023,** *145* (12), 6622-6627.

66. Liu, X.; Liu, T.; Ouyang, T.; Deng, J. G.; Liu, Z. Q., Ce^3+^/Ce^4+^ Ion Redox Shuttle Stabilized Cu^δ+^ for Efficient CO_2_ Electroreduction to C_2_H_4_. *Angew. Chem.-Int. Edit.* **2025,** *64*, e202419796.

67. Yang, X. P.; Wu, Z. Z.; Li, Y. C.; Sun, S. P.; Zhang, Y. C.; Duanmu, J. W.; Lu, P. G.; Zhang, X. L.; Gao, F. Y.; Yang, Y.; Wang, Y. H.; Yu, P. C.; Li, S. K.; Gao, M. R., Atomically dispersed cerium on copper tailors interfacial water structure for efficient CO-to-acetate electroreduction. *Nat. Commun.* **2025,** *16*, 2811.

68. Wang, M. D.; Shu, M. X.; Long, M.; Shan, W. Z.; Wang, H. M., Efficient Conversion of CO_2_ to Ethanol by Utilizing the Topological Surface States of Rare-Earth Cuprates. *Nano Lett.* **2025,** *25* (7), 2732-2740.

69. Xiao, Y. T.; Yu, F. Q.; Xia, C. F.; Zhu, D. Y.; Chen, J. W.; Liu, N. J.; Zhao, Y. Y.; Qi, R. J.; Guo, W.; You, B.; Yao, T.; Pang, Y. J.; Wang, Z. Y.; Wang, H. M.; Song, F.; Xia, B. Y., Asymmetric CO-CHO Coupling over Pr Single-Atom Alloy Enables Industrial-Level Electrosynthesis of Ethylene. *J. Am. Chem. Soc.* **2025,** *147* (18), 15654-15665.

70. Feng, J. Q.; Wu, L. M.; Liu, S. J.; Xu, L.; Song, X. N.; Zhang, L. B.; Zhu, Q. G.; Kang, X. C.; Sun, X. F.; Han, B. X., Improving CO_2_-to-C_2+_ Product Electroreduction Efficiency *via* Atomic Lanthanide Dopant-Induced Tensile-Strained CuO_x_ Catalysts. *J. Am. Chem. Soc.* **2023,** *145* (17), 9857-9866.

71. Jia, S. Q.; Cheng, H. L.; Zhu, Q. G.; Chen, X.; Xue, C.; Deng, T.; Dong, M. K.; Xia, Z. H.; Jiao, J. P.; Chen, C. J.; Wu, H. H.; He, M. Y.; Han, B. X., Tuning Multi-Active Sites in Cu Catalyst via Ag/Ni Doping for Enhanced CO_2_ Electroreduction to C_2+_ Products. *Angew. Chem.-Int. Edit.* **2025,** *64*, e202501833.
